# A Lysosome-Targeted Tetrazine for Organelle-Specific Click-to-Release Chemistry in Antigen Presenting Cells

**DOI:** 10.1021/jacs.3c02139

**Published:** 2023-06-03

**Authors:** Nina A.M. Ligthart, Mark A.R. de Geus, Merel A.T. van de Plassche, Diana Torres García, Marjolein M.E. Isendoorn, Luuk Reinalda, Daniëlle Ofman, Tyrza van Leeuwen, Sander I. van Kasteren

**Affiliations:** Leiden Institute of Chemistry and The Institute for Chemical Immunology, Leiden University, Einsteinweg 55, 2333 CC Leiden, The Netherlands

## Abstract

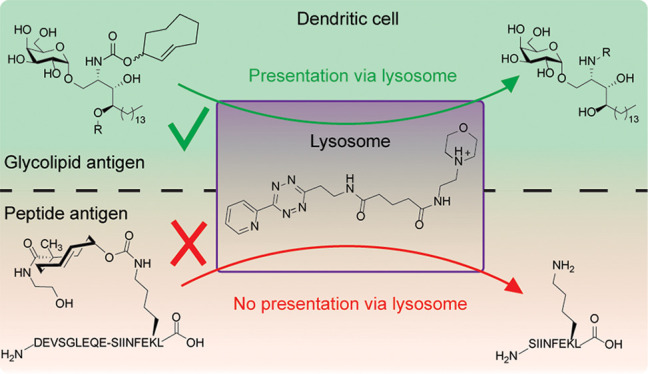

Bioorthogonal deprotections are readily used to control biological function in a cell-specific manner. To further improve the spatial resolution of these reactions, we here present a lysosome-targeted tetrazine for an organelle-specific deprotection reaction. We show that *trans*-cyclooctene deprotection with this reagent can be used to control the biological activity of ligands for invariant natural killer T cells in the lysosome to shed light on the processing pathway in antigen presenting cells. We then use the lysosome-targeted tetrazine to show that long peptide antigens used for CD8^+^ T cell activation do not pass through this organelle, suggesting a role for the earlier endosomal compartments for their processing.

## Introduction

Bioorthogonal chemistries are the family of chemical conversions that can be performed in a living system with a high degree of selectivity.^1,2^ They can be broadly subdivided into bond-forming and bond-breaking reactions.^3,4^ The latter are often used to control the activation of molecules of interest in cellular systems,^3^ for example, to locally activate chemotherapeutics and other drugs,^5,6^ activate enzyme activities in living cells,^7−9^ and control the activation of T cells.^10^ Various bond-breaking methods are available for this: from metal-based reactions,^11,12^ to the inverse electron-demand Diels–Alder (IEDDA) pyridazine elimination (the Click-to-Release reaction). The latter is the reaction between a tetrazine and a *trans*-cyclooctene (TCO)^13^ with allylic substitution that results in the formation of a dihydropyridazine intermediate, which tautomerizes and subsequently releases the allylic substituent (colloquially known as the “click-to-release”-approach).^3−5,9,10,13−18^ It is particularly favorable, as a wide range of chemical functionalities can be protected/deprotected by it (amines,^14^ alcohols,^19,20^ and carboxylic acids^21^).^22^ It is also very fast and has a strong in vivo pedigree,^5,16,23^ with a clinical trial using this chemistry having started recently.^17,24^

A recent development in the Click-to-Release field has been the move toward higher spatial resolution of these reactions. For example, Fox and co-workers have performed an IEDDA ligation reaction using two-photon activation of a tetrazine with submicrometer resolution.^25^ Dzijak et al.^26^ and Zheng et al.^27^ reported a mitochondrion-restricted bioorthogonal deprotection reaction, which they achieved by using both a mitochondrion-targeted deprotecting agent and prodrug to achieve mitochondrion-localized deprotection. We felt these advances could offer an exciting opportunity to unravel the subcellular topology of certain biological processes.

Our interest in this regard lies in the role of the dendritic cell (DC) lysosome during presentation of different classes of antigen.^28,29^ For example, invariant natural killer T cells (iNKTs) are activated by DCs presenting glycolipid antigens on CD1d receptors,^30,31^ and CD8^+^ T cells are activated by peptide antigens presented by these cells.^32^ Tools to precisely study the subcellular topology of the processes leading to the activation of these cells do not yet exist, but are highly important, as the precise contribution of the lysosome to these processes can be controversial. To study this, an organelle-specific bioorthogonal deprotection chemistry is required, without the requisite that both reagents are pretargeted to the organelle of interest.

**Figure 1:**
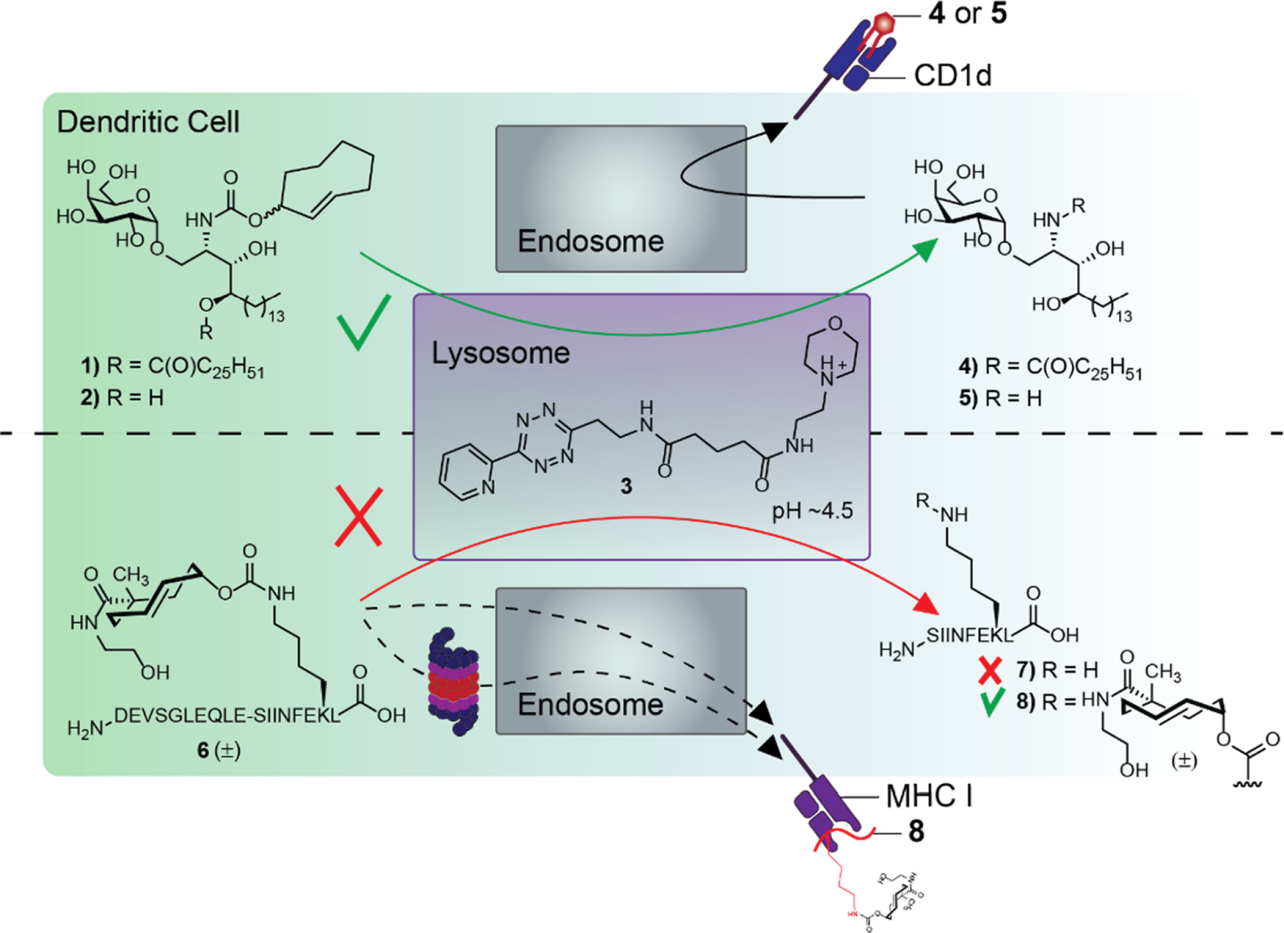
Bioorthogonal chemistry to study the subcellular routing before antigen presentation of CD1d-glycolipid (top) and MHC-I-peptide (bottom) processing by DCs. With the use of a lysosome-specific tetrazine, the TCO-protecting group will only be removed when the antigens enter the lysosome before presentation at the cell surface. While the glycolipid antigens TCO-α-GalCer (**1**) and TCO-α-GalPhyto (**2**) pass the lysosome and will therefore activate the iNKT cells, the mbTCO-OVA18 (**6**) peptide does not enter the lysosome, resulting in no CD8^+^ T cell activation.

iNKT cells are hybrids between T cells and NK cells, expressing semi-invariant T cell receptors (TCRs) as well as NK cell markers (e.g., NK1.1).^33,34^ They show important dual functions (cell killing and cytokine secretion),^35^ particularly in the regulation of immune responses against cancer and certain infections.^36^ These cells are activated by DCs displaying glycolipid antigens on the CD1d receptors at their cell surface. The activation and loading biology of these ligands onto the CD1d receptors is complex. Lipid loading can take place in the endoplasmic reticulum (ER), where endogenous phosphatidylinositols^37^ and phosphocholines are preferentially loaded.^38^ The lysosomal population of CD1d on the other hand is mostly loaded by lipids extracted from the lysosomal membrane by Saposin B,^39,40^ such as lyso-phosphatidylserine and lysophosphocholine,^38^ which they can attain by taking on a pH-dependent lipid-receptive conformation.^41,42^ Exogenous glycolipids have not been found loaded on the endo-lysosomal pool of CD1d. This is surprising because the most potent exogenous iNKT-activator is α-galactosyl-ceramide (KRN7000, α-GalCer, **4**, Figure 1).^43,44^ This lipid, which is heavily pursued clinically,^36,45−52^ and its lower affinity variant α-galactosyl phytosphingosine^53^ (α-GalPhyto, **5**) are both taken up via endocytosis and are therefore *most likely* loaded onto CD1d^54,55^ in the endosome or lysosome (Figure 1). However, there is at present no method to determine the relative contribution of lysosomally loaded or endosomally loaded **4** and **5** to the activation of iNKT cells.^42,44,56,57^

The activation of CD8^+^ killer T cells is another area in which the lysosome plays a contentious role.^58,59^ In this process, polypeptide antigens are taken up by various endocytic mechanisms in the DC, yet end up on the major histocompatibility complex I (MHC-I) that is normally reserved for the presentation of endogenous peptides derived from cytosolic compartments. It has been a long-running debate to what extent cross-presented antigens pass through the lysosome.^60^ One proposed route suggests that a compartment with active cathepsin S is key in cross presentation,^61^ whereas other studies have suggested endosomal escape to the cytosol,^62^ or directly to the ER.^63^ Whether any of these escape events happen from an early endosome, or whether they occur from the lysosome remains unknown (Figure 1).

To study both these questions, tools for the lysosome-specific activation of these ligands would be essential. We here present such a system, in the form of a lysosome-specific bioorthogonal deprotection approach that allows us to study whether the CD1d ligands α-GalCer (**4**) and α-GalPhyto (**5**) as well as a CD8-activating long peptide antigen pass through lysosomes in the process leading to their loading on CD1d and MHC-I (Figure 1), respectively. We achieve this through the development and analysis of a lysosome-targeted tetrazine reagent (**3**, Figure 1) in combination with TCO-protected derivatives of α-GalCer (TCO α-GalCer, **1**, Figure 1), α-GalPhyto (TCO α-GalPhyto, **2**, Figure 1), and an 18-mer peptide containing the H-2Kb restricted model epitope SIINFEKL (mbTCO-OVA18, **6**, Figure 1). We show with this approach that the glycolipids do pass through the lysosome during the CD1d loading process, but the long peptide antigen does not during MHC-I loading.

## Results and Discussion

To perform a selective lysosomal deprotection, we required an uncaging reagent that would accumulate in the lysosome. For this we turned to the (2-aminoethyl)-morpholine group.^64^ This group is protonated at lysosomal pH-values and therefore accumulates in compartments with pH ≤ 4.5, which effectively leads to its retention in the lysosome. This group has been used to target a wide variety of cargo to the lysosome, including an NO-sensor,^64^ and the IR780 fluorophore.^65^ We opted to link this moiety to the recently reported 3-(pyridin-3-yl)-6-aminoethyl-1,2,4,5-tetrazine,^66^ using a glutaric anhydride ring opening followed by an amide condensation reaction to yield 3 (Figures 1 and S1).

**Figure 2:**
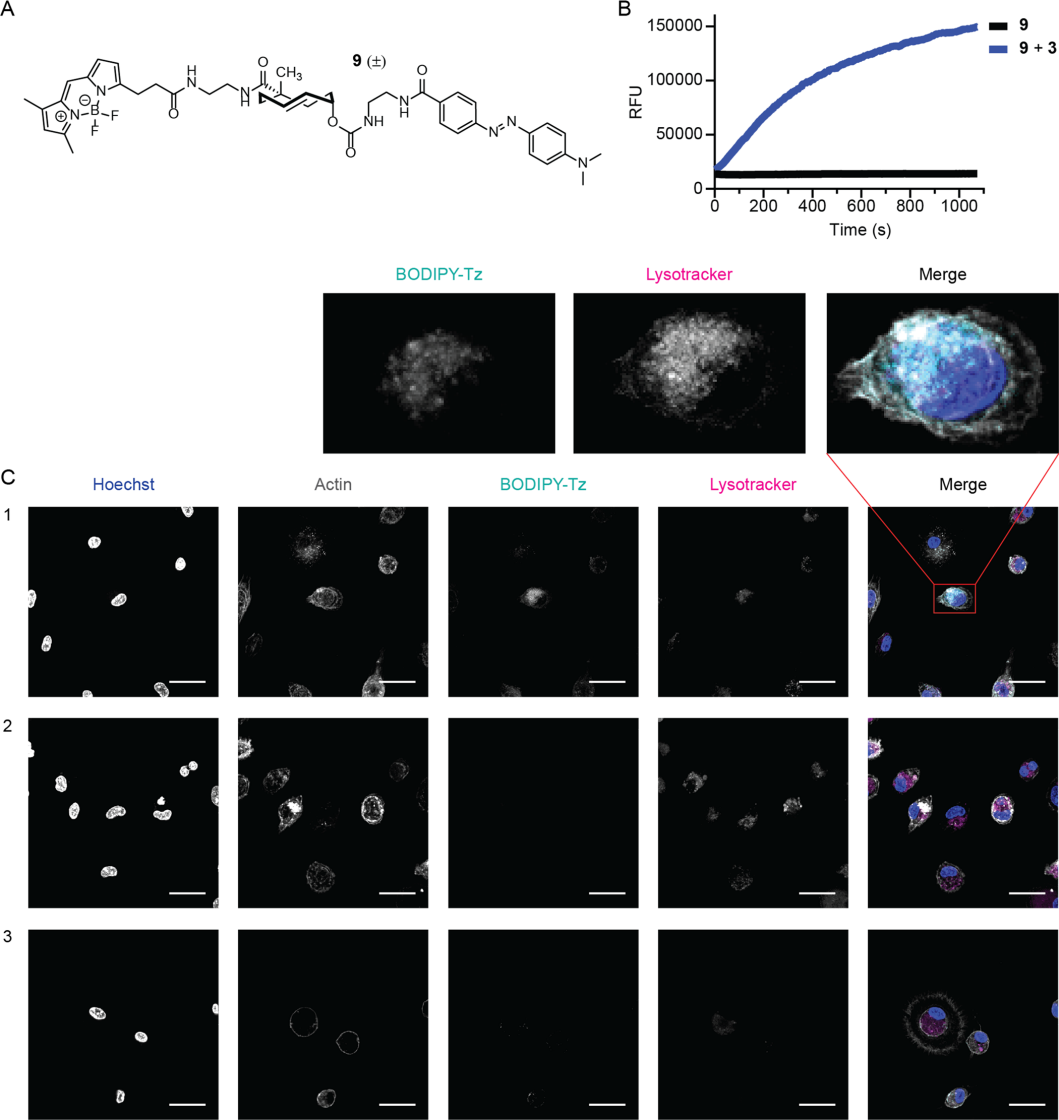
Evaluation of LysoTz (**3**). (A) Structure of DABCYL-TCO-BODIPY (**9**). (B) Turn-on rate of DABCYL-TCO-BODIPY (**9**, 1 μM) ± LysoTz **3** (10 μM) in H_2_O measured by increased fluorescent signal (spectral properties can be found in Figure S2). (C) Confocal microscopy images of bone marrow derived DCs (BMDCs) stained with Hoechst 33342 (DNA), CellMask Orange Actin tracking stain and LysoTracker Deep Red as reference. Legend: 1. LysoTz (**3**) + DABCYL-TCO-BODIPY (**9**); 2. Negative control for **3**; 3. Negative control for 9. All scale bars represent 20 μm.

We then characterized the lysosomal targeting properties of **3** by live cell microscopy, as the lysosome retention of the morpholine group is crucially dependent on the pH of the lysosome. We quantified the overlap with the known lysosome-specific fluorophore LysoTracker Deep Red, which is only fluorescent in the lysosome due to a combination of a targeting moiety and a H^+^-conditional fluorophore. Imaging **3** with a cell-permeable TCO-BODIPY fluorophore (**10**) resulted in poor signal-to-noise ratios (Figure S2). To reduce this background, we designed a cell-permeable quenched TCO-reagent (**9**) based on our previously reported fluorogenic bifunctional TCO probe for tetrazine characterization.^67^ As before, we utilized the bifunctional TCO^5^ to conjugate a 4-[4-(dimethylamino)phenylazo]benzoyl (DABCYL) quencher to a fluorophore which can undergo elimination, but in this case we used a cell-permeable BODIPY instead of 5-(2-aminoethylamino)naphtha-lene-1-sulfonic acid (EDANS) as the quenched fluorophore (Figures 2A and S1).^68^ Indeed, the DABCYL quenched the BODIPY fluorescence 10-fold (Figure 2B) without affecting the excitation/emission wavelengths of the dye (Figure S3). When we used DABCYL-TCO-BODIPY (**9**) to determine whether LysoTz **3** localized to the lysosome (Figure 2C) by confocal microscopy on BMDCs, we found that **3** labeled and colocalized with the commercially available LysoTracker Deep Red (Manders split coefficient M1 = 0.741 ± 0.089 and the Pearson’s correlation coefficient per cell gives *r* = 0.808 ± 0.052 or per image *r* = 0.460 ± 0.079). These results suggest good signal overlay between LysoTz **3** and LysoTracker Deep Red, thus confirming the accumulation of **3** in the lysosome. The lack of co-localization for other organelles, such as the nucleus, ER, and mitochondria (Pearson’s scores <0.05) further confirms this finding (Figure S4).

**Figure 3:**
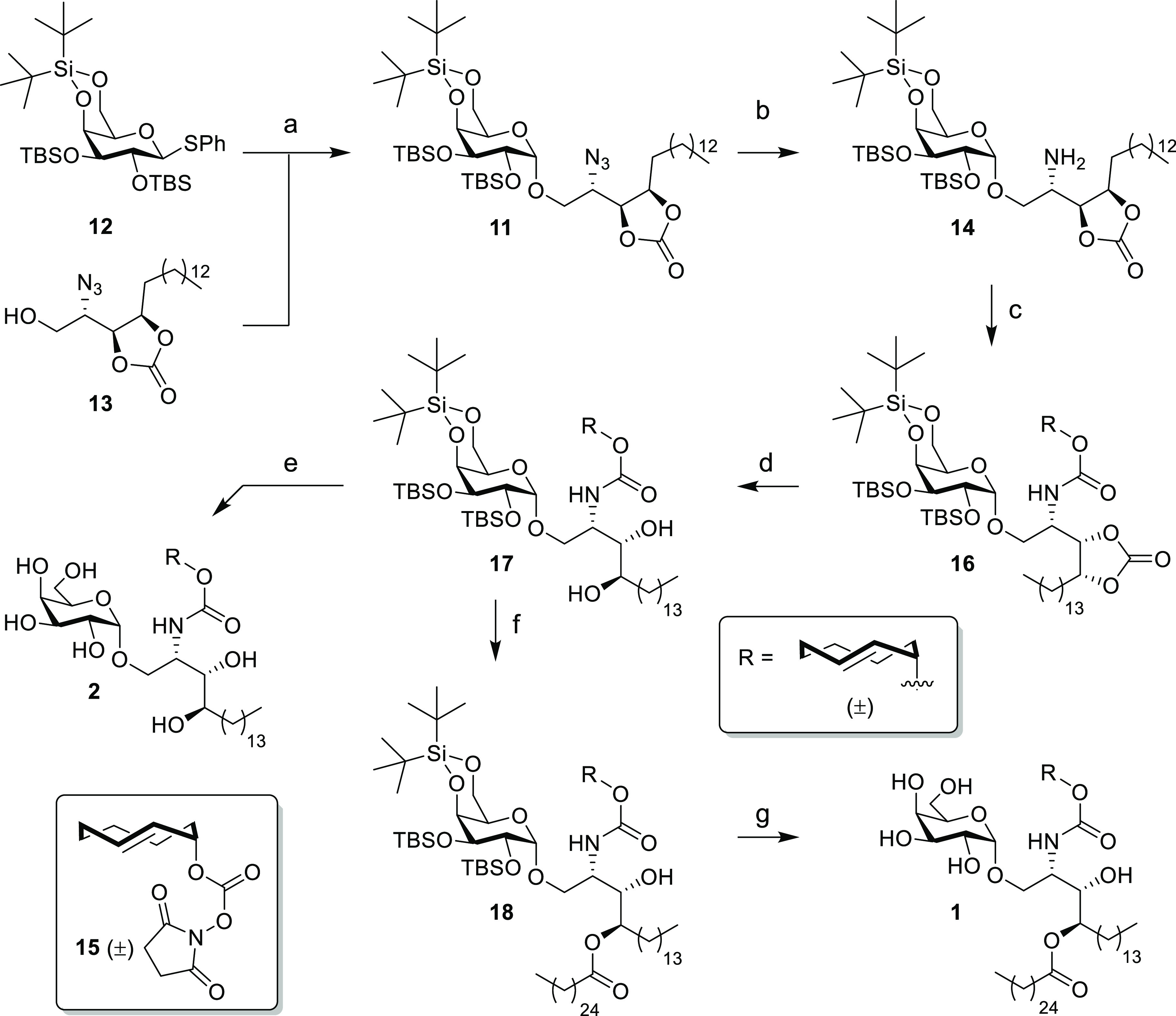
Synthesis of TCO-protected α-GalCer (**1**) and α-GalPhyto (**2**). Reagents/conditions: (a) NIS, TMS-OTf, DCM, −40 °C, 67%; (b) PtO_2_, H_2_ (g), THF, rt; (c) TCO-NHS, DIPEA, DMAP, DMF, rt, 89% over two steps; (d) LiOH, THF, H_2_O, rt, quant.; (e) Et_3_N·3HF, THF, rt, 84%; (f) hexacosonoic acid, EDC·HCl, DIPEA, DMAP, DCM, rt, 31–34%; (g) Et_3_N·3HF, THF, rt, 23%.

With lysosome-targeted tetrazine **3** in hand, we next turned to the synthesis of TCO-protected analogues of glycolipids **4** and **5.** Previous work by Painter and Hermans has shown that key contact residues for iNKT activation can be masked using a prodrug approach in which an enzymatically cleavable protecting group prevents CD1d/ TCR interaction and N→O acyl migration to form **4**.^49,53^ Furthermore, the Trauner lab modulated the activity of these ligands via azobenzene photoisomerization.^69^ Inspired by this, we chose to protect the amine functionality of the proform of **4** with an allylic TCO modality to yield a TCO-protected α-GalCer (**1**, Figures 1 and 3). Upon IEDDA pyridazine elimination, the resulting compound rearranges to form CD1d-ligand **4**. A similar approach was used for **5** to obtain TCO-protected α-GalPhyto **2** (Figure 1).

A key consideration for designing the synthetic route toward **1** and **2** (Figure 3) was the stability of the TCO moiety, which ruled out late-stage (global) deprotection by means of hydrogenolysis or acid, as is often the case for α-GalCer (**4**) syntheses reported in the literature.^70^ Formation of **11** was envisaged by combining 4,6-di-*tert*-butylsilylene (DTBS)-directed α-galactosylation^71−73^ with an azide protected phytosphingosine acceptor, as reported by Veerapen et al.^74^ More specifically, we selected 2,3-TBS-4,6-DTBS protected donor **12**(75) and 2-azido-3,4-cyclic carbonate acceptor **13**(76) as building blocks (full experimental details for **12** and **13** in Figure S5). This approach would enable selective saponification of the cyclic carbonate moiety after glycosylation, in addition to a mild desilylation as the final deprotection step.

For the glycosylation, employing a mixture of NIS and catalytic TMS-OTf at −40°C^74^ resulted in α-selective glycosylation using donor **12** and 1.5 equivalents of acceptor **13** to obtain **11** in 67% yield (Table S1). Hydrogenation of the α-galactosylated product (**11**) in the presence of Adam’s catalyst afforded amine **14**. Next, axial TCO carbonate **15**(10) was employed as a reagent to install the TCO carbamate moiety on **14**, in the presence of DIPEA and DMAP, to obtain **16** in 89% over two steps after chromatographic purification. Saponification of the cyclic carbonate functionality was performed with LiOH in a mixture of THF and H_2_O to obtain **17** as a crude product which could be directly used for subsequent steps. Steglich esterification^77^ of **17** and hexacosonoic acid in the presence of 1-ethyl-3-(3 -dimethylaminopropyl) carbodiimide hydrochloride (EDC·HCl), DMAP and DIPEA afforded **18** in 31–34% yield (Table S2).

Simultaneous deprotection of the cyclic DTBS protecting group and two TBS groups on the galactose moiety was evaluated for both **17** and **18** to obtain **2** and **1**, respectively (Table S3). Initial attempts investigated the deprotection of **17** with HF·pyridine and tetra-*n*-butylammoniumfluoride (TBAF), as individual reports on α-GalCer derivatives have shown both of these reagents to be effective for 4,6-DTBS deprotection.^74,78,79^ While these conditions resulted in a complex mixture of products or a lack of conversion, (prolonged) exposure to Et_3_N·3HF in THF afforded **2** in up to 84% yield. Et_3_N·3HF mediated deprotection conditions also enabled conversion of **18** to **1** in 23% yield without observing hydrolysis of the ester bond. NMR analysis for both **18** and **1** indicated the presence of a regioisomeric byproduct. As migration of the ester moiety was not observed during the deprotection of **18** to **1**, we suspect that the ester bond was installed without complete regioselectivity. Additionally, liquid chromatography–mass spectrometry (LC-MS) experiments with a nonreleasing tetrazine (Figure S6A–D) confirmed the *trans* configuration of the double bond for **2** and **1**. Taken together, while further optimization for the esterification and deprotection steps is warranted for **1** specifically, the results described confirm the compatibility of the deprotection conditions toward the envisioned synthetic strategy.

**Figure 4:**
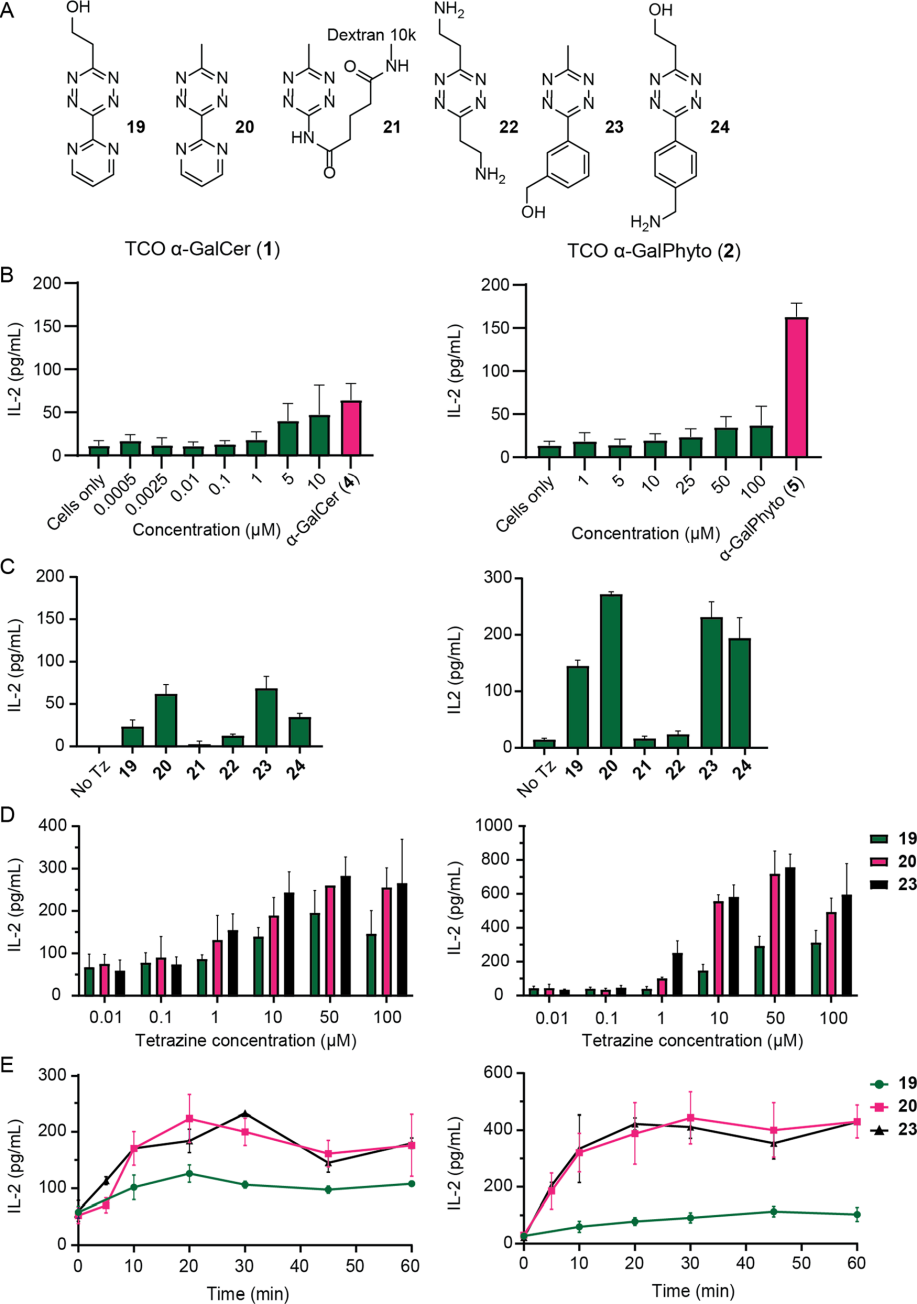
Characterization of iNKT cell activation with TCOα-galactosylceramide (α-GalCer, **1**) and TCOα-galactosyl phytosphingosine (α-GalPhyto, **2**) uncaged with tetrazines; the y-axis shows the IL-2 levels measured with ELISA as readout for DN32.D3 iNKT cell activation. (A) Structures of the six different tetrazines used for characterization. (B) iNKT cell activation measured by IL-2 levels upon addition of TCO α-GalCer (**1**, 0.3 μM) and TCO α-GalPhyto (**2**, 15 μM) at different concentrations. α-GalCer (**4**, 10 nM) and α-GalPhyto (**5**, 10 μM) were used as positive control. (C) iNKT cell activation measured upon uncaging of TCO with different tetrazines. (D) Concentration optimization of Tz-concentration for both 1 (left) and 2 (right)-based reagents; (E) IL-2 production with increasing Tz-incubation time showing near maximal uncaging after a 20-minutes Tz incubation. All experiments were performed in triplicate and with BMDCs from three different mice.

We next studied the biology of TCOα-GalCer (**1**) and TCOα-GalPhyto (**2**) in order to gain knowledge about the efficiency of the reaction in our cell-system. We first assessed whether TCO-caging could block iNKT activation (Figure 4). For this we used the DN32.D3 iNKT cell line the activation of which can be measured by IL-2 secretion. For **1**, no increase in IL-2 was observed at concentrations <5 μM, whereas for **2** this was <25 μM (Figure 4B).

This led us to choose a maximum concentration of 0.3 μM (for **1**) and 15 μM (for **2**) for all further experiments. Afterward, we assessed whether the addition of tetrazine to the ligand-loaded BMDCs led to the recovery of iNKT activation capacity. For this, we preincubated the BMDCs with the caged ligands followed by different tetrazines that we and others had previously shown to be capable of uncaging TCO on BMDCs (**19–24**).^5,7,10,66,80^ It was found that addition of tetrazines **19**, **20**, **23**, and **24** (Figure 4A) resulted in increased levels of IL-2 compared to the caged controls (Figure 4C). The improved recovery of the iNKT cell activation in living cells of **20** compared to **19** is in consensus with earlier work of Peng Chen et al.^7^ Tetrazine-functionalized dextran (**21**) displayed limited uncaging despite having been reported as an exclusively extracellular tetrazine.^5,81^ This is most likely the result of its hydrophilic and bulky character, which is in discord with the ligand located in the hydrophobic CD1d pocket.^82^ The toxicity of the tetrazines against BMDCs was also tested, and all were found to be nontoxic at 10 μM concentration (Figure S7).

Optimal tetrazine concentrations were investigated for three of these tetrazines (**19**, **20**, and **23**, Figure 4D). Although increased IL-2 levels were already observed at a concentration of 1 μM for **20** and **23**, **19** was less efficient at decaging **1** and **2**. We determined that 10 μM of **20** and **23** afforded an optimal uncaging yield (Figure 4D). Finally, the optimal in vitro uncaging time was assessed for both ligands and found to plateau after 20 min of incubation with the respective tetrazine (Figure 4E).

**Figure 5:**
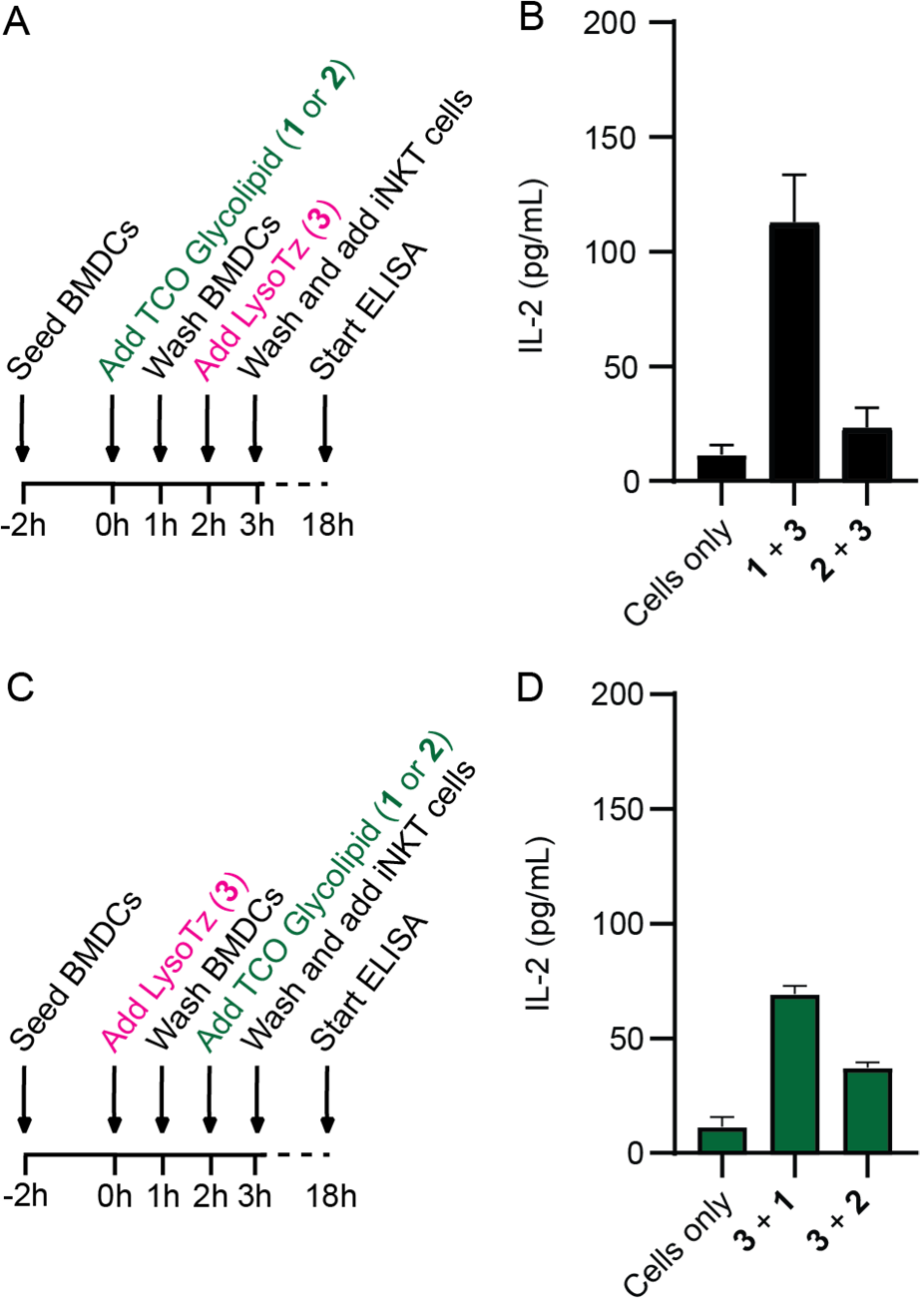
TCOα-galactosylceramide (α-GalCer) (**1**) and TCOα-galactosyl phytosphingosine (α-GalPhyto) (**2**) are both present in the lysosome before presentation by BMDCs; the y axis shows the IL-2 levels measured with ELISA as readout for DN32.D3 iNKT cell activation. (A) Experimental set-up for glycolipid uncaging with LysoTz (**3**) in BMDCs; (B) iNKT cell activation measured by IL-2 levels upon addition of TCOα-GalCer (**1**) and TCOα-GalPhyto (**2**) with thereafter addition of LysoTz (**3**); (C) experimental set-up for lysosomal-specific uncaging in BMDCs; (D) iNKT cell activation measured by IL-2 levels after preincubation with LysoTz (**3**) and after a wash addition of TCOα-GalCer (**1**) or TCOα-GalPhyto (**2**). All experiments were performed in triplicate and with BMDCs from three different mice.

Having confirmed the suitability of all reagents, we turned to studying lysosomal routing of ligands **1** and **2**. First, we tested whether LysoTz (**3**) was able to uncage the ligands extracellularly, after presentation by BMDCs (Figure 5A,B). Here, we observed activation of the iNKT cells for both compounds. Second, the lysosomal routing was tested by preincubating the DCs with LysoTz (**3**, 10 μM), before adding the caged ligands (**1**, 0.3 μM; **2**, 15 μM) and finally adding the iNKT cells, with washes in between all steps (Figure 5C,D). iNKT activation was observed for both ligands, confirming the successful release of the TCO protecting group in the lysosome. Interestingly, the uncaging of TCOα-GalPhyto (**2**) in the lysosome was more efficient compared to extracellular uncaging, possibly due to the shielding by CD1d when presented at the cell surface. Furthermore, applying our previously reported fluorogenic TCO-reporter-quencher assay^67^ on **3** at pH 7.2 and 4.5 revealed a high pH dependency of uncaging by **3**, both increasing the plateau from <10% elimination yield at pH 7.2 to ≥30% at pH 4.5 with a concomitant increase in the observed reaction rate (Figure S8). While it is generally accepted that a lower pH results in faster and more efficient decaging,^22,66,83^ this effect is perhaps more drastic for LysoTz **3** due to increased protonation of the morpholine moiety. Results for the other tetrazines investigated again emphasize the discrepancy between uncaging in a mere aqueous buffer and cellular systems^80^ (Figures 4 and S8). Altogether, we show that ligands **1** and **2** do pass the lysosomes before being presented by DCs.

We next studied whether peptides also pass through the lysosome during antigen cross-presentation, as the contribution of this compartment in this process is still contentious. We have previously shown that protection of a minimal 8-mer epitope of the model MHC-I restricted epitope SIINFEKL with the bifunctional mbTCO-group allowed control over its recognition by a cognate T cell.^10^ The peptides that were used in this work could be loaded on the surface of the cell, therefore not requiring any intracellular antigen processing and routing before presentation. In order to study whether peptide antigens pass through the lysosome during antigen cross-presentation, we made a variant peptide containing this same epitope that cannot be loaded onto MHC-I on the surface. This is achieved by extending the peptide at the *N*-terminus so that it requires proteolysis before the epitope fits in the MHC-I.^84^ We synthesized a TCO-protected 18-mer peptide **6**, containing the MHC-I restricted epitope with TCO protection on the ε-amino group of Lys-7, using the previously established N-terminal methylsulfonylethyloxycarbonyl (MSc)^85^ protection strategy to selectively install the TCO moiety (Figure S9).^10^

**Figure 6:**
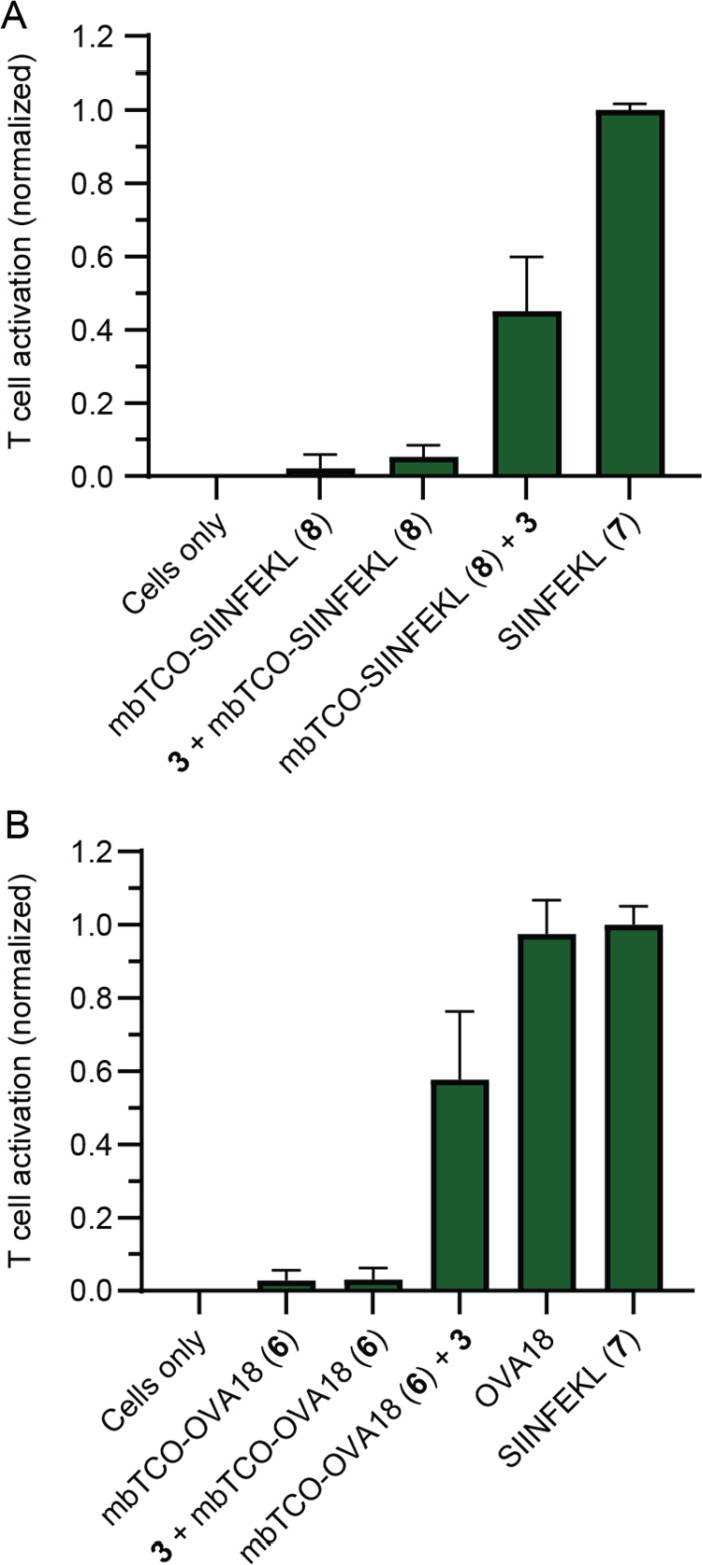
SIINFEKL peptides do not enter the lysosome before presentation by DCs. B3Z T cell activation assay was used to assess the amount of “uncaged” antigen presented by D1 cells;^86^ the y axis shows the normalized absorbance at 570 nm. (A) T cell activation upon addition of 100 nM mbTCO-SIINFEKL (**8**) ± LysoTz (**3**). As control, LysoTz (**3**) is also added after peptide presentation and the samples are normalized between the negative cells only and the positive 100 nM SIINFEKL control. (B) T cell activation upon addition of 50 μM mbTCO-OVA18 (**6**) ± LysoTz (**3**). LysoTz (**3**) is also added as control after peptide presentation and the samples are normalized between the negative cells only and the positive 10 nM SIINFEKL (**7**) controls. All experiments were performed three times and in triplicate.

We first determined whether **3** was capable of deprotecting the caged epitope. The cells were first incubated with the mbTCO-modified minimal SIINFEKL epitope (**8**, 100 nM) which could be loaded directly in MHC-I on the surface of the DC. After this 1 h loading period, the cells were washed and **3** was added for 1 h at 10 μM. A SIINFEKL-specific T cell hybridoma B3Z^86^ and its activation (measured by quantifying the NFAT-induced expression of the reporter protein β-galactosidase) served as a proxy for surface MHC-I-restricted SIINFEKL concentration. This assay showed that **3** could uncage approximately 60% of the mbTCO group, which was in a similar range to previously reported tetrazines (Figure 6A).^10^ Preloading the DCs with **3** followed by incubation with mbTCO-SIINFEKL did not result in uncaging, suggesting no exocytosis of the reagent into the medium over the timescales of the experiment (Figure 6A).

We then determined whether LysoTz (**3**) could also uncage the long peptide **6** during antigen processing. We assessed whether preloading the lysosome with **3** (10 μM) followed by the addition of **6** (20 μM) yielded any deprotected MHC-I-loaded SIINFEKL (Figure 6B). In the experiments analogous to those performed for CD1d ligands **1** and **2**, no T cell activation was found, suggesting no role for the lysosomal compartments during cross-presentation of this particular peptide.

## Conclusions

With the use of the bioorthogonal bond-breaking IEDDA reaction,^3,4^ we created a method for organelle-specific targeting. Here, we synthesized a tetrazine which only accumulates in lysosomes, resulting in lysosome-specific cleavage of the blocking TCO group. With this method, we studied the routing of caged iNKT glycolipids TCO α-GalCer (**1**) and TCO α-GalPhyto (**2**) through the late endosomal pathway. We showed that the TCO moiety rendered the CD1d ligands inactive, as iNKT cells incubated with the caged lipids would not activate in the absence of a tetrazine trigger. Only after preincubation with LysoTz (**3**), followed by the administration of the caged lipids **1** or **2**, could the iNKT cells be activated. This in combination with the already confirmed presence of CD1d^41,42^ in the lysosome suggests that loading of these glycolipids occurs in the late endosome. This lysosome-specific deprotection strategy cannot only be used for the processing pathway of glycolipids but can be well translated to different antigens, such as peptide antigens for CD8^+^ and CD4^+^ T cell activation. In this study, we show that mbTCO-OVA18 (**6**) is not present in the lysosome during cross-presentation, indicating that the processing of this synthetic long peptide (SLP) is confined to early endosomal compartments or cytosolic proteases.

## Supporting Information Available

Experimental procedures and methods, characterization data, and excitation and emission spectra, confocal microscopy images, and LC-MS analysis results (PDF)
