# Supplementary material for: A Lysosome-Targeted Tetrazine for Organelle-Specific Click-to-Release Chemistry in Antigen Presenting Cells
Source: J Am Chem Soc. 2023 Jun 3;145(23):12630–40. doi: 10.1021/jacs.3c02139 (PMC10273227; doi:10.1021/jacs.3c02139)
Supplement: Supplementary file 1 — ja3c02139_si_001.pdf [file ja3c02139_si_001.pdf]

Supporting Information (SI):

A Lysosome-Targeted Tetrazine for Organelle-Specific Click-To-Release Chemistry In Antigen Presenting Cells.

Nina A.M. Ligthart[1,#], Mark A.R. de Geus[1,†,#], Merel A.T. van de Plassche[1], Diana Torres García[1], Marjolein M.E. Isendoorn[1], Luuk Reinalda[1], Daniëlle Ofman[1], Tyrza van Leeuwen[1], and Sander I. van Kasteren[1,*]

[1]Leiden Institute of Chemistry and The Institute for Chemical Immunology, Leiden University, Einsteinweg 55, 2333 CC Leiden, The Netherlands

Table of Contents

1. Supplementary Figures 4
Figure S1. 4
Figure S2. 5
Figure S3. 5
Figure S4. 6
Figure S5. 7
Figure S6-A. 8
Figure S6-B. 9
Figure S6-C. 10
Figure S6-D. 11
Figure S7. 12
Figure S8 13
Figure S9. 14
2. Supplementary Tables 15
Table S1 15
Table S2 16
Table S3 17
3. Experimental Procedures – Immunology 18
3.1 Cell culture 18
3.2 iNKT cell activation by IL-2 ELISA 19
3.3 B3Z T-cell activation assay 19
3.3 Confocal microscopy 20
3.4 Characterization DABCYL-TCO-BODIPY (9) 20
3.5 Fluorogenic TCO uncaging assay 20
4. Experimental Procedures – Organic Synthesis 21
5. NMR spectra 35
^{1}H and ^{13}C APT spectra of 26 36
^{1}H, ^{13}C APT, ^{1}H COSY and HSQC spectra of 3 37
^{1}H spectrum of 50 38
^{1}H and ^{13}C spectrum of 24 40
^{1}H spectrum of 28 41
^{1}H, ^{13}C APT, ^{1}H COSY and HSQC spectra of 31 42
^{1}H, ^{13}C APT, ^{1}H COSY and HSQC spectra of 36 43
^{1}H, ^{13}C APT, ^{1}H COSY and HSQC spectra of 37 46
^{1}H, ^{13}C APT, ^{1}H COSY and HSQC spectra of 38 48

^{1}H, ^{13}C APT, ^{1}H COSY and HSQC spectra of 12 50
^{1}H, ^{13}C APT, ^{1}H COSY and HSQC spectra of 41 51
^{1}H, ^{13}C APT, ^{1}H COSY and HSQC spectra of 42 54
^{1}H, ^{13}C APT, ^{1}H COSY and HSQC spectra of 43 56
^{1}H, ^{13}C APT, ^{1}H COSY and HSQC spectra of 13 58
^{1}H, ^{13}C APT, ^{1}H COSY and HSQC spectra of 11 60
^{1}H, ^{13}C APT, ^{1}H COSY and HSQC spectra of 14 62
^{1}H, ^{13}C APT, ^{1}H COSY and HSQC spectra of 16 64
^{1}H, ^{13}C APT, ^{1}H COSY and HSQC spectra of 17 66
^{1}H, ^{13}C APT, ^{1}H COSY and HSQC spectra of 2 (Pyridine-d_5) 68
^{1}H, ^{13}C APT, ^{1}H COSY and HSQC spectra of 2 (Dioxane-d_8) 70
^{1}H, ^{13}C APT, ^{1}H COSY and HSQC spectra of 18 72
^{1}H, ^{13}C APT, ^{1}H COSY and HSQC spectra of 1 75
6. LC-MS spectra 78
LC-MS spectrum of BODIPY-TCO (10) 79
LC-MS spectrum of BODIPY-TCO-DABCYL (9) 80
LC/MS analysis of 44 81
LC/MS analysis of 46 82
LC/MS analysis of 47 83
LC/MS analysis of 6 84
7. Supporting References 85

1. Supplementary Figures

Figure S1.

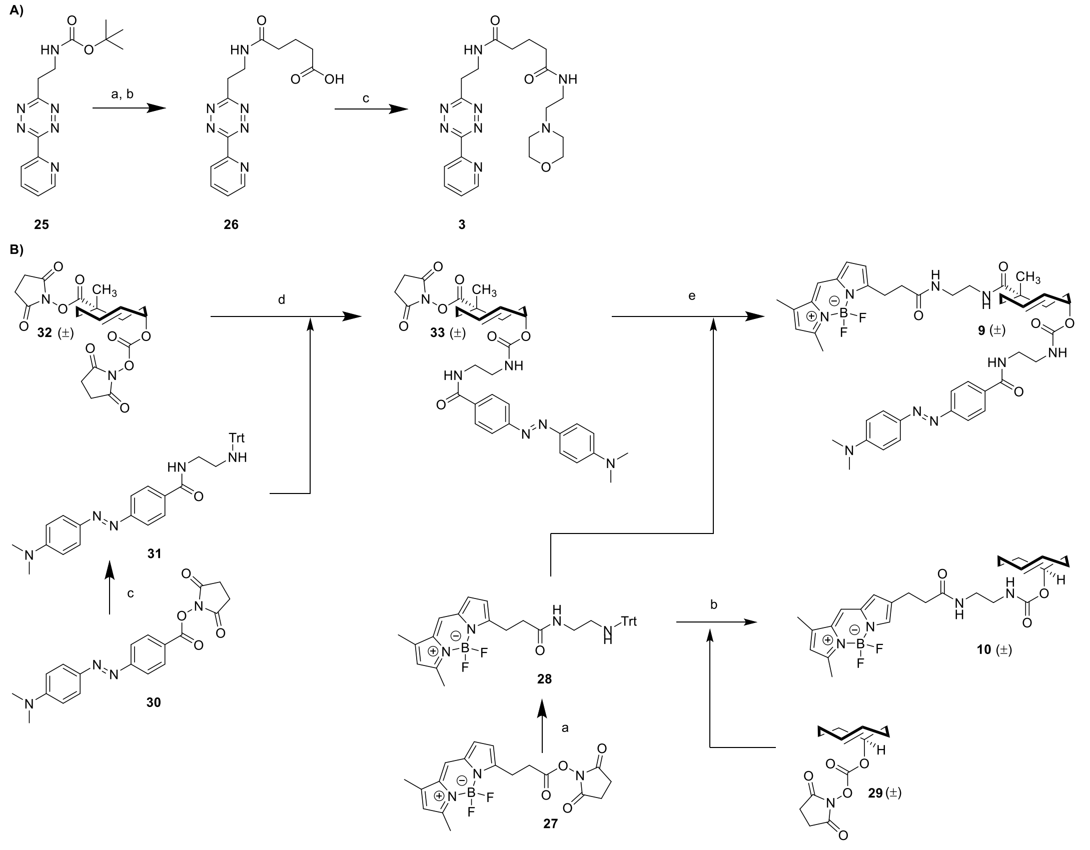

Supporting Figure S1 | Synthesis of LysoTz (3), DABCYL-TCO-BODIPY (9) and TCO-BODIPY (10). A) Reagents/conditions: a) 4M HCL in dioxane, rt; b) glutaric anhydride, Et_3N, MeCN, rt, 73% over two steps; c) PyBOP, DIPEA, 4-(2-aminoethylmorpholine), DMF, rt, 25%. B) Reagents/conditions: a) N-tritylethylenediamine, DIPEA, DMF, rt, 75%; b) i) 1% TFA in DCM, rt; ii) 4-TCO-NHS **29**, DIPEA, DCM, rt, 86%; c) N-tritylethylamine, DIPEA, DCM, 100%; d) i) 1% TFA in DCM, rt; ii) Bis-NHS-TCO **32**, DIPEA, DCM, rt, 77%; e) 1% TFA in DCM, rt; ii) **33**, DIPEA, DMF, rt, 52%.

Figure S2.

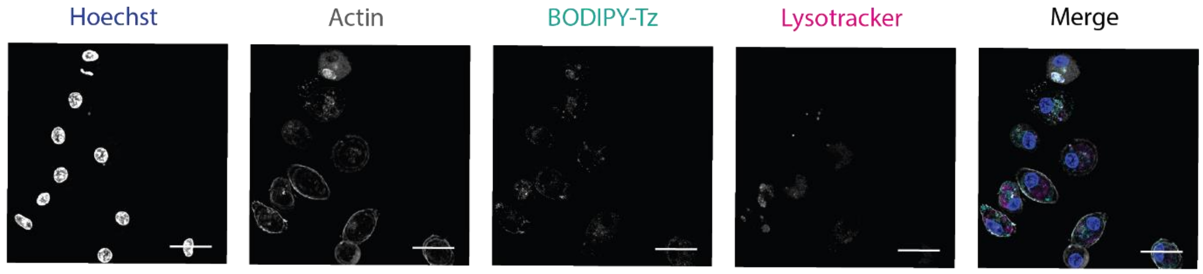

Supporting Figure S2 | Microscopy with TCO-BODIPY (10). Confocal microscopy images of bone marrow derived dendritic cells (BMDCs) stained with TCO-Bodipy **10**, Hoechst 33342 (DNA), CellMask™ Orange Actin tracking stain and LysoTracker™ Deep Red as reference.

Figure S3.

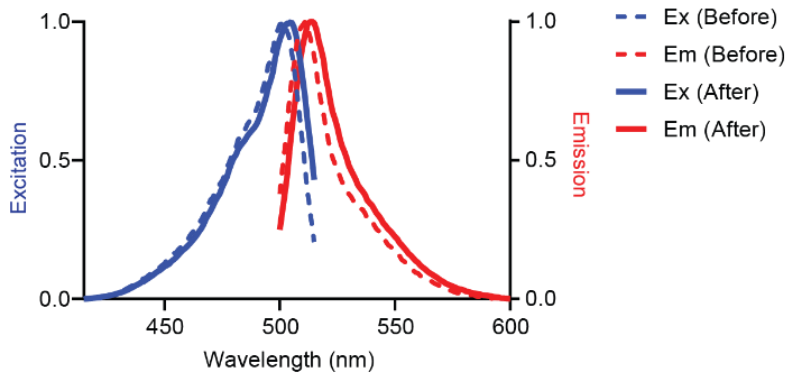

Supporting Figure S3 | Excitation and emission of DABCYL-TCO-BODIPY (9). Excitation and emission spectra of DABCYL-TCO-BODIPY (**9**) before and after addition of LysoTz (**3**). Excitation/emission at 477-14/530-40 nm and dichroic filter 497. Values were normalized to their respective maxima.

Figure S4.

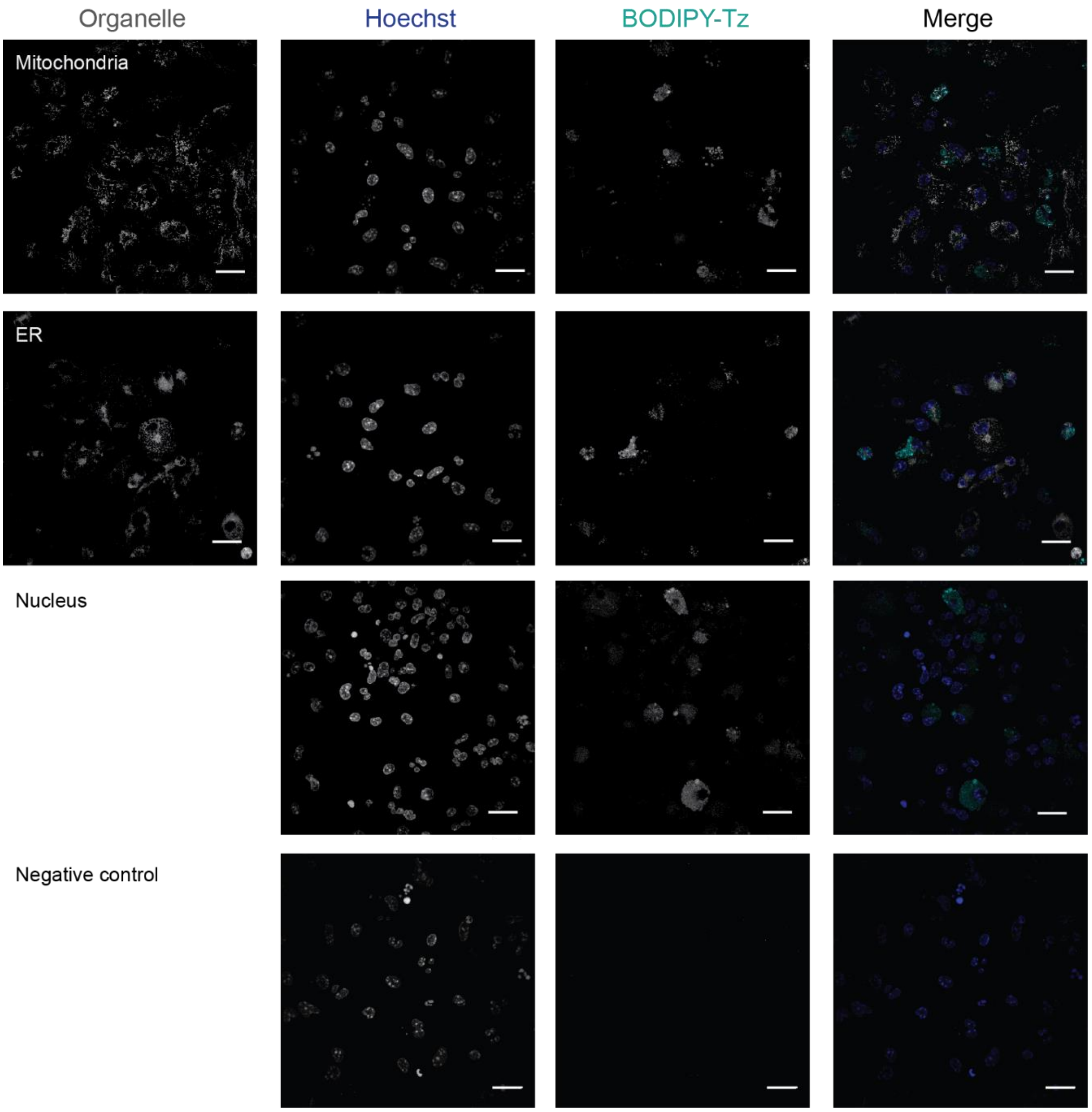

Supporting Figure S4 | Co-localization of LysoTz 3 with different organelles. Confocal microscopy images of bone marrow derived dendritic cells (BMDCs) stained with TCO-BODIPY-DABSYL **9** bound to LysoTz **3**, MitoSpy™ Orange or ER-Tracker™ Red and Hoechst 33342 (DNA) as reference. The negative control is without LysoTz **3**. All scale bars represent 20 μm. Mitochondria; Manders split coefficient M1 = 0.205 ± 0.006 and the Pearson's correlation coefficient r = 0.042 ± 0.019. Endoplasmic reticulum (ER); M1 = 0.338 ± 0.050 and the Pearson's correlation coefficient r = 0.042 ± 0.019. Nucleus; M1 = 0.132 ± 0.020 and the Pearson's correlation coefficient r = 0.018 ± 0.014.

Figure S5.

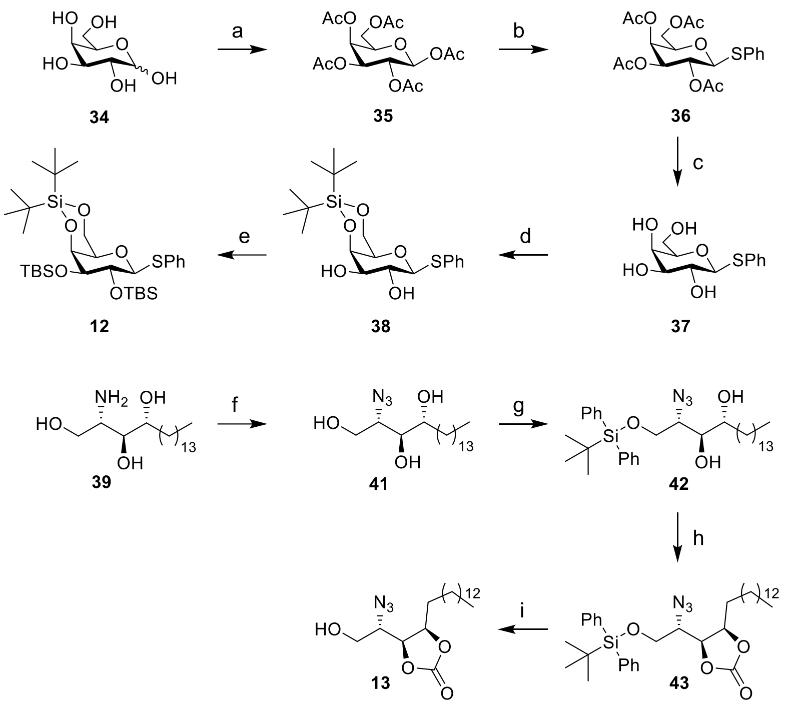

Supporting Figure S5 │ Synthesis of galactose donor 12 and phytosphingosine acceptor 13. Reagents/conditions: (a) Ac_2O, NaOAc, reflux, 52%; (b) PhSH, $BF_3 \cdot OEt_2$, DCM, 0°C to rt, 95%; (c) NaOMe, MeOH, rt, 95%; (d) DTBS-OTf, pyridine, DMF, -40°C, 83%; (e) TBS-OTf, DMAP, pyridine, 0°C to rt, 95%; (f) imidazole-1-sulfonyl azide hydrogen sulfate (**40**), K_2CO_3, Cu(II) · 5 H_2O, MeOH, DCM, rt; (g) TBDPS-Cl, Et_3N, DMAP, DCM, rt, 83% over two steps; (h) CDI, DCM, rt, 79%; (i) HF · pyridine, pyridine, rt, 92%.

Figure S6-A.

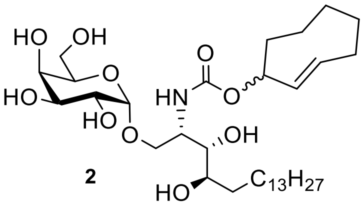

2

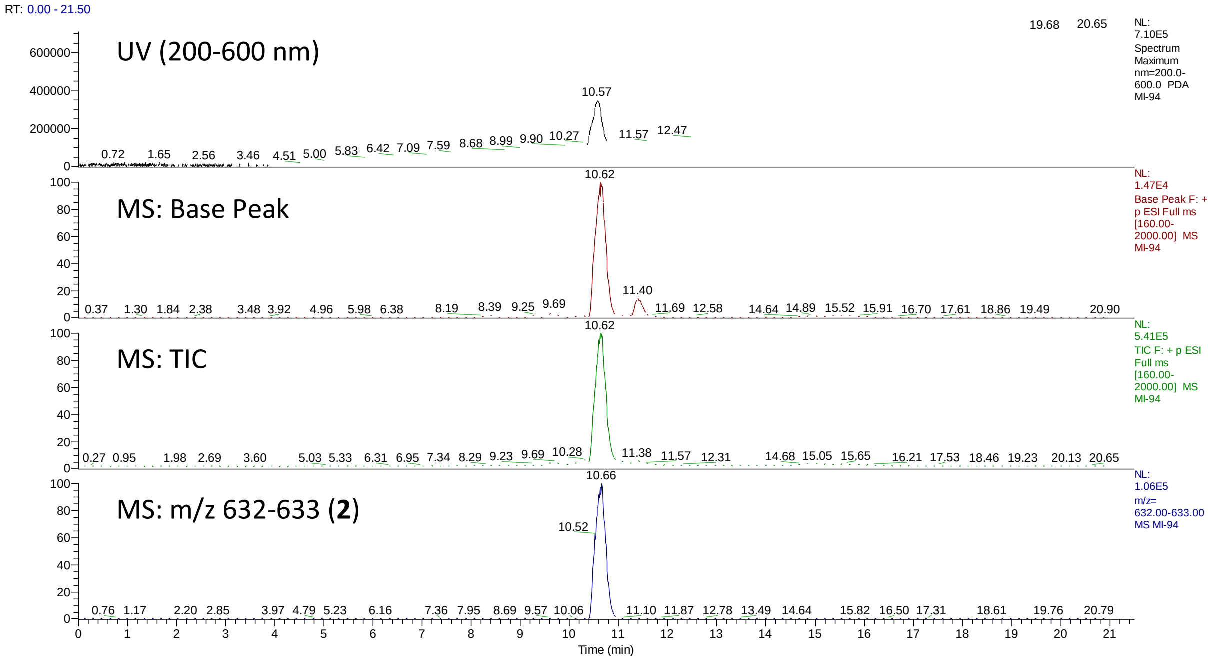

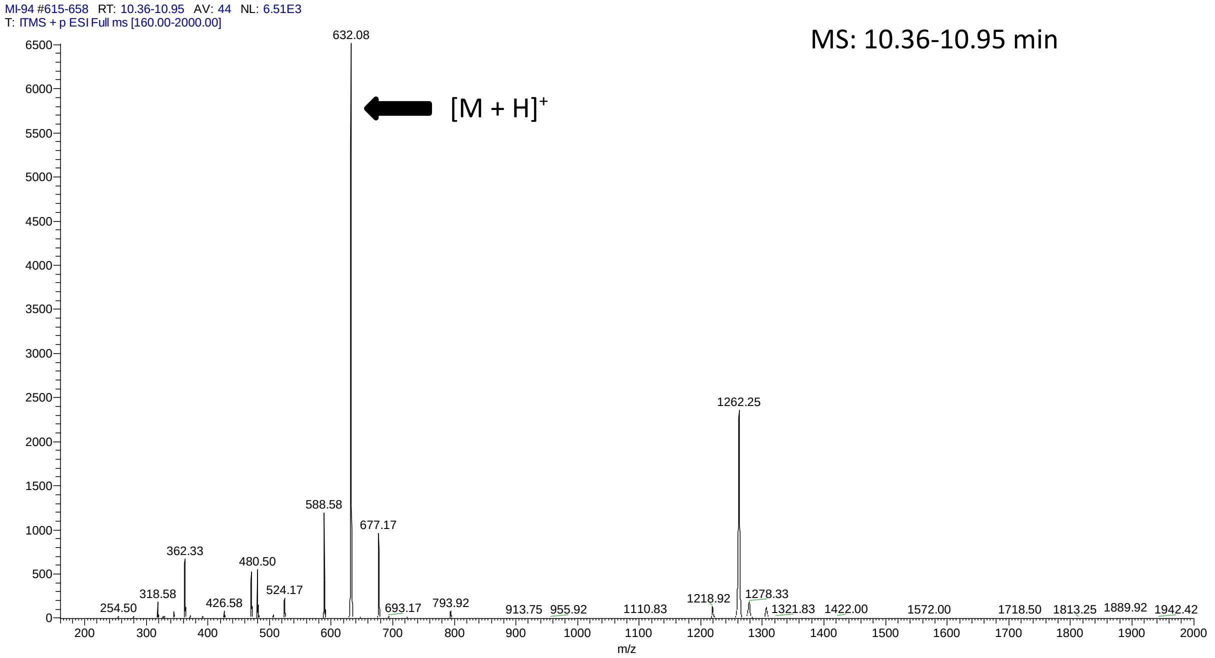

Supporting Figure S6A | Reference LC-MS spectrum of 2.
Diphenyl column, 10→90% MeCN, 1% TFA, 15 min.

Figure S6-B.

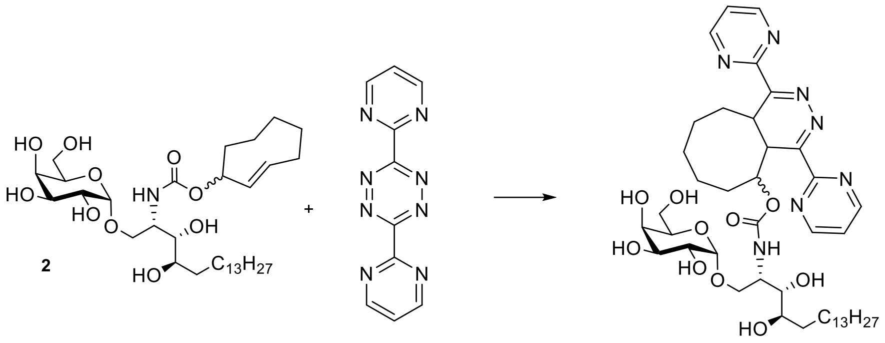

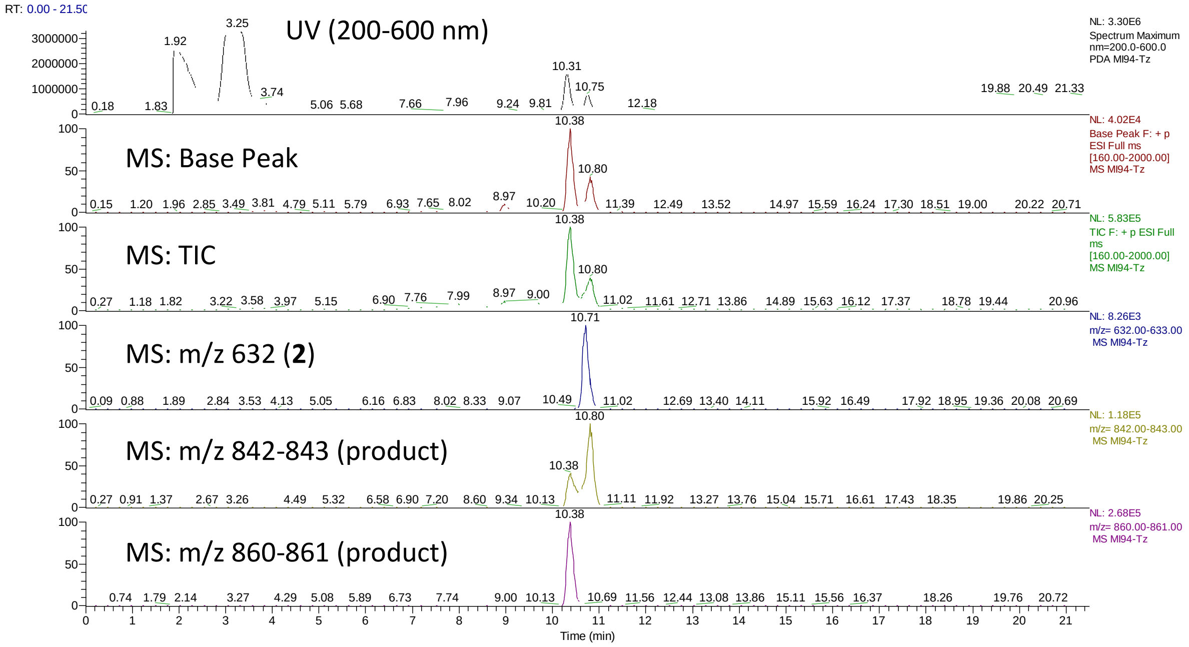

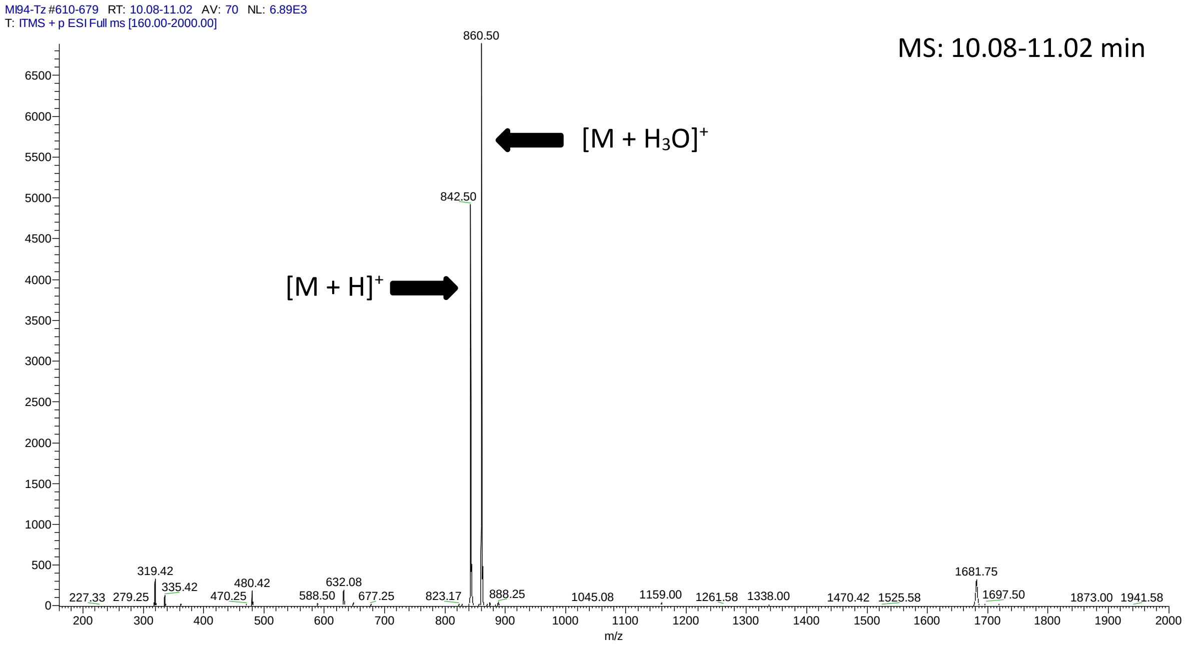

Supporting Figure S6-B | LC-MS analysis of click reaction mixture (2 + BPymTz[1]).
Diphenyl column, 10→90% MeCN, 1% TFA, 15 min.

Figure S6-C.

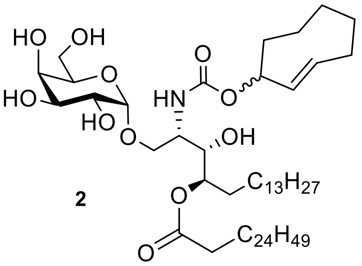

2

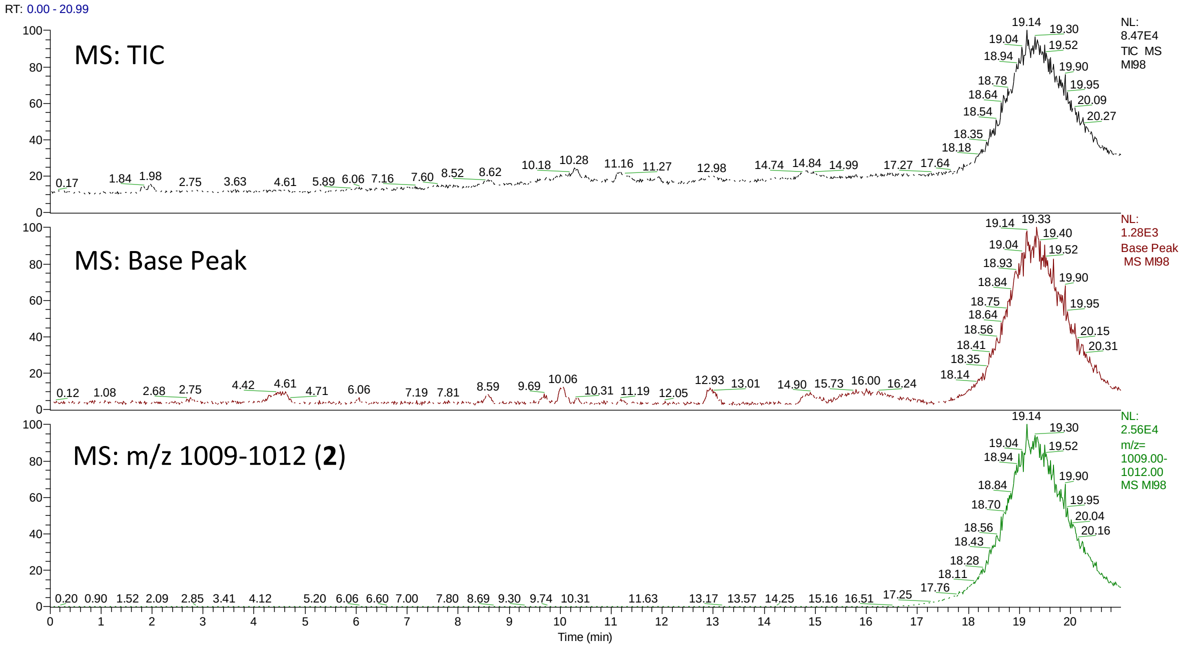

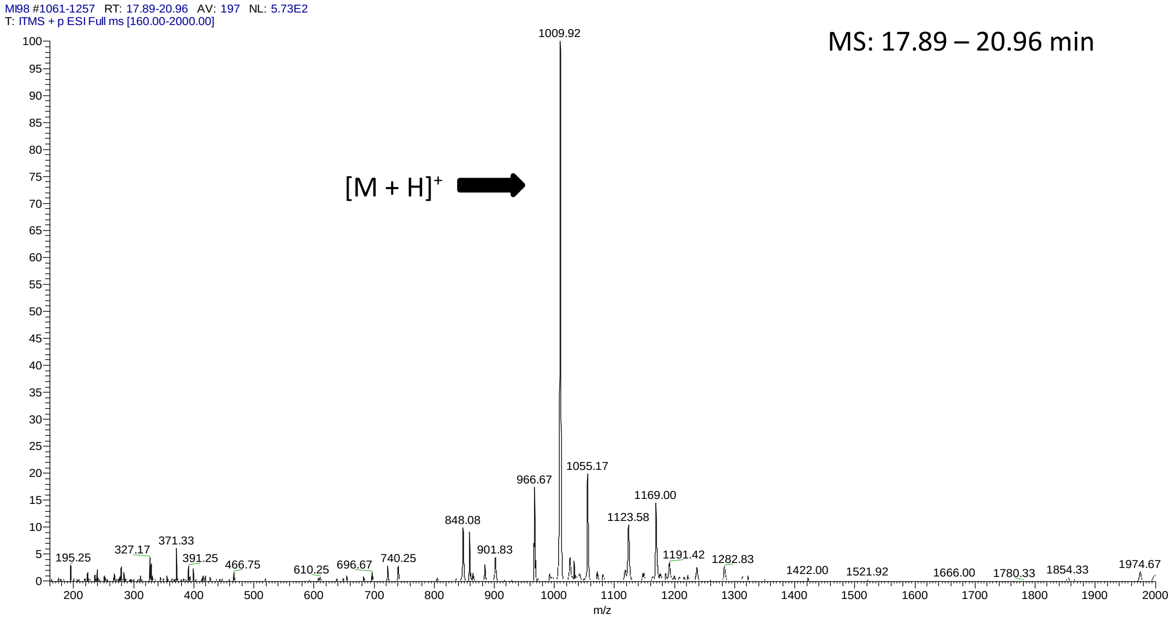

Supporting Figure S6-C | Reference LC-MS spectrum of 1.
Diphenyl column, 10→90% MeCN, 1% TFA, 15 min.

Figure S6-D.

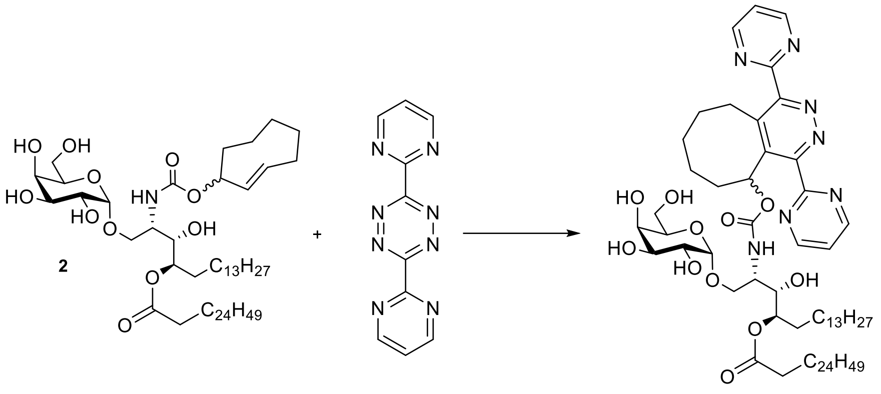

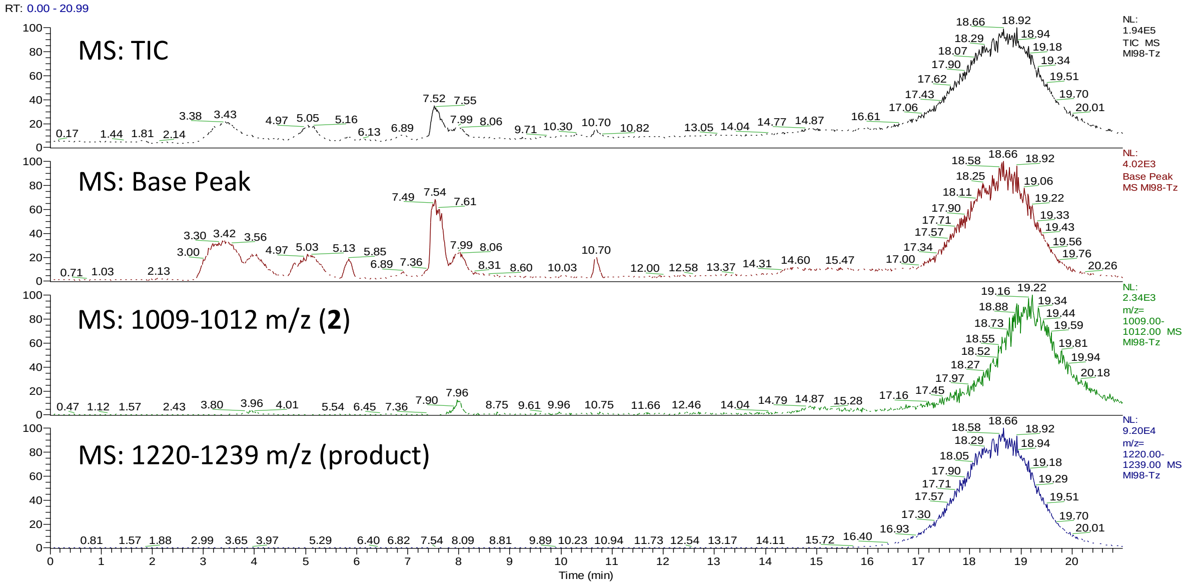

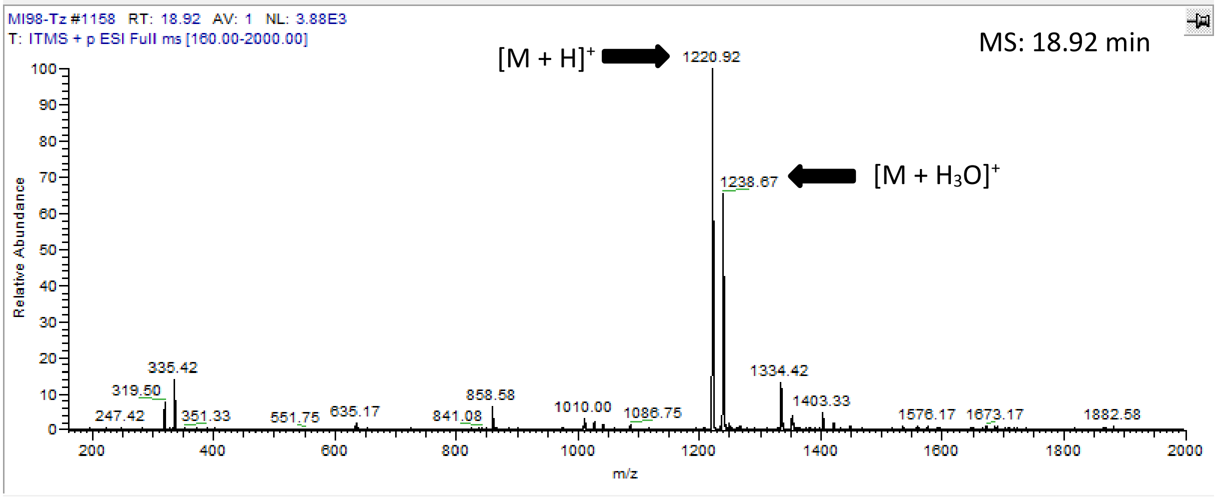

Supporting Figure S6-D | LC-MS analysis of click reaction mixture (1 + BPymTz[1]).
Diphenyl column, 10→90% MeCN, 1% TFA, 15 min.

Figure S7.

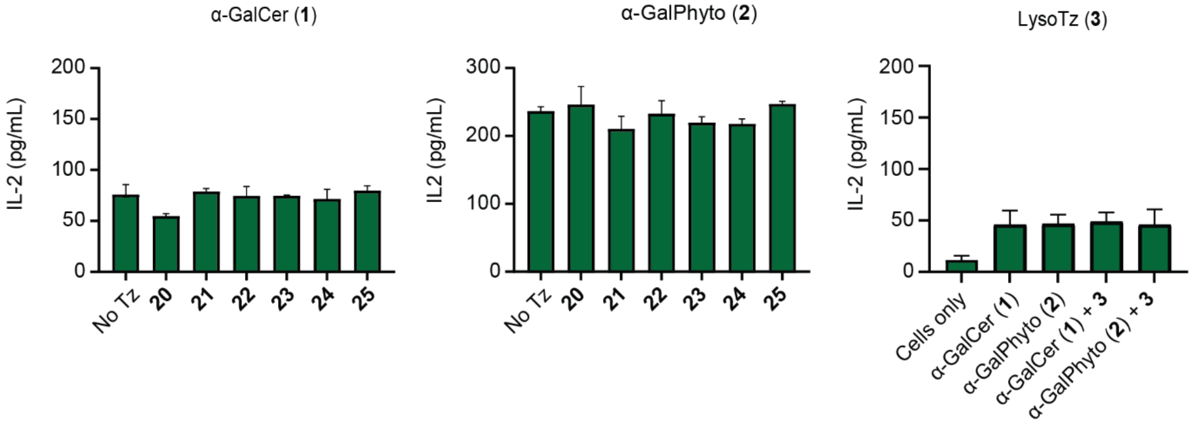

Supporting Figure S7 | Tetrazine toxicity in iNKT activation assay. Toxicity measurement where iNKT cells activation is measured after addition of non-caged α-GalCer (**1**,10 nM) and α-GalPhyto (**2**, 10 µM) ± tetrazine (10 µM). All experiments were performed in triplicate and with BMDCs from 3 different mice. The y axis shows the IL-2 levels measured with ELISA as readout for DN32.D3 iNKT cell activation.

Figure S8

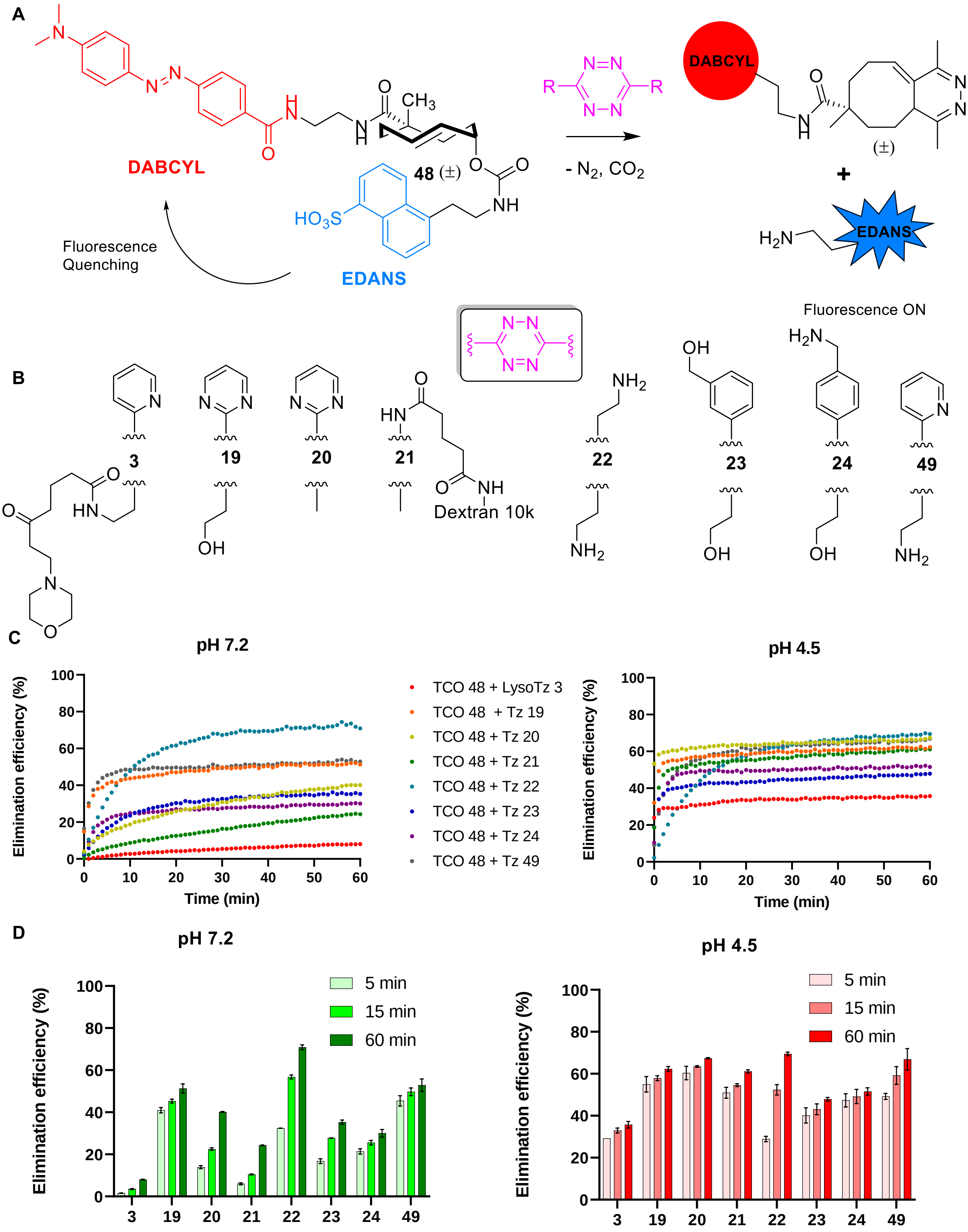

Supporting Figure S7 | Fluorogenic TCO uncaging assay[2] with a panel of tetrazines at pH 7.2 and 4.5 (N = 1, n = 2). A) Underlying principle of the fluorogenic assay. B) Panel of tetrazines investigated. C) Elimination efficiency (%) observed for TCO **48**[2] (10 μM) in the presence of tetrazine derivatives (100 μM) at pH 7.2 (PBS) and 4.5 (0.1 M sodium citrate buffer). Raw fluorescence data (λex = 340-10 nm, λem = 495-10 nm) was normalized using EDANS (10 μM) and TCO **48** (10 μM) as 100% and 0% reference values, respectively. D) Bar graph representation of the data shown in C) showing data after 5 min, 15 min and 60 min.

Figure S9.

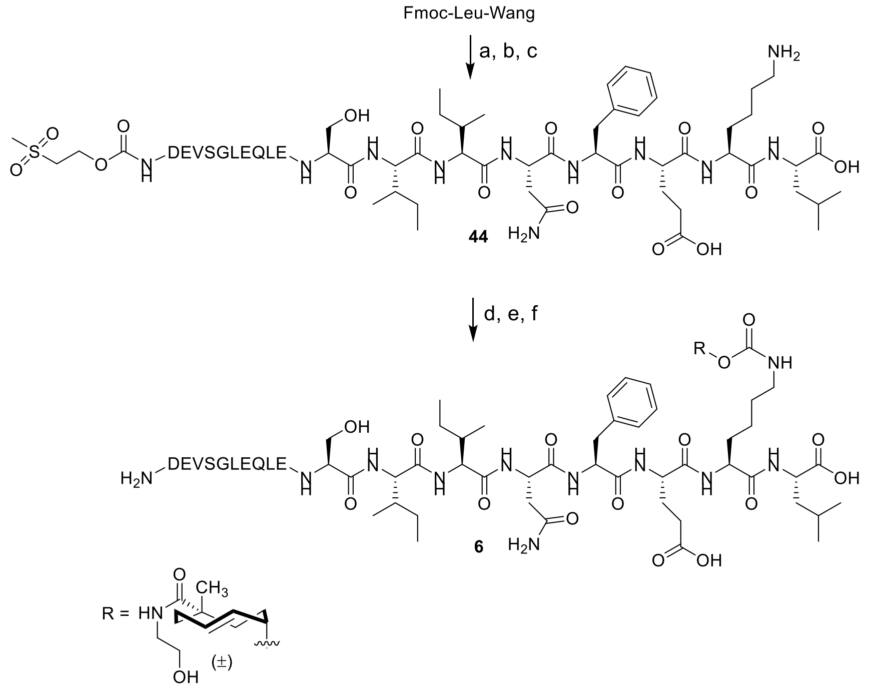

Supporting Figure S8 │ Synthesis of mbTCO-OVA18 (6). Reagents/conditions: (a) Fmoc SPPS from Fmoc-Leu-Wang (A); (b) methylsulfonylethyl succinimido-carbonate (**45**), DIPEA, NMP, rt; (c) TFA / H_2O / TIPS (95:2.5:2.5), rt, 14%; (d) NHS-mbTCO (**32**), DIPEA, DMSO, rt; (e) ethanolamine, DMSO, rt; (f) dioxane/MeOH/4 M NaOH (7.5:2.25:0.25), rt, 41% over three steps.

2. Supplementary Tables

Table S1

Table 1 Glycosylation of galactose donor **12** and phytosphingosine acceptor **13** to form **11**.

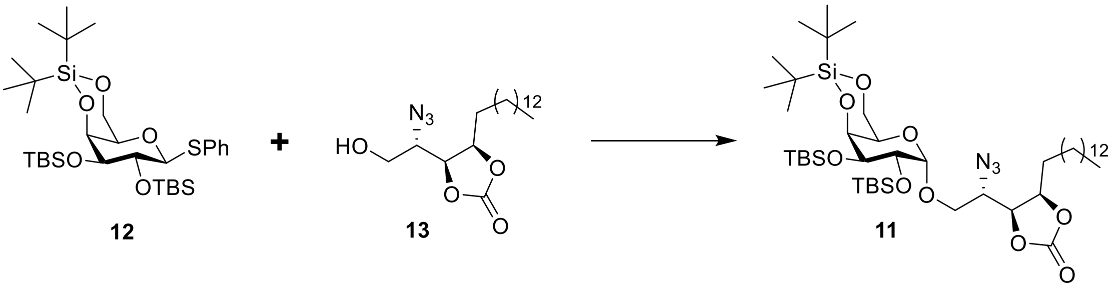

Entry	Scale (mmol)	Donor 12 (equiv)	Promotor system (equiv)	Solvent (M)	Temp. (°C)	Time (min)	Yield (%)[a]
1	0.1	1.5	IDCP (3.0)	DCM (0.2)	0 → rt	-	-
2	0.15	1.5	NIS (1.5), AgOTf (0.3)	DCM (0.2)	0 → rt	-	-
3	0.1	1.5	NIS (1.5), TfOH (0.2)	DCM (0.1)	-40	15	63
4	0.4	1.5	NIS (1.5), TfOH (0.2)	DCM (0.1)	-40	60	52
5	0.6	1.5	NIS (1.5), TfOH (0.1)	DCM (0.1)	-40	60	34
6	0.2	1.5	NIS (1.5), TMS-OTf (0.2)	DCM (0.1)	-40	15	85
7	2.4	1.2	NIS (1.5), TMS-OTf (0.2)	DCM (0.1)	-40	180	59
8	5.3	1.5	NIS (1.5), TMS-OTf (0.2)	DCM (0.1)	-40	300	67

[a]Isolated yield after aqueous workup and chromatographic purification.

Table S2

Table 2 Esterification of **17** to obtain **18**

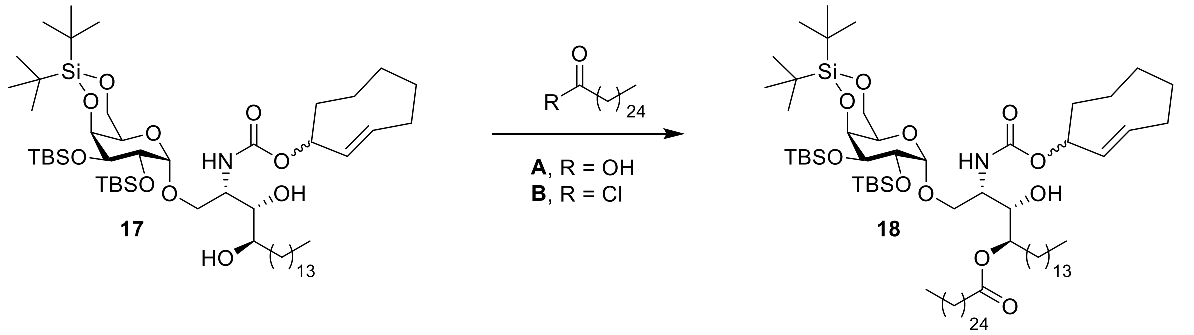

Entry	Scale (mmol)	Reagent (equiv)	Coupling conditions (equiv)	Solvent (M)	Temp. (°C)	Time (min/h/d)	Yield (%)[a]
1	0.08	**A** (1.5)	PyBOB (1.5)<br>DIPEA (3.0)	DCM (0.04)	rt	5 d	-
2	0.09	**A** (1.3)	EEDQ (2.0)	EtOH (0.06)	0 → rt → 50	2 d	-
3	0.10	**B** (1.3)	DIPEA (4.0)	DCM (0.02)	-20 → rt	30 min	-
4	0.10	**A** (1.5)	EDC · HCl (1.5), DMAP (6.0)<br>DIPEA (3.0)	DCM (0.03)	0 → rt	20 h	31
5	0.2	**A** (1.5)	EDC · HCl (1.5), DMAP (6.0)<br>DIPEA (3.0)	DCM (0.05)	0 → rt	3 d	32
6	0.08	**A** (1.5)	TCBC (6.0), DMAP (6.0)<br>Et_3N (6.0)	DCM (0.04)	rt	3 d	34

[a]Isolated yield after aqueous workup and chromatographic purification.

Table S3

Table 3 Silyl deprotection of **17** and **18** to obtain **2** and **1**.

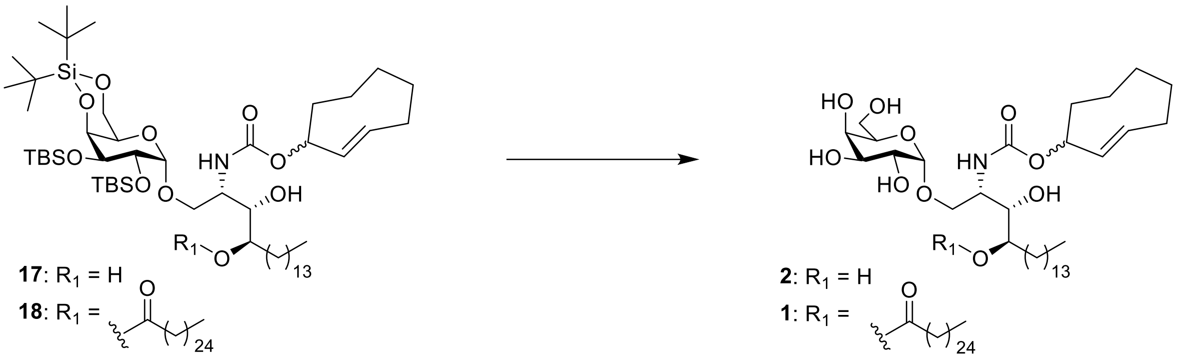

Entry	Compound (mmol)	Deprotection conditions (equiv)	Solvent (M)	Temp. (°C)	Time (h)	Yield (%)[a]
1	**17** (0.08)	HF · pyridine (excess)	-	rt	16	-
2	**17** (0.06)	HF · pyridine (10)	THF (0.03)	0	16	-
3	**17** (0.03)	HF · pyridine (10)	Pyridine (0.03)	rt	72	-
4	**17** (0.10)	TBAF (15)	THF (0.1)	rt	16	-
5	**17** (0.10)	TBAF (15), AcOH (4)	THF (0.1)	rt	16	-
6	**17** (0.10)	$Et_3N \cdot 3$ HF (6)	THF (0.1)	0 → rt	16	**2** (28)
7	**17** (0.56)	$Et_3N \cdot 3$ HF (6)	THF (0.1)	0 → rt	96	**2** (84)
8	**18** (0.30)	$Et_3N \cdot 3$ HF (10)	THF (0.1)	0 → rt	27	**1** (23)

[a]Isolated yield after aqueous workup and chromatographic purification.

3. Experimental Procedures – Immunology

3.1 Cell culture

iNKT activation assays were performed with DN32.D3 cells, an hybridoma iNKT cell line, kindly provided by Dr. Janneke N. Samsom. These cells were cultured in DMEM enriched with 1% Glutamax, 200 µg/mL penicillin and streptavidin, 10% FCS (all obtained from Sigma Aldrich) and Non-Essential Amino Acids (Gibco). The cells were split every 2-3 days and grown at 37 °C and 5% CO_2.

For presentation of the TCO α-GalCer (**1**) and TCO α-GalPhyto (**2**) antigens BMDCs from 8-12 weeks old male C57BL/6J mice were used. The use of the mice was approved by the animal welfare committee of Leiden University. The mice were offered by cervical dislocation and bone marrow was spun out of the cleaned tibia and femur. After red blood cell lysis with RBC-buffer (Gibco) and filtration through a 70 µm cell strainer (Falcon). The cells were plated in IMDM + 1% GlutaMAX™, 200 µg/mL penicillin and streptavidin, 10% FCS (all obtained from Sigma Aldrich) + 20 ng/mL GM-CSF (Peprotech) + 50 µM β-mercaptoethanol (Gibco). At day 2 extra medium is added, at day 4 the loosely adherend cells were split and at day 8 the cells were characterized with FACS and used for the experiments.

Characterization of the BMDCs was for each batch performed at day 8 with the following antibodies; Fc-block CD16/CD32 (1:100, BD Biosciences cat#553142), anti-CD11c-APC (1:200, Invitrogen cat #17-0114-81) anti-MHCII-FITC (1:500, Invitrogen cat #11-5321-81), anti-CD86-PE (1:66.7, Invitrogen 12-0861-81), anti-F4/80-FITC (1:66.7, BioLegend cat #123107) and anti-CD115-PE (1:200, BioLegend cat #135505). Flow cytometry was performed using the Guava EasyCyte HT Flow Cytometer. Data were analyzed using FlowJo™ software (version 10.7.2).

For presentation of mbTCO-SIINFEKL (**8**) and mbTCO-OVA18 (**6**) the D1 cell line was used for processing and presentation. These cells were cultured in IMDM enriched with 1% GlutaMAX™, 200 µg/mL penicillin and streptavidin, 10% FCS (all obtained from Sigma Aldrich), 50 µM 2-mercaptoethanol (Gibco) and, 30% fibroblast supernatant from NIH/3T3 cells[3] (collected from confluent cultures and filtered) containing 10-20 ng/ml mouse rGM-CSF (kindly provided by Prof. Ferry Ossendorp). The cells were split every 3-4 days and grown at 37 °C and 5% CO_2.

The B3Z hybridoma cell line was used to test the amount of SIINFEKL loaded MHC-I complexes. These cells were cultured in IMDM enriched with 1% GlutaMAX™, 200 µg/mL penicillin and streptavidin, 10% FCS (all obtained from Sigma Aldrich), 250 µM 2-mercaptoethanol (Gibco). The cells were split every 2-3 days and grown at 37 °C and 5% CO_2.

3.2 iNKT cell activation by IL-2 ELISA

BMDCs were seeded on a non-treated 96-well microtiter plate (50 000 cells per well) and allowed to settle for at least 2 hours at 37 °C, 5% CO_2. The cells were pulsed with TCO α-GalCer (**1**) or TCO α-GalPhyto (**2**) for two hours at indicated concentration, whereafter different tetrazines (**20, 21, 22, 23, 24, 25**) were added for distinct time periods and concentrations.

For internalization upfront the LysoTz (**3**, 10 µM) was added for one hour after which the samples were washed and incubated for another hour, then the ligands were added for one hour and again all samples were washed. Lysotracker tetrazine was added for 45 minutes after one hour incubation with the ligands as control. For all ELISA experiments DN32.D3 iNKT cells were added in 1:1 ratio O/N (~17 hours), and incubated at 37 °C, 5% CO_2. The next day the plates were spun down (5 min, 300 rcf, RT), supernatant was taken to a new plate and again spun down. Of this supernatant 30 µl was used for IL-2 ELISA (Invitrogen by Thermofisher Scientific).

3.3 B3Z T-cell activation assay

D1 cells were plated in 96-well tissue-culture treated microtiter plates (50,000 cells/well) and allowed to adhere at 37 °C for at least 2 hours. mbTCO-SIINFEKL (**8**, 100 nM; synthesis described in a prior publication[1]) or mbTCO-OVA18 (**6**, 20 µM) was added for 1 or 2 hour(s) respectively. Peptide-pulsed DCs were then treated with LysoTz (**3**, 10 µM) in full IMDM for 30 minutes unless indicated differently. After removal of the reduction medium, the cells were washed and T cell hybridoma B3Z cells (50,000 cells/well) were added in full IMDM. For lysosome specific uncaging the D1 cells were first loaded with LysoTz (**3**, 10 µM) for 30 minutes whereafter the remaining extracellular LysoTz (**3**) was washed away. The cells were left in the incubator for 30 minutes to assure lysosomal accumulation whereafter the mbTCO-SIINFEKL (**8**) or mbTCO-OVA18 (**6**) were respectively added for 1 and 2 hour(s). After removal of the reduction medium, the cells were washed and T cell hybridoma B3Z cells (50,000 cells/well) were added in full IMDM. In both set-ups the DCs and T cells were co-cultured O/N (~17 hours) at 37 °C. B3Z activity was measured by a colorimetric assay using CPRG (chlorophenol red-β-D-galactopyranoside) (Merck Millipore) as a substrate. To every well 100 µl of lysis buffer (9,6 ml PBS; 90 µl $MgCl_2$ 1M; 125 µl IGEPAL; 71 µl 2-Mercaptoethanol; ±6 mg CPRG) was added followed by short shaking of the plate and 2 hours incubation at 37°C in the dark. B3Z has the LacZ reporter gene, which produces the β-galactosidase enzyme, incorporated behind the IL-2 promoter. Upon activation of the T cell the IL-2 promoter will become activated thus producing IL-2 and β-galactosidase. The levels of expression of β-galactosidase correlate directly with the IL-2 levels and can be measured by the catalytic hydrolysis of the CPRG substrate from yellow to a dark red product, which was measured at the optical density of 570 nm on a CLARIOstar® *Plus* plate reader (BMG LABTECH).

3.3 Confocal microscopy

1.5E5 BMDCs were seeded per well (µ-slide 8-well ibiTreat) and treated with with 100 ng/mL LPS-EB (InvivoGen) O/N at 37 °C and 5% CO_2. The following day the cells were washed and medium was changed to IMDM without phenol red (Gibco) + 200 µg/mL penicillin and streptavidin and 10% FCS (both from Sigma Aldrich). LysoTz (**3**, 10 µM) was added for 30 min washed and the cells were incubated for 60 min. Thereafter DABCYL-TCO-BODIPY (**9**, 2 µM) was added for 30 min and washed. Last the cells were stained with 1x CellMask™ Orange Actin Tracking Stain (Invitrogen), 75 nM LysoTracker™ Deep Red (Invitrogen) and 5 µg/mL Hoechst 33342 (Thermo Fisher). Measurements were started after 15 minutes on an AR1 HD25 confocal microscope (Nikon), with CFI Apo LWD Lambda 40x/1.15 water immersion objective. Hoechst 33342, DABCYL-TCO-BODIPY (**9**) or TCO-BODIPY (**10**), CellMask™ Orange Actin tracking stain and LysoTracker™ deep red were excited using the 405 nm, 488 nm, 561 nm and 647 nm laser lines, respectively. Co-localization with the other organelles as the endoplasmic reticulum (1 µM, ER-Tracker™ TR, Thermo Fisher) and mitochondria (50 nM, MitoSpy™ Orange, BioLegend) were imaged on an AR1 HD25 confocal microscope (Nikon), equipped with a Ti2-E inverted microscope, LU-NV Series laser unit, and CFI Plan Apo Lambda 100x/1.45 oil objective, both with the 561 laser line. Z-stacks were made in seven steps of 0.20 micron and presented as maximum intensity projections. Brightness and contrast were adjusted for all samples using ImageJ. Brightness and contrast were adjusted identically for samples that are directly compared to each other to make sure the relative intensity between the samples remain the same. The Manders split coefficient and the Pearson's correlation coefficient were calculated with the ImageJ plug-in JaCoP[4] and averages were calculated of three representative images or cells.

3.4 Characterization DABCYL-TCO-BODIPY (9)

Turn-on experiment: A solution of **9** (1 µM) was prepared in H_2O (1% DMSO) and aliquoted in a black 96-well plate (100 µL per sample). LysoTz **3** was added in a 10 µM final concentration. The plate was immediately scanned for fluorescence on a CLARIOstar® *Plus* plate reader (BMG LABTECH) with excitation/emission at 477-14/530-40 nm and dichroic filter 497. Fluorescence was measured once every 3 seconds for 30 minutes. The results were processed and plotted in GraphPad Prism 9 showing the turn-on ratio between the sample and negative control, as an average of the triplicated samples and with indicated standard deviations.

Ex/Em spectra: Excitation and Emission spectra of (**9**) were measured at a final concentration of 1 µM in H_2O (1% DMSO). Spectra were measured in a black 96-well plate on a CLARIOstar® *Plus* plate reader (BMG LABTECH).

3.5 Fluorogenic TCO uncaging assay

The fluorogenic TCO uncaging assay was performed as previously described.[2] A black 96-well plate, was charged with 180 µL PBS (pH 7.2) or sodium citrate buffer (0.1 M, pH 4.5) at 25°C. 10 µL of a 0.2 mM solution of **48** in DMSO was added, followed by 10 µL of 2 mM solution of the desired tetrazine in DMSO was added. The fluorescence intensity (λex = 340-10 nm, λem = 495-10 nm, dichroic filter 417.5, gain 1500) was measured over a period of 1 hour at 1 minute intervals using a CLARIOstar® *Plus* plate reader (BMG LABTECH). Raw fluorescence was normalized using EDANS (10 µM) and TCO **48** (10 µM) as 100% and 0% reference values, respectively. Data was plotted using Graphpad Prism.

4. Experimental Procedures – Organic Synthesis

General methods: Commercially available reagents and solvents were used as received. Bodipy NHS **27**(CAS 146616-66-2) and 4-TCO-NHS **29** (CAS 1191901-33-3) were obtained from commercial sources. The syntheses of axial TCO carbonate **15**[1], Tetrazine **22**[5], Tetrazine **23**[6], Boc-protected tetrazine **25**[5], DABCYL-NHS **30**[2], bifunctional TCO reagent **32**,[2] and 2-methylsulfonylethyl N-succinimidyl carbonate (**45**)[1] were described in previous publications.

Moisture and oxygen sensitive reactions were performed under N_2 atmosphere (balloon). DCM, toluene, THF, dioxane and Et_2O were stored over (flame-dried) 4 Å molecular sieves (8-12 mesh). Methanol was stored over (flame-dried) 3 Å molecular sieves. Pyridine, DIPEA and Et_3N were stored over KOH pellets. TLC analysis was performed using aluminum sheets, pre-coated with silica gel (Merck, TLC Silica gel 60 F_{254}). Compounds were visualized by UV absorption (λ = 254 nm), by spraying with either a solution of $KMnO_4$ (20 g/L) and K_2CO_3 (10 g/L) in H_2O, a solution of $(NH_4)_6Mo_7O_{24} \cdot 4H_2O$ (25 g/L) and $(NH_4)_4Ce(SO_4)_4 \cdot 2H_2O$ (10 g/L) in 10% H_2SO_4, 20% H_2SO_4 in EtOH, or phosphomolybdic acid in EtOH (150 g/L), where appropriate, followed by charring at ca. 150°C. Column chromatography was performed on Screening Devices b.v. Silica Gel (particle size 40-63 µm, pore diameter 60 Å). Celite Hyflo Supercel (Merck) was used to impregnate the reaction mixture prior to silica gel chromatography when indicated. ^1H, ^{13}C APT, ^1H COSY, HSQC and HMBC spectra were recorded with a Bruker AV-400 (400/100 MHz), AV-500 (500/125 MHz) or AV-600 (600/150 MHz) spectrometer. Chemical shifts are reported as δ values (ppm) and were referenced to tetramethylsilane (δ = 0.00 ppm) or the residual solvent peak as internal standard. *J* couplings are reported in Hz.

LC-MS analysis was performed on a Finnigan Surveyor HPLC system (detection at 200-600 nm) with an analytical C_{18} column (Gemini, 50 x 4.6 mm, 3 µm particle size, Phenomenex) coupled to a Finnigan LCQ Advantage MAX ion-trap mass spectrometer (ESI^+). A notable exception are the spectra shown in Figure S5 A-D, which were recorded using a diphenyl column. The applied buffers were H_2O, MeCN and either 100 mM NH_4OAc in H_2O (10 mM NH_4OAc end concentration) or 1.0% TFA in H_2O (0.1% TFA end concentration). Methods used are: 10% → 90% MeCN, 13.5 min (0→0.5 min: 10% MeCN; 0.5→8.5 min: gradient time; 8.5→10.5 min: 90% MeCN; 10.5→13.5 min: 90% → 10% MeCN); 10% → 50% MeCN, 13.5 min (0→0.5 min: 10% MeCN; 0.5→8.5 min: gradient time; 8.5→10.5 min: 90% MeCN; 10.5→13.5 min: 90% → 10% MeCN); 0% → 50% MeCN, 13.5 min (0→0.5 min: 0% MeCN; 0.5→8.5 min: gradient time; 8.5→10.5 min: 50% MeCN; 10.5→13.5 min: 50% → 0% MeCN). HPLC purification was performed on a Gilson HPLC system (detection at 214 nm) coupled to a semi-preparative C_{18} column (Gemini, 250 x 10 mm, 5 µm particle size, Phenomenex). The applied buffers were H_2O, MeCN and either 100 mM NH_4OAc in H_2O (10 mM NH_4OAc end concentration) or 1.0% TFA in H_2O (0.1% TFA end concentration). High resolution mass spectra were recorded by direct injection (2 µL of a 2 µM solution in H_2O/MeCN 1:1 and 0.1% formic acid) on a mass spectrometer (Thermo Finnigan LTQ Orbitrap) equipped with an electrospray ion source in positive mode (source voltage 3.5 kV, sheath gas flow 10, capillary temperature 250°C) with resolution R = 60,000 at m/z 400 (mass range m/z = 150-2,000) and dioctylphthalate (m/z = 391.28428) as a “lock mass”. The high resolution mass spectrometer was calibrated prior to measurements with a calibration mixture (Thermo Finnigan).

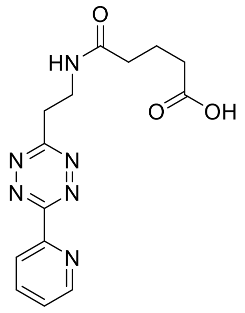

Compound 26: Boc-protected tetrazine **25**[5] (45.7 mg, 0.23 mmol, 1.0 equiv) was dissolved in 4M HCl in dioxane and stirred for 1 hour. The resulting suspension was centrifuged and the colorless supernatant was removed. The colorful precipitate was washed two times via re-suspension in 10 mL of dioxane, centrifugation, and partitioning from the colorless supernatant. The resulting pink solid was dissolved in acetonitrile. Triethylamine (0.32 mL, 2.3 mmol, 10 equiv) and glutaric anhydride were added and reaction mixture was stirred for 4 hours at room temperature. The reaction mixture was concentrated and dissolved in 20 mL ethyl acetate. The organic layer was washed with 1M HCl (3 x) and brine (3 x), dried over $MgSO_4$, filtered and concentrated *in vacuo*. The crude product was purified by silica gel chromatography (1 → 5% MeOH in DCM with 0.1% AcOH) to obtain **26** (52.9 mg, 0.17 mmol, 73%) as a pink solid: ^{1}H NMR (500 MHz, MeOD) δ 7.27 – 7.22 (m, 1H), 7.12 – 7.06 (m, 1H), 6.54 (td, *J* = 7.8, 1.7 Hz, 1H), 6.10 (ddd, *J* = 7.6, 4.8, 1.1 Hz, 1H), 2.22 (t, *J* = 6.4 Hz, 2H), 1.99 (t, *J* = 6.4 Hz, 2H), 1.72 (dt, *J* = 3.3, 1.6 Hz, 1H), 0.67 (t, *J* = 7.4 Hz, 2H), 0.58 (t, *J* = 7.5 Hz, 2H), 0.21 (p, *J* = 7.4 Hz, 2H); ^{13}C NMR (126 MHz, MeOD) δ 175.70, 170.35, 164.64, 151.37, 151.33, 139.46, 128.03, 125.25, 38.76, 36.16, 35.98, 34.25, 22.20.

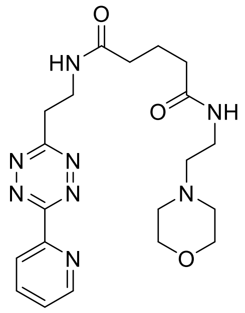

LysoTz 3: Compound **26** (10 mg, 31.6 µmol, 1.0 equiv) was dissolved in anhydrous DMF. PyBOP (24 mg, 47 µmol, 1.5 equiv) and DIPEA (8.2 µL, 47 µmol, 1.5 equiv) were added and reaction mixture was stirred for 5 minutes at room temperature. 4-(2-Aminoethyl)morpholine (6.2 µL, 47 µmol, 1.5 equiv) was added and the reaction mixture was stirred for 3 hours at room temperature. After completion on TLC, the reaction mixture was concentrated *in vacuo*. The reaction mixture was washed with 1M HCl (3 x) and brine (3 x), dried over $MgSO_4$, filtered and concentrated *in vacuo*. The crude product was purified by HPLC to LysoTz **3** (3.32 mg, 7.75 µmol, 25%) as a pink powder: ^{1}H NMR (500 MHz, $CDCl_3$) δ 8.93 (d, *J* = 4.6 Hz, 1H), 8.67 (d, *J* = 7.9 Hz, 1H), 8.00 (td, *J* = 7.8, 1.7 Hz, 1H), 7.61 – 7.54 (m, 1H), 6.76 – 6.67 (m, 1H), 6.59 (s, 1H), 3.90 (q, *J* = 6.1 Hz, 2H), 3.71 (t, *J* = 4.5 Hz, 4H), 3.66 – 3.59 (m, 2H), 3.39 (q, *J* = 5.7 Hz, 2H), 2.60 – 2.44 (m, 6H), 2.21 (q, *J* = 7.0 Hz, 4H), 1.90 (p, *J* = 7.0 Hz, 2H); ^{13}C NMR (126 MHz, $CDCl_3$) δ 173.12, 172.91, 169.03, 163.95, 150.97, 150.26, 137.70, 126.68, 124.26, 66.66 (x2), 57.48, 53.46 (x2), 37.69, 35.54, 35.41, 35.22, 35.17, 31.37, 21.73; HRMS: calculated for $C_{20}H_{29}N_3O_3$ 429.2363 $[M+H]^+$; found 429.2357.

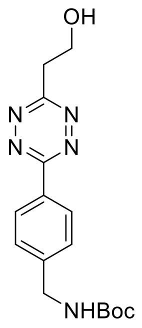

Boc-Tetrazine 50: A microwave vial was charged with tert-butyl (4-cyanobenzyl)carbamate (0.94 g, 4.06 mmol, 1.0 equiv), 3-hydroxypropionitrile (1.4 mL, 20.5 mmol, 5.0 equiv), $Zn(OTf)_2$ (0.38 g, 1.04 mmol, 0.26 equiv) and hydrazine monohydrate (10 mL, 200 mmol, 50 equiv). The microwave tube was capped, stirred for 10 min at room temperature, then stirred overnight at 60°C. The membrane was punctured, and the reaction mixture was slowly added to DCM/AcOH (80 mL, 1:1 v/v) whilst stirring. $NaNO_2$ was added portion-wise to the stirring mixture. The resulting mixture was concentrated *in vacuo* and redissolved in EtOAc. The organic layer was washed with $NaHCO_3$ (satd.), H_2O (2 x), dried over $MgSO_4$, filtered and concentrated *in vacuo*. The crude product was purified by silica gel chromatography (40% EtOAc in pentane → 60% EtOAc in pentane) to obtain **50** (0.32 g, 0.97 mmol, 25%) as a pink solid: R_f = 0.4 (50% EtOAc in pentane); ^{1}H NMR (400 MHz, $CDCl_3$) δ 8.43 (d, *J* = 8.1 Hz, 2H), 7.44 (d, *J* = 8.4 Hz, 2H), 5.44 (t, *J* = 5.4 Hz, 1NH), 4.40 (d, *J* = 5.9 Hz, 2H), 4.28 (t, *J* = 6.0 Hz, 2H), 3.59 (t, *J* = 5.9 Hz, 2H), 1.48 (s, 9H).

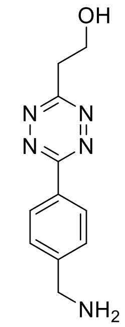

Tz 24: A 4 M solution of HCl in dioxane (135 µL, 0.54 mmol, 5.0 equiv) was added to a solution of Boc-tetrazine **50** (35 mg, 0.11 mmol, 1.0 equiv) in 0.4 mL anhydrous dioxane at room temperature and stirred for 2 hours. The precipitate was filtered, washed with Et_2O, redissolved in a minimal amount of MeOH and recrystallized from Et_2O yielding tetrazine **24** (17 mg, 0.07 mmol, 70 % yield): ^{1}H NMR (400 MHz, D_2O) δ 8.36 (d, *J* = 8.4 Hz, 2H), 7.64 (d, *J* = 8.4 Hz, 2H), 4.28 (s, 2H), 4.18 (t, *J* = 6.1 Hz, 2H), 3.55 (t, *J* = 6.2 Hz, 2H); ^{13}C NMR (101 MHz, D_2O) δ 167.9, 164.0, 137.3, 131.8, 129.7 (x2), 128.6 (x2), 59.4, 42.7, 37.0.

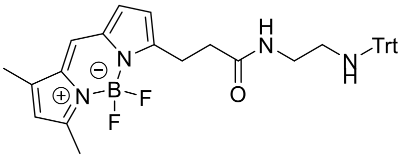

Compound 28: Bodipy-NHS **27** (1 mg, 2.56 µmol, 1.0 equiv) was dissolved in 0.5 mL dry DMF. N-tritylethylenediamine (1.2 mg, 3.84 µmol, 1.5 equiv) and DIPEA (2.3 µL, 12.8 µmol, 5 equiv) were added and the reaction mixture was stirred for 2 hours at room temperature. After completion of the reaction on TLC, the reaction mixture was concentrated *in vacuo*. The crude product was purified by silica gel chromatography (0 → 1 % MeOH in DCM) to obtain **28** (1.1 mg, 1.9 µmol, 75%) as an orange solid: ^{1}H NMR (500 MHz, $CDCl_3$) δ 7.43 – 7.39 (m, 6H), 7.24 (d, *J* = 7.9 Hz, 5H), 7.19 – 7.15 (m, 3H), 6.97 (s, 1H), 6.75 (d, *J* = 4.0 Hz, 1H), 6.26 (d, *J* = 4.0 Hz, 1H), 6.12 (s, 1H), 5.90 (t, *J* = 4.7 Hz, 1H), 3.32 (q, *J* = 5.9 Hz, 2H), 3.28 (t, *J* = 7.4 Hz, 2H), 2.65 (t, *J* = 7.4 Hz, 2H), 2.53 (s, 3H), 2.24 (s, 3H), 2.23 (t, *J* = 5.9 Hz, 2H).

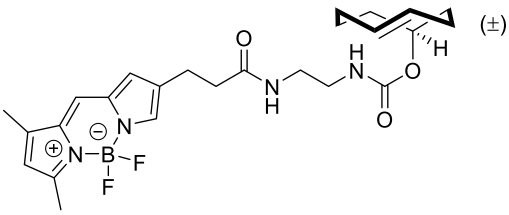

TCO-BODIPY 10: Compound **28** (0.55 mg, 0.95 µmol, 1.0 equiv) was dissolved in 1% TFA in DCM and stirred for 1 hour at room temperature. The reaction mixture was concentrated *in vacuo* and dissolved in DCM. 4-TCO-NHS **29** (0.27 mg, 1 µmol, 1.05 equiv) and DIPEA (0.8 µL, 4.75 µmol, 5.0 equiv) were added and reaction mixture was stirred for 3 hours at room temperature. After completion on TLC, the reaction mixture was concentrated *in vacuo*. The crude product was purified by silica gel chromatography (0 → 2% MeOH in DCM) to obtain **10** (0.4 mg, 0.8 µmol, 86%) as an orange solid: LC-MS (linear gradient 10 → 90% MeCN, 0.1% TFA, 11 min): R_t (min): 7.30 (ESI-MS (m/z): 486.83 (M+H$^+$)); HRMS: calculated for $C_{25}H_{34}BF_2N_4O_3$ [(M + H)$^+$]: 487.2692, found: 487.2687.

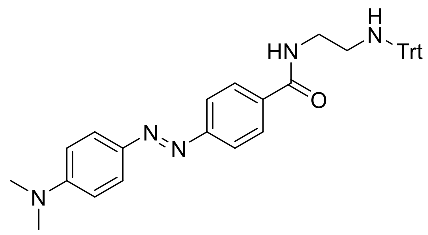

Compound 31: DABSYL-NHS **30**[2] (0.366 g, 1 mmol, 1.0 equiv) was dissolved in DCM. N-tritylethylenediamine (0.4 g, 1.3 mmol, 1.3 equiv) and DIPEA (0.870 mL, 5 mmol, 5.0 equiv) were added and the reaction mixture was stirred for 2 hours at room temperature. After completion on TLC, the reaction mixture was concentrated *in vacuo*. The crude product was purified by silica gel chromatography (1% MeOH in DCM, isocratic) to obtain **31** (568.7 mg, 1 mmol, 100%) as an orange powder: ^{1}H NMR (300 MHz, $CDCl_3$) δ 7.93 – 7.88 (m, 1H), 7.88 – 7.82 (m, 5H), 7.49 – 7.40 (m, 6H), 7.28 – 7.19 (m, 6H), 7.19 – 7.11 (m, 3H), 6.78 (t, *J* = 5.5 Hz, 1NH), 6.74 – 6.66 (m, 2H), 3.52 (q, *J* = 5.7 Hz, 2H), 3.03 (s, 6H), 2.40 (t, *J* = 5.9 Hz, 2H), 1.83 (s, 1NH); ^{13}C NMR (75 MHz, $CDCl_3$) δ 167.17, 155.05, 152.82, 145.80 (x3), 143.68, 134.72, 128.59 (x6), 127.98 (x6), 127.86 (x2), 126.48 (x3), 125.46 (x2), 122.31 (x2), 111.51 (x2), 70.77, 43.54, 40.67, 40.30 (x2).

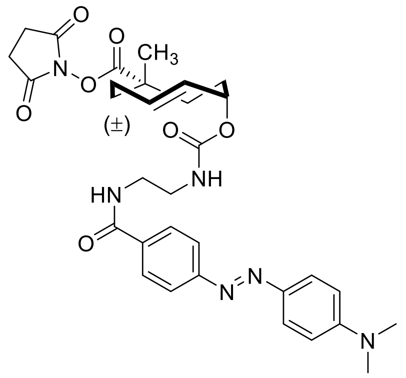

Compound 33: Compound **31** (6.7 mg, 12.5 µmol, 1.0 equiv) was dissolved in 1 % TFA in DCM and stirred for 1 hour. The reaction mixture was concentrated *in vacuo* and dissolved in DCM. Bifunctional TCO reagent **32**[2] (5 mg, 12.5 µmol, 1.0 equiv) and DIPEA (11 µL, 62.5 µmol, 5.0 equiv) were added and the reaction mixture was stirred for 2 hours. After completion on TLC, the reaction mixture was concentrated *in vacuo*. The crude product was purified by silica gel chromatography (1 → 2% MeOH in DCM) to obtain **33** (6 mg, 9.7 µmol, 77%) as an orange solid. Spectroscopic data was in agreement with previously published work.[2]

DABCYL-TCO-BODIPY 9: Compound **28** (3.35 mg, 6.7 µmol, 1.0 equiv) was dissolved in 1% TFA in DCM and stirred for 1 hour at room temperature. The reaction mixture was concentrated *in vacuo* and dissolved in 0.5 mL dry DMF. Compound **33** (6 mg, 9.7 µmol, 1.5 equiv) and DIPEA (5.8 µL, 33.5 µmol, 5 equiv) were added and the reaction mixture was stirred for 2 hours. After completion on TLC, the reaction was concentrated. The crude product was purified by silica gel chromatography (1 → 5% MeOH in DCM) to obtain **9** (2.87 mg, 3.4 µmol, 52%) as an orange solid: LC-MS (linear gradient 10 → 90% MeCN, 0.1% TFA, 11 min): R_t (min): 6.35 (ESI-MS (m/z): 838.08 ($M+H^+$)); HRMS: calculated for $C_{44}H_{54}BF_2N_9O_5$ [$(M + H)^+$]: 838.4387, found: 838.4389.

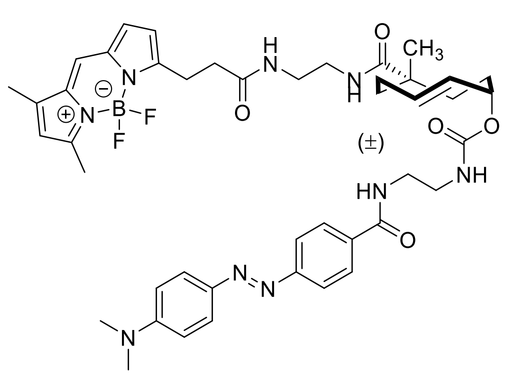

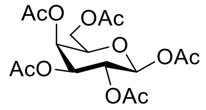

Peracetylated β-D-galactopyranoside 35: Synthesis was performed according to a modified procedure.[7] A suspension of sodium acetate (25.0 g, 305 mmol, 1.1 equiv) in acetic anhydride (350 mL, 3.71 mol, 13.4 equiv) was stirred in a three-neck, round-bottom flask and heated towards reflux in an oil bath set at 160°C. When the suspension was fully refluxing, the flask was removed from the oil bath and D-galactose (**34**, 50.0 g, 278 mmol, 1.0 equiv) was slowly added in portions to the mixture. The reaction mixture turned into a clear, yellow solution and was stirred for a further 5-10 min before pouring it into ice water (2 L). The aqueous mixture was stirred for 1 h at room temperature. DCM (600 mL) was added and the organic layer was washed with H_2O (1.5 L), $NaHCO_3$ (satd., 1.5 L), brine (1 L), dried over $MgSO_4$, filtered and concentrated *in vacuo*. The crude product was obtained as a light yellow solid and purified by recrystallization in EtOH to obtain **35** (56.4 g, 144 mmol, 52%) as white crystals: R_f = 0.4 (30% EtOAc in pentane); ^{1}H NMR (400 MHz, $CDCl_3$) δ 1H NMR (400 MHz, CDCl3) δ 5.71 (d, J = 8.3 Hz, 1H), 5.43 (dd, J = 3.4, 1.1 Hz, 1H), 5.34 (dd, J = 10.4, 8.3 Hz, 1H), 5.09 (dd, J = 10.4, 3.4 Hz, 1H), 4.21 – 4.03 (m, 3H), 2.17 (s, 3H), 2.13 (s, 3H), 2.05 (2 s, 6H), 2.00 (s, 3H); ^{13}C NMR (101 MHz, $CDCl_3$) δ 170.4, 170.2, 170.0, 169.5, 169.1, 92.2, 71.8, 70.9, 67.9, 66.9, 61.1, 20.9, 20.7, 20.7, 20.6; HRMS: calculated for $C_{16}H_{22}O_{11}Na$ 413.10543 $[M+Na]^+$; found 413.10521. Spectroscopic data was in agreement with literature.[7]

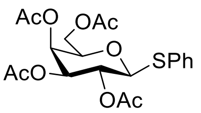

Thiogalactoside 36: Synthesis was performed according to a modified procedure.[7] β-D-galactose pentaacetate (**35**, 32.8 g, 84.0 mmol, 1.0 equiv) was dissolved in anhydrous DCM (~600 mL) under N_2. The solution was cooled down to 0°C before slowly adding thiophenol (12.9 mL, 126 mmol, 1.5 equiv) and boron trifluoride etherate (15.5 mL, 126 mmol, 1.5 equiv). The reaction mixture was stirred for 24 h and allowed to warm to room temperature. The reaction mixture was cooled to 0°C and quenched by adding Et_3N (20 mL, 143 mmol, 1.7 equiv) and subsequently washed with $NaHCO_3$ (satd., 1 L) and back-extracted with DCM (500 mL). The combined organic layers were washed with NaOH (5 % w/w, 1 L), dried over $MgSO_4$, filtered and concentrated *in vacuo.* The crude product was purified by silica gel chromatography (20% EtOAc in pentane → 30% EtOAc in pentane) to obtain **36** (35.2 g, 79.9 mmol, 95%) as a colorless waxy solid: R_f = 0.7 (50% EtOAc in pentane); ^{1}H NMR (400 MHz, $CDCl_3$) δ 7.55 – 7.48 (m, 2H), 7.35 – 7.29 (m, 3H), 5.42 (d, *J* = 2.7 Hz, 1H), 5.24 (t, *J* = 10.0 Hz, 1H), 5.05 (dd, *J* = 9.9, 3.3 Hz, 1H), 4.72 (d, *J* = 10.0 Hz, 1H), 4.20 (dd, *J* = 11.3, 7.0 Hz, 1H), 4.12 (dd, *J* = 11.3, 6.2 Hz, 1H), 3.94 (t, *J* = 6.6 Hz, 1H), 2.13 (s, 3H), 2.10 (s, 3H), 2.05 (s, 3H), 1.98 (s, 3H); ^{13}C NMR (101 MHz, $CDCl_3$) δ 170.5, 170.3, 170.2, 169.6, 132.7 (x2), 132.6, 129.0 (x2), 128.3, 86.8, 74.6, 72.1, 67.4, 67.3, 61.8, 21.0, 20.8, 20.8, 20.7; HRMS: calculated for $C_{20}H_{24}O_9SNa$ 463.10332 $[M+Na]^+$; found 463.10277. Spectroscopic data was in agreement with literature.[7]

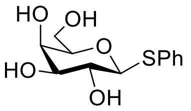

Thiogalactoside 37: Compound **36** (35.1 g, 79.8 mmol, 1.0 equiv) was dissolved in anhydrous MeOH (250 mL) under N_2. The pH of the reaction mixture was adjusted to > 10 by adding slowly adding sodium whilst stirring. The resulting reaction mixture was stirred overnight and subsequently neutralized by adding Amberlyst® (H^+ form, washed 3 x with MeOH prior to usage) in small portions, gently swirling the flask and monitoring the pH until neutral. The neutralized solution was filtered and concentrated *in vacuo* to obtain **37** (20.6 g, 75.6 mmol, 95%) as a colorless oil: R_f = 0.4 (20% MeOH in DCM); ^{1}H NMR (400 MHz, MeOD) δ 7.58 – 7.52 (m, 2H), 7.32 – 7.25 (m, 2H), 7.25 – 7.19 (m, 1H), 4.60 (d, *J* = 9.7 Hz, 1H), 3.91 (d, *J* = 3.2 Hz, 1H), 3.77 (dd, *J* = 11.4, 6.9 Hz, 1H), 3.71 (dd, *J* = 11.5, 5.2 Hz, 1H), 3.62 (t, *J* = 9.4 Hz, 1H), 3.57 (t, *J* = 6.1 Hz, 1H), 3.51 (dd, *J* = 9.2, 3.3 Hz, 1H); ^{13}C NMR (101 MHz, MeOD) δ 136.0, 132.0 (x2), 129.8 (x2), 127.9, 90.2, 80.5, 76.2, 70.9, 70.3, 62.5; HRMS: calculated for $C_{12}H_{16}O_5SNa$ 295.06107 $[M+Na]^+$; found 295.06106. Spectroscopic data was in agreement with literature.[8,9]

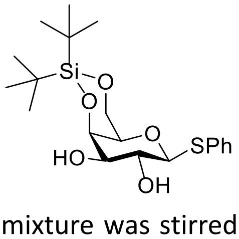

Thiogalactoside 38: compound **37** (15.8 g, 57.9 mmol, 1.0 equiv) was co-evaporated with anhydrous DMF (150 mL) in a 1 L round-bottom flask before dissolving the starting material in anhydrous DMF (240 mL) under N_2. The solution was cooled to -40°C before slowly adding di-*tert*-butylsilyl bis(trifluoromethanesulfonate) (24.2 g, 55.0 mmol, 0.95 equiv). The reaction mixture was stirred at -40°C for 30 min before adding anhydrous pyridine (14.1 mL, 174 mmol, 3.0 equiv). The reaction mixture was stirred for 45 min and was subsequently diluted with Et_2O (1 L), washed with H_2O (4 x 500 mL), brine (750 mL), dried over $MgSO_4$, filtered and concentrated *in vacuo*. The crude product was purified by silica gel chromatography (5% acetone in DCM, isocratic) to obtain the silylated product **38** (19.8 g, 48.0 mmol, 83%) as a clear viscous oil which crystallized under reduced pressure: R_f = 0.4 (5% acetone in DCM); ^{1}H NMR (500 MHz, $CDCl_3$) δ 7.58 – 7.52 (m, 2H), 7.33 – 7.25 (m, 3H), 4.56 (d, *J* = 9.8 Hz, 1H), 4.44 (d, *J* = 3.4 Hz, 1H), 4.29 – 4.22 (m, 2H), 3.75 (t, *J* = 9.3 Hz, 1H), 3.58 – 3.50 (m, 1H), 3.47 (s, 1H), 2.86 (br s, 2OH), 1.05 (s, 9H), 1.03 (s, 9H); ^{13}C NMR (126 MHz, $CDCl_3$) δ 133.2, 132.7 (x2), 129.0 (x2), 128.0, 89.1, 75.3, 75.2, 72.6, 70.7, 67.2, 27.6 (x3), 27.5 (x3), 23.4, 20.7;

HRMS: calculated for $C_{20}H_{32}O_5SSiNa$ 435.16319 $[M+Na]^+$; found 435.16279. Spectroscopic data was in agreement with literature.[10]

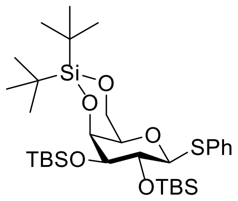

Thiogalactoside donor 12: Compound **38** (2.06 g, 5.0 mmol, 1.0 equiv) and DMAP (61 mg, 0.5 mmol, 0.1 equiv) were dissolved in anhydrous pyridine (20 mL) under N_2. The solution was cooled to 0°C before slowly adding TBS-OTf (4.59 mL, 20.0 mmol, 4.0 equiv). The reaction mixture was stirred for 16 h and allowed to warm to room temperature. The reaction mixture was concentrated *in vacuo*, diluted with 100 mL EtOAc, washed with HCl (1 M, 100 mL), $NaHCO_3$ (satd., 100 mL) and brine (100 mL). The aqueous layers were back-extracted with EtOAc (50 mL). The combined organic layers were dried over $MgSO_4$, filtered and concentrated *in vacuo*. The crude product was purified by silica gel chromatography (pentane → 20% DCM in pentane → 40% DCM in pentane) to obtain **12** (3.03 g, 4.73 mmol, 95%) as a clear oil: R_f = 0.3 (40% DCM in pentane); ^{1}H NMR (400 MHz, $CDCl_3$) δ 7.51 – 7.45 (m, 2H), 7.29 – 7.19 (m, 4H), 4.56 (d, *J* = 9.4 Hz, 1H), 4.32 (dd, *J* = 3.0, 1.0 Hz, 1H), 4.19 (dd, *J* = 12.2, 1.6 Hz, 1H), 4.15 (dd, *J* = 12.1, 1.7 Hz, 1H), 4.01 (t, *J* = 9.0 Hz, 1H), 3.52 (dd, *J* = 8.6, 2.8 Hz, 1H), 3.36 – 3.31 (m, 1H), 1.12 (s, 9H), 1.04 (s, 9H), 0.96 (s, 9H), 0.95 (s, 9H), 0.26 (s, 3H), 0.15 (s, 3H), 0.12 (s, 3H), 0.10 (s, 3H); ^{13}C NMR (101 MHz, $CDCl_3$) δ 136.0, 131.8 (x2), 128.8 (x2), 127.1, 90.6, 78.0, 74.8, 74.7, 70.4, 67.4, 27.9 (x3), 27.5 (x3), 26.7 (x3), 26.6 (x3), 23.6, 20.9, 18.4, 18.4, -1.9, -3.2, -3.3, -3.6; HRMS: calculated for $C_{36}H_{64}O_5SSi_3N$ 658.38075 $[M+NH_4]^+$; found 658.38031. Spectroscopic data was in agreement with literature.[10]

**Note:* this *procedure could also be performed at 10 gram scale (24 mmol) to obtain similar results.*

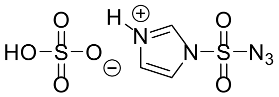

Imidazole-1-sulfonyl azide hydrogen sulfate (40): Synthesis was performed according to literature precedence.[11] Sodium azide (7.50 g, 115 mmol, 1.0 equiv) was placed in a 500 mL round-bottom flask and subsequently dissolved in anhydrous ethyl acetate (120 mL) under N_2. The suspension was cooled to 0°C before slowly adding sulfuryl chloride (9.38 mL, 115 mmol, 1.0 equiv) over 10 min. The yellow reaction mixture was stirred for 19 h and allowed to warm to room temperature. Subsequently, the reaction mixture was cooled to 0°C before slowly adding imidazole (14.9 g, 219 mmol, 1.9 equiv) over 5 min whilst maintaining an inert atmosphere. The reaction mixture was stirred for 3 h at 0°C before slowly adding $NaHCO_3$ (satd., 225 mL) to basify the reaction mixture. The organic layer was washed with H_2O (225 mL) and dried over $MgSO_4$. The dried organic phase was filtered, cooled to 0°C and placed under a continuous stream of N_2 before slowly adding sulfuric acid (6.15 mL, 115 mmol, 1.0 equiv) over 5 min. The acidified solution was stirred for 30 min and allowed to warm to room temperature. A colorless precipitate formed, which was collected by filtration to obtain **40** (22.5 g, 83.0 mmol, 72%) as a white solid. Spectroscopic data was in agreement with literature.[11]

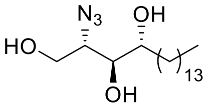

Phytosphingosine 41: D-*ribo*-phytosphingosine (**39**, 10.0 g, 31.5 mmol, 1.0 equiv) was suspended in a mixture of MeOH (300 mL) and DCM (100 mL) under N_2. K_2CO_3 (10.5 g, 76.0 mmol, 2.4 equiv) and Cu(II) · 5 H_2O (79 mg, 0.32 mmol, 1.0 mol%) were dissolved in H_2O (100 mL) and the resulting aqueous solution was added to the suspension to give a foamy reaction mixture. After 5 min, imidazole-1-sulfonyl azide hydrogen sulfate (**40**, 10.3 g, 37.8 mmol, 1.2 equiv) was added and the reaction mixture was stirred for 20 h at room temperature. The reaction mixture was partially concentrated *in vacuo* (≥ 100 mbar, 40°C) before adding HCl (1 M, 250 mL). The aqueous phase was extracted with EtOAc (3 x 350 mL, 40°C). The combined organic layers

were washed with $NaHCO_3$ (satd., 250 mL), brine (250 mL), dried over $MgSO_4$, filtered and concentrated *in vacuo* to obtain **41** (10.8 g, 31.5 mmol, 100%) as a solid which was used in the next step without further purification: R_f = 0.5 (10% MeOH in DCM); ^1H NMR (400 MHz, $CDCl_3$) δ 4.00 (dd, *J* = 11.7, 5.5 Hz, 1H), 3.89 (dd, *J* = 11.7, 4.5 Hz, 1H), 3.84 – 3.74 (m, 2H), 3.66 (q, *J* = 4.9 Hz, 1H), 1.65 – 1.44 (m, 3H), 1.38 – 1.21 (m, 23H), 0.88 (t, *J* = 7.0 Hz, 3H); ^{13}C NMR (101 MHz, $CDCl_3$) δ 74.7, 72.7, 63.2, 61.8, 32.1, 32.0, 29.8 (x2), 29.8, 29.8 (x2), 29.7, 29.7 (x2), 29.5, 25.9, 22.8, 14.3; HRMS: calculated for $C_{18}H_{38}N_3O_3$ 344.29077 $[M+H]^+$; found 344.29020. Spectroscopic data was in agreement with literature.[12,13]

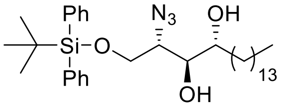

Phytosphingosine 42: Synthesis was performed according to a modified procedure.[14] Crude 2-azido-phytosphingosine (**41**, 10.8 g, 31.5 mmol, 1.0 equiv) was dissolved in anhydrous DCM (155 mL) and anhydrous DMF (35 mL) under N_2. The solution was cooled to 0°C before adding Et_3N (11.0 mL, 79.0 mmol, 2.5 equiv), DMAP (192 mg, 1.58 mmol, 0.1 equiv) and *tert*-butyldiphenylchlorosilane (TBDPS-Cl, 9.83 mL, 37.8 mmol, 1.2 equiv). The reaction mixture was stirred for 25 h and allowed to warm to room temperature. The reaction mixture was quenched with MeOH (1.53 mL, 37.8 mmol, 1.2 equiv) and diluted with EtOAc (1 L). The organic phase was washed with brine (2 x 600 mL) and the combined aqueous layers were back-extracted with EtOAc (500 mL). The combined organic layers were dried over $MgSO_4$, filtered and concentrated *in vacuo*. The crude product was purified by silica gel chromatography (pentane → 2.5% acetone in pentane → 10% acetone in pentane) to obtain **42** (15.1 g, 26.0 mmol, 83% over 2 steps) as an oil: R_f = 0.2 (5% acetone in pentane); ^1H NMR (400 MHz, $CDCl_3$) δ 7.74 – 7.64 (m, 4H), 7.51 – 7.35 (m, 6H), 4.03 (dd, J = 10.9, 4.2 Hz, 1H), 3.91 (dd, J = 11.0, 5.7 Hz, 1H), 3.72 – 3.64 (m, 2H), 3.59 – 3.53 (m, 1H), 2.52 (d, J = 4.1 Hz, 1OH), 2.00 (br s, 1OH), 1.57 – 1.37 (m, 3H), 1.37 – 1.20 (m, 23H), 1.08 (s, 9H), 0.88 (t, J = 6.8 Hz, 3H); ^{13}C NMR (101 MHz, $CDCl_3$) δ 135.8 (x2), 135.7 (x2), 132.7, 132.6, 130.2 (x2), 128.1 (x2), 128.0 (x2), 74.3, 72.5, 64.3, 63.5, 32.1, 32.0, 29.8, 29.8, 29.8, 29.8 (x2), 29.8, 29.7, 29.7, 29.5, 26.9 (x3), 25.8, 22.8, 19.2, 14.3; HRMS: calculated for $C_{34}H_{55}N_3O_3SiNa$ 604.39049 $[M+Na]^+$; found 604.39029. Spectroscopic data was in agreement with literature.[14]

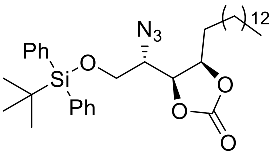

Phytosphingosine 43: Phytosphingosine **42** (8.40 g, 14.4 mmol, 1.0 equiv) was dissolved in anhydrous DCM (100 mL) under N_2. 1,1'-Carbonyldiimidazole (CDI, 7.02 g, 43.3 mmol, 3.0 equiv) was added and the reaction mixture was stirred for 72 h at room temperature. The reaction mixture was concentrated *in vacuo* and the resulting crude product was purified by silica gel chromatography (pentane → 5% Et_2O in pentane) to obtain **43** (6.95 g, 11.4 mmol, 79%) as a white solid: R_f = 0.2 (5% Et_2O in pentane); ^1H NMR (400 MHz, $CDCl_3$) δ 7.71 – 7.65 (m, 4H), 7.50 – 7.39 (m, 6H), 4.69 (ddd, *J* = 10.4, 7.2, 2.7 Hz, 1H), 4.54 (dd, *J* = 10.1, 7.2 Hz, 1H), 4.03 (dd, *J* = 11.1, 2.7 Hz, 1H), 3.88 (dd, *J* = 11.1, 6.1 Hz, 1H), 3.61 (ddd, *J* = 9.7, 6.1, 2.7 Hz, 1H), 1.80 – 1.68 (m, 1H), 1.68 – 1.55 (m, 2H), 1.48 – 1.19 (m, 23H), 1.09 (s, 9H), 0.88 (t, *J* = 6.8 Hz, 3H); ^{13}C NMR (101 MHz, $CDCl_3$) δ 135.7 (x2), 135.7 (x2), 132.5, 132.3, 130.3, 130.2, 128.1 (x2), 128.1 (x2), 79.6, 75.6, 64.3, 60.0, 32.1, 29.8, 29.8, 29.8 (x2), 29.7, 29.6, 29.5, 29.5, 29.3, 29.0, 26.9 (x3), 25.6, 22.8, 19.3, 14.3.

*Note: the ^{13}C signal for the carbonate protecting group (C=O) was not reported due to a lack of resolution on the spectrum of **43**.*

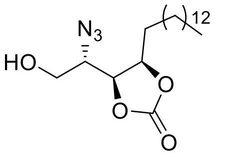

Phytosphingosine acceptor 13: Phytosphingosine **43** (6.95 g, 11.4 mmol, 1.0 equiv) was dissolved in HF · pyridine (10.3 mL, 114 mmol, 10 equiv) in a plastic tube under N_2. The reaction mixture was stirred for 22 h at room temperature. The reaction mixture was slowly added to $NaHCO_3$ (satd., 50 mL) and the resulting mixture was extracted with DCM (3 x 50 mL). The combined organic layers were washed with $CuSO_4$ (1 M, 3 x 30 mL), H_2O (30 mL), dried over $MgSO_4$, filtered and concentrated *in vacuo*. The crude product was purified by silica gel chromatography (20% EtOAc in pentane, isocratic) to obtain acceptor **13** (3.88 g, 10.5 mmol, 92%) as a white solid: R_f = 0.3 (20% EtOAc in pentane); ^{1}H NMR (400 MHz, $CDCl_3$) δ 4.76 (ddd, *J* = 10.4, 7.3, 2.9 Hz, 1H), 4.62 (dd, *J* = 9.9, 7.3 Hz, 1H), 4.08 (dd, *J* = 11.9, 2.4 Hz, 1H), 3.91 (dd, *J* = 11.9, 5.5 Hz, 1H), 3.70 (ddd, *J* = 9.8, 5.4, 2.7 Hz, 1H), 3.38 (br s, 1OH), 1.84 – 1.65 (m, 2H), 1.64 – 1.53 (m, 1H), 1.49 – 1.18 (m, 23H), 0.88 (t, *J* = 6.8 Hz, 3H); ^{13}C NMR (101 MHz, $CDCl_3$) δ 154.2, 79.9, 75.8, 62.3, 59.8, 32.0, 29.7, 29.7, 29.7 (x2), 29.6, 29.6, 29.4, 29.4, 29.2, 28.9, 25.6, 22.7, 14.1; HRMS: calculated for $C_{19}H_{36}N_3O_4$ 370.27003 $[M+H]^+$; found 370.26988. Spectroscopic data was in agreement with literature.[15]

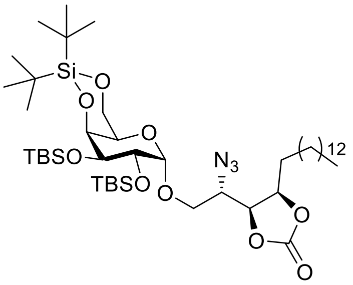

Compound 11: Galactose donor **12** (5.07 g, 7.91 mmol, 1.5 equiv) and phytosphingosine acceptor **13** (1.95 g, 5.27 mmol, 1.0 equiv) were co-evaporated with toluene (3 x 3 mL) before dissolving the reactants in anhydrous DCM (40 mL) in the presence of flame-dried molecular sieves (3Å) under N_2. After 15 min, the reaction mixture was cooled to -40°C before adding *N*-iodosuccinimide (NIS, 1.78 g, 7.91 mmol, 1.5 equiv) and trimethylsilyl trifluoromethanesulfonate (TMS-OTf, 191 µL, 1.05 mmol, 0.2 equiv). The reaction mixture was stirred for 5 h at -40°C and subsequently quenched by adding Et_3N (7.35 mL, 52.7 mmol, 10 equiv). The crude mixture was diluted with EtOAc (250 mL), washed with $NaHCO_3$ (satd.,150 mL), $Na_2S_2O_3$ (satd., 150 mL) and brine (150 mL), dried over $MgSO_4$, filtered, impregnated with Celite and concentrated *in vacuo*. The impregnated crude product was purified by silica gel chromatography (pentane → 2% EtOAc in pentane → 5% EtOAc in pentane) to obtain the glycosylated product **11** (3.19 g, 3.54 mmol, 67%) as a yellow oil: R_f = 0.3 (5% EtOAc in pentane); ^{1}H NMR (400 MHz, $CDCl_3$) δ 4.83 (d, *J* = 3.4 Hz, 1H), 4.77 – 4.70 (m, 2H), 4.32 (d, *J* = 2.2 Hz, 1H), 4.26 (dd, *J* = 12.5, 1.8 Hz, 1H), 4.18 – 4.12 (m, 3H), 3.87 (dd, *J* = 9.6, 2.9 Hz, 1H), 3.72 – 3.65 (m, 2H), 3.62 – 3.54 (m, 1H), 1.86 – 1.74 (m, 1H), 1.73 – 1.55 (m, 2H), 1.47 – 1.19 (m, 23H), 1.04 (s, 9H), 1.04 (s, 9H), 0.93 (s, 9H), 0.91 (s, 9H), 0.88 (t, *J* = 6.7 Hz, 3H), 0.09 (s, 3H), 0.09 (s, 3H), 0.07 (s, 6H); ^{13}C NMR (101 MHz, $CDCl_3$) δ 153.5, 101.4, 79.5, 75.3, 74.9, 70.9, 69.2, 68.4, 68.3, 67.3, 57.9, 32.1, 29.8, 29.8, 29.8 (x2), 29.7, 29.6, 29.5, 29.5, 29.3, 29.0, 27.6 (x3), 27.5 (x3), 26.2 (x3), 26.1 (x3), 25.6, 23.6, 22.8, 20.8, 18.2, 18.2, 14.3, -4.0, -4.1, -4.3, -4.5; HRMS: calculated for $C_{45}H_{93}N_4O_9Si_3$ 917.62449 $[M+NH_4]^+$; found 917.62451.

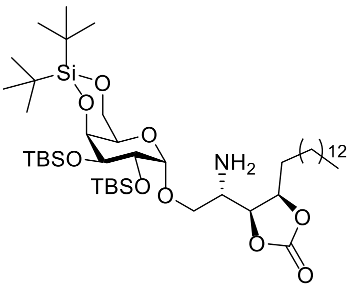

Compound 14: Azide **11** (1.34 g, 1.49 mmol, 1 equiv) was dissolved anhydrous THF (30 mL) under N_2. N_2 was purged through the stirring solution for 15 min (flow) before adding PtO_2 (101 mg, 0.45 mmol, 0.3 equiv) and purging N_2 through the stirred suspension for 15 min (flow). The reaction mixture was purged with H_2 (balloon) whilst stirring and was subsequently left to stir under H_2 (balloon) for 24 h. The reaction mixture was purged with N_2 (flow), filtered over a pad of Celite and concentrated *in vacuo* to obtain the crude amine **14** (1.31 g) as a yellow oil which was used in the next step without further purification: R_f = 0.2 (15% EtOAc in pentane); ^{1}H

NMR (400 MHz, $CDCl_3$) δ 4.80 (d, *J* = 3.4 Hz, 1H), 4.75 – 4.68 (m, 1H), 4.50 (dd, *J* = 9.8, 7.2 Hz, 1H), 4.31 (d, *J* = 2.4 Hz, 1H), 4.23 (dd, *J* = 12.4, 1.6 Hz, 1H), 4.15 – 4.09 (m, 2H), 3.87 – 3.81 (m, 2H), 3.61 (br s, 1H), 3.38 (dd, *J* = 10.0, 5.8 Hz, 1H), 3.16 (ddd, *J* = 9.2, 5.7, 3.1 Hz, 1H), 1.91 – 1.81 (m, 1H), 1.72 – 1.54 (m, 2H), 1.41 – 1.19 (m, 23H), 1.03 (s, 9H), 1.03 (s, 9H), 0.93 (s, 9H), 0.89 (s, 9H), 0.87 (t, *J* = 7.0 Hz, 3H), 0.09 (s, 6H), 0.08 (s, 3H), 0.07 (s, 3H); ^{13}C NMR (101 MHz, $CDCl_3$) δ 154.4, 101.2, 80.2, 78.9, 74.9, 71.2, 70.9, 69.5, 68.0, 67.3, 49.7, 32.0, 29.8, 29.8, 29.8 (x2), 29.7, 29.6, 29.6, 29.5, 29.4, 28.8, 27.6 (x3), 27.4 (x3), 26.2 (x3), 26.1 (x3), 25.6, 23.5, 22.8, 20.8, 18.2, 18.2, 14.2, -3.9, -4.2, -4.2, -4.5; HRMS: calculated for $C_{45}H_{92}NO_9Si_3$ 874.60744 $[M+H]^+$; found 874.60676.

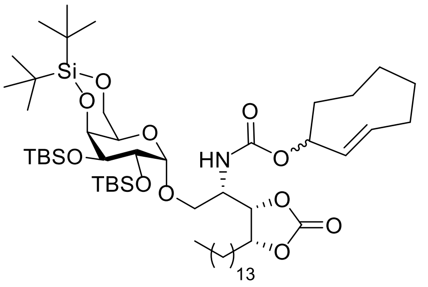

Compound 16: The crude amine **14** (1.31 g) obtained in the previous hydrogenation step and axial TCO carbonate **15** (481 mg, 1.80 mmol, 1.2 equiv) were dissolved in anhydrous DMF (15 mL) under N_2. DIPEA (0.39 mL, 2.25 mmol, 1.5 equiv) and DMAP (37 mg, 0.30 mmol, 0.2 equiv) were added and the reaction mixture was stirred for 21 h at room temperature. Subsequently, EtOAc (100 mL) was added and the organic phase was washed with HCl (1 M, 80 mL), $NaHCO_3$ (satd., 3 x 80 mL), brine (80 mL), dried over $MgSO_4$, filtered and concentrated *in vacuo*. The crude product was purified by silica gel chromatography (7% EtOAc in pentane, isocratic) to obtain the diastereomeric mixture **16** (**16**$_A$: **16**$_B$, ~ **1** : **1**, 1.36 g, 1.32 mmol, 89% over two steps) as a yellow oil: R_f = 0.3 (10% EtOAc in pentane); ^{1}H NMR (400 MHz, $CDCl_3$) δ 5.81 – 5.67 (m, 1H, **16**$_A$ + **16**$_B$), 5.54 (dd, *J* = 16.6, 1.9 Hz, 1H, **16**$_A$ + **16**$_B$), 5.41 – 5.20 (m, 1H + 1NH, **16**$_A$ + **16**$_B$), 4.82 (d, *J* = 3.5 Hz, 1H, **16**$_A$ + **16**$_B$), 4.81 – 4.76 (m, 1H, **16**$_A$ + **16**$_B$), 4.75 – 4.67 (m, 1H, **16**$_A$ + **16**$_B$), 4.32 (br s, 1H, **16**$_A$ + **16**$_B$), 4.21 (d, *J* = 12.6 Hz, 1H, **16**$_A$ + **16**$_B$), 4.17 – 4.04 (m, 3H, **16**$_A$ + **16**$_B$), 3.85 (d, *J* = 9.4 Hz, 1H, **16**$_A$ + **16**$_B$), 3.81 – 3.73 (m, 1H, **16**$_A$ + **16**$_B$), 3.68 (dd, *J* = 10.4, 2.8 Hz, 1H, **16**$_A$ + **16**$_B$), 3.59 (br s, 1H, **16**$_A$ + **16**$_B$), 2.52 – 2.39 (m, 1H, **16**$_A$ + **16**$_B$), 2.08 – 1.95 (m, 3H, **16**$_A$ + **16**$_B$), 1.94 – 1.82 (m, 1H, **16**$_A$ + **16**$_B$), 1.81 – 1.20 (m, 30H, **16**$_A$ + **16**$_B$), 1.04 (s, 18H, **16**$_A$ + **16**$_B$), 0.95 (s, 9H, **16**$_A$), 0.94 (s, 9H, **16**$_B$), 0.91 (s, 9H, **16**$_A$ + **16**$_B$), 0.88 (t, *J* = 6.9 Hz, 3H, **16**$_A$ + **16**$_B$), 0.84 – 0.73 (m, 1H, **16**$_A$ + **16**$_B$), 0.13 (s, 3H, **16**$_A$), 0.12 (s, 3H, **16**$_A$ + **16**$_B$), 0.11 (s, 3H, **16**$_B$), 0.10 (s, 3H, **16**$_A$ + **16**$_B$), 0.09 (s, 3H, **16**$_A$ + **16**$_B$); ^{13}C NMR (101 MHz, $CDCl_3$) δ 155.0, 155.0, 153.9 (x2), 131.9 (x2), 131.4, 131.2, 101.4, 101.3, 79.9, 79.8, 77.7, 77.3, 74.8, 74.8, 74.6, 74.6, 71.2, 71.1, 69.4, 69.4, 68.2, 68.1, 67.2, 67.2, 67.1, 67.1, 49.4 (x2), 40.8, 40.7, 36.0, 36.0, 36.0, 35.9, 32.0 (x2), 29.8 (x2), 29.8 (x2), 29.8 (x4), 29.7 (x2), 29.7 (x2), 29.6 (x2), 29.5 (x2), 29.5 (x2), 29.2 (x2), 29.1, 29.0, 28.6, 28.6, 27.5 (x6), 27.4 (x6), 26.2 (x6), 26.1 (x3), 26.1 (x3), 25.7, 25.6, 24.2, 23.5 (x2), 22.8 (x2), 20.8 (x2), 18.2, 18.2 (x2), 14.2 (x2), -3.8, -3.9, -4.1 (x2), -4.2 (x2), -4.6 (x2); HRMS: calculated for $C_{54}H_{104}NO_{11}Si_3$ 1026.69117 $[M+H]^+$; found 1026.69013.

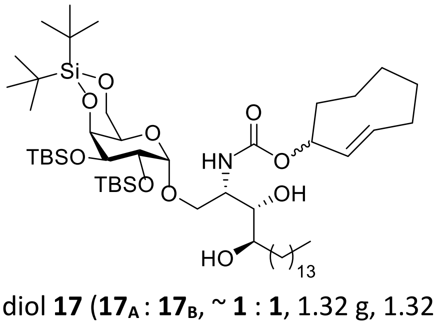

Compound 17: Carbonate **16** (1.36 g, 1.32 mmol, 1.0 equiv) was dissolved in a mixture of THF (7.5 mL) and H_2O (2.5 mL) under N_2. The solution was cooled to 0°C before adding LiOH (253 mg, 10.6 mmol, 8.0 equiv). The reaction mixture was stirred for 24 h and allowed to warm to room temperature. The pH of the reaction mixture was neutralized by adding dry ice. Subsequently, the reaction mixture was concentrated *in vacuo* to obtain the crude diol **17** (**17**$_A$: **17**$_B$, ~ **1** : **1**, 1.32 g, 1.32 mmol, quant.) as an oil which was used for the next step without further purification: R_f = 0.4 (10% EtOAc in pentane); ^{1}H NMR (400 MHz, $CDCl_3$) δ 5.86 – 5.65 (m, 1H, **17**$_A$ + **17**$_B$), 5.59 – 5.40 (m, 1H + 1NH, **17**$_A$ + **17**$_B$), 5.39 – 5.26 (m, 1H, **17**$_A$ + **17**$_B$), 4.89 – 4.81 (m, 1H, **17**$_A$ + **17**$_B$), 4.30 (d, *J* = 2.0 Hz, 1H, **17**$_A$ + **17**$_B$), 4.24 – 4.05 (m, 4H, **17**$_A$ + **17**$_B$), 4.02 – 3.87 (m, 1H, **17**$_A$ + **17**$_B$),

3.79 (td, *J* = 9.5, 8.5, 3.0 Hz, 1H, $\mathbf{17_A}$ + $\mathbf{17_B}$), 3.68 (d, *J* = 10.2 Hz, 1H, $\mathbf{17_A}$ + $\mathbf{17_B}$), 3.62 – 3.46 (m, 3H, $\mathbf{17_A}$ + $\mathbf{17_B}$), 2.49 – 2.40 (m, 1H, $\mathbf{17_A}$ + $\mathbf{17_B}$), 2.08 – 1.93 (m, 3H, $\mathbf{17_A}$ + $\mathbf{17_B}$), 1.92 – 1.21 (m, 31H, $\mathbf{17_A}$ + $\mathbf{17_B}$), 1.03 (s, 18H, $\mathbf{17_A}$ + $\mathbf{17_B}$), 0.95 (s, 9H, $\mathbf{17_A}$), 0.94 (s, 9H, $\mathbf{17_B}$), 0.92 (s, 9H, $\mathbf{17_A}$ + $\mathbf{17_B}$), 0.88 (t, *J* = 6.8 Hz, 3H, $\mathbf{17_A}$ + $\mathbf{17_B}$), 0.85 – 0.72 (m, 1H, $\mathbf{17_A}$ + $\mathbf{17_B}$), 0.12 (s, 9H, $\mathbf{17_A}$ + $\mathbf{17_B}$), 0.10 (s, 3H, $\mathbf{17_A}$ + $\mathbf{17_B}$); ^{13}C NMR (101 MHz, $CDCl_3$) δ 155.5, 155.3, 132.0, 132.0, 131.6, 131.4, 100.4, 100.1, 77.0, 76.9, 74.8, 74.8, 74.2, 73.9, 73.2 (x2), 71.6, 71.5, 69.4, 69.3, 68.3, 68.2, 67.2, 67.1, 66.7 (x2), 50.7, 50.5, 40.9, 40.9, 36.1, 36.1, 36.0 (x2), 34.3, 34.2, 32.1 (x2), 29.9 (x2), 29.8 (x10), 29.8 (x4), 29.8 (x2), 29.5 (x2), 29.2, 29.1, 27.6 (x6), 27.4 (x6), 26.3 (x6), 26.2 (x6), 24.2 (x2), 23.5, 23.5, 22.8 (x2), 20.8 (x2), 18.4, 18.2, 14.3 (x2), -3.8, -3.8, -4.1 (x2), -4.3, -4.3, -4.5 (x2); HRMS: calculated for $C_{53}H_{106}NO_{10}Si_3$ 1000.71190 $[M+H]^+$; found 1000.71102.

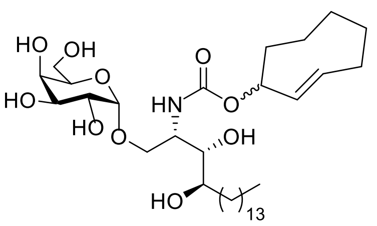

TCO Caged αGalPhs (2): The crude diol **17** (562 mg, 0.56 mmol, 1.0 equiv) was dissolved in anhydrous THF (5.6 mL) under N_2. The solution was cooled to 0°C before adding $Et_3N \cdot 3HF$ (0.55 mL, 3.40 mmol, 6.0 equiv). The reaction mixture was stirred for 96 h and allowed to warm to room temperature. Subsequently, the reaction mixture was concentrated *in vacuo*, redissolved in distilled EtOAc (150 mL), washed with H_2O (2 x 100 mL), brine (100 mL), dried over $MgSO_4$, filtered, impregnated with Celite and concentrated *in vacuo*. The impregnated crude product was purified by silica gel chromatography (1% MeOH in DCM → 2.5% MeOH in DCM → 5% MeOH in DCM → 10% MeOH in DCM) to obtain caged αGalPhs **2** ($\mathbf{2_A}$: $\mathbf{2_B}$, ~ **1** : **1**, 297 mg, 0.47 mmol, 84%) as a crystalline solid: R_f = 0.25 (10% MeOH in DCM); ^1H NMR (600 MHz, Pyridine-d_5) δ 7.94 (d, *J* = 9.0 Hz, 1NH, $\mathbf{2_A}$), 7.89 (d, *J* = 8.8 Hz, 1NH, $\mathbf{2_B}$), 7.11 – 5.95 (m, 6OH, $\mathbf{2_A}$ + $\mathbf{2_B}$), 5.88 (ddd, *J* = 15.1, 11.5, 3.2 Hz, 1H, $\mathbf{2_A}$), 5.82 (ddd, *J* = 15.2, 11.5, 3.2 Hz, 1H, $\mathbf{2_B}$), 5.61 (br s, 1H, $\mathbf{2_A}$ + $\mathbf{2_B}$), 5.59 (d, *J* = 14.2 Hz, 1H, $\mathbf{2_A}$), 5.53 (d, *J* = 16.2 Hz, 1H, $\mathbf{2_B}$), 5.48 (d, *J* = 3.4 Hz, 1H, $\mathbf{2_A}$ + $\mathbf{2_B}$), 4.94 – 4.85 (m, 1H, $\mathbf{2_A}$ + $\mathbf{2_B}$), 4.66 – 4.60 (m, 1H, $\mathbf{2_A}$ + $\mathbf{2_B}$), 4.59 – 4.55 (m, 1H, $\mathbf{2_A}$ + $\mathbf{2_B}$), 4.54 – 4.46 (m, 1H, $\mathbf{2_A}$ + $\mathbf{2_B}$), 4.43 – 4.18 (m, 7H, $\mathbf{2_A}$ + $\mathbf{2_B}$), 2.37 – 2.28 (m, 1H, $\mathbf{2_A}$ + $\mathbf{2_B}$), 2.27 – 2.18 (m, 1H, $\mathbf{2_A}$ + $\mathbf{2_B}$), 2.16 – 2.09 (m, 1H, $\mathbf{2_A}$ + $\mathbf{2_B}$), 1.99 – 1.79 (m, 4H, $\mathbf{2_A}$ + $\mathbf{2_B}$), 1.77 – 1.68 (m, 1H, $\mathbf{2_A}$ + $\mathbf{2_B}$), 1.68 – 1.58 (m, 2H, $\mathbf{2_A}$ + $\mathbf{2_B}$), 1.56 – 1.48 (m, 1H, $\mathbf{2_A}$ + $\mathbf{2_B}$), 1.48 – 1.15 (m, 23H, $\mathbf{2_A}$ + $\mathbf{2_B}$), 1.13 – 1.01 (m, 1H, $\mathbf{2_A}$ + $\mathbf{2_B}$), 0.92 (t, *J* = 7.1 Hz, 3H, $\mathbf{2_A}$ + $\mathbf{2_B}$), 0.76 – 0.66 (m, 1H, $\mathbf{2_A}$ + $\mathbf{2_B}$); ^{13}C NMR (151 MHz, Pyridine-d_5) δ 157.1, 157.1, 133.3, 133.0, 132.4, 132.0, 101.8, 101.7, 77.1, 77.1, 74.4, 74.4, 73.3, 73.3, 73.0, 72.9, 72.0 (x2), 71.5, 71.5, 70.7, 70.7, 68.8 (x2), 63.1, 63.1, 53.5 (x2), 41.6, 41.6, 36.8 (x2), 36.7, 36.6, 34.9, 34.8, 32.8 (x2), 31.0, 31.0, 30.8, 30.8, 30.7 (x2), 30.7 (x8), 30.6 (x2), 30.3 (x2), 29.8, 29.8, 27.1, 27.1, 25.0, 25.0, 23.6 (x2), 15.0 (x2); ^1H NMR (500 MHz, Dioxane-d_8) δ 6.16 – 5.96 (m, 1NH, $\mathbf{2_A}$ + $\mathbf{2_B}$), 5.86 – 5.69 (m, 1H, $\mathbf{2_A}$ + $\mathbf{2_B}$), 5.53 (d, *J* = 16.4 Hz, 1H, $\mathbf{2_A}$ + $\mathbf{2_B}$), 5.27 (br s, 1H, $\mathbf{2_A}$ + $\mathbf{2_B}$), 4.84 (d, *J* = 4.2 Hz, 1H, $\mathbf{2_A}$), 4.83 (d, *J* = 3.9 Hz, 1H, $\mathbf{2_B}$), 4.04 – 3.94 (m, 1H, $\mathbf{2_A}$ + $\mathbf{2_B}$), 3.92 – 3.70 (m, 4H, $\mathbf{2_A}$ + $\mathbf{2_B}$), 3.68 – 3.57 (m, 4H, $\mathbf{2_A}$ + $\mathbf{2_B}$), 3.49 – 3.37 (m, 2H, $\mathbf{2_A}$ + $\mathbf{2_B}$), 2.51 – 2.38 (m, 1H, $\mathbf{2_A}$ + $\mathbf{2_B}$), 2.08 – 1.90 (m, 3H, $\mathbf{2_A}$ + $\mathbf{2_B}$), 1.89 – 1.78 (m, 1H, $\mathbf{2_A}$ + $\mathbf{2_B}$), 1.72 – 1.56 (m, 3H, $\mathbf{2_A}$ + $\mathbf{2_B}$), 1.56 – 1.42 (m, 2H, $\mathbf{2_A}$ + $\mathbf{2_B}$), 1.41 – 1.19 (m, 24H, $\mathbf{2_A}$ + $\mathbf{2_B}$), 1.13 – 1.01 (m, 1H, $\mathbf{2_A}$ + $\mathbf{2_B}$), 0.88 (t, *J* = 6.9 Hz, 3H, $\mathbf{2_A}$ + $\mathbf{2_B}$), 0.86 – 0.79 (m, 1H, $\mathbf{2_A}$ + $\mathbf{2_B}$); ^{13}C NMR (126 MHz, Dioxane-d_8) δ 156.0 (x2), 133.3, 133.1, 131.8, 131.5, 100.7 (x2), 76.7, 76.6, 74.3, 74.3, 72.5, 72.5, 72.3, 72.2, 71.5 (x2), 70.6, 70.5, 70.2 (x2), 68.2, 68.1, 62.6, 62.6, 52.5 (x2), 41.2, 41.2, 36.8 (x2), 36.4, 36.4, 34.2, 34.2, 32.7 (x2), 30.6, 30.6, 30.6, 30.5 (x5), 30.5 (x6), 30.4 (x2), 30.1 (x2), 29.8, 29.7, 26.6 (x2), 25.1, 25.0, 23.4 (x2), 14.4 (x2); HRMS: calculated for $C_{33}H_{62}NO_{10}$ 632.43682 $[M+H]^+$; found 632.43640. Compound **2** was redissolved in dioxane and lyophilized in small quantities for immunology experiments.

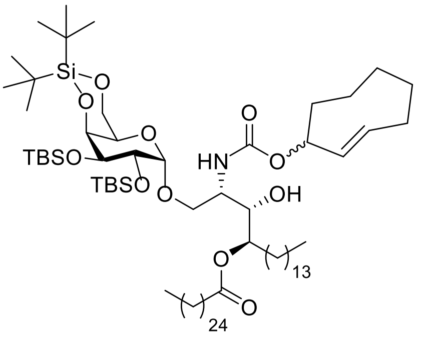

Compound 18: Hexacosanoic acid (60 mg, 0.15 mmol, 1.5 equiv), EDC · HCl (29 mg, 0.15 mmol, 1.5 equiv) and DMAP (73 mg, 0.60 mmol, 6 equiv) were dissolved in anhydrous DCM (1.0 mL) under N_2. The suspension was cooled to 0°C and stirred for 45 min. A solution of compound **17** (100 mg, 100 µmol, 1.0 equiv) in anhydrous DCM (2.0 mL) under N_2 was subsequently added to the reaction mixture. DIPEA (52 µL, 0.30 mmol, 3.0 equiv) was added and the reaction mixture was stirred for 20 h and allowed to warm to room temperature. The reaction mixture was diluted with EtOAc (30 mL), washed with HCl (1 M, 20 mL), $NaHCO_3$ (satd., 20 mL), brine (20 mL), dried over $MgSO_4$, filtered and concentrated *in vacuo*. The crude product was purified by silica gel chromatography (3% EtOAc in pentane, isocratic) to obtain compound **18** ($\mathbf{18_A}$: $\mathbf{18_B}$, ~ **1** : **1**, 42.3 mg, 31.0 µmol, 31%) as a yellow oil: R_f = 0.7 (10% EtOAc in pentane); ^{1}H NMR (500 MHz, $CDCl_3$) δ 5.86 – 5.68 (m, 1H, $\mathbf{18_A}$ + $\mathbf{18_B}$), 5.55 – 5.46 (m, 1H, $\mathbf{18_A}$ + $\mathbf{18_B}$), 5.42 (d, *J* = 8.2 Hz, 1NH, $\mathbf{18_A}$), 5.37 (d, *J* = 8.1 Hz, 1NH, $\mathbf{18_B}$), 5.34 – 5.26 (m, 1H, $\mathbf{18_A}$ + $\mathbf{18_B}$), 4.95 – 4.85 (m, 2H, $\mathbf{18_A}$ + $\mathbf{18_B}$), 4.31 (d, *J* = 1.9 Hz, 1H, $\mathbf{18_A}$ + $\mathbf{18_B}$), 4.28 – 4.20 (m, 2H, $\mathbf{18_A}$ + $\mathbf{18_B}$), 4.18 – 4.12 (m, 2H, $\mathbf{18_A}$ + $\mathbf{18_B}$), 3.80 – 3.71 (m, 2H, $\mathbf{18_A}$ + $\mathbf{18_B}$), 3.70 – 3.61 (m, 3H, $\mathbf{18_A}$ + $\mathbf{18_B}$), 2.51 – 2.41 (m, 1H, $\mathbf{18_A}$ + $\mathbf{18_B}$), 2.38 (t, *J* = 7.4 Hz, 2H, $\mathbf{18_A}$ + $\mathbf{18_B}$), 2.36 – 2.26 (m, 3H, $\mathbf{18_A}$ + $\mathbf{18_B}$), 2.08 – 1.92 (m, 3H, $\mathbf{18_A}$ + $\mathbf{18_B}$), 1.90 – 1.81 (m, 1H, $\mathbf{18_A}$ + $\mathbf{18_B}$), 1.73 – 1.20 (m, 73H, $\mathbf{18_A}$ + $\mathbf{18_B}$), 1.05 – 1.01 (m, 18H, $\mathbf{18_A}$ + $\mathbf{18_B}$), 0.95 – 0.93 (m, 9H, $\mathbf{18_A}$ + $\mathbf{18_B}$), 0.93 – 0.91 (m, 9H, $\mathbf{18_A}$ + $\mathbf{18_B}$), 0.88 (t, *J* = 7.0 Hz, 6H, $\mathbf{18_A}$ + $\mathbf{18_B}$), 0.83 – 0.73 (m, 1H, $\mathbf{18_A}$ + $\mathbf{18_B}$), 0.14 (s, 3H, $\mathbf{18_A}$ + $\mathbf{18_B}$), 0.12 (s, 3H, $\mathbf{18_A}$ + $\mathbf{18_B}$), 0.11 (s, 3H, $\mathbf{18_A}$), 0.11 (s, 3H, $\mathbf{18_B}$), 0.10 (s, 3H, $\mathbf{18_A}$), 0.10 (s, 3H, $\mathbf{18_B}$); 177.4 (x2),* 174.1 (x2), 155.5 (x2), 155.2 (x2),* 132.1 (x2), 131.9 (x2),* 131.6 (x2),* 131.3 (x2), 101.3 (x2), 78.0, 78.0, 75.0 (x2),* 74.8 (x2), 74.3, 74.2, 73.9*, 74.8,* 71.5 (x2), 70.9, 70.8, 69.5 (x2), 69.2 (x2)* 68.3 (x2), 67.5, 67.2 (x3), 51.6, 51.4, 43.0 (x2), 40.8 (x2), 36.0 (x4), 34.6 (x2), 34.5, 34.3, 34.3, 33.8, 32.1 (x2), 29.9 (x 50), 29.7 (x2), 29.6 (x2), 29.5 (x2), 29.5 (x2), 29.4 (x2), 29.3 (x2), 29.2, 29.2, 27.6 (x6), 27.5 (x6), 26.3 (x6), 26.2 (x6), 25.1 (x3), 24.9, 24.2, 24.0, 23.6, 22.8 (x2), 20.8, 18.5, 18.2, 14.3 (x4), -3.9 (x2), -4.0 (x2), -4.3 (x2), -4.6 (x2).

*Note: Additional ^{13}C signals encountered which indicate the presence of an additional regioisomer are denoted.**

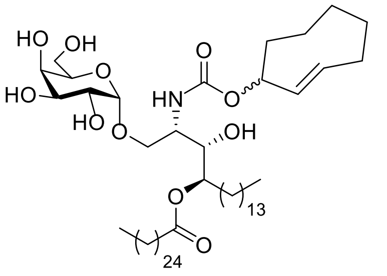

TCO caged αGalCer produg (1): Compound **18** (41.0 mg, 30.0 µmol, 1.0 equiv) was dissolved in anhydrous THF (300 µL) under N_2. The solution was cooled to 0°C before adding Et_3N · 3HF (48 µL, 297 µmol, 10.0 equiv). The reaction mixture was stirred for 27 h and allowed to warm to room temperature. The reaction mixture was concentrated *in vacuo*, redissolved in distilled EtOAc (20 mL), washed with H_2O (2 x 10 mL), brine (10 mL), dried over $MgSO_4$, filtered and concentrated *in vacuo*. The crude product was purified by silica gel chromatography (100% distilled EtOAc, isocratic) to obtain caged αGalCer **1** ($\mathbf{1_A}$: $\mathbf{1_B}$, ~ **1** : **1**, 7.0 mg, 6.93 µmol, 23%) as a crystalline solid: R_f = 0.2 (100% EtOAc); ^{1}H NMR (600 MHz, Dioxane-d_8) δ 6.27 (d, *J* = 9.3 Hz, 1NH)*, 6.04 (d, *J* = 9.1 Hz, 1NH, $\mathbf{1_A}$), 6.01 (d, *J* = 8.8 Hz, 1NH, $\mathbf{1_B}$), 5.85 – 5.66 (m, 1H, $\mathbf{1_A}$ + $\mathbf{1_B}$), 5.52 (d, *J* = 16.4 Hz, 1H, $\mathbf{1_A}$ + $\mathbf{1_B}$), 5.24 (d, *J* = 13.7 Hz, 1H, $\mathbf{1_A}$ + $\mathbf{1_B}$), 4.99 – 4.85 (m, 1H, $\mathbf{1_A}$ + $\mathbf{1_B}$), 4.81 – 4.68 (m, 1H, $\mathbf{1_A}$ + $\mathbf{1_B}$), 4.21 – 4.09 (m, 1H, $\mathbf{1_A}$ + $\mathbf{1_B}$), 3.98 – 3.38 (m, 9H, $\mathbf{1_A}$ + $\mathbf{1_B}$), 2.51 – 2.39 (m, 1H, $\mathbf{1_A}$ + $\mathbf{1_B}$), 2.35 – 2.26 (m, 2H, $\mathbf{1_A}$ + $\mathbf{1_B}$), 2.22 (t, *J* = 7.4 Hz, 1H)*, 2.09 – 1.90 (m, 3H, $\mathbf{1_A}$ + $\mathbf{1_B}$), 1.88 – 1.77 (m, 1H, $\mathbf{1_A}$ + $\mathbf{1_B}$), 1.73 – 1.13 (m, 75H, $\mathbf{1_A}$ + $\mathbf{1_B}$), 1.12 – 0.99 (m, 1H, $\mathbf{1_A}$ + $\mathbf{1_B}$), 0.88 (t, *J* = 6.9 Hz, 6H, $\mathbf{1_A}$ + $\mathbf{1_B}$); ^{13}C

NMR (151 MHz, Dioxane-d_8) δ 174.8 (x2),* 173.8 (x2), 156.0 (x2), 133.3,* 133.2, 133.1, 133.1,* 131.8, 131.5, 101.5,* 101.3,* 100.5, 100.4, 77.0 (x2), 74.5 (x2), 72.4 (x2), 71.5 (x2), 70.7, 70.6, 70.4 (x2), 70.3 (x2), 67.9,* 67.8,* 67.5, 67.4, 62.6, 62.5, 52.9 (x2),* 52.0 (x2), 42.9,* 41.2 (x2), 36.8 (x2), 36.4 (x2), 34.9, 34.8, 34.0,* 33.9,* 32.7 (x4), 30.4 (x 50), 30.1 (x6), 30.0 (x2), 29.7 (x2), 26.4, 26.1, 25.9, 25.7, 25.6, 25.5, 25.1, 25.1, 24.5,* 24.1,* 23.4 (x4), 14.5 (x4); HRMS: calculated for $C_{59}H_{111}NO_{11}$ 1010.82299 $[M+H]^+$; found 1010.82277. Compound **1** was redissolved in dioxane and lyophilized in small quantities for immunology experiments.

*Note: Additional ^1H and ^{13}C signals encountered which indicate the presence of an additional regioisomer are denoted.**

Peptide Synthesis. Peptide sequences were synthesized using Fmoc Solid Support Chemistry. The C-terminal amino acid was supported using a Wang resin. Elongation of the peptide sequence was accomplished using an automated and repetitive cycle of: *i.* 20% piperidine in NMP (Fmoc deprotection); *ii.* NMP wash; *iii.* Fmoc-protected amino acid (4 equiv), HCTU (4 equiv), DIPEA (8 equiv), NMP; *iv.* NMP wash; *v.* Ac_2O, DIPEA, NMP (capping); *vi.* NMP wash; *vii.* DCM wash. After completing the sequence, the N-terminal amino acid was also deprotected using 20% piperidine in NMP. An analytical amount of crude product was cleaved from the solid support (95% TFA, 2.5% H_2O, 2.5% TIS, > 1.5 h) and precipitated in cold, anhydrous Et_2O (TFA/Et_2O ≈ 1:10 *v/v*, wash resin once with TFA). The Et_2O solution was centrifuged, Et_2O was decanted and the pellet was dissolved in DMSO/MeCN/H_2O/*t*-BuOH (3:1:1:1 *v/v*) for LC-MS analysis.

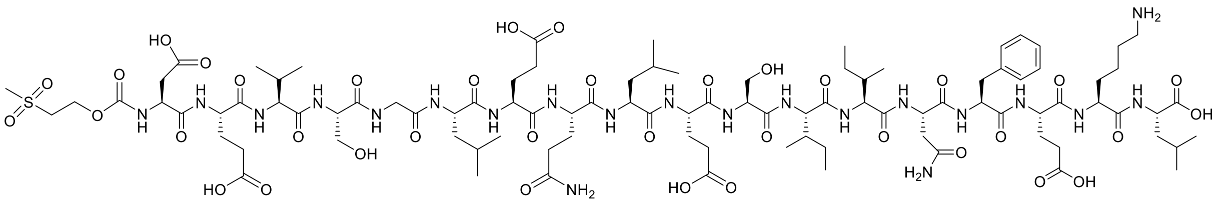

MSc- DEVSGLEQLESIINFEKL (44): After SPPS, using 250 µmol Fmoc-Leu-Wang resin (1.0 equiv, based on theoretical resin loading), resin bound DEVSGLEQLESIINFEKL was washed with DCM (3 x 10 mL) and NMP (3 x 10 mL) before adding a solution of MSc-OSu[1] (**45**, 332 mg, 1.25 mmol, 5.0 equiv) in NMP (6 mL). DIPEA (250 µL, 1 M in NMP, 1.0 equiv) was added and the resin suspension was shaken at room temperature for 4 h. The reaction mixture was drained, MSc-OSu[1] (**45**, 332 mg, 1.25 mmol, 5.0 equiv) in NMP (6 mL) and DIPEA (250 µL, 1 M in NMP, 1.0 equiv) were added and the resin suspension was shaken for an additional 2 h. The reaction mixture was drained and the resin-bound peptide was washed with NMP (3 x 10 ml) and DCM (3 x 10 ml). The crude product was cleaved from the solid support (95% TFA, 2.5% H_2O and 2.5% TIS, ~ 6 mL, > 1.5 h) and precipitated in cold, anhydrous Et_2O (TFA/Et_2O ≈ 1:10 *v/v*, wash resin once with TFA). The Et_2O suspension was centrifuged, Et_2O was decanted and an analytical amount of precipitated product was dissolved in DMSO/MeCN/H_2O/*t*-BuOH (3:1:1:1 *v/v*) for LC-MS analysis. The crude product was purified with HPLC (25 → 50% MeCN in H_2O with 0,1% TFA) to obtain **44** (80 mg, 36 µmol, 14%) as a solid after lyophilization: LC-MS (linear gradient 10 → 90% MeCN, 0.1% TFA, 11 min): R_t (min): 5.66 (ESI-MS (m/z): 1107.47 ($M+2H^+$)).

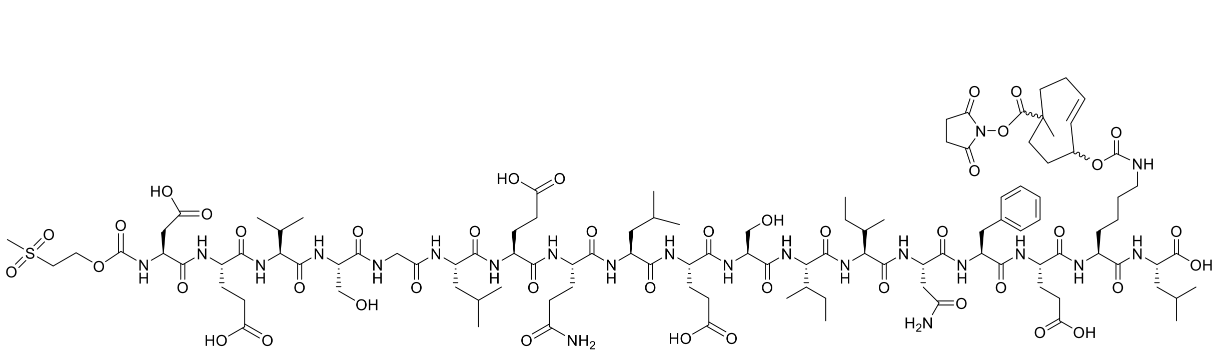

MSc-DEVSGLEQLESIINFEK(NHS-bTCO)L (46): MSc-DEVSGLEQLESIINFEKL (**44**, 20 mg, 9.04 µmol, 1.0 equiv) and bifunctional TCO reagent **32**[2] (8.7 mg, 21 µmol, 2.3 equiv) were combined in an Eppendorf tube (15 mL) and dissolved in DMSO (1.5 mL). Anhydrous DIPEA (15 µL, 86 µmol, 9.5 equiv) was added,

the tube was briefly sonicated and flushed with N_2 before shaking the reaction mixture at room temperature. The Eppendorf tube was shielded with aluminum foil during the reaction. After 20 h, the reaction mixture was added to cold, anhydrous Et_2O (45 ml) to precipitate the product. The Et_2O suspension was briefly centrifuged, Et_2O was decanted and the crude product **46** was dried over a stream of N_2 before using it in the next step without further purification: LC-MS (linear gradient 10 → 90% MeCN, 0.1% TFA, 11 min): R_t (min): 6.56 (ESI-MS (m/z): 1260.67 (M+2H$^+$)).

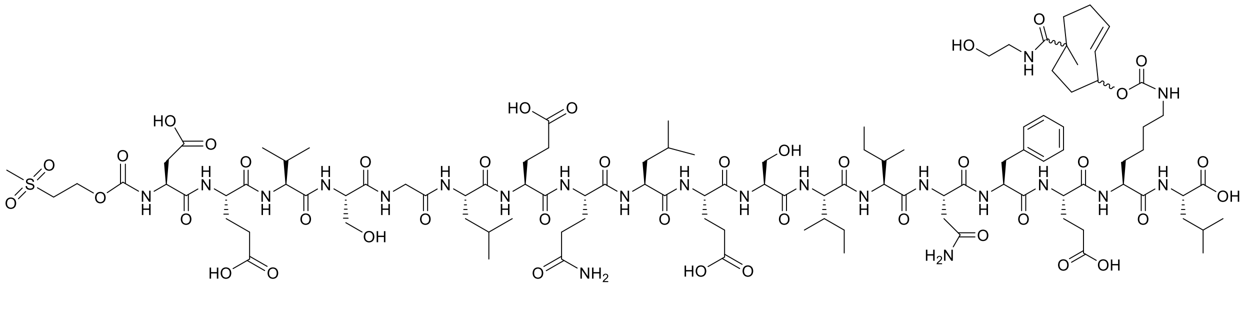

MSc-DEVSGLEQLESIINFEK(mbTCO)L (47): The crude MSc-DEVSGLEQLESIINFEK(NHS-bTCO)L (**46**) preciously described was dissolved in DMSO (1.5 mL). Ethanolamine (5.0 µL, 83 µmol, 9.2 equiv) was added, the tube was briefly sonicated and flushed with N_2 before shaking the reaction mixture at room temperature. The Eppendorf tube was shielded with aluminum foil during the reaction. After 20 h, the reaction mixture was added to cold, anhydrous Et_2O (45 ml) to precipitate the product. The Et_2O suspension was briefly centrifuged, Et_2O was decanted and the crude product **47** was dried over a stream of N_2 before using it in the next step without further purification: LC-MS (linear gradient 10 → 90% MeCN, 0.1% TFA, 11 min): R_t (min): 6.07 (ESI-MS (m/z): 1233.87 (M+2H$^+$)).

Note: synthesis of* ***32* *from* ***10*** *could also be performed without work-up after LC-MS analysis indicated the first step was complete.*

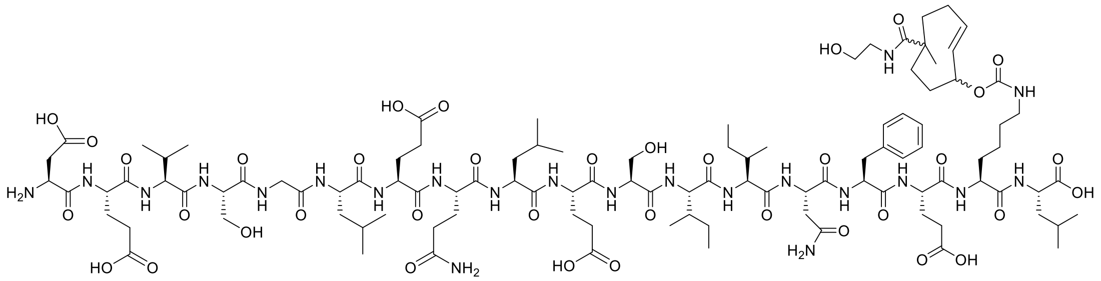

DEVSGLEQLESIINFEK(mbTCO)L (6): The crude MSc-DEVSGLEQLESIINFEK(mbTCO)L (**47**) preciously described was dissolved in dioxane/MeOH/4 M NaOH (7.5:2.25:0.25 *v/v*, 10 mL). The reaction mixture was sonicated and occasionally shaken for 15 min. The reaction mixture was neutralized by adding acetic acid (54 µL, 0.94 mmol) before precipitating the product in cold, anhydrous Et_2O (~ 40 mL). The Et_2O suspension was centrifuged, Et_2O was decanted and the crude product was dried over a stream of N_2. The crude product was then purified with HPLC (25 → 50% MeCN in H_2O with 10 mM NH_4OAc) to obtain mbTCO-OVA18 **6** (8.6 mg, 3.71 µmol, 41% over three steps) as a solid after lyophilization: LC-MS (linear gradient 10 → 90% MeCN, 0.1% TFA, 11 min): R_t (min): 5.53 (ESI-MS (m/z): 1158.73 (M+2H$^+$)); HRMS: calculated for $C_{104}H_{168}N_{22}O_{37}$ 1158.59649 $[M+2H]^{2+}$; found 1158.59632.

Note: during HPLC purification of* ***6**, addition of* NH_4OAc *can increase the quantities of crude product injected per run.*

5. NMR spectra

^{1}H and ^{13}C APT spectra of 26

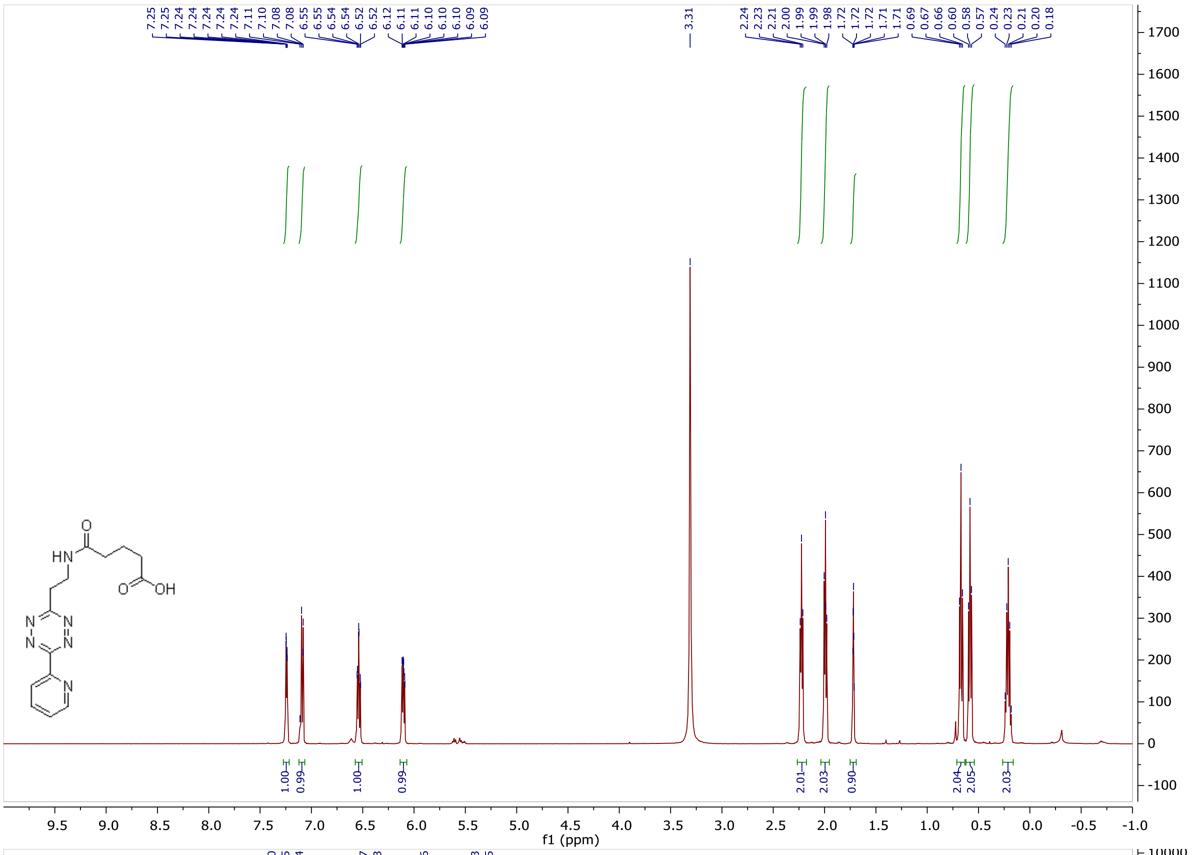

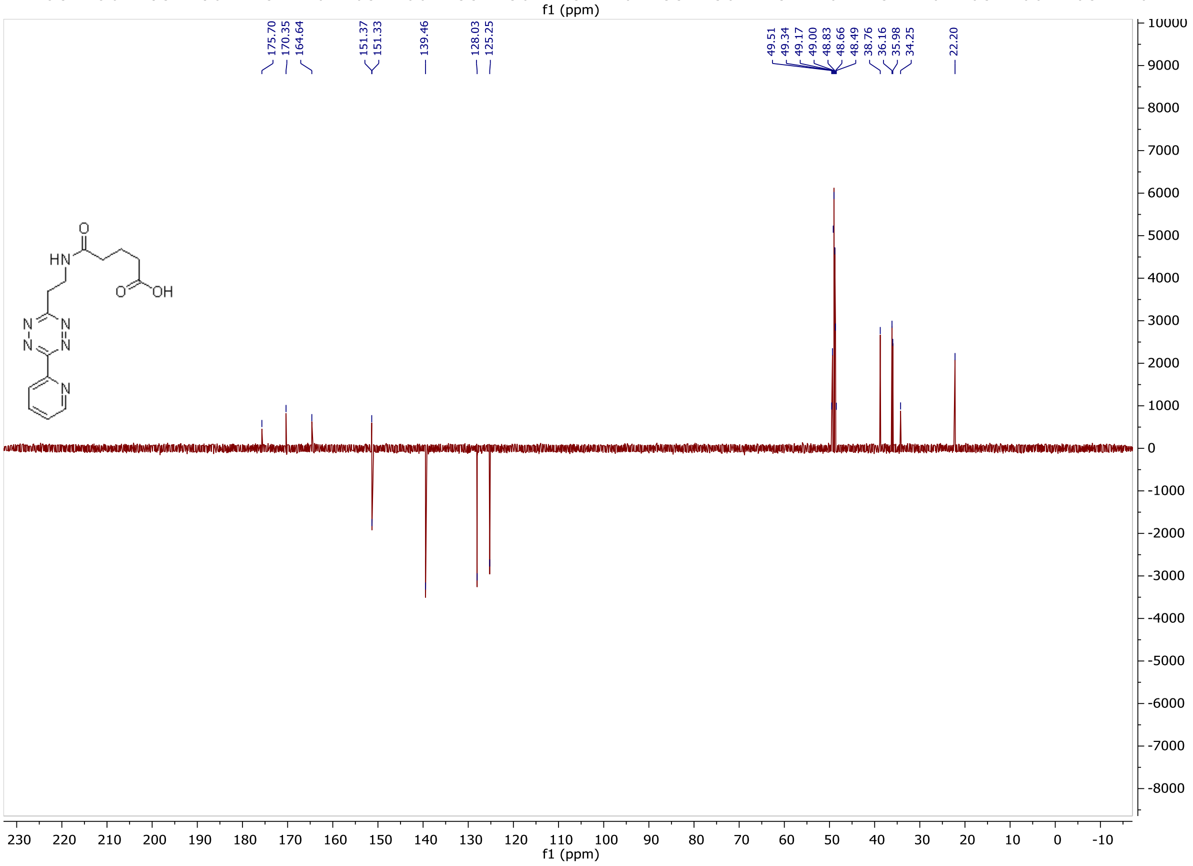

^{1}H, ^{13}C APT, ^{1}H COSY and HSQC spectra of 3

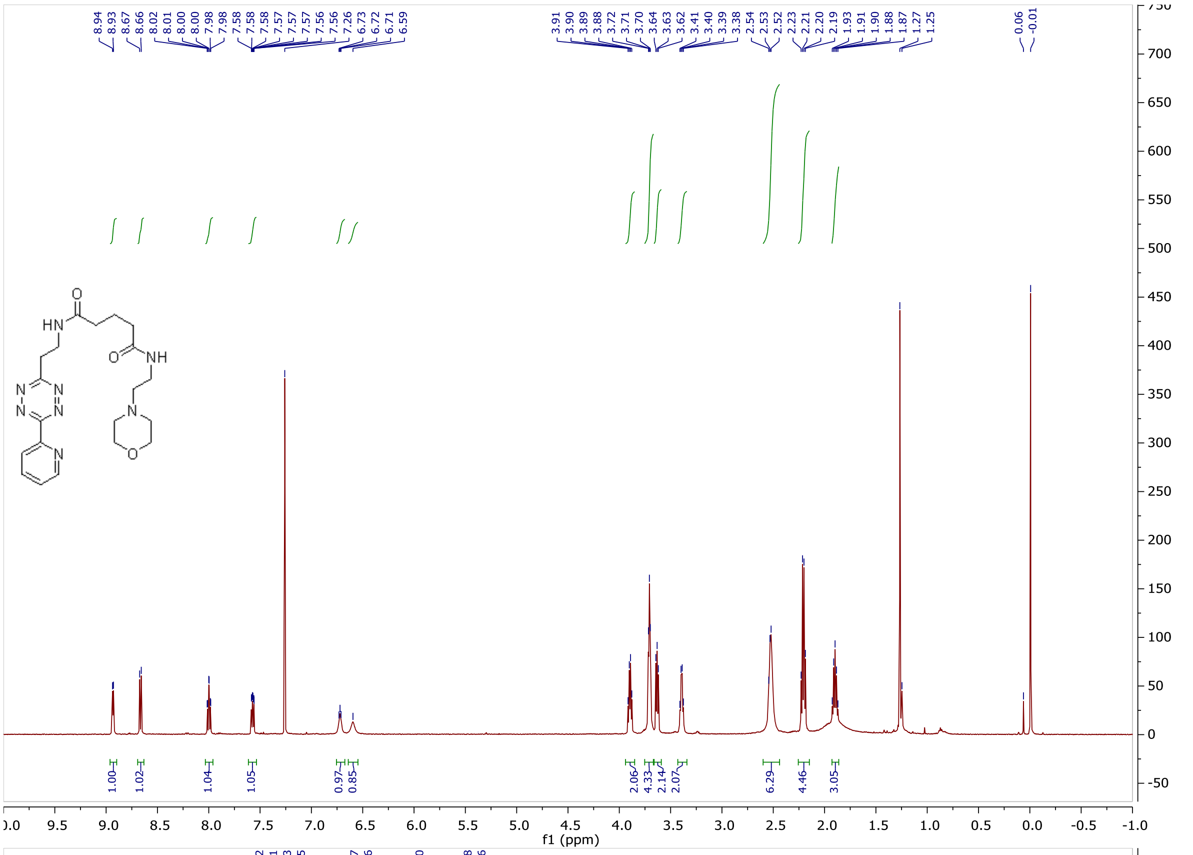

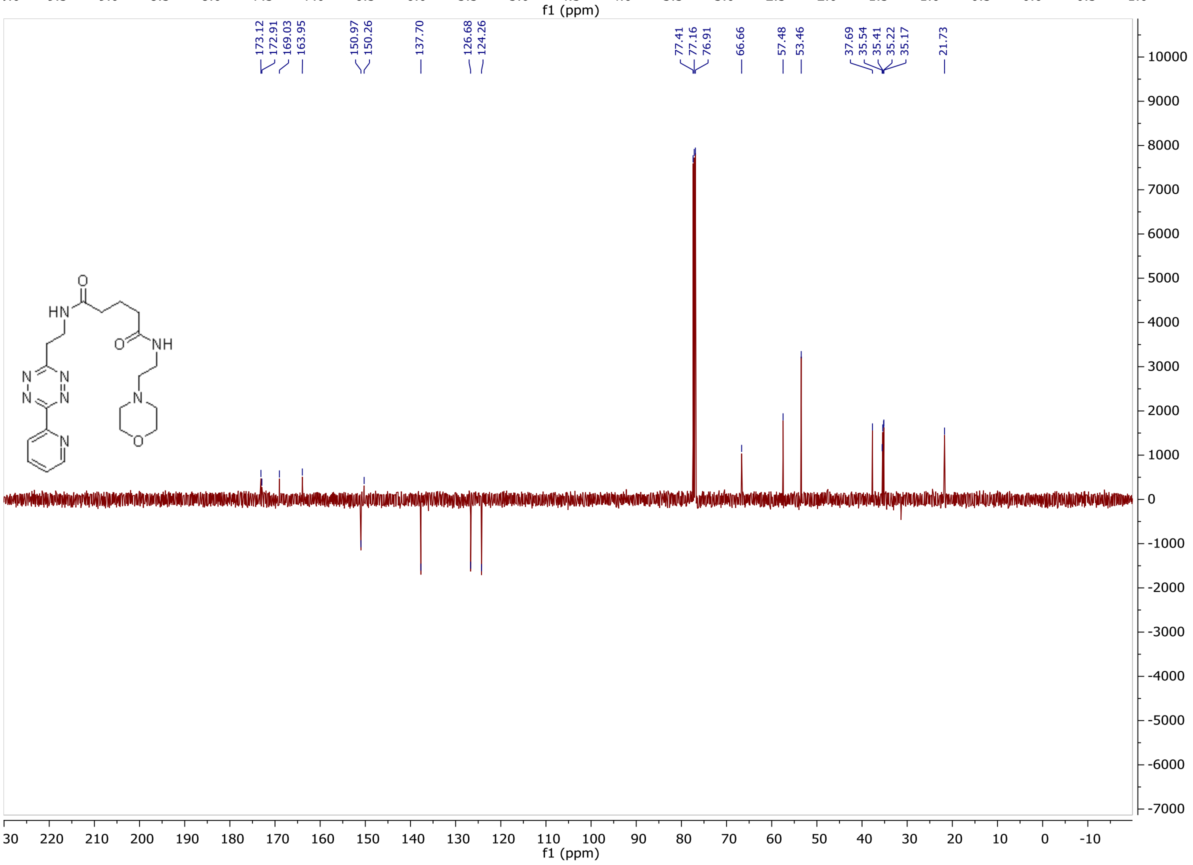

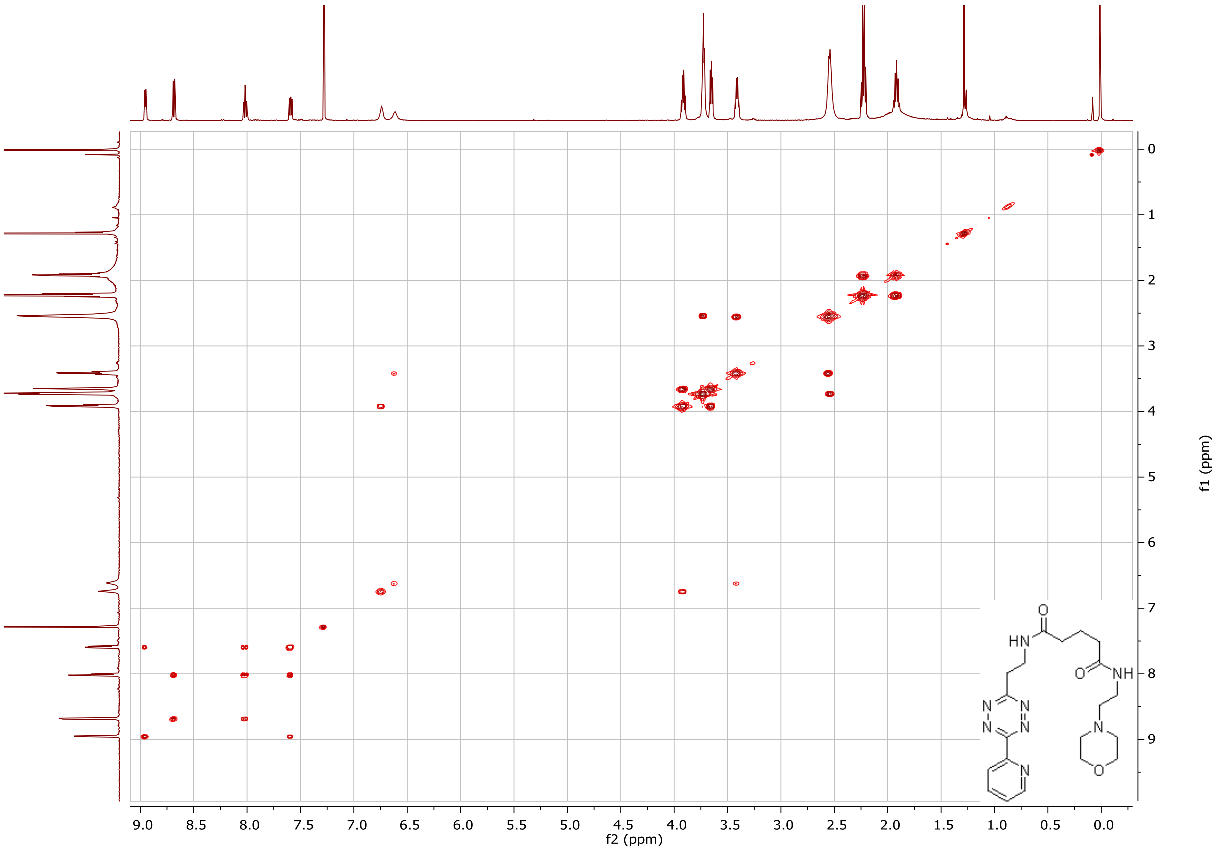
f2 (ppm)
f1 (ppm)
HN
O
NH
N
O

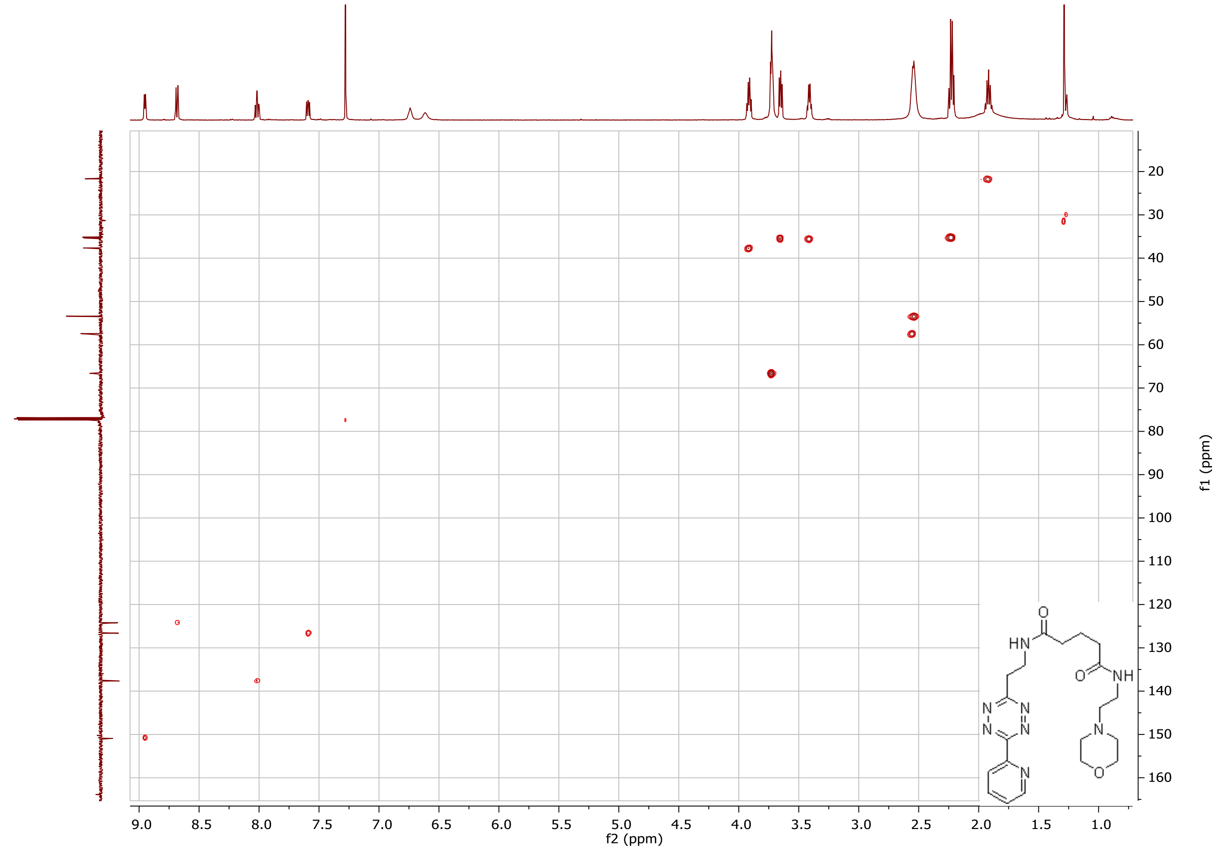
f2 (ppm)
f1 (ppm)
HN
O
NH
N
O

^{1}H spectrum of 50

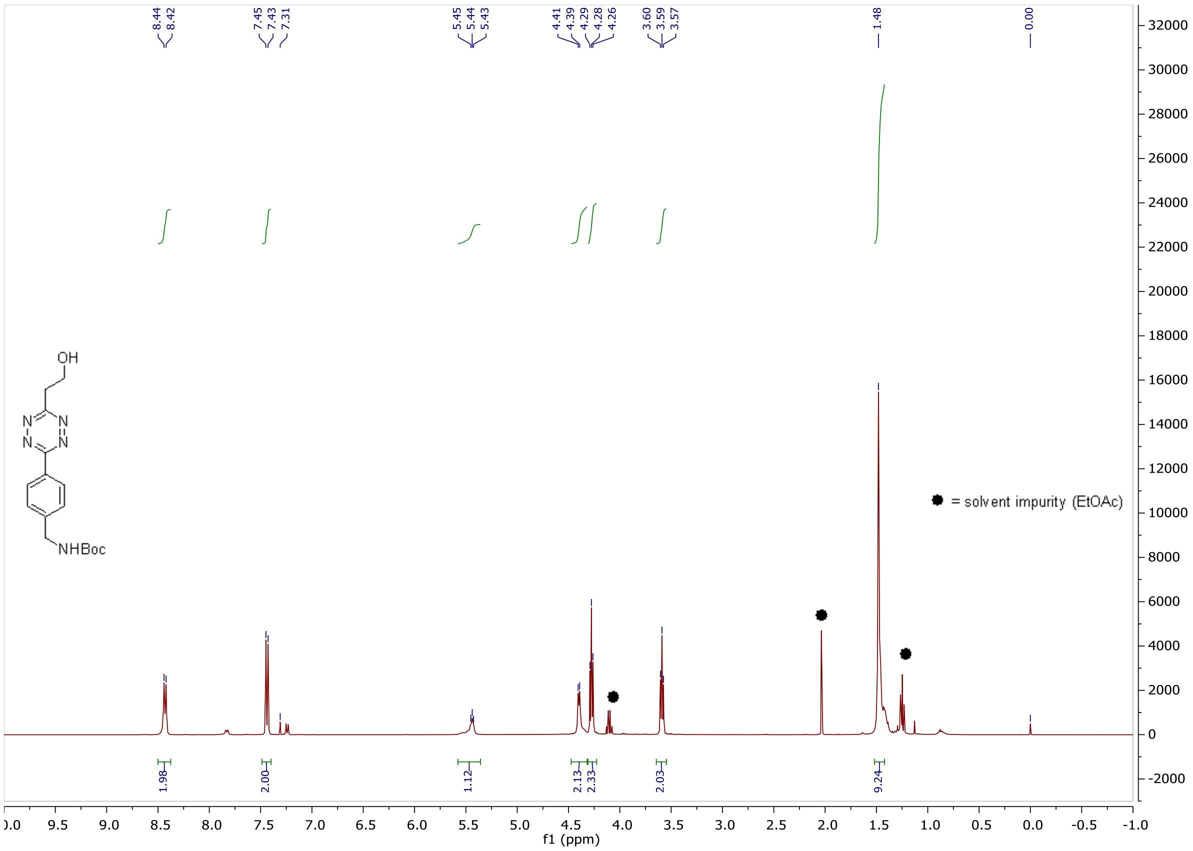

^{1}H and ^{13}C spectrum of 24

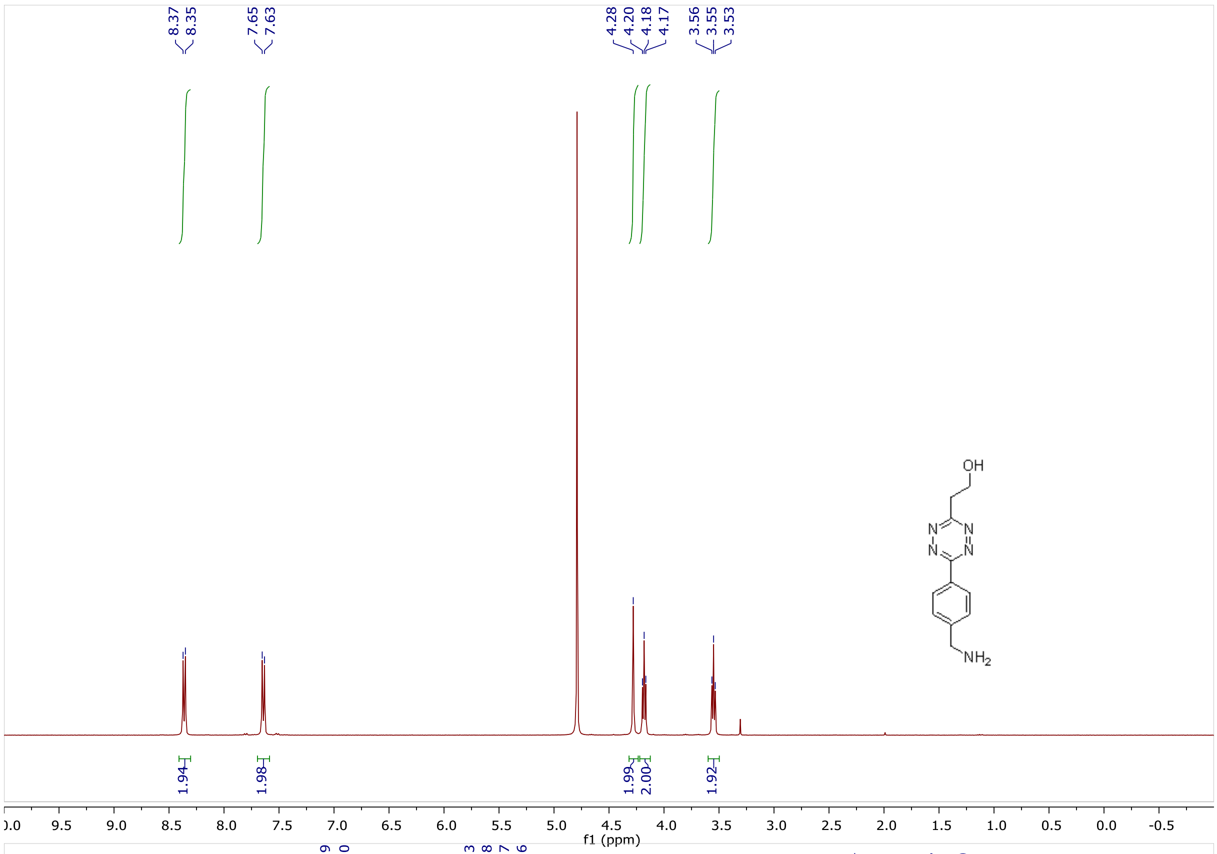

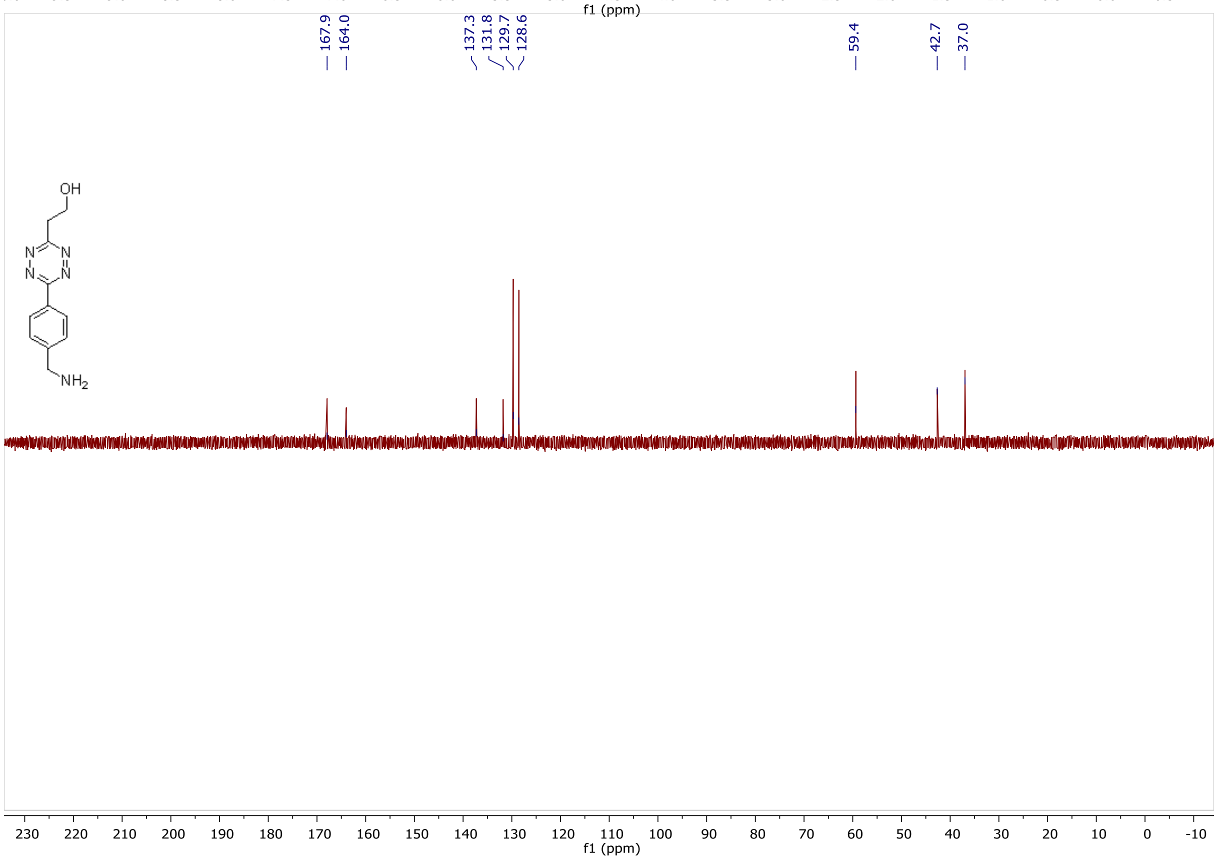

^{1}H spectrum of 28

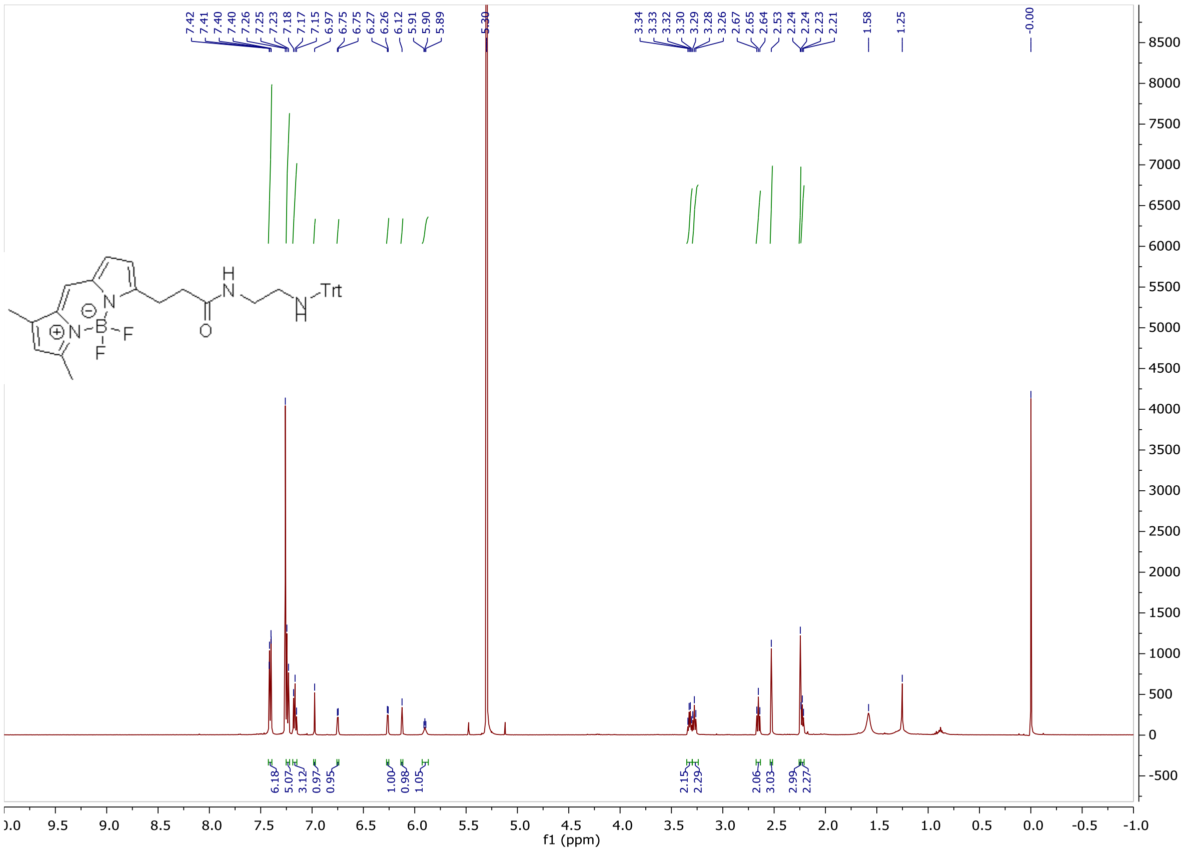

^{1}H, ^{13}C APT, ^{1}H COSY and HSQC spectra of 31

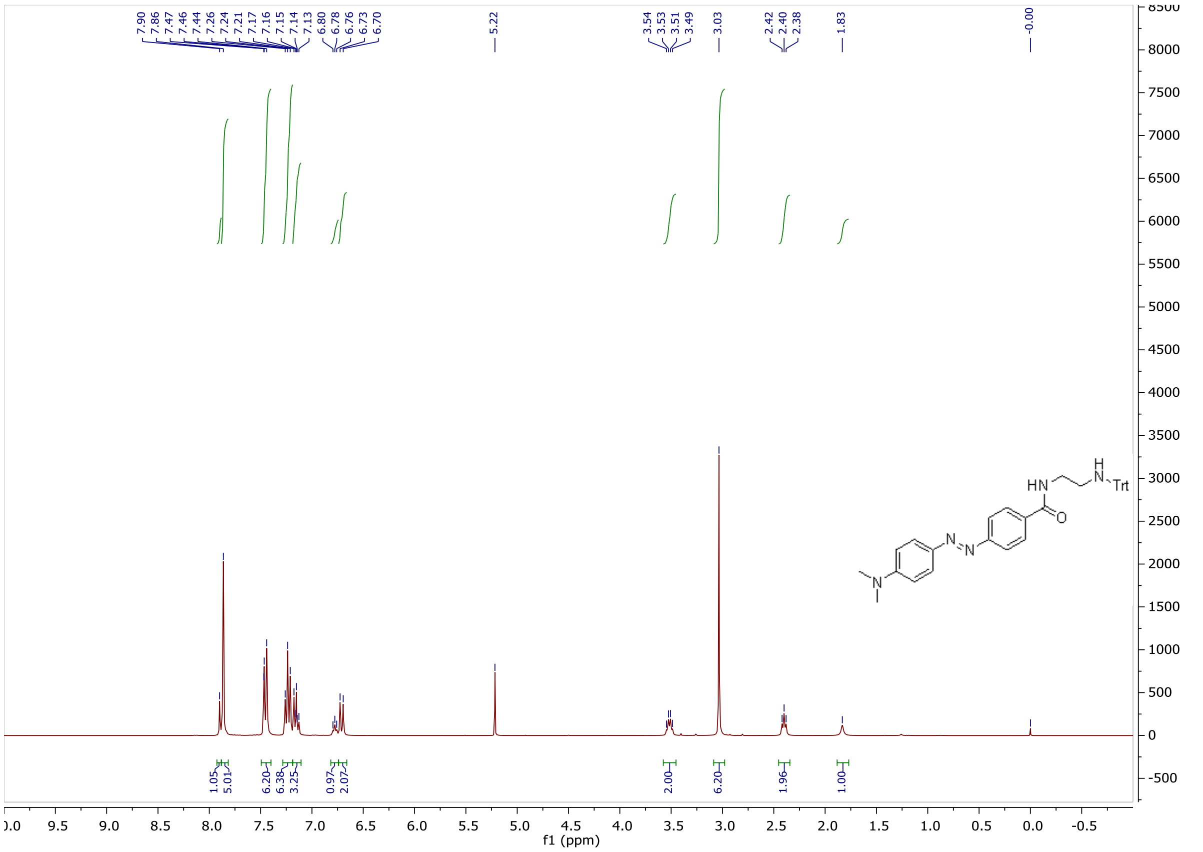

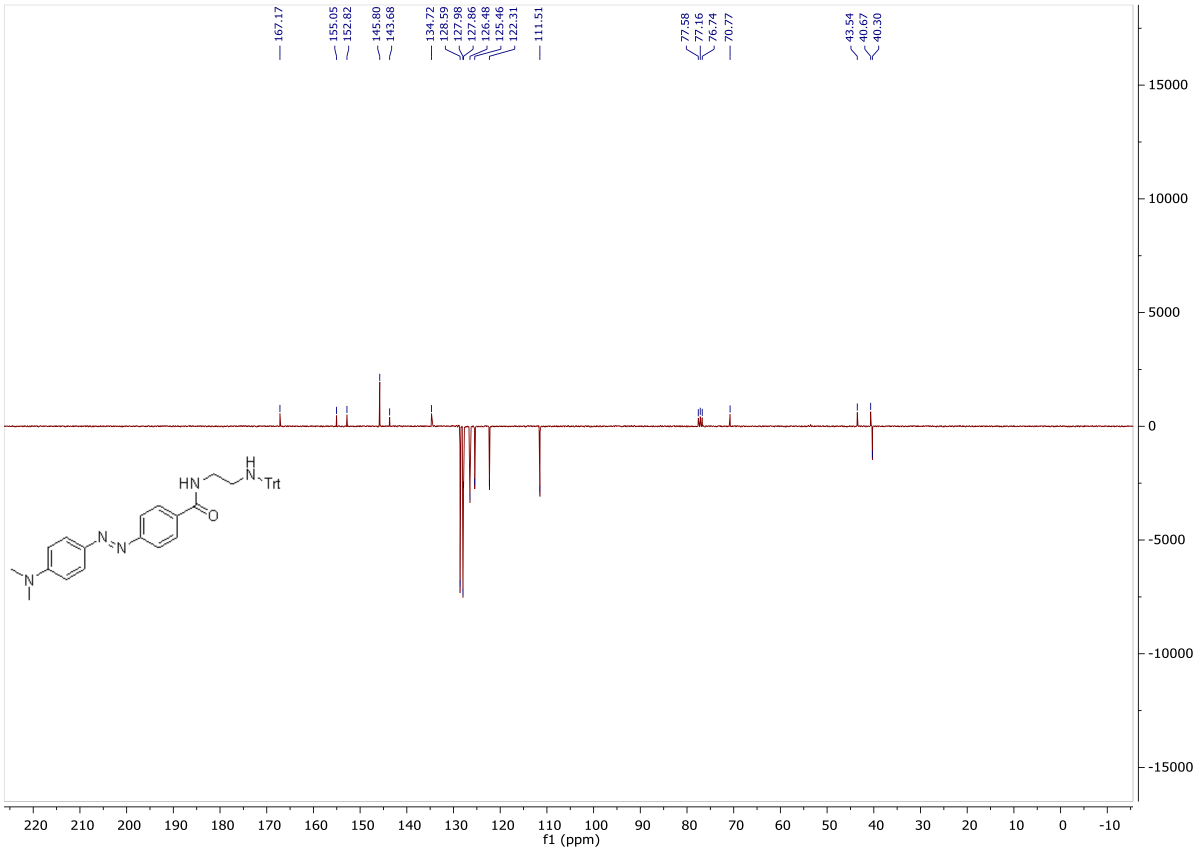

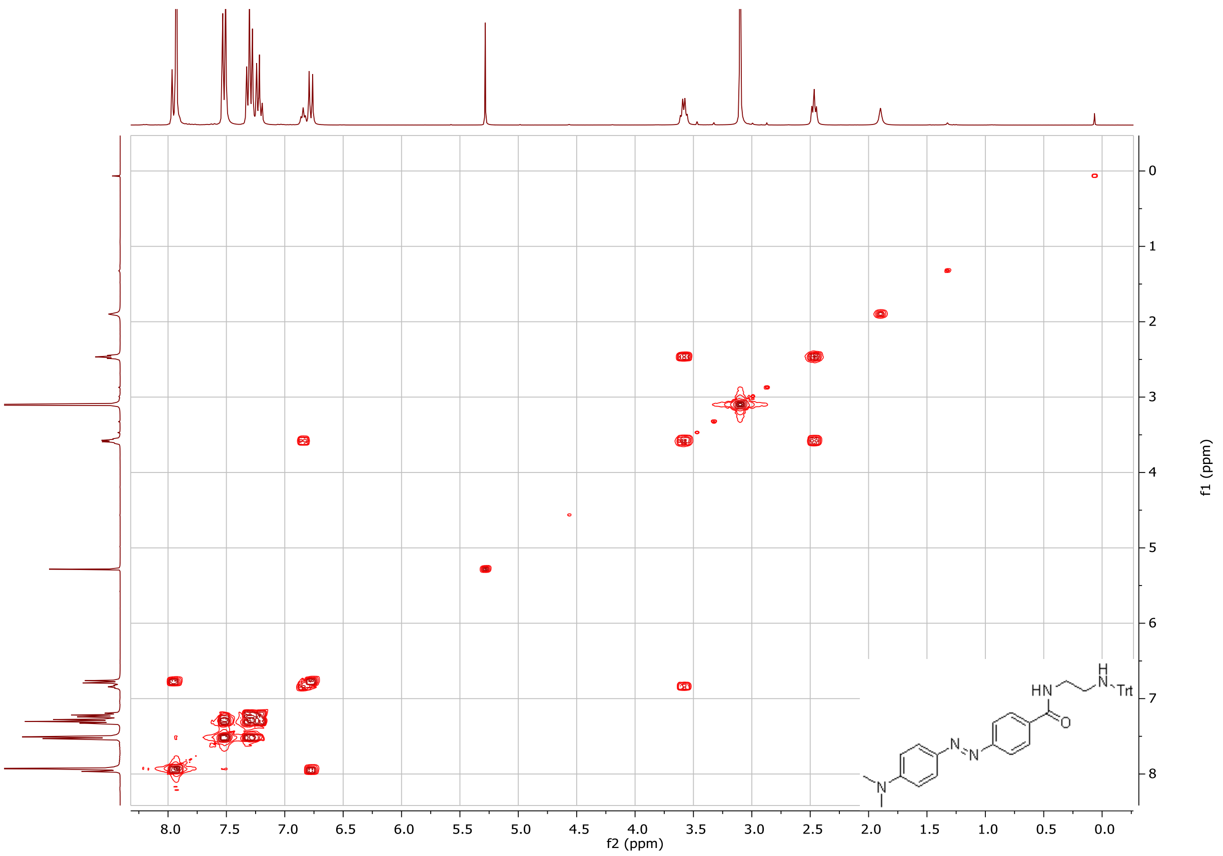
f2 (ppm)
f1 (ppm)
HN
N
Trt
H
N
N
N
O

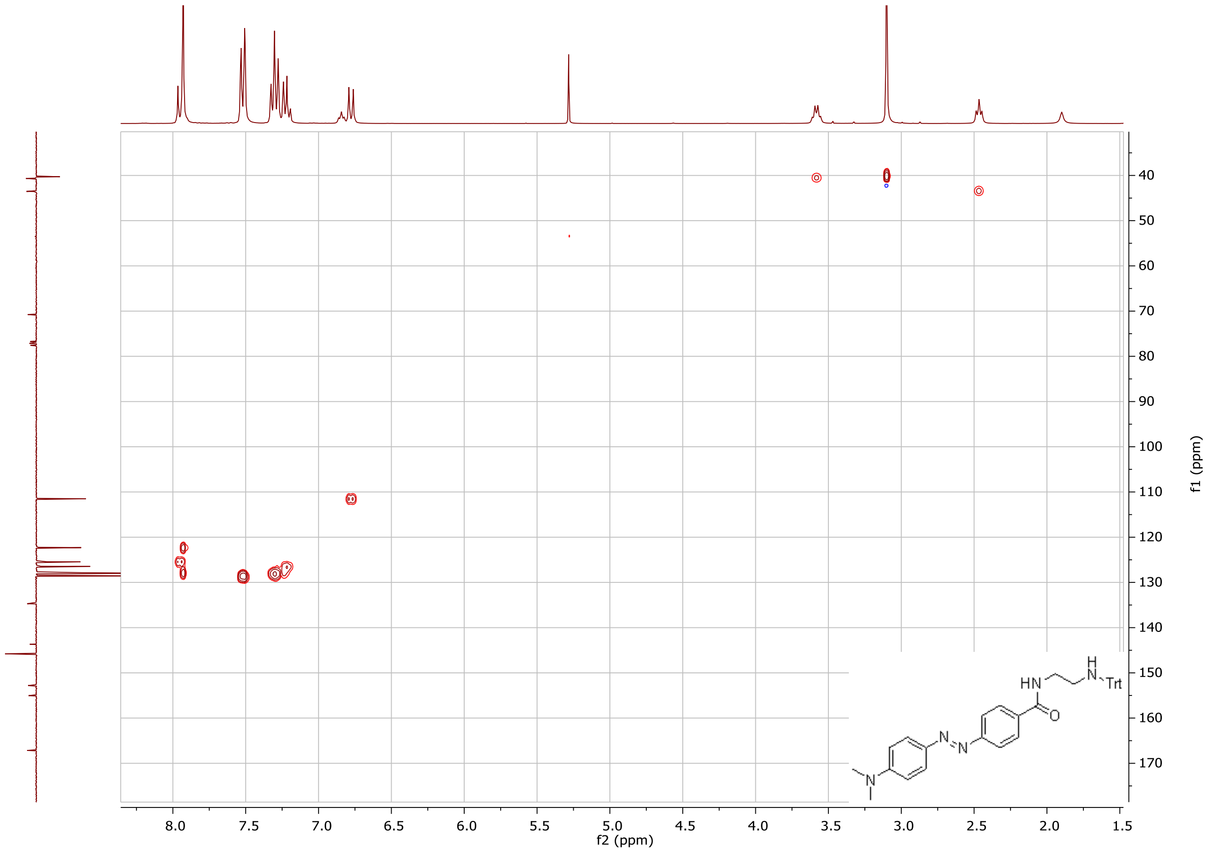
f2 (ppm)
f1 (ppm)
HN
N
Trt
H
N
N
N
O

^{1}H, ^{13}C APT, ^{1}H COSY and HSQC spectra of 36

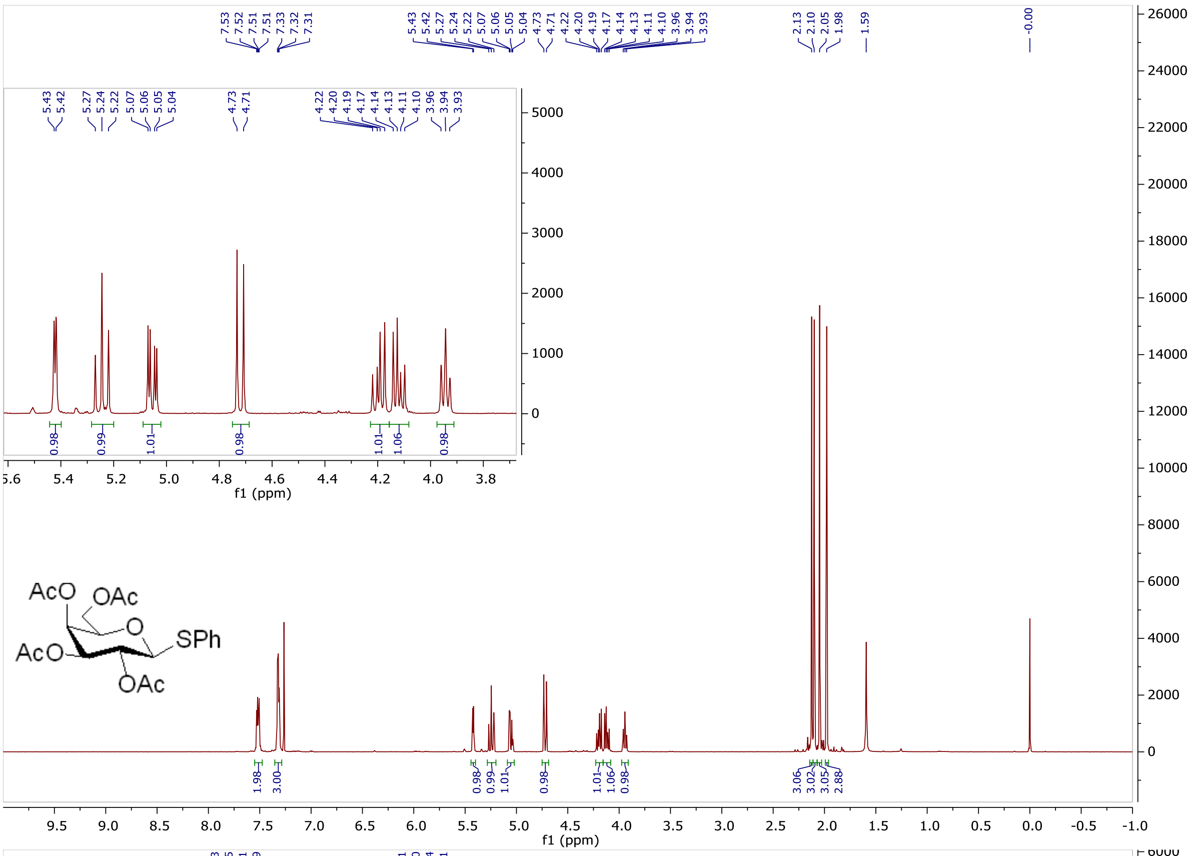

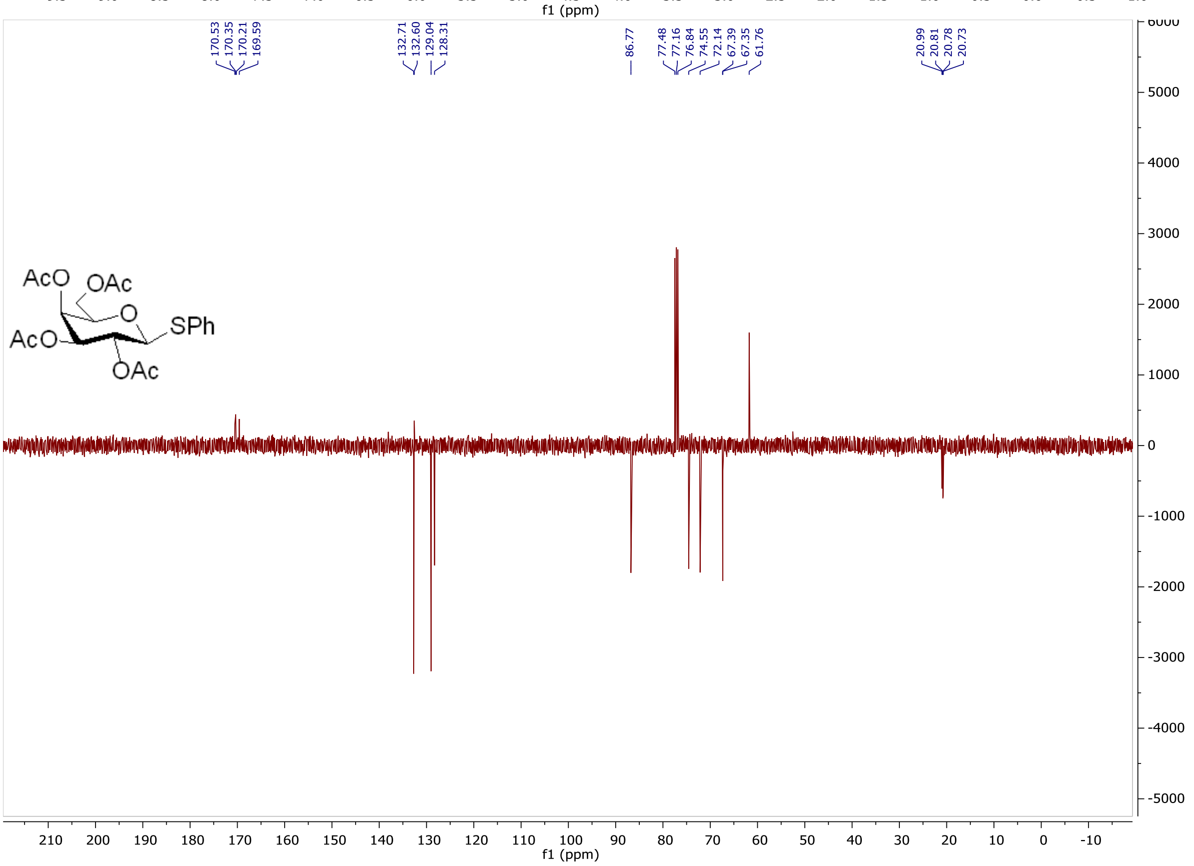

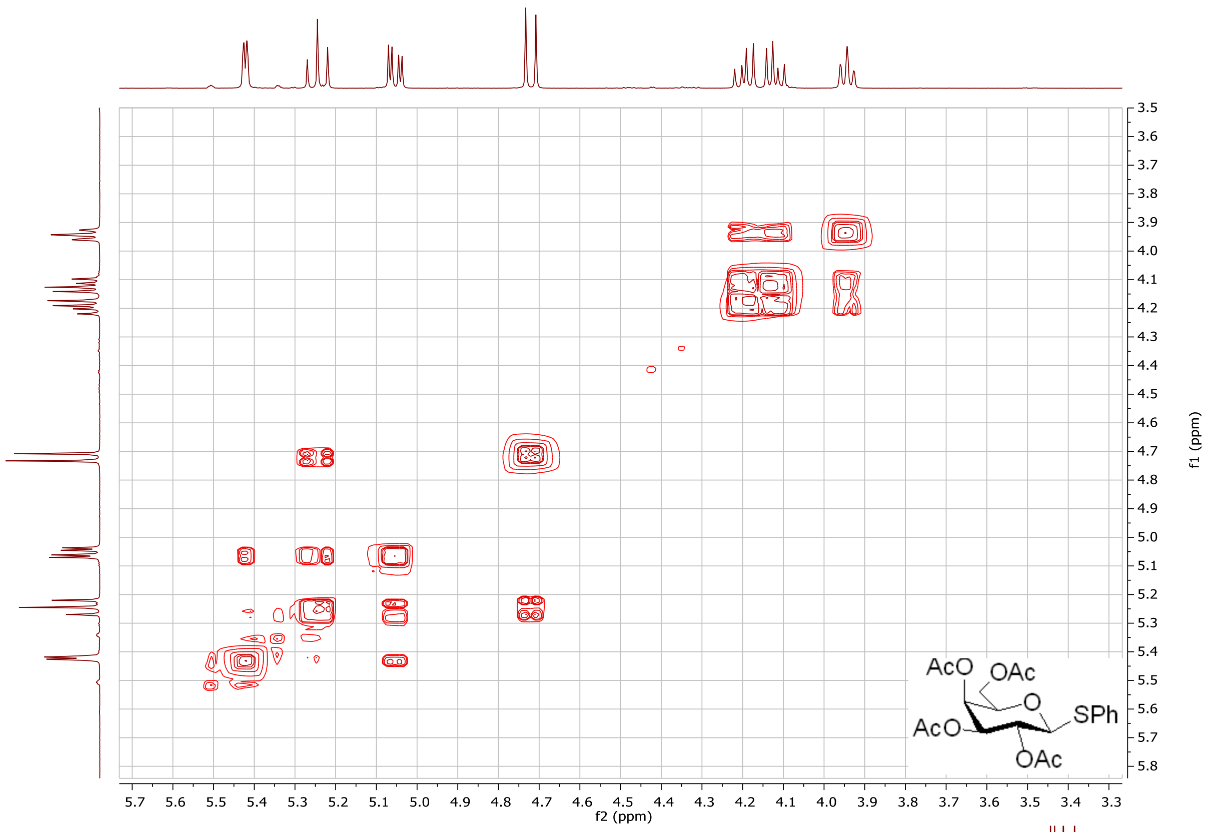
AcO
OAc
O
SPh
AcO
OAc
f2 (ppm)
f1 (ppm)

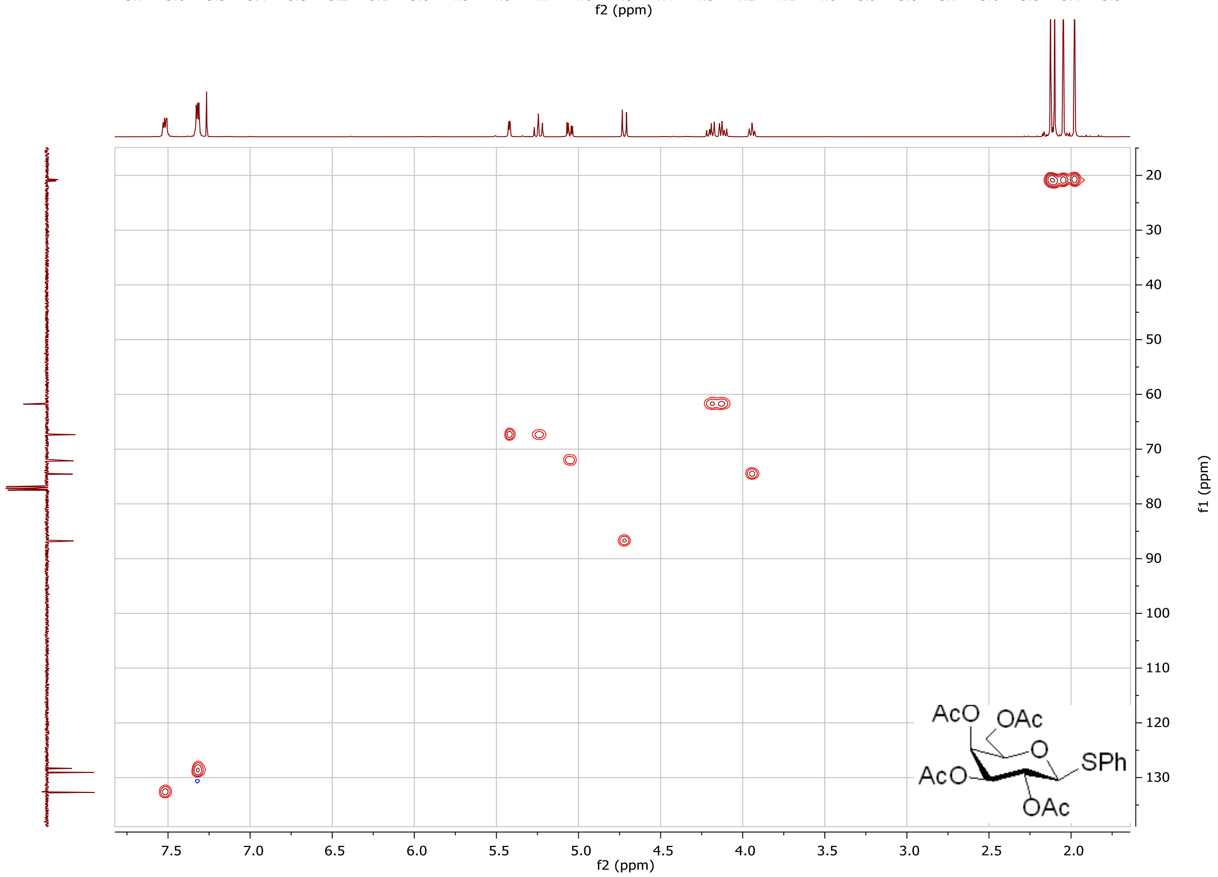
AcO
OAc
O
SPh
AcO
OAc
f2 (ppm)
f1 (ppm)

^{1}H, ^{13}C APT, ^{1}H COSY and HSQC spectra of 37

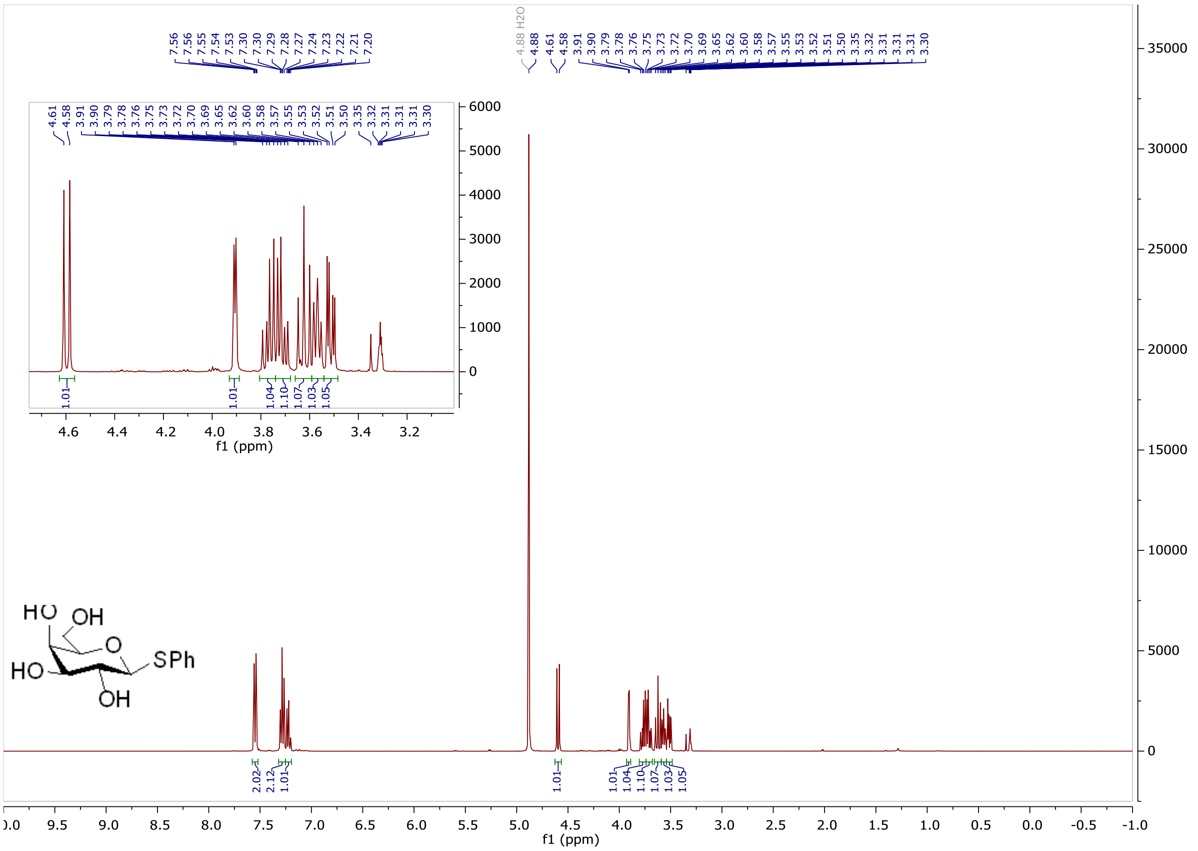

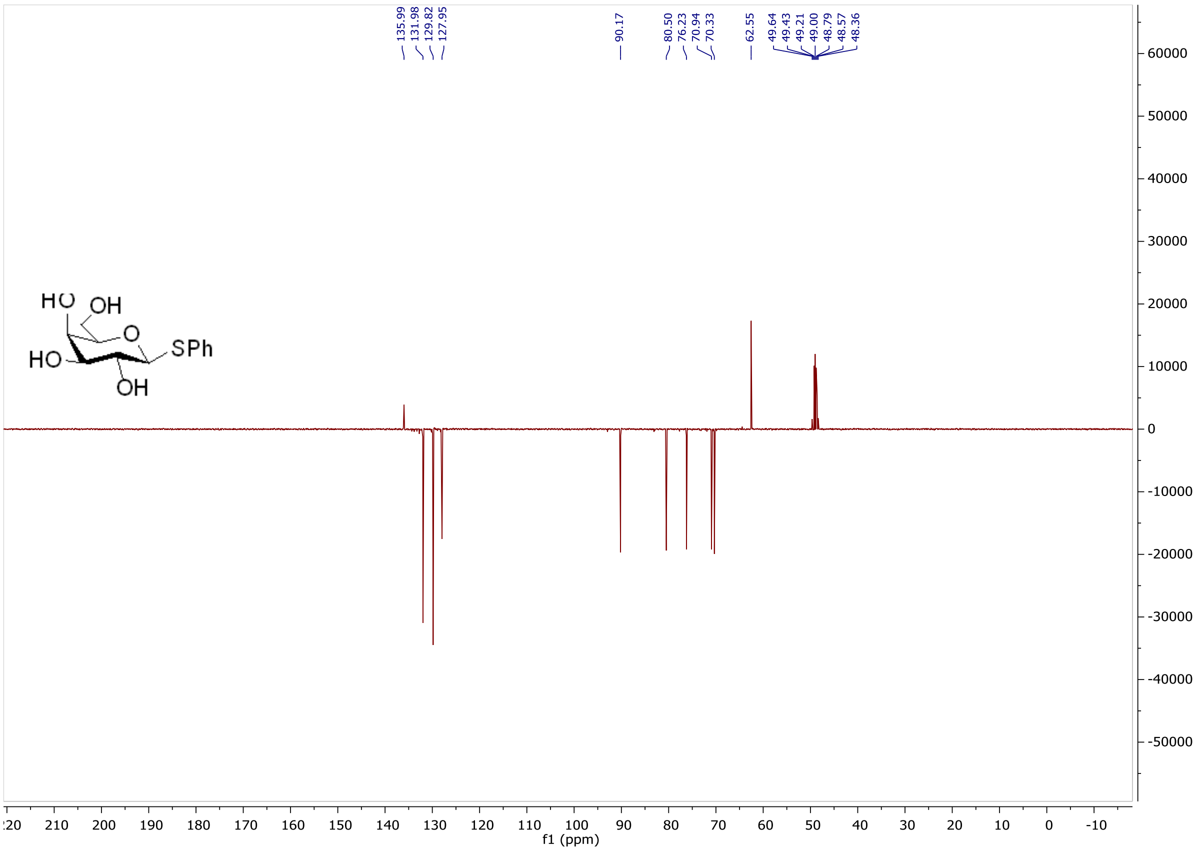

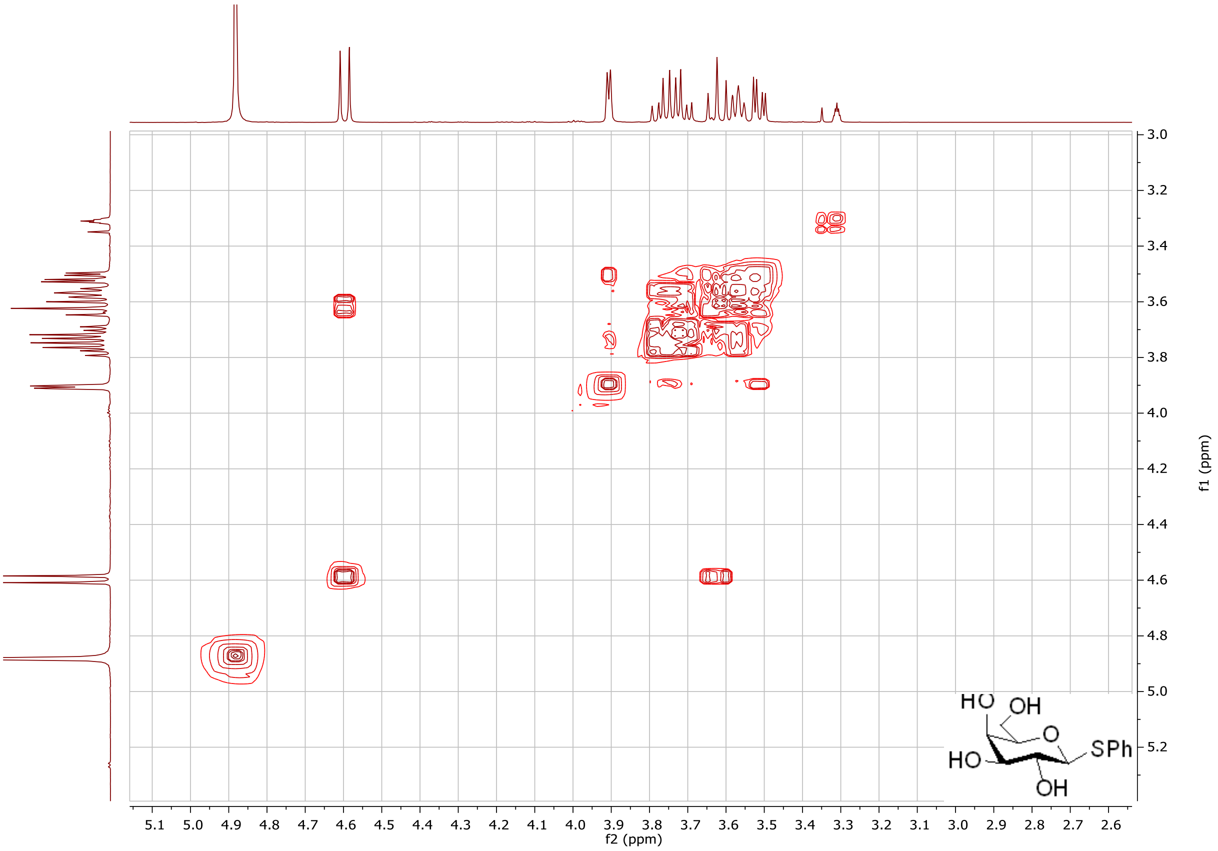
f1 (ppm)
f2 (ppm)
HO OH
O
SPh
HO
OH

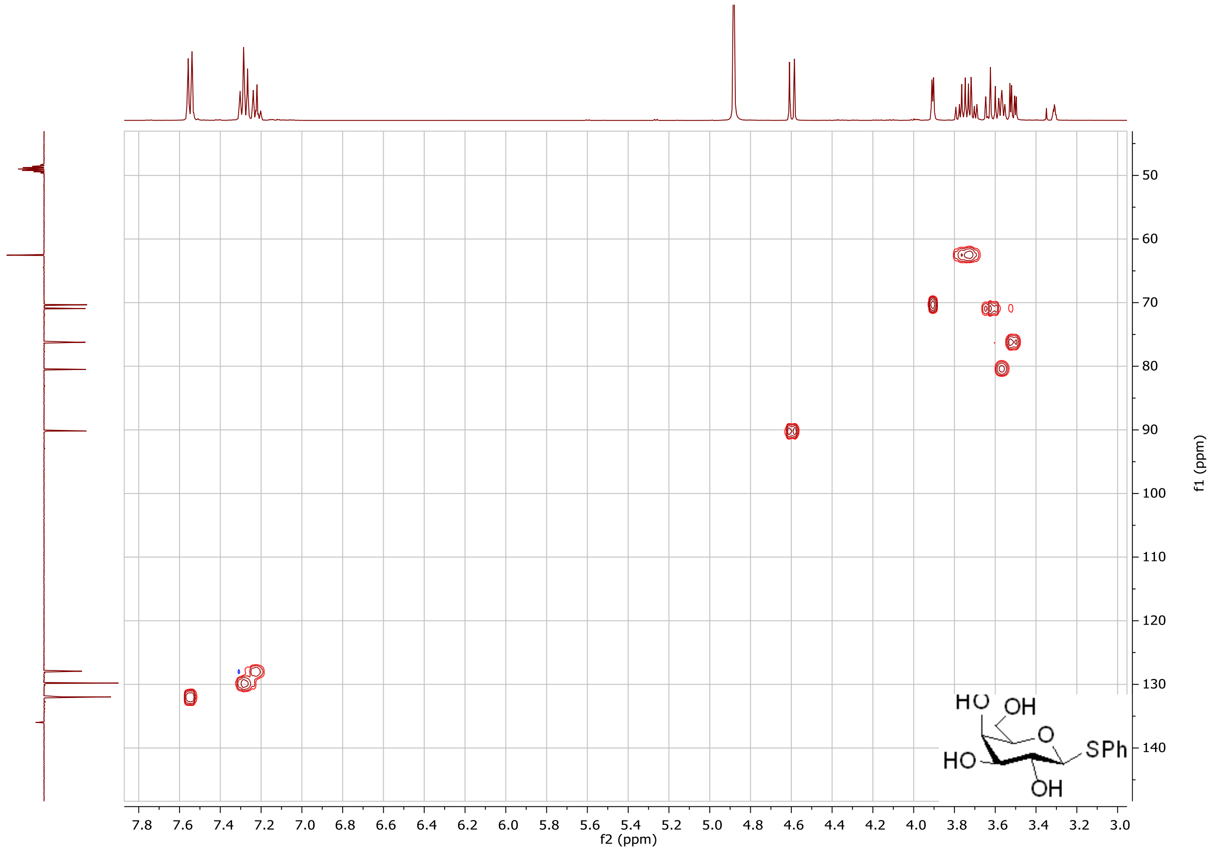
f1 (ppm)
f2 (ppm)
HO OH
O
SPh
HO
OH

^{1}H, ^{13}C APT, ^{1}H COSY and HSQC spectra of 38

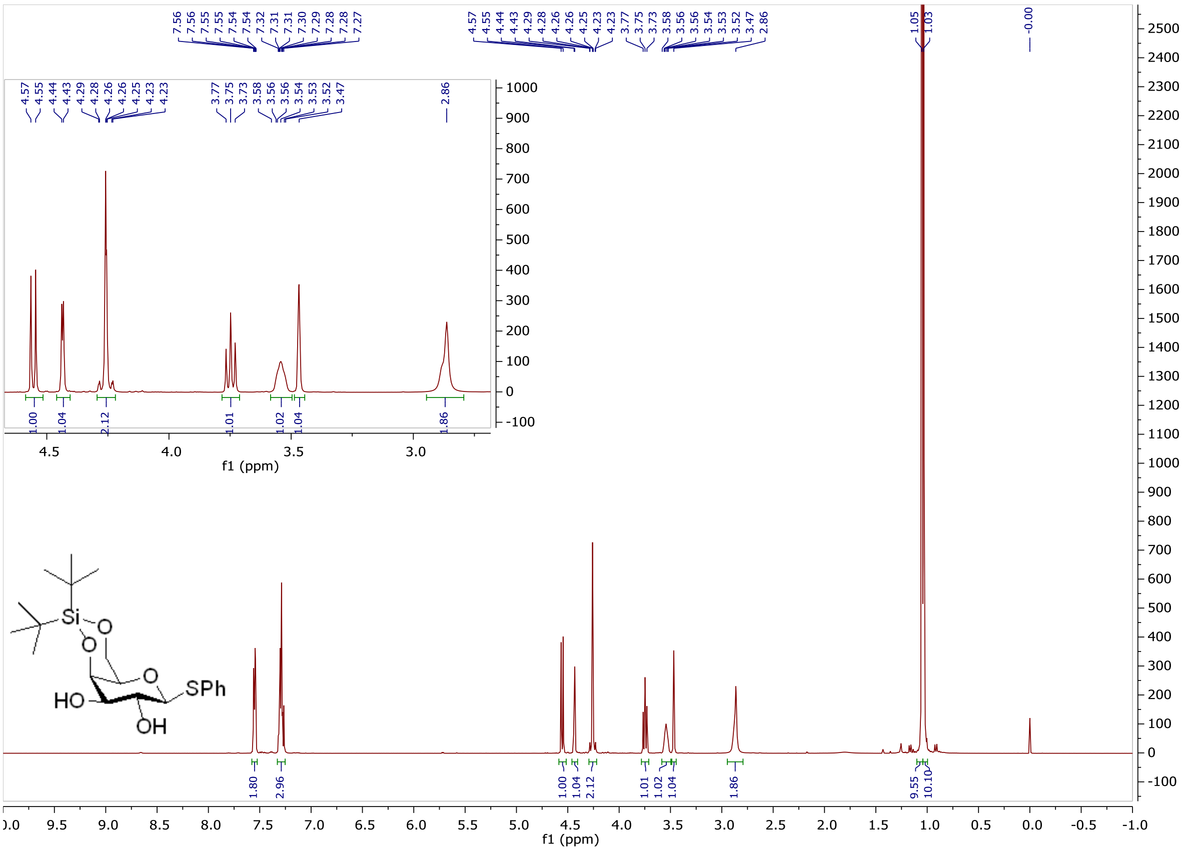

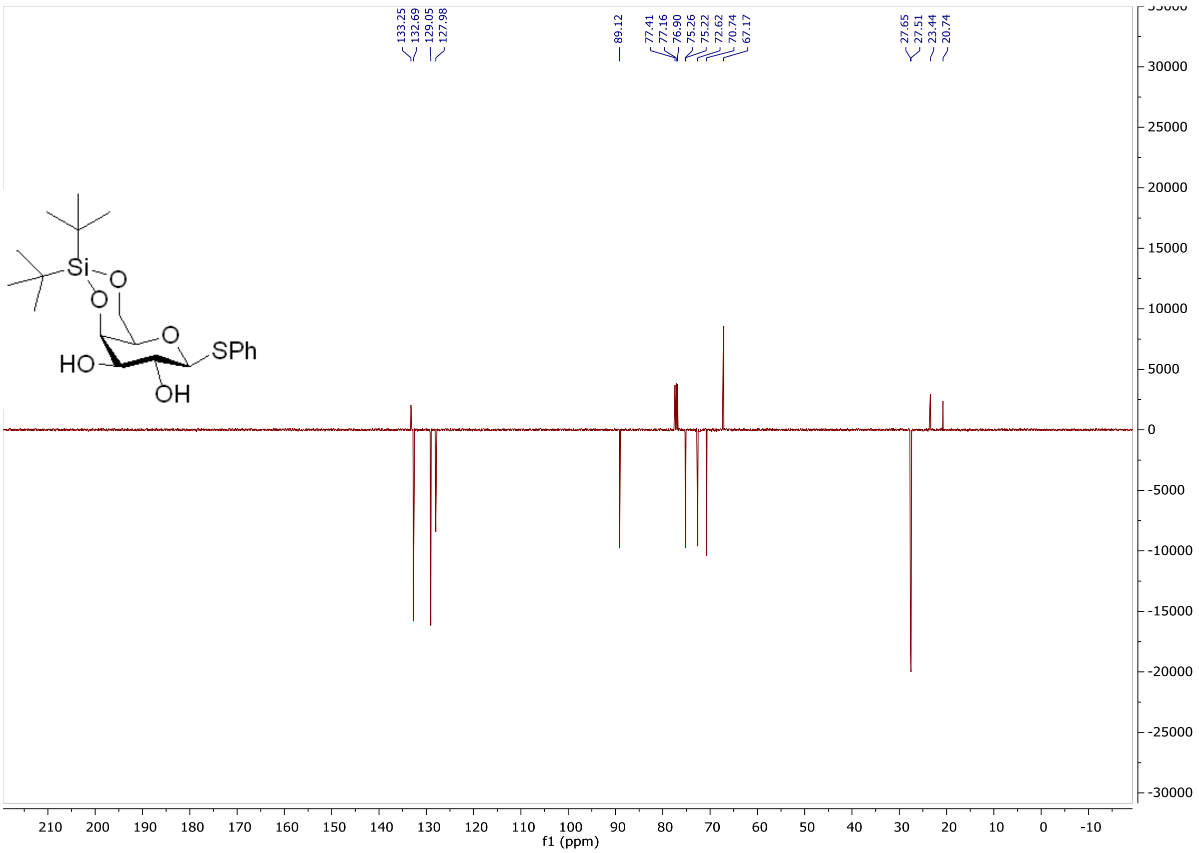

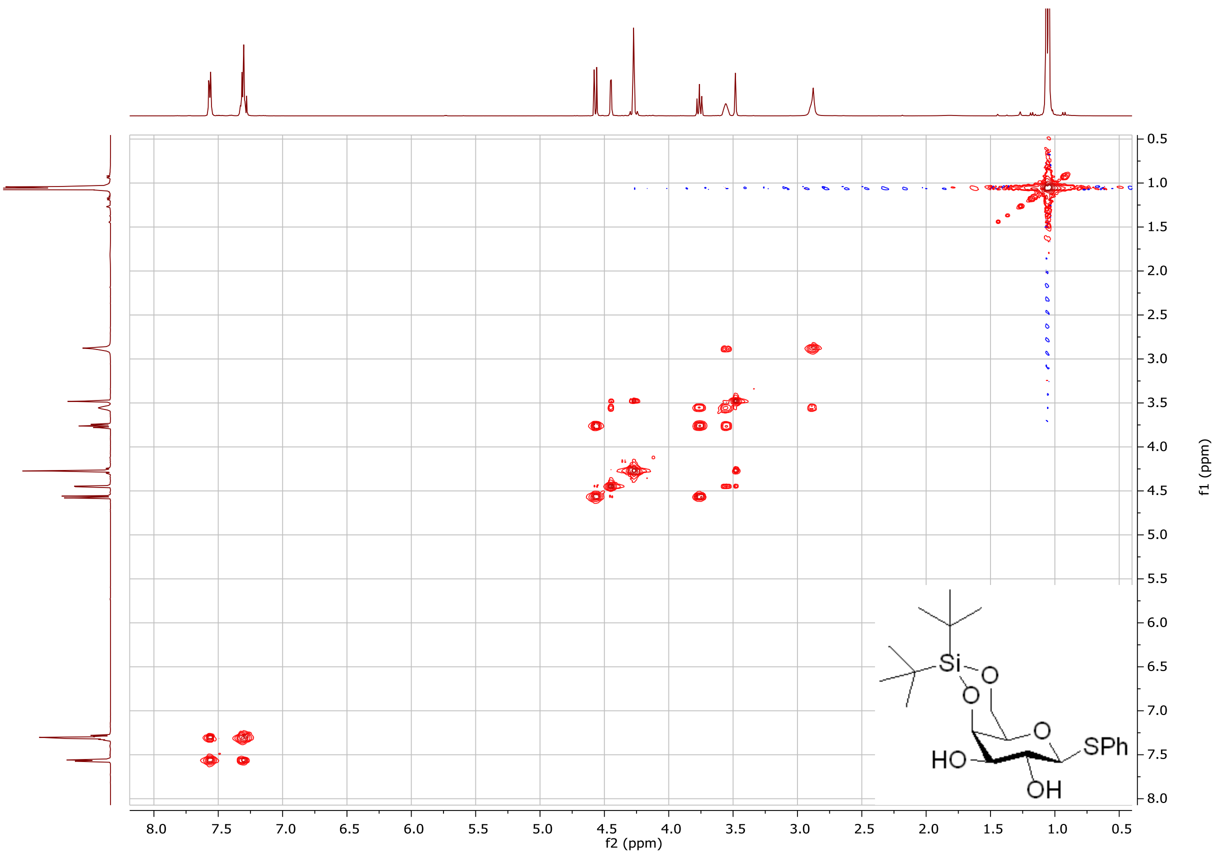
f2 (ppm)
f1 (ppm)
Si
O
O
O
HO
OH
SPh

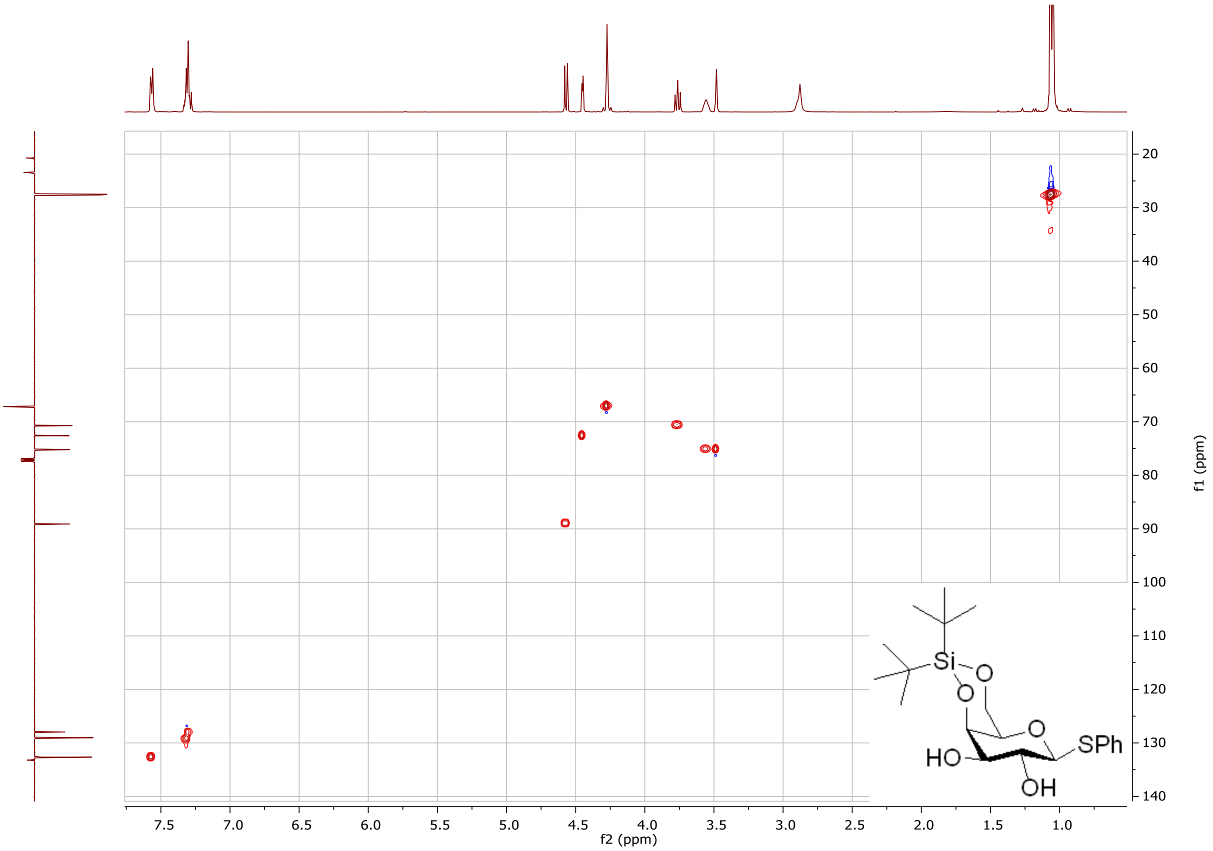
f2 (ppm)
f1 (ppm)
Si
O
O
O
HO
OH
SPh

^{1}H, ^{13}C APT, ^{1}H COSY and HSQC spectra of 12

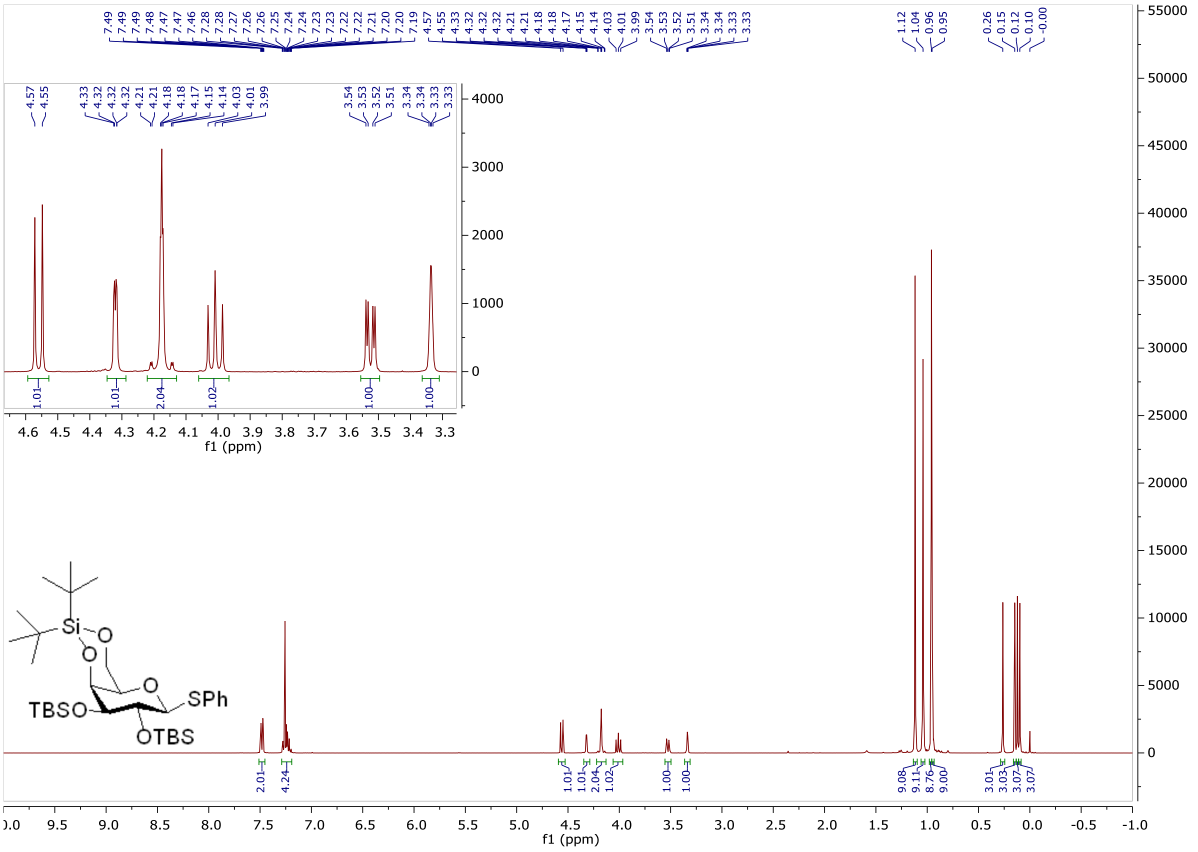

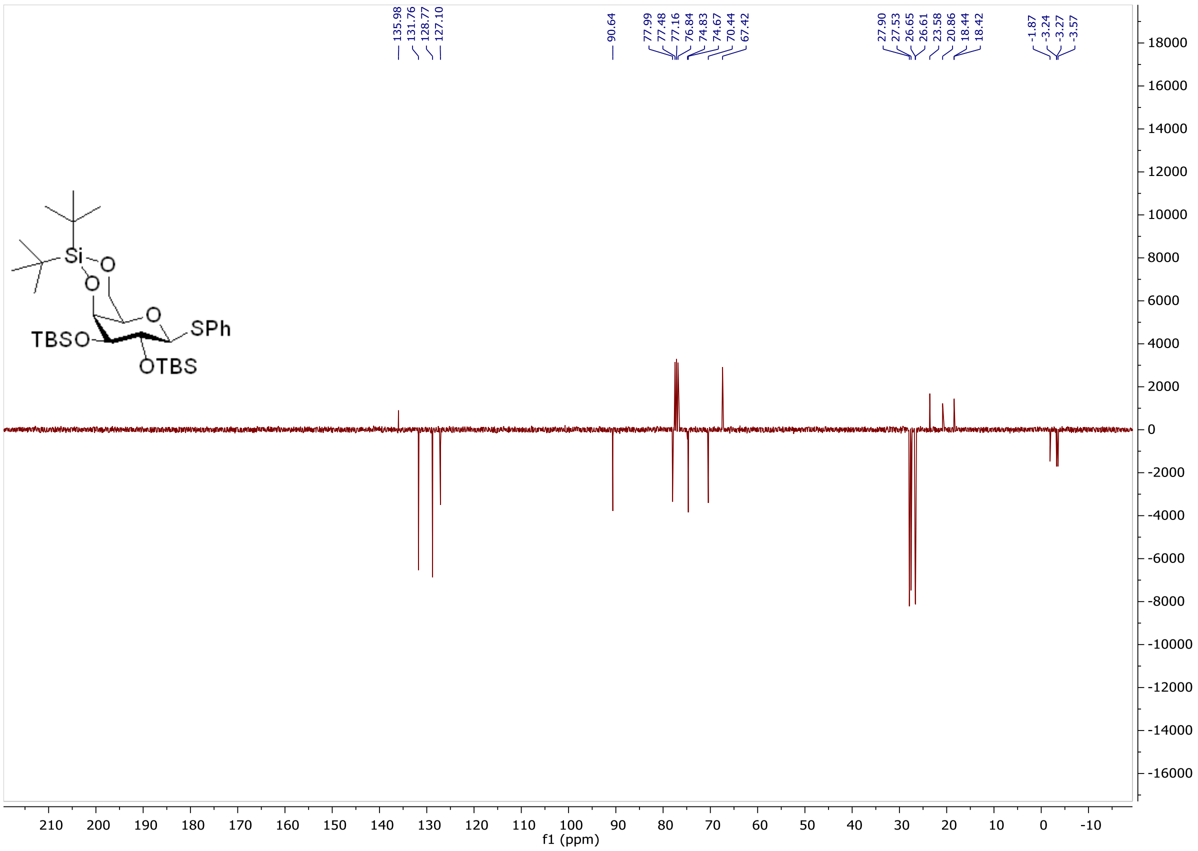

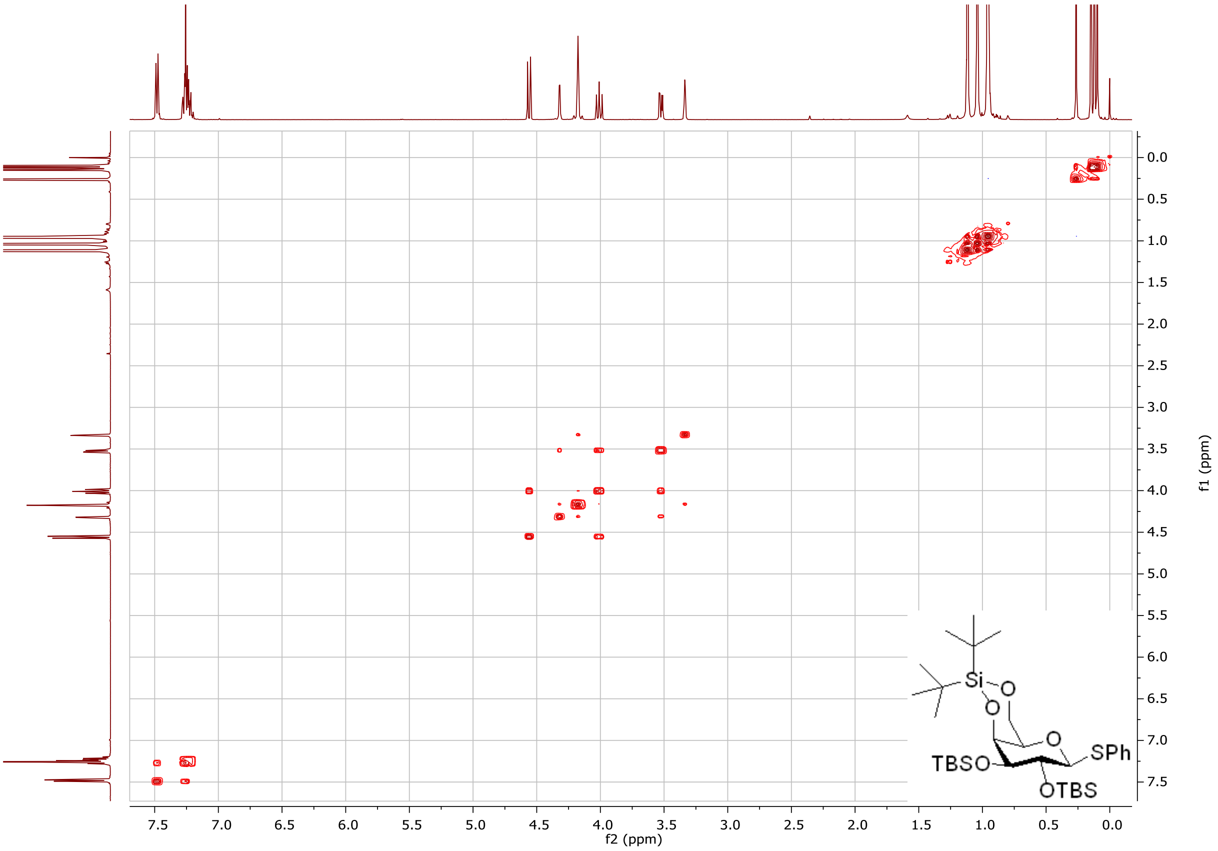
f2 (ppm)
f1 (ppm)
TBSO
OTBS
SPh
Si
O

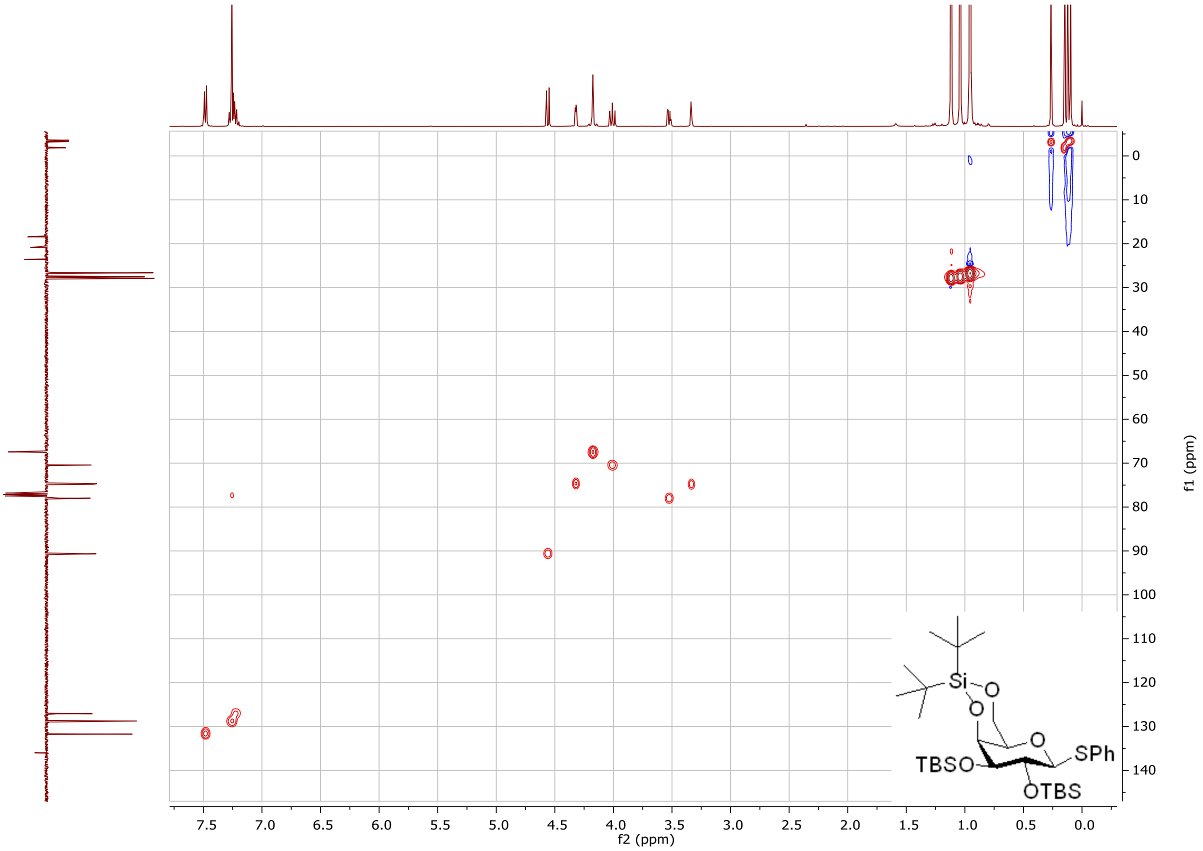
f2 (ppm)
f1 (ppm)
TBSO
OTBS
SPh
Si
O

^{1}H, ^{13}C APT, ^{1}H COSY and HSQC spectra of 41

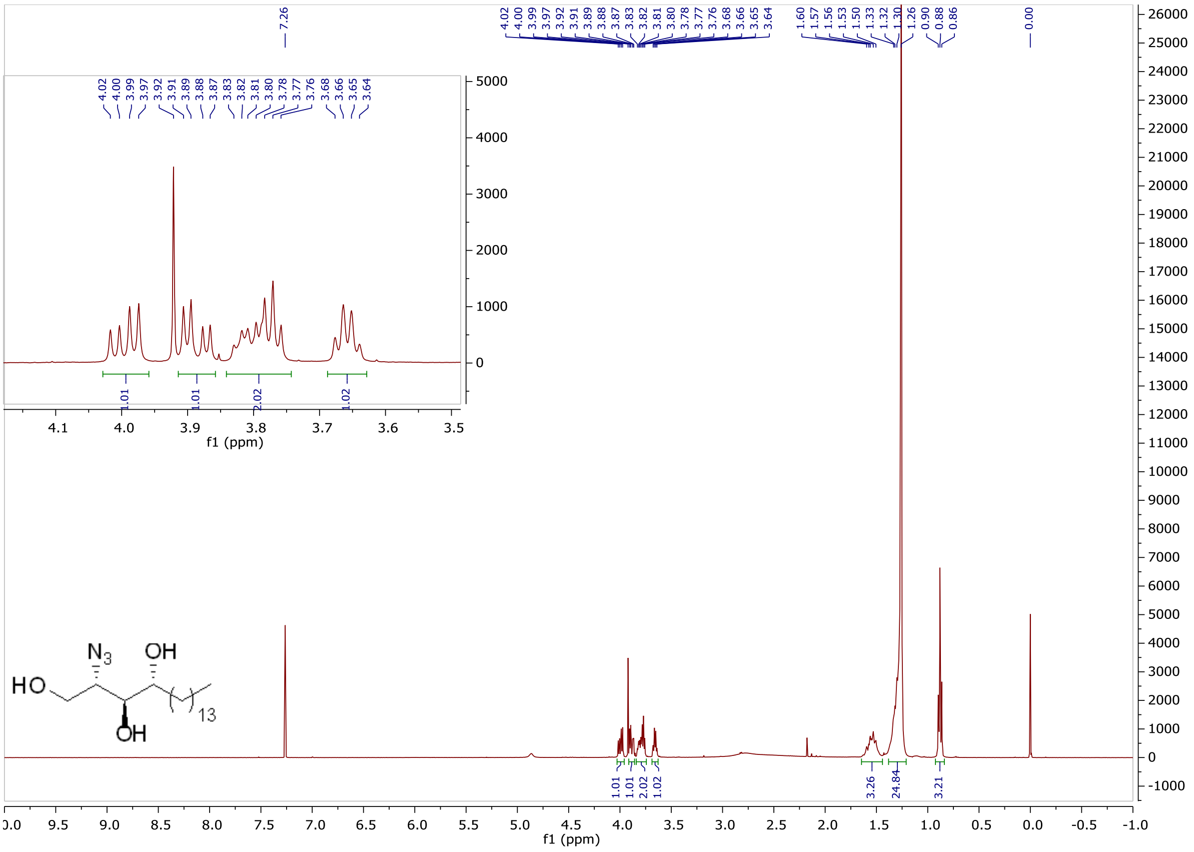

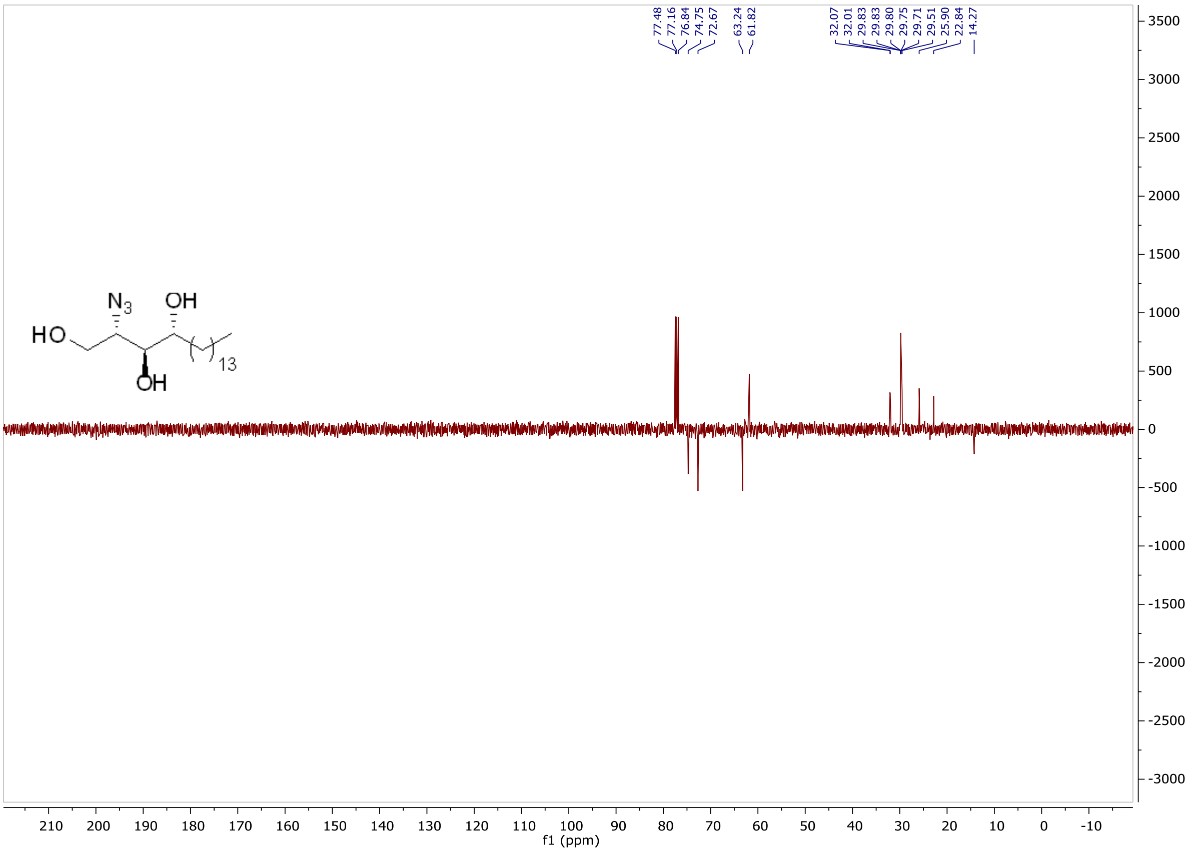

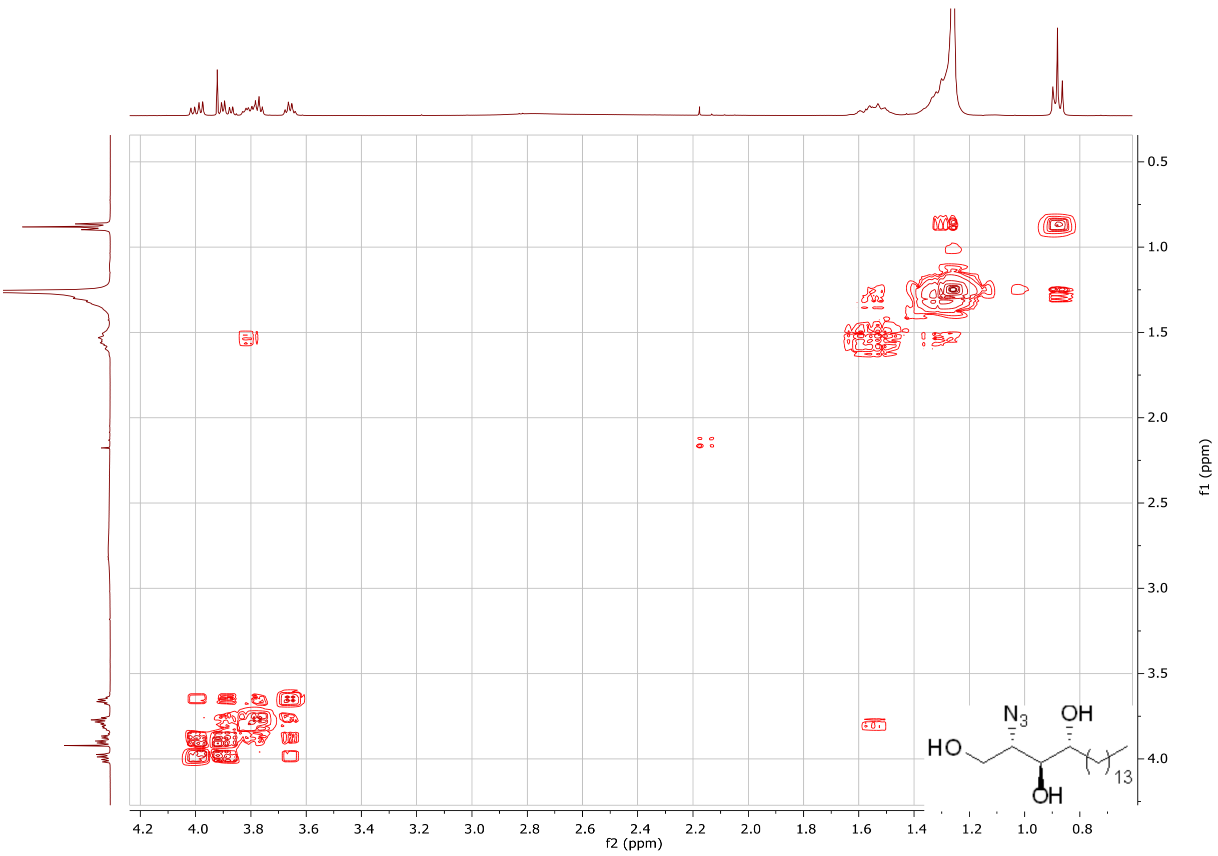
N3
OH
HO
OH
13
f2 (ppm)
f1 (ppm)

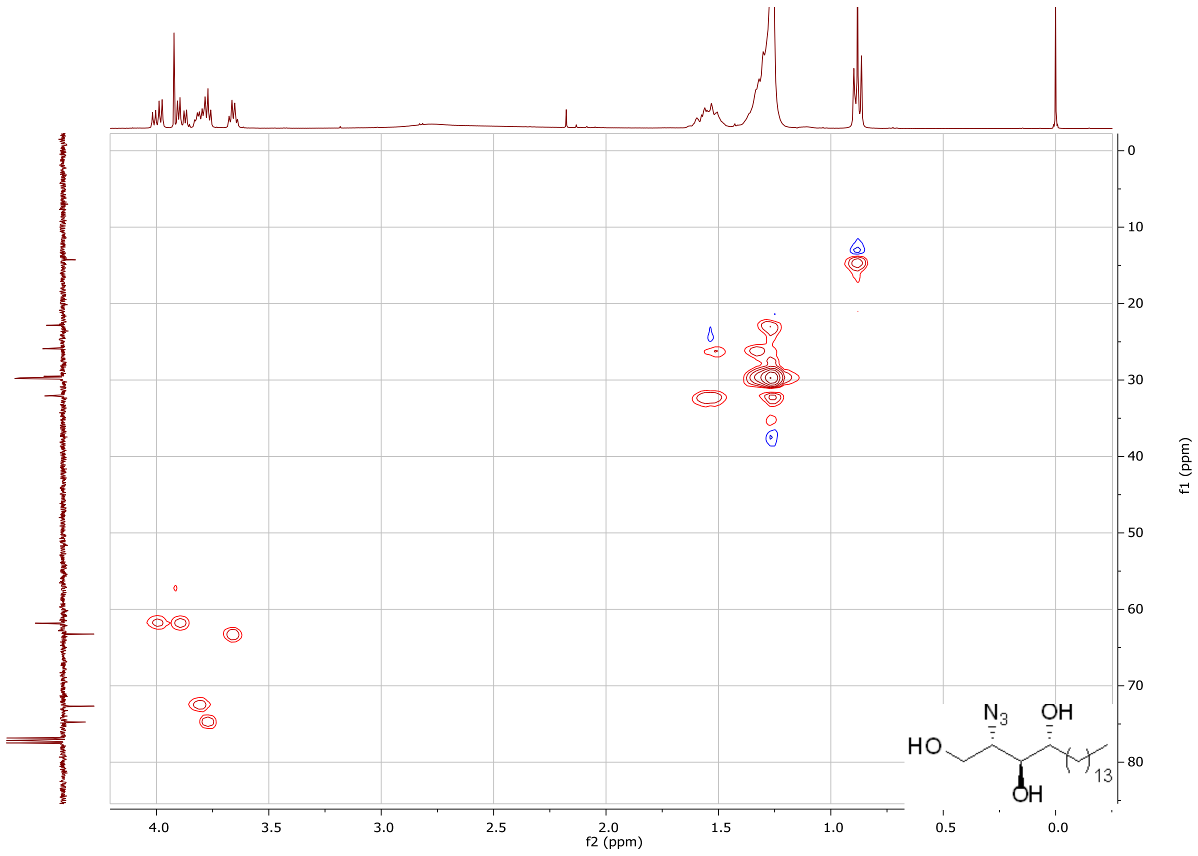
N3
OH
HO
OH
13
f2 (ppm)
f1 (ppm)

^{1}H, ^{13}C APT, ^{1}H COSY and HSQC spectra of 42

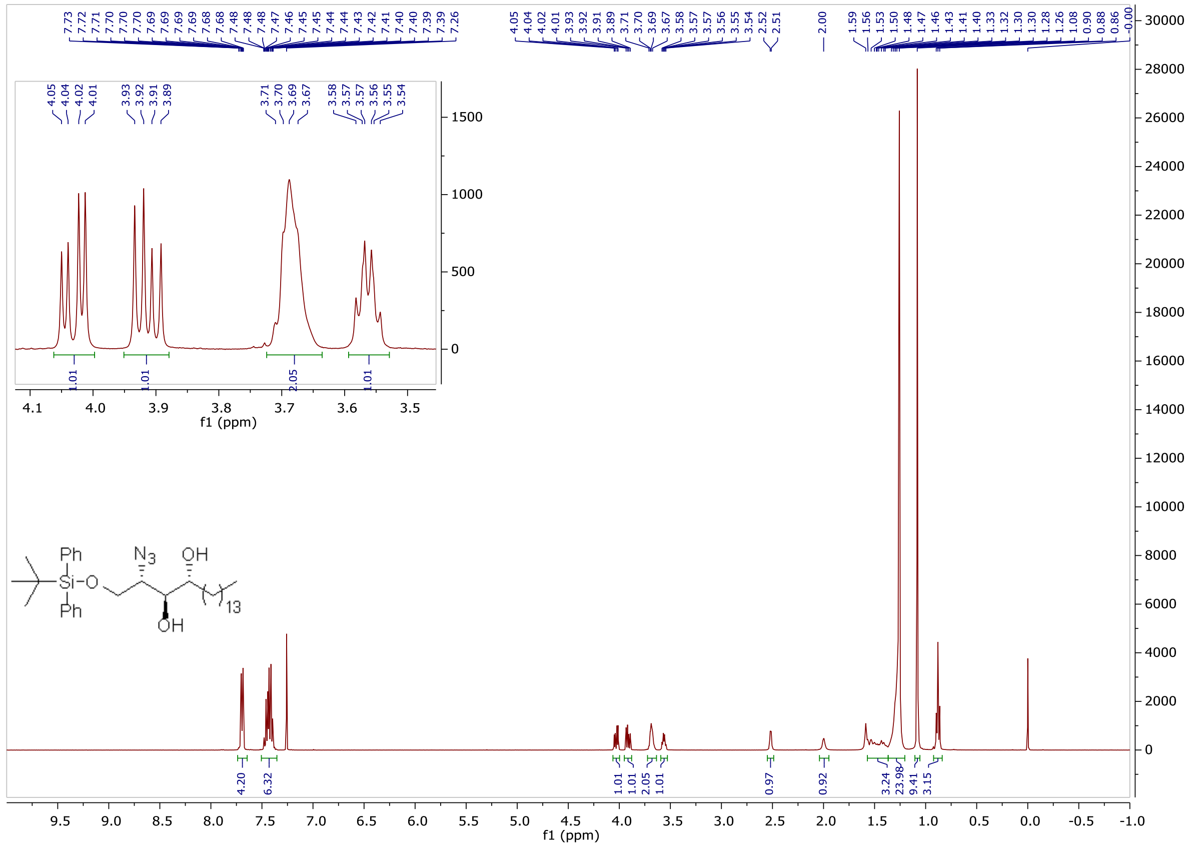

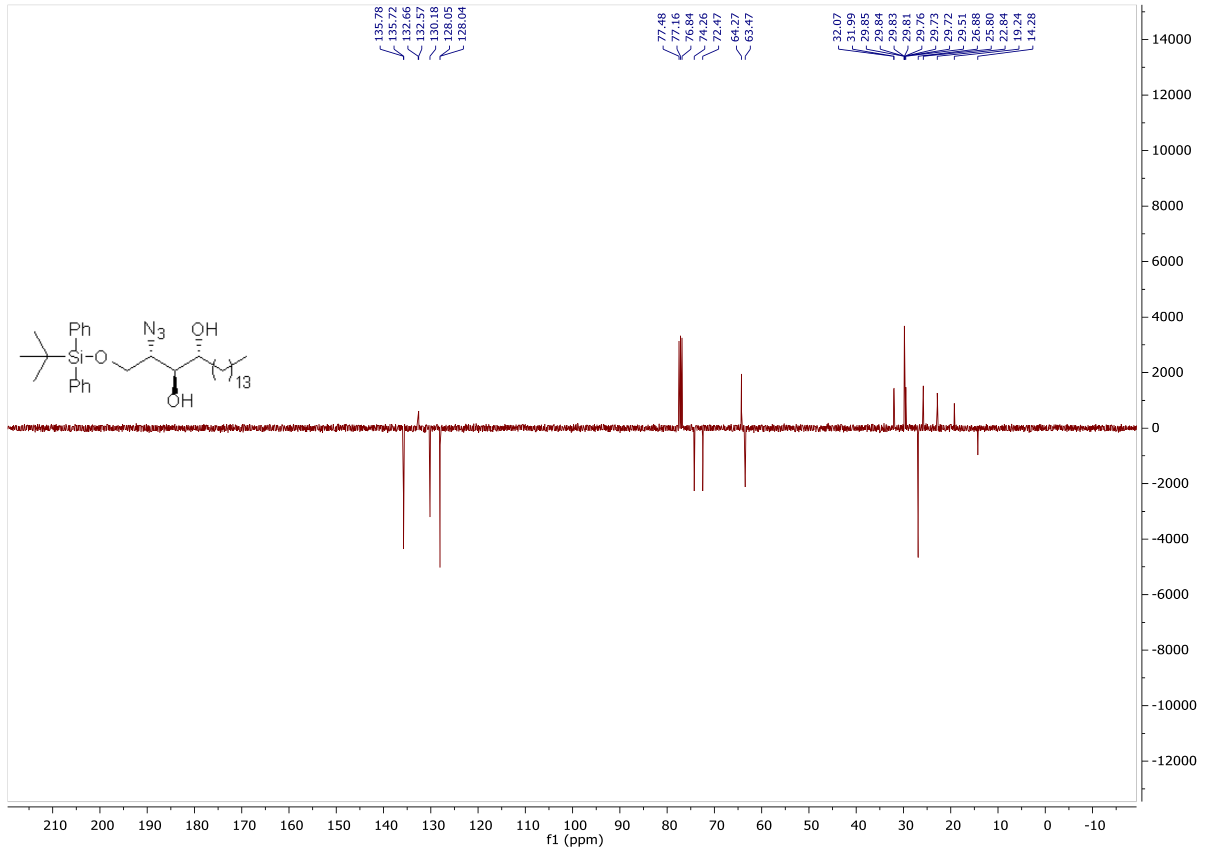

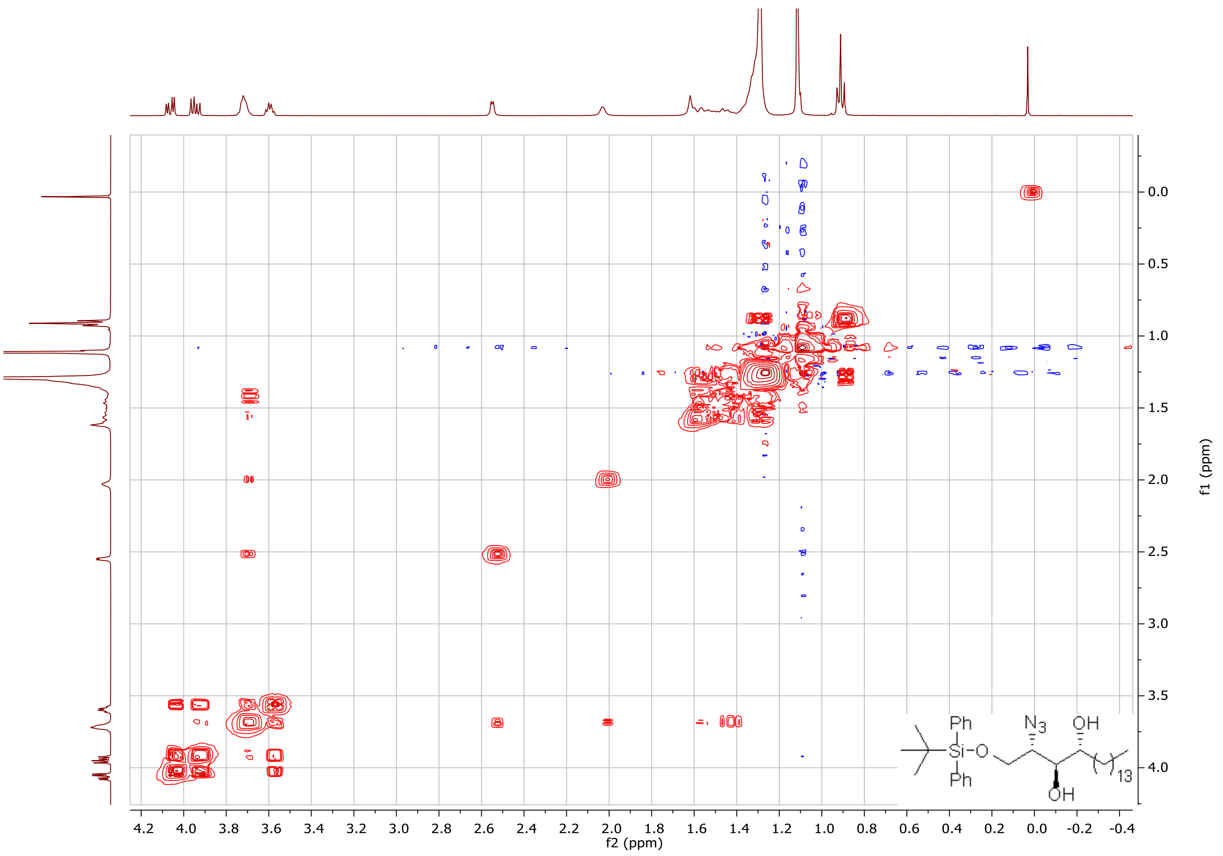
f2 (ppm)
f1 (ppm)
Ph
Si
O
Ph
N3
OH
OH
13

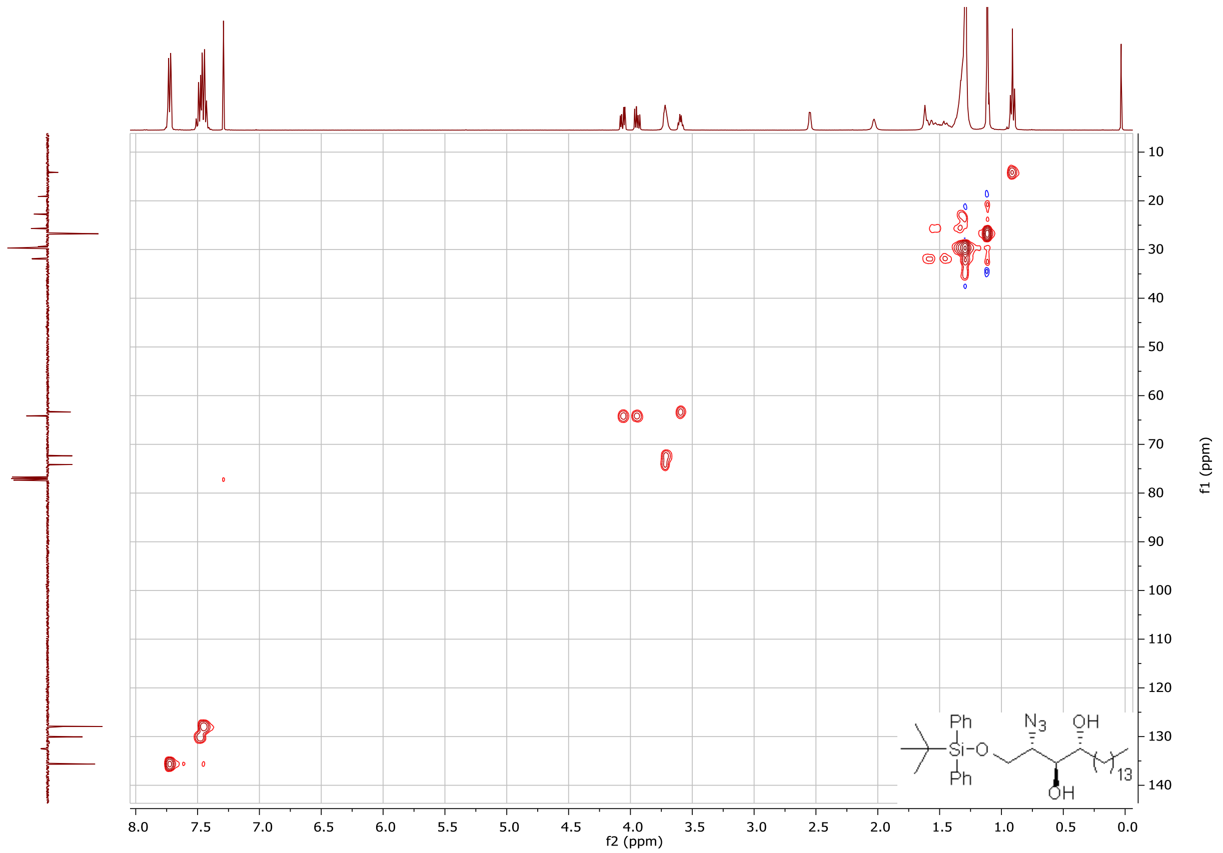
f2 (ppm)
f1 (ppm)
Ph
Si
O
Ph
N3
OH
OH
13

^{1}H, ^{13}C APT, ^{1}H COSY and HSQC spectra of 43

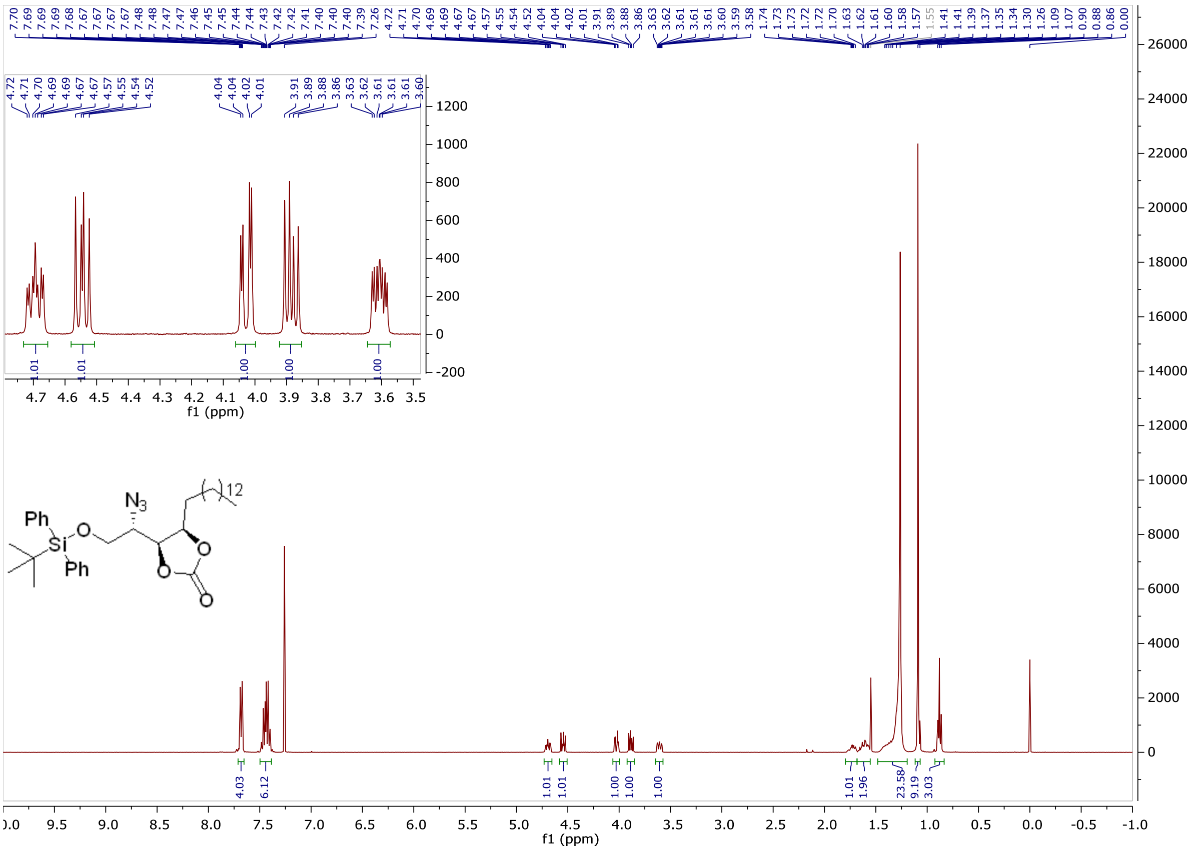

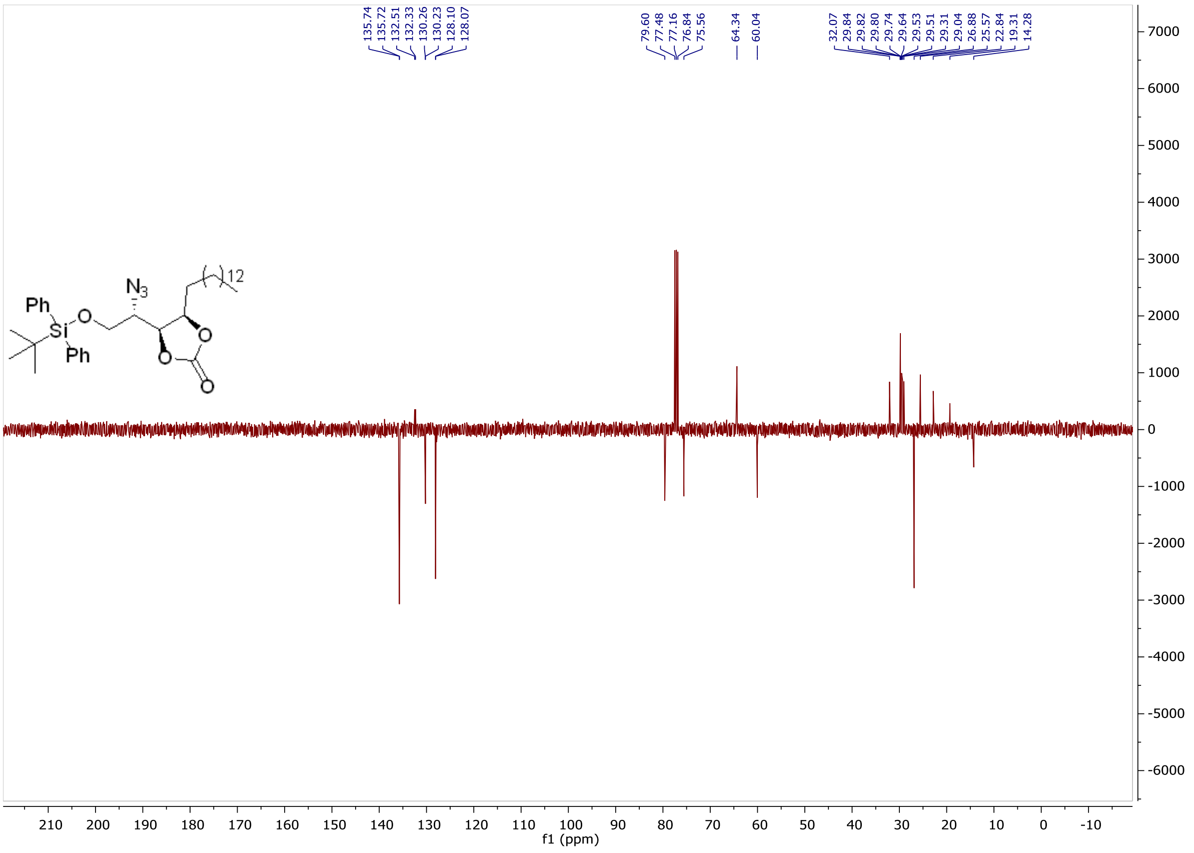

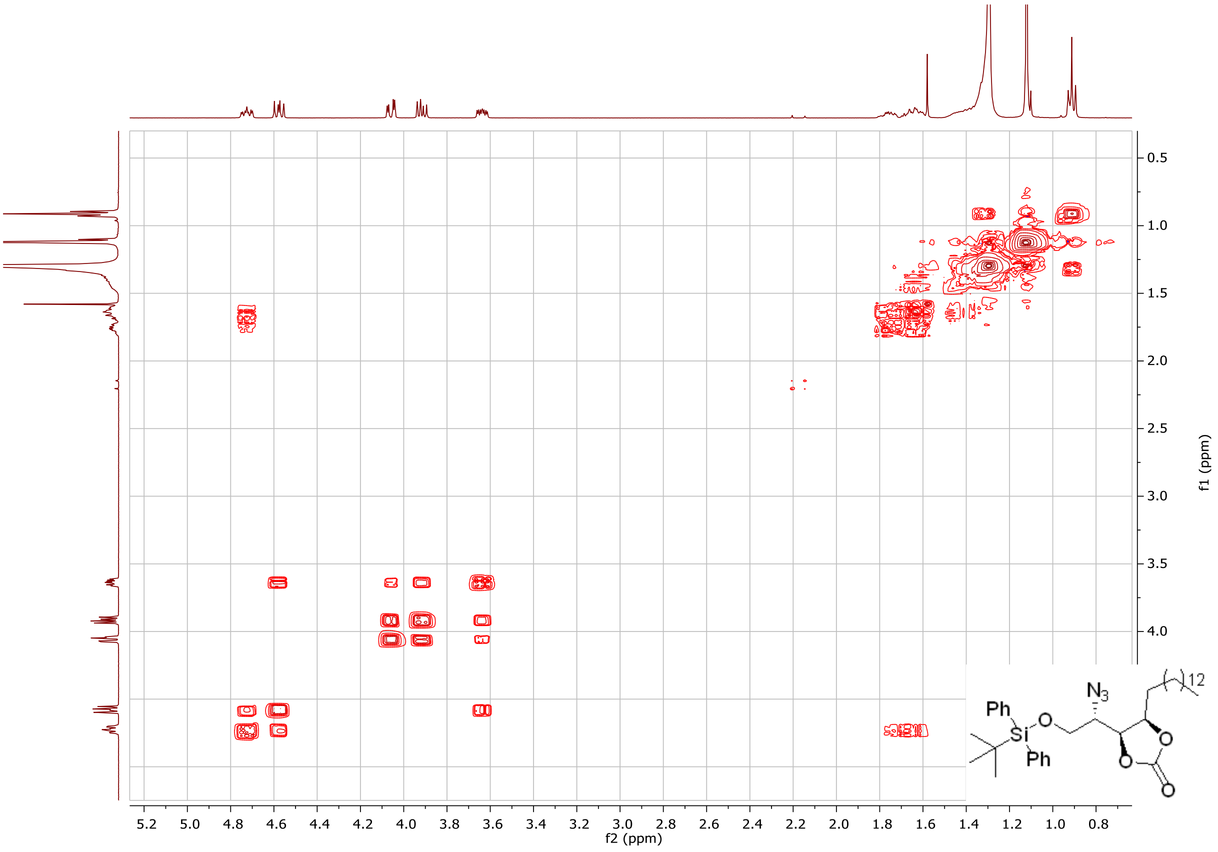
f2 (ppm)
f1 (ppm)
Ph
Si
O
Ph
N3
O
O
O
12

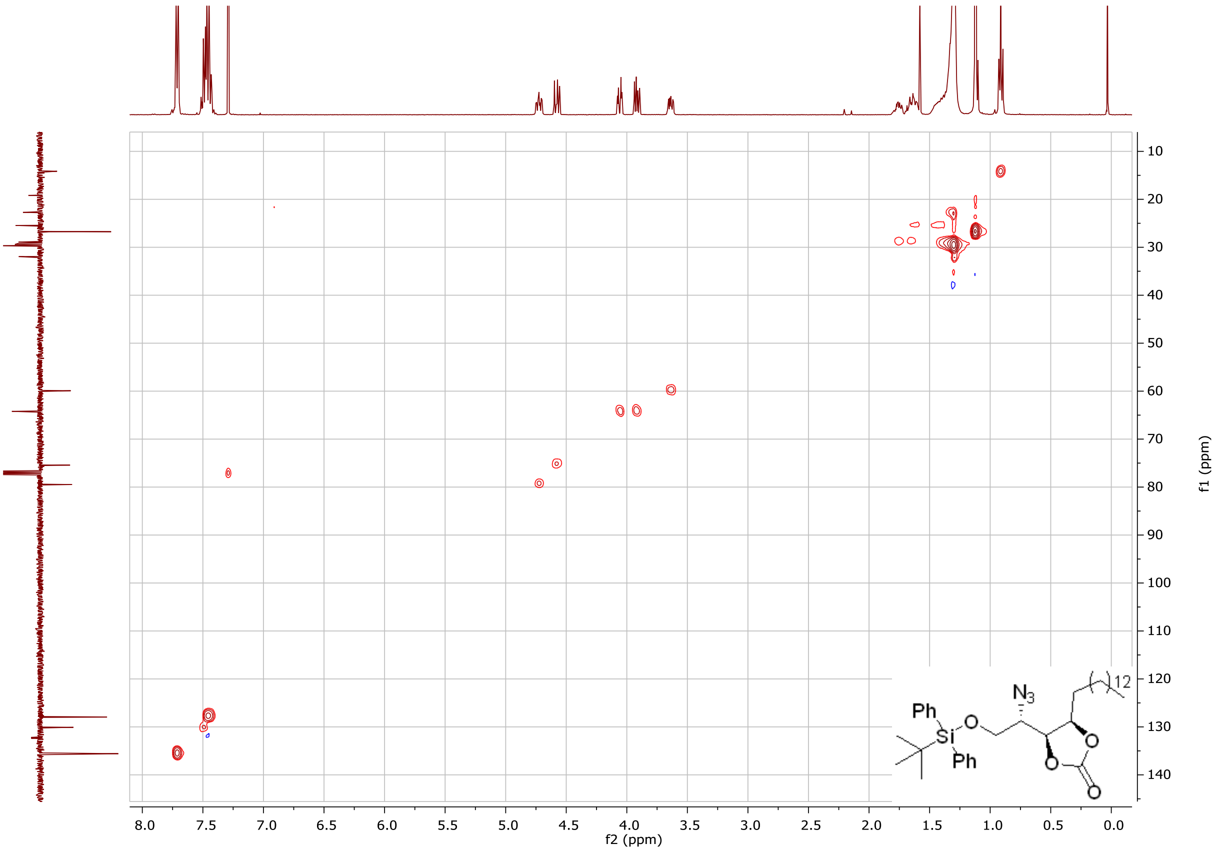
f2 (ppm)
f1 (ppm)
Ph
Si
O
Ph
N3
O
O
O
12

^{1}H, ^{13}C APT, ^{1}H COSY and HSQC spectra of 13

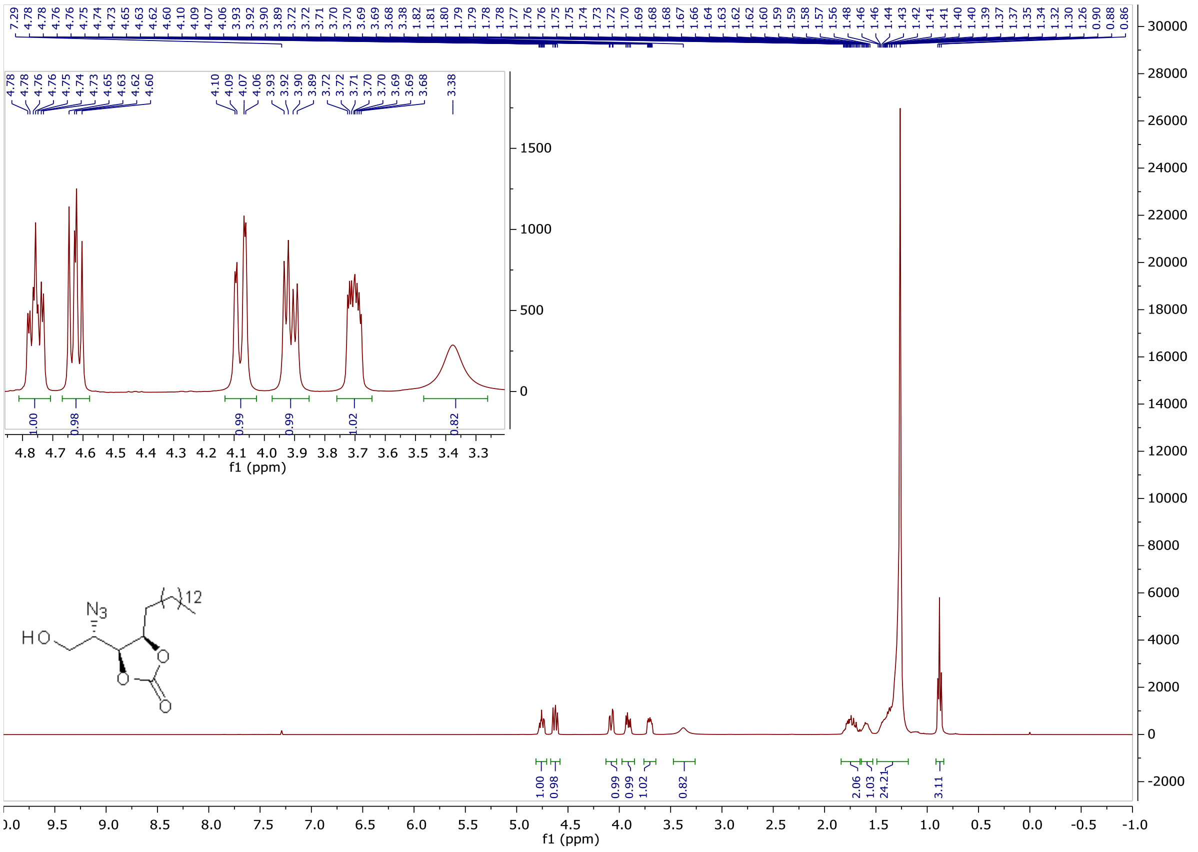

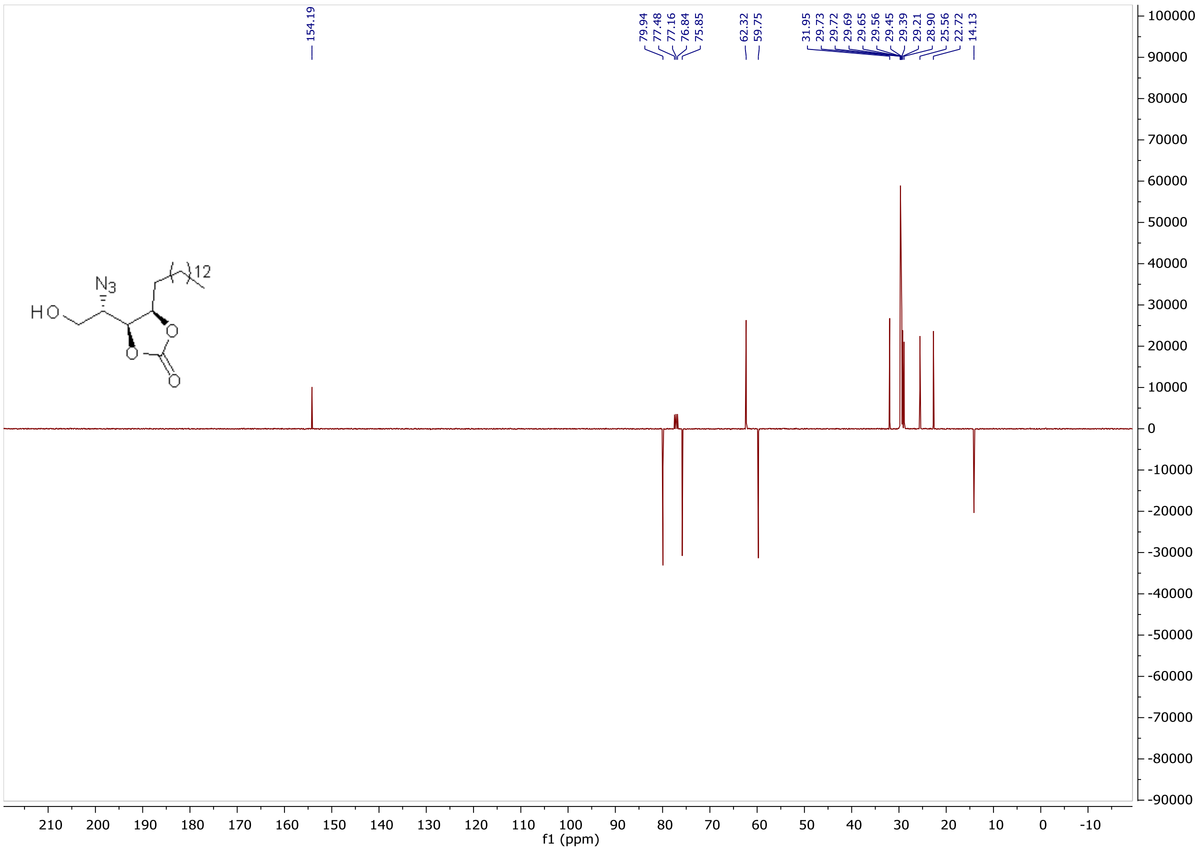

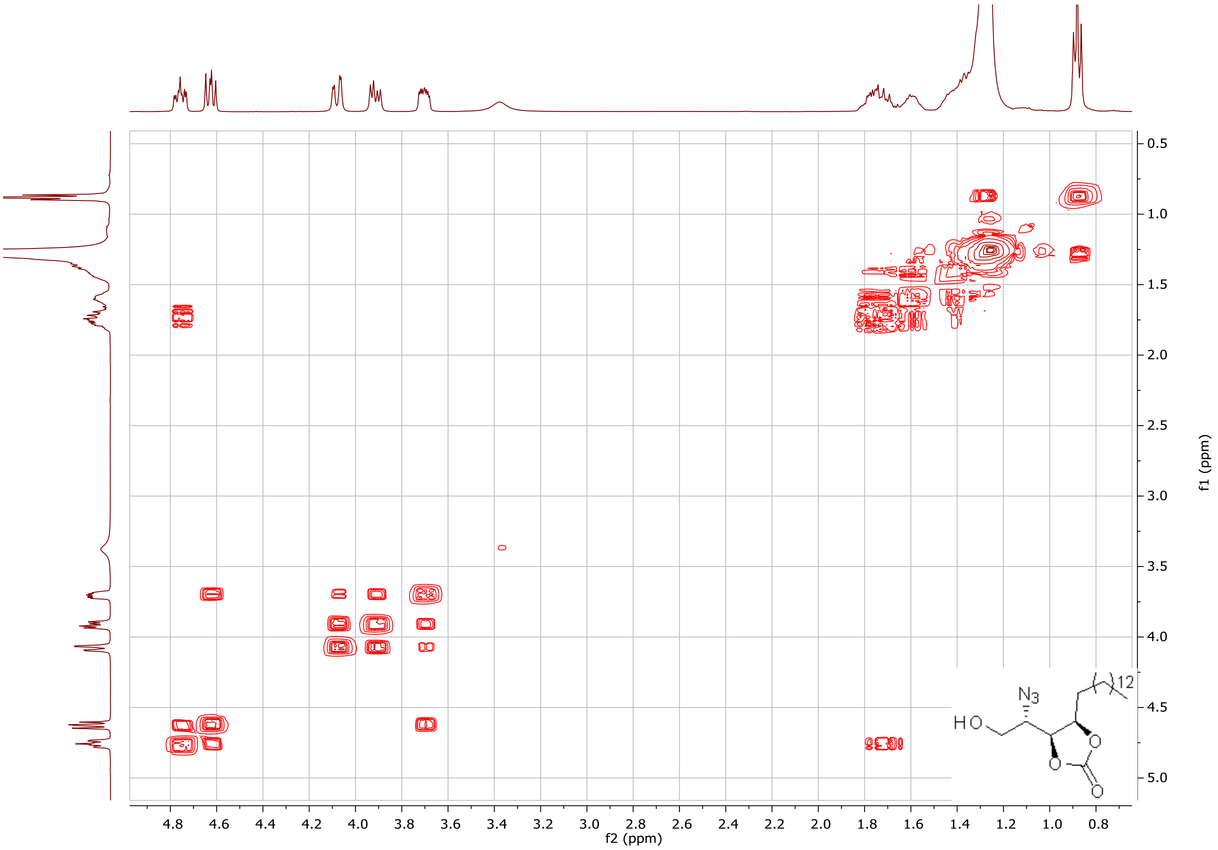
f2 (ppm)
f1 (ppm)
N3
HO
O
O
O
12

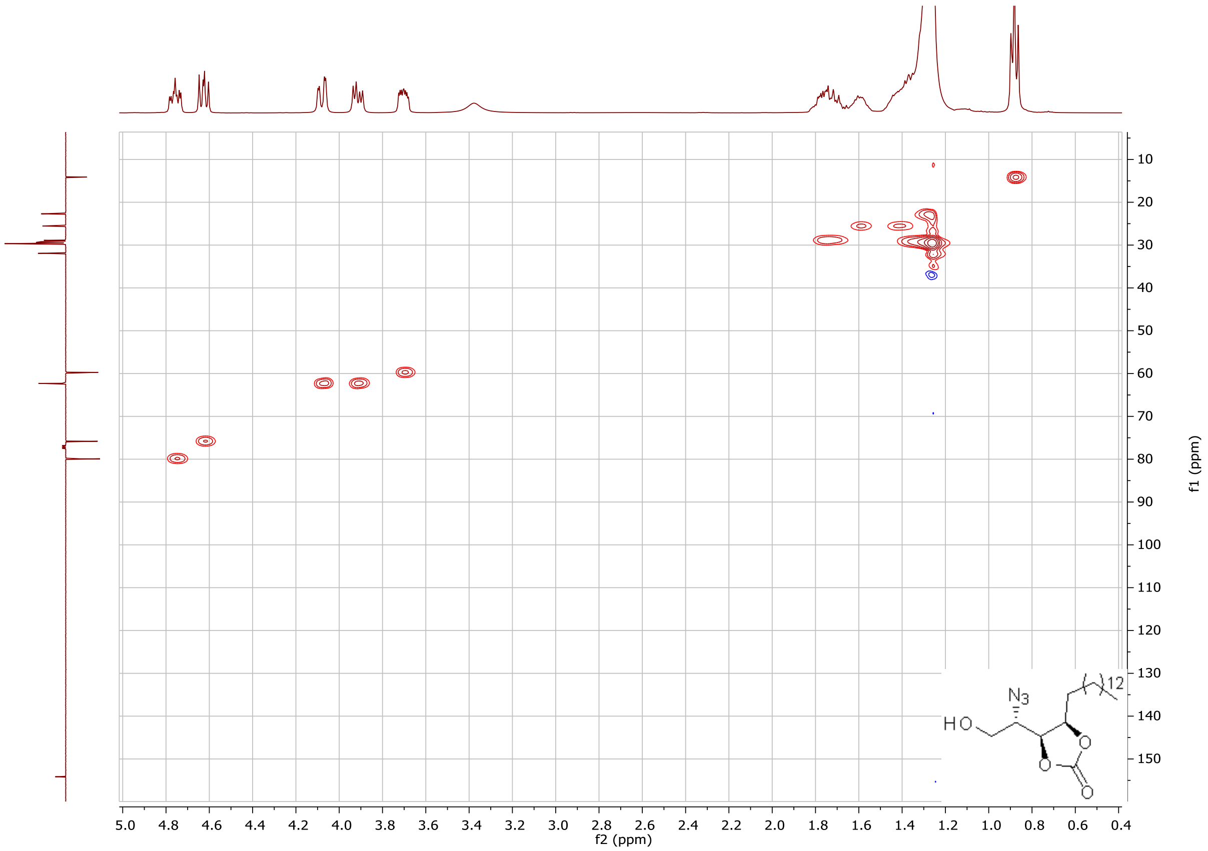
f2 (ppm)
f1 (ppm)
N3
HO
O
O
O
12

^{1}H, ^{13}C APT, ^{1}H COSY and HSQC spectra of 11

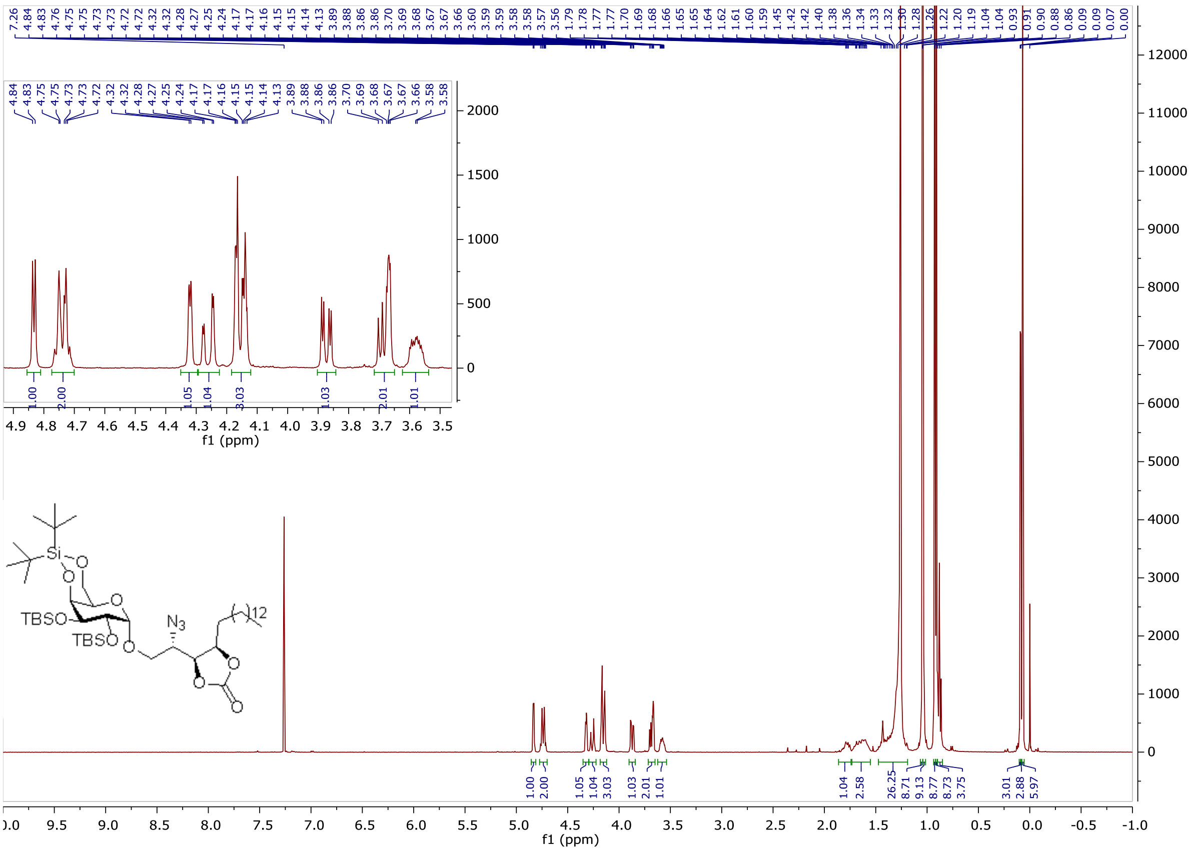

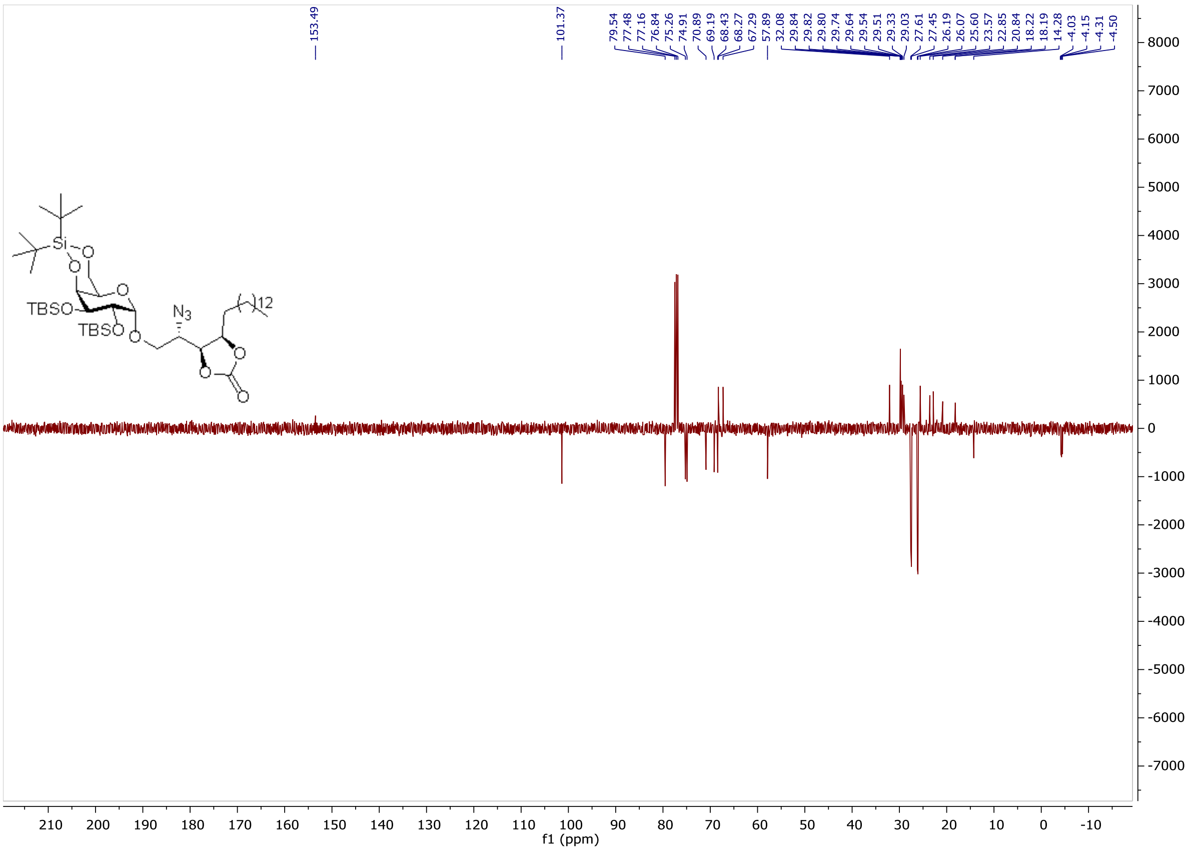

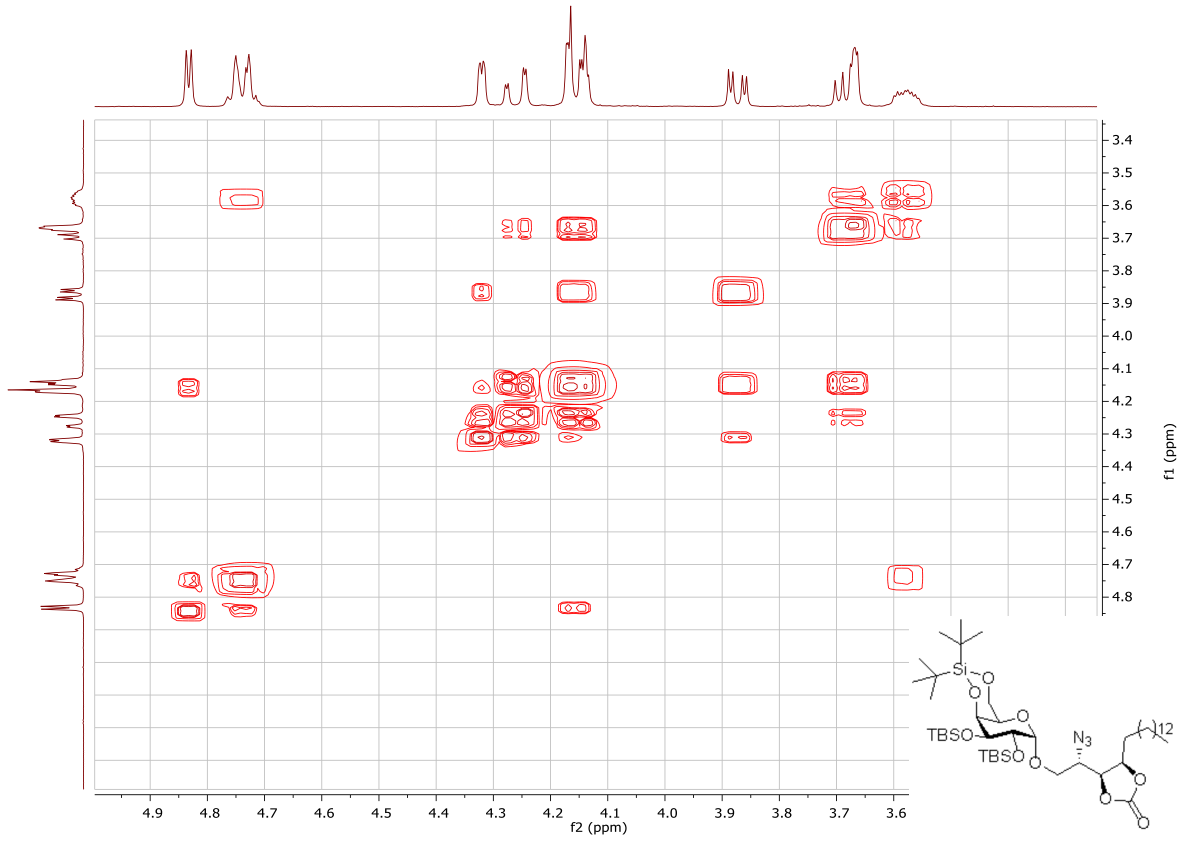
f2 (ppm)
f1 (ppm)
TBSO
TBSO
N_3
12

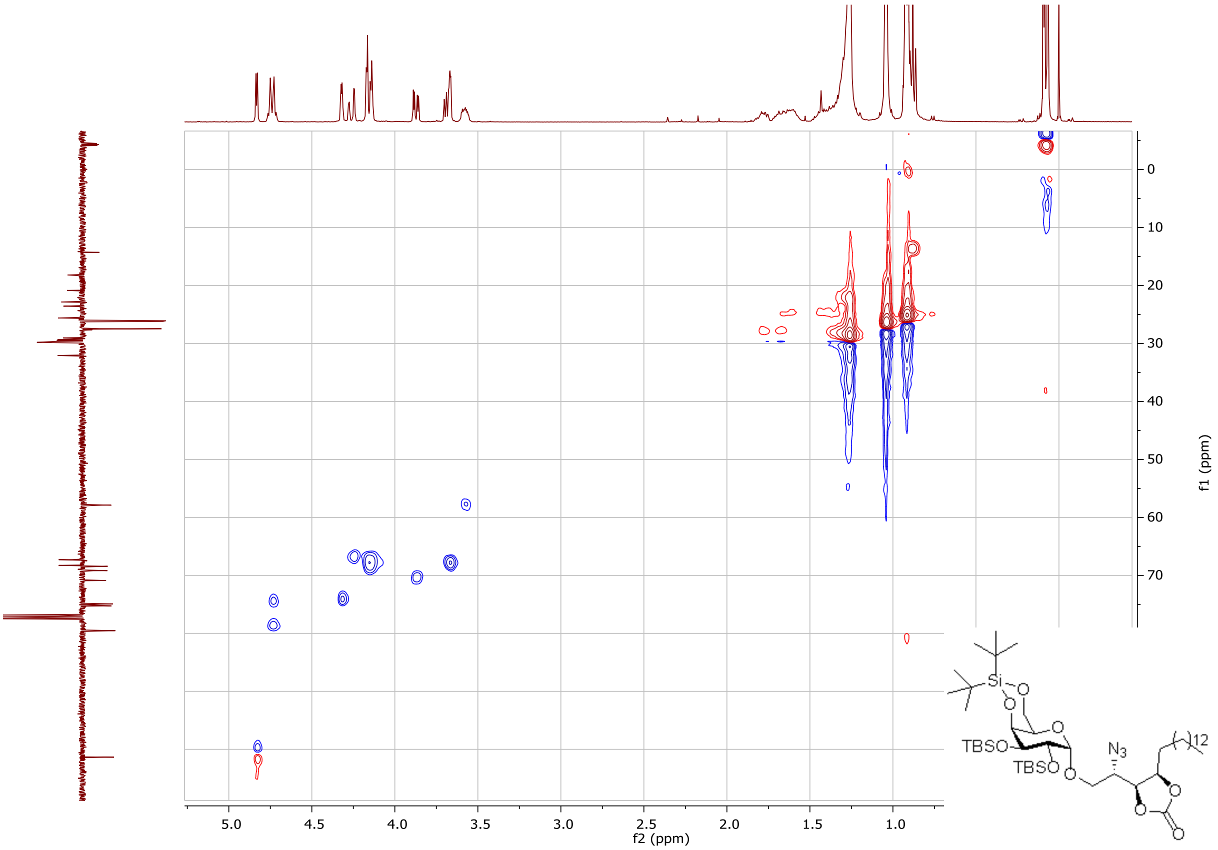
f2 (ppm)
f1 (ppm)
TBSO
TBSO
N_3
12

^{1}H, ^{13}C APT, ^{1}H COSY and HSQC spectra of 14

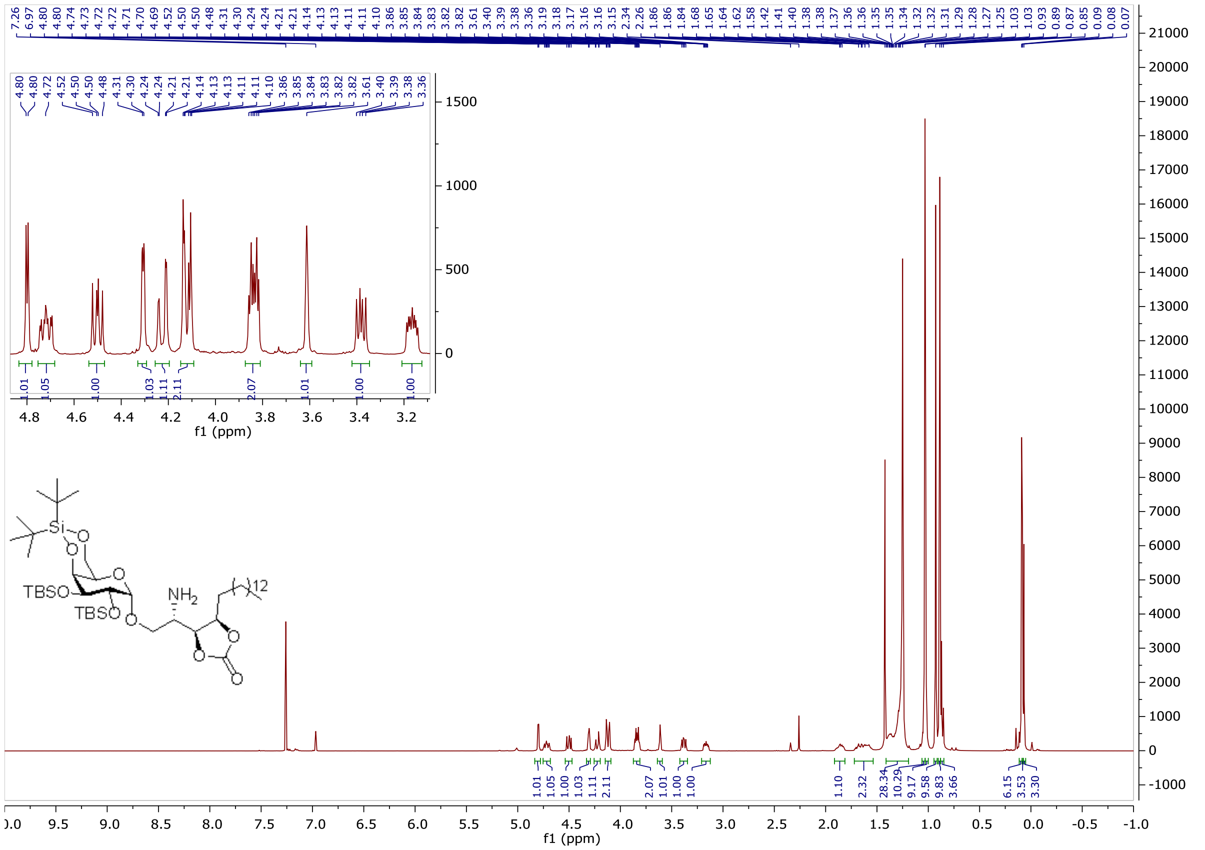

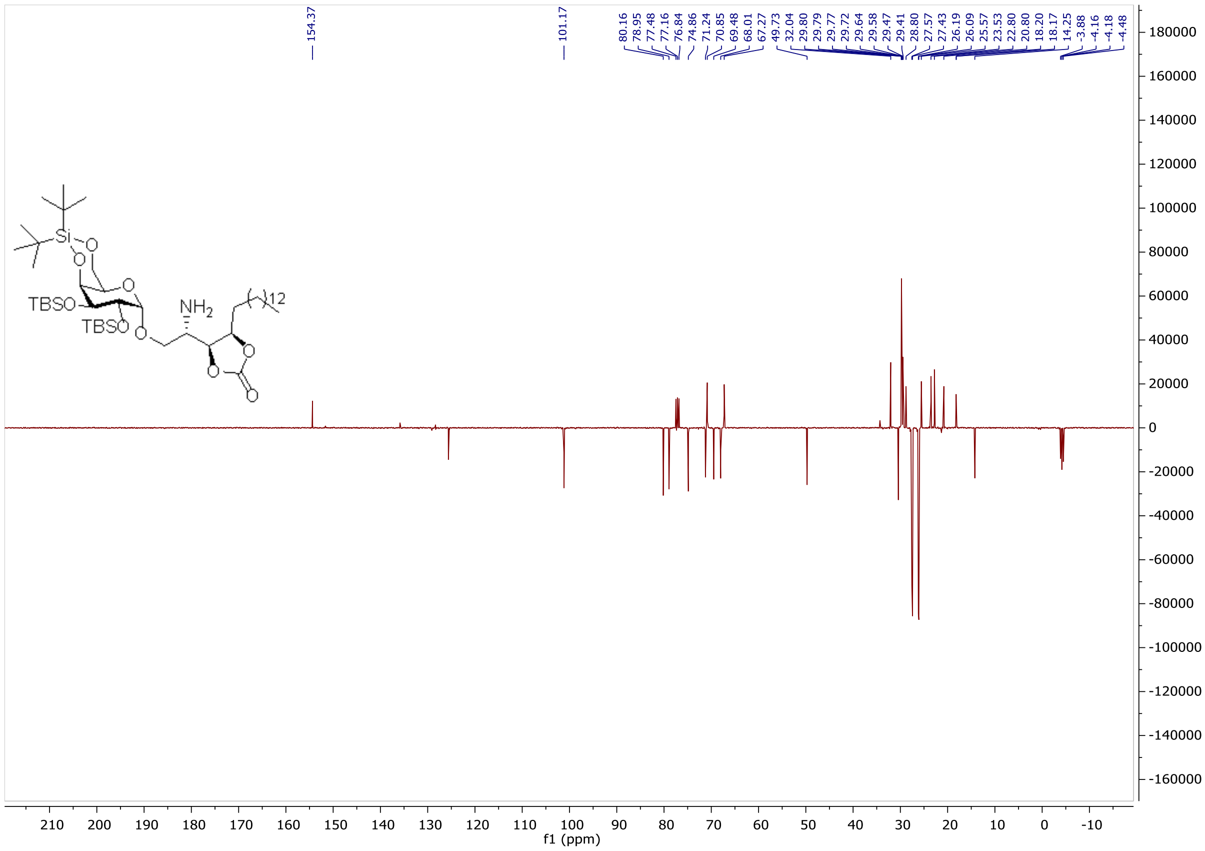

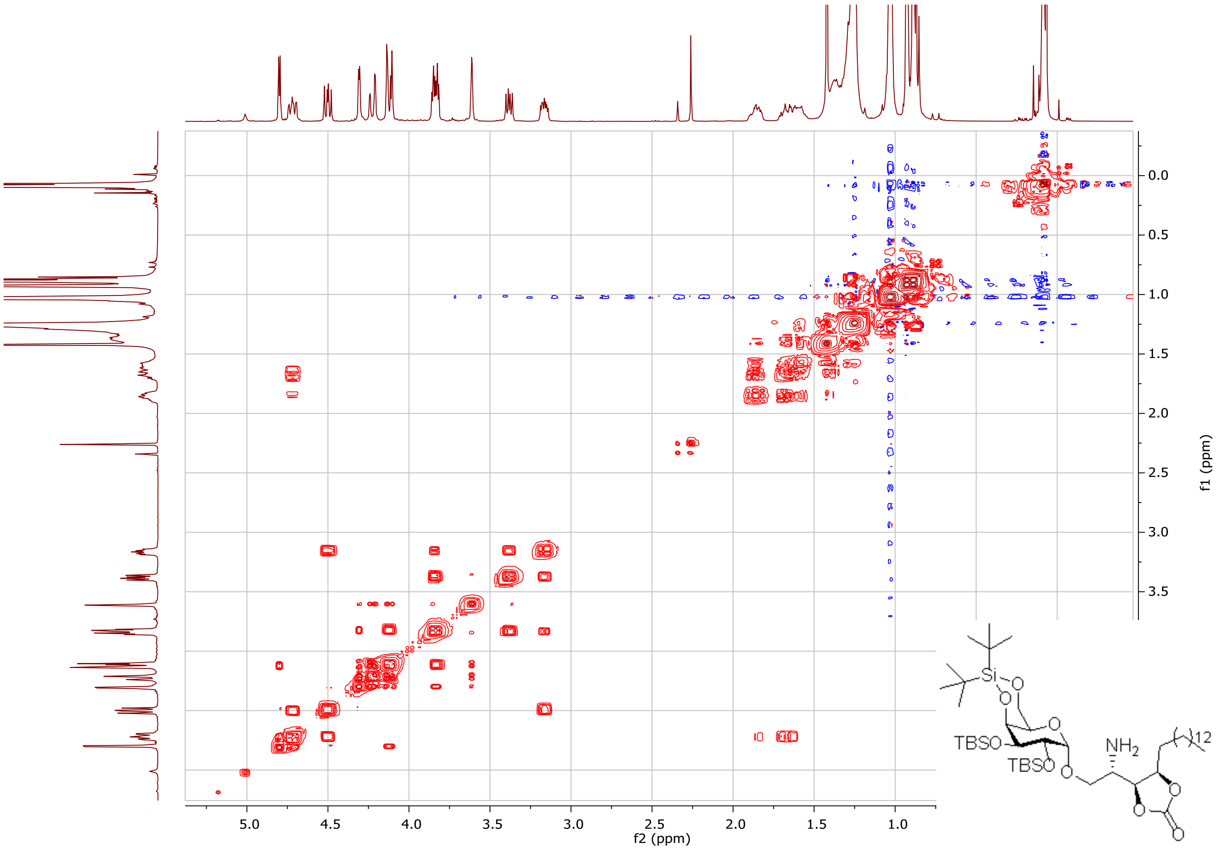
f2 (ppm)
f1 (ppm)
TBSO
TBSO
NH2
12
Si
O

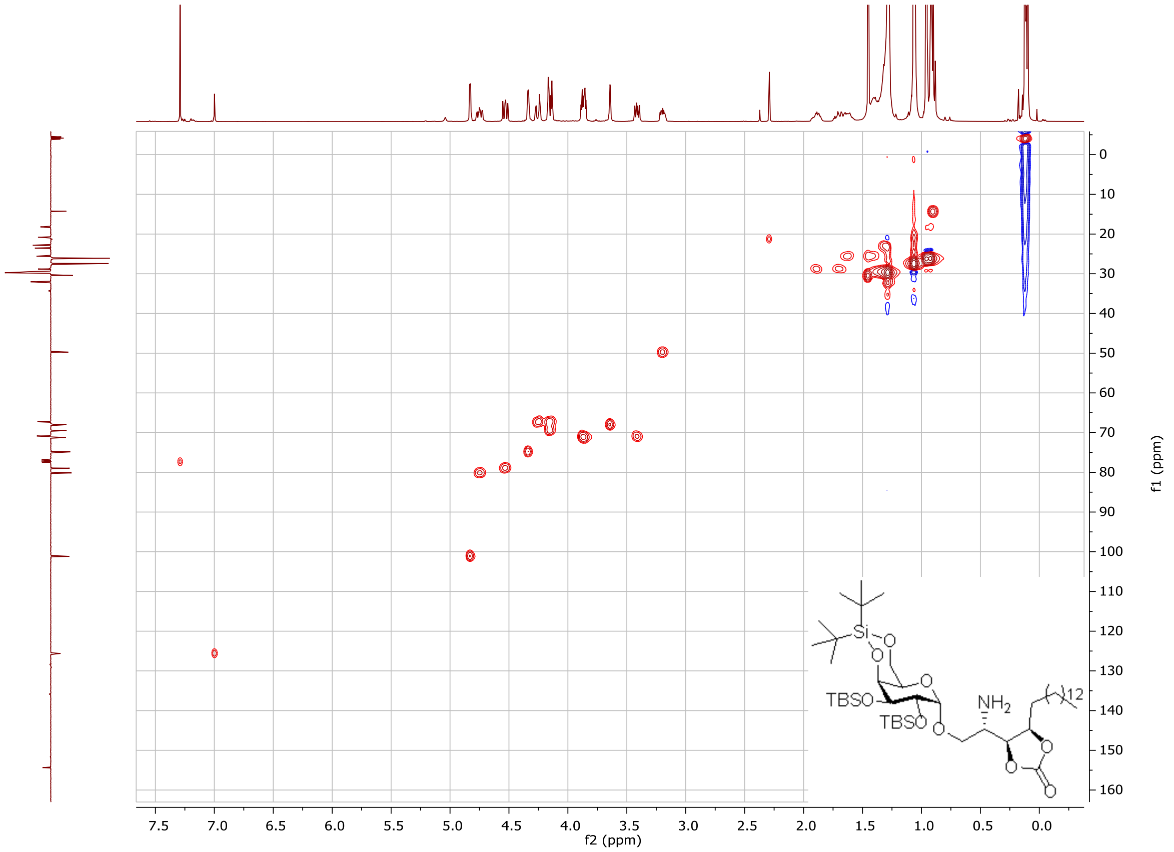
f2 (ppm)
f1 (ppm)
TBSO
TBSO
NH2
12
Si
O

^{1}H, ^{13}C APT, ^{1}H COSY and HSQC spectra of 16

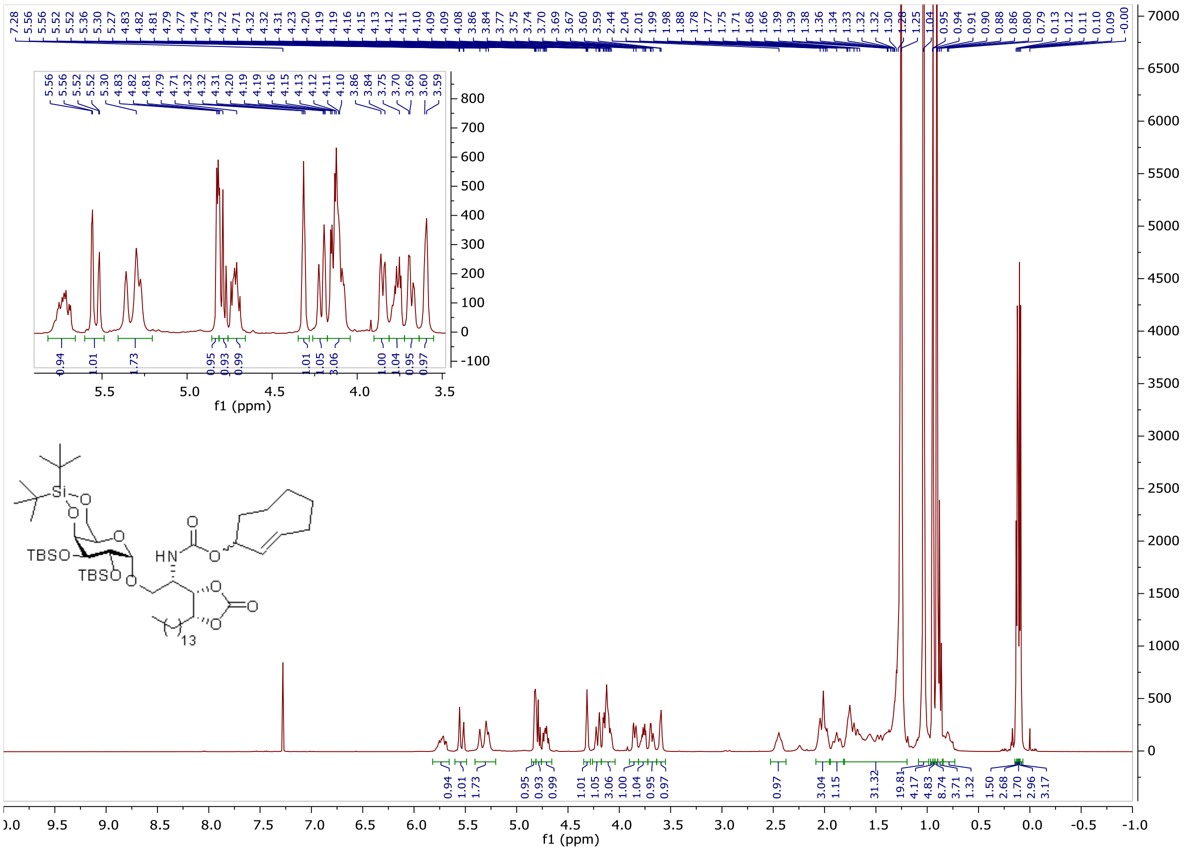

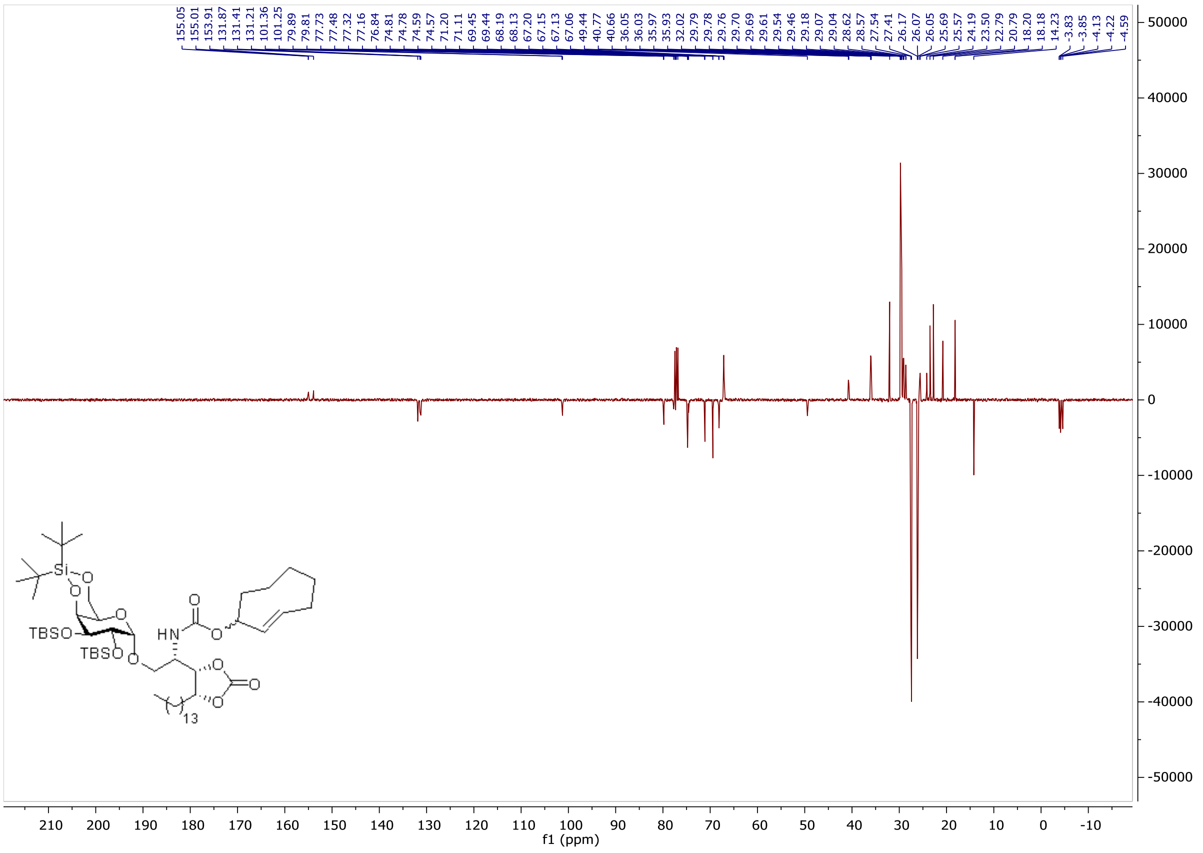

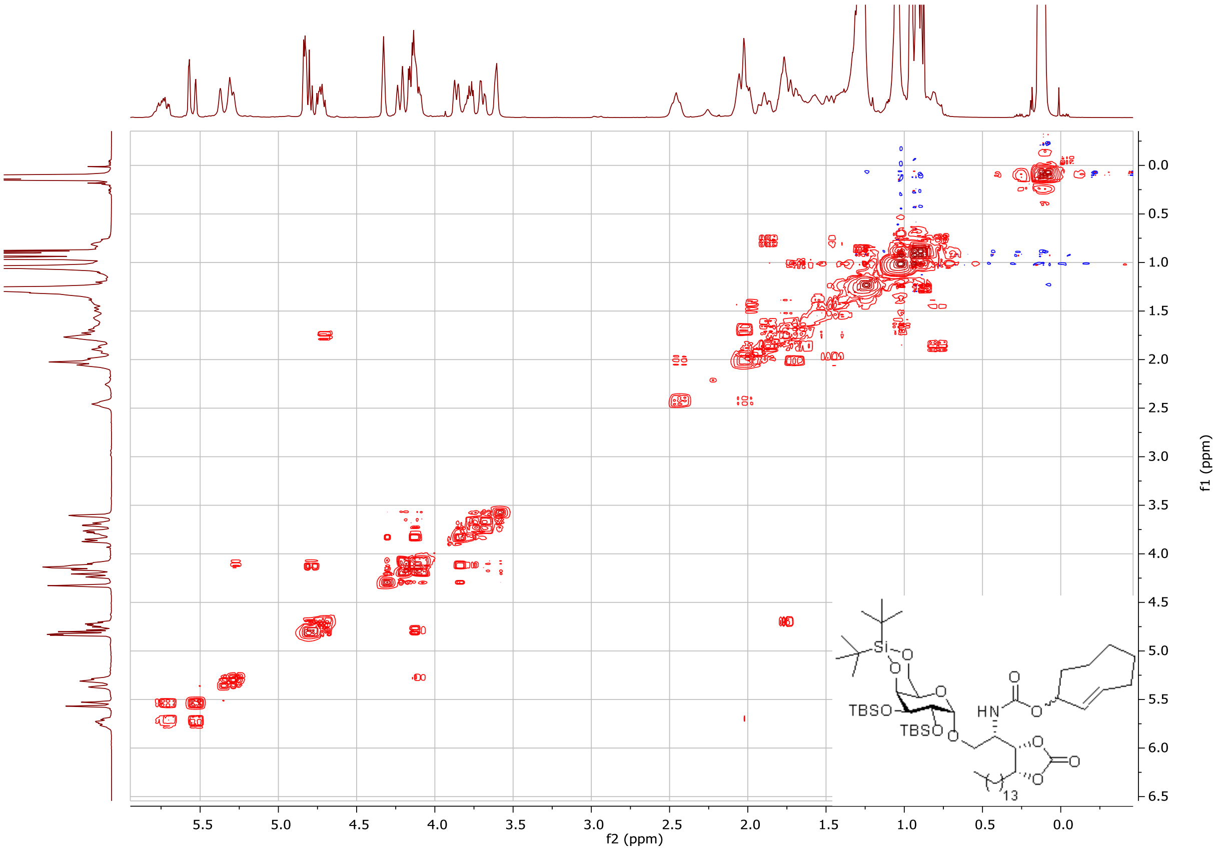

f1 (ppm)
f2 (ppm)
TBSO
TBSO
HN
Si
13

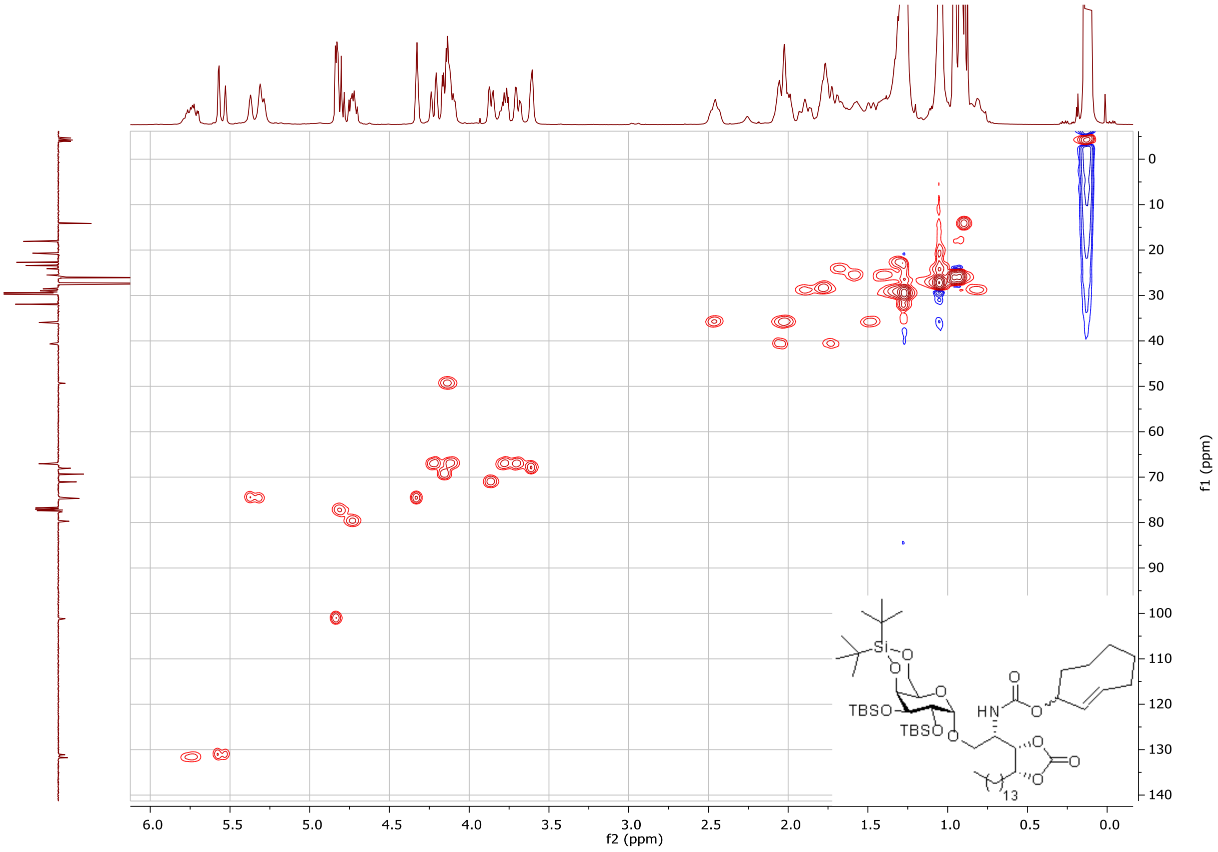

f1 (ppm)
f2 (ppm)
TBSO
TBSO
HN
Si
13

^{1}H, ^{13}C APT, ^{1}H COSY and HSQC spectra of 17

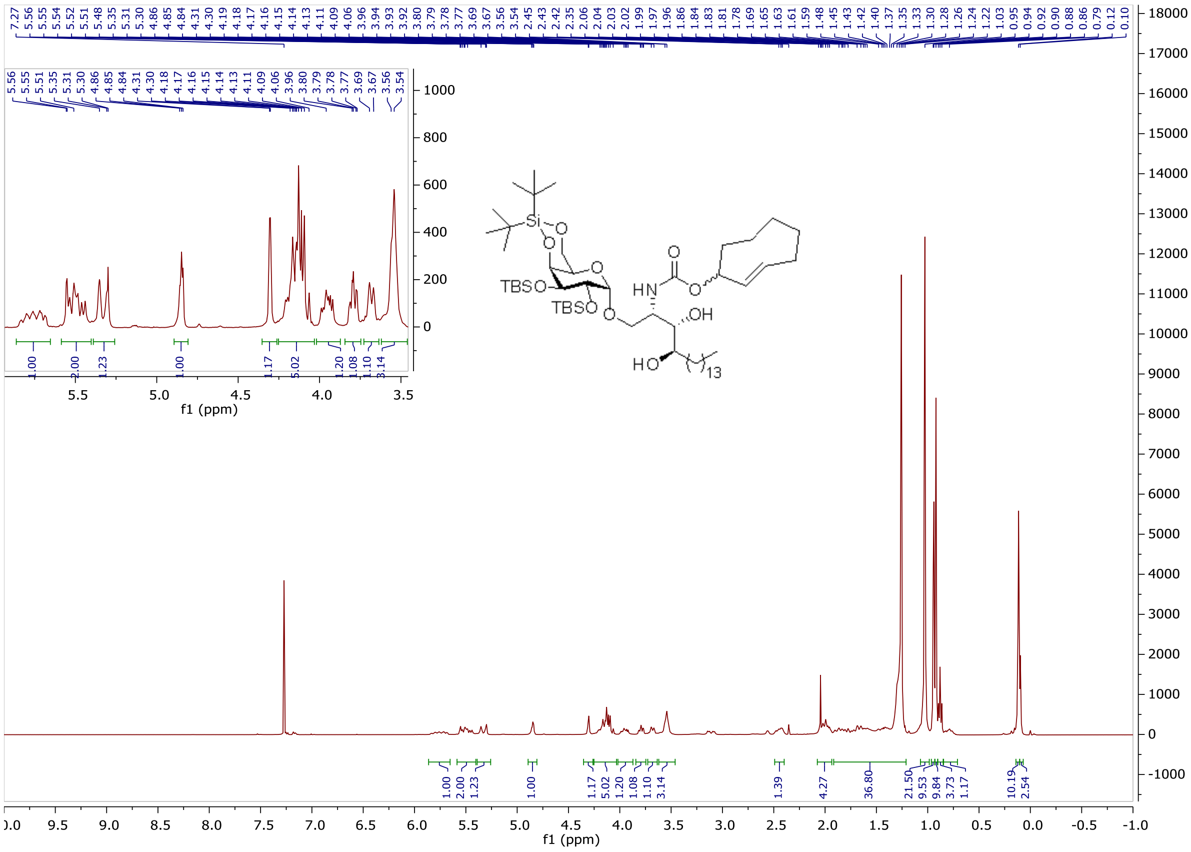

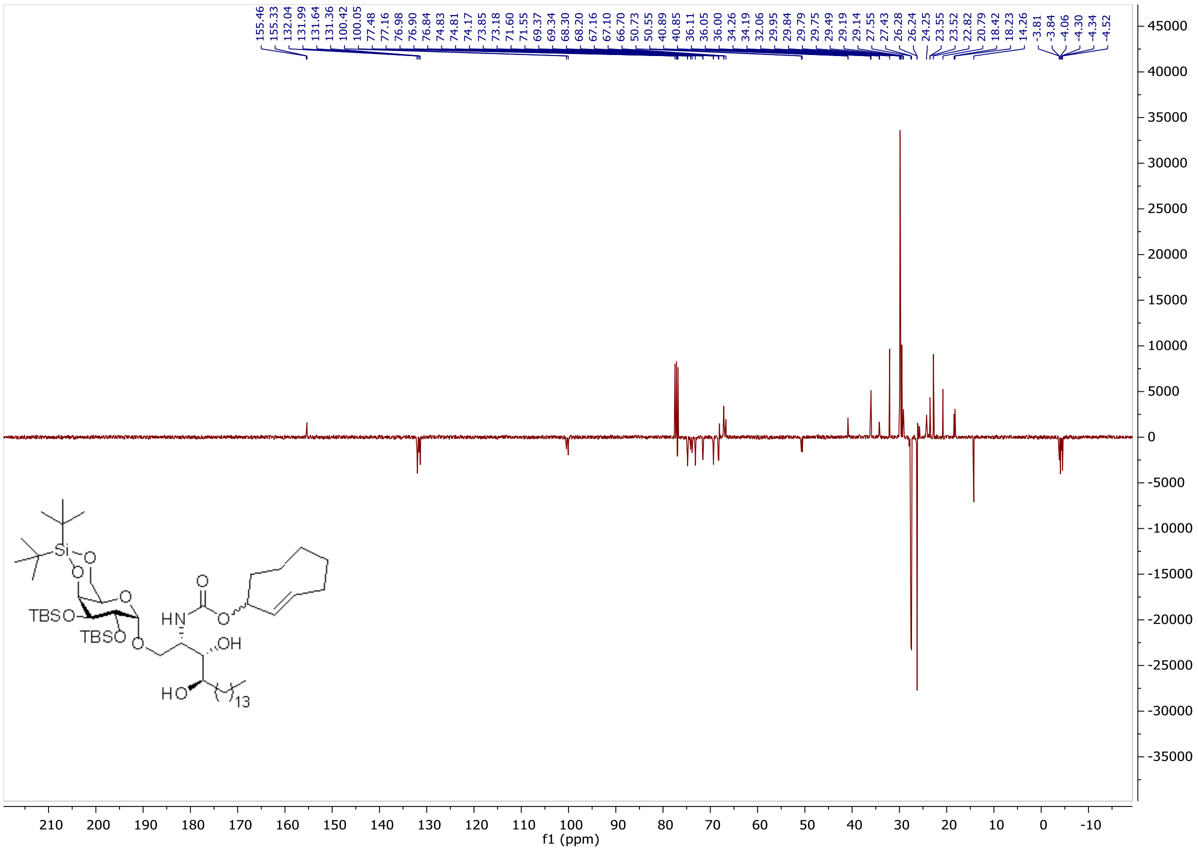

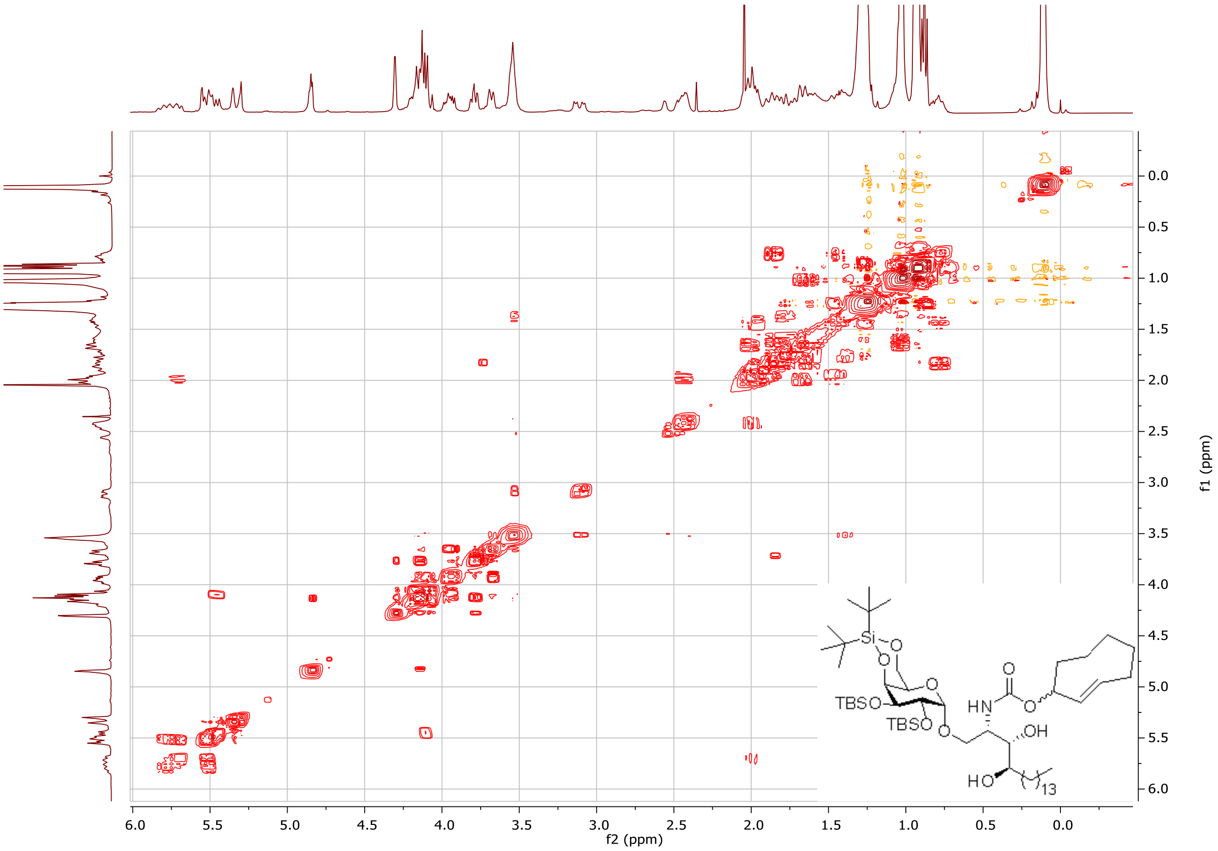
6.0
5.5
5.0
4.5
4.0
3.5
3.0
2.5
2.0
1.5
1.0
0.5
0.0
f2 (ppm)
f1 (ppm)
TBSO
TBSO
HN
OH
HO
13
Si

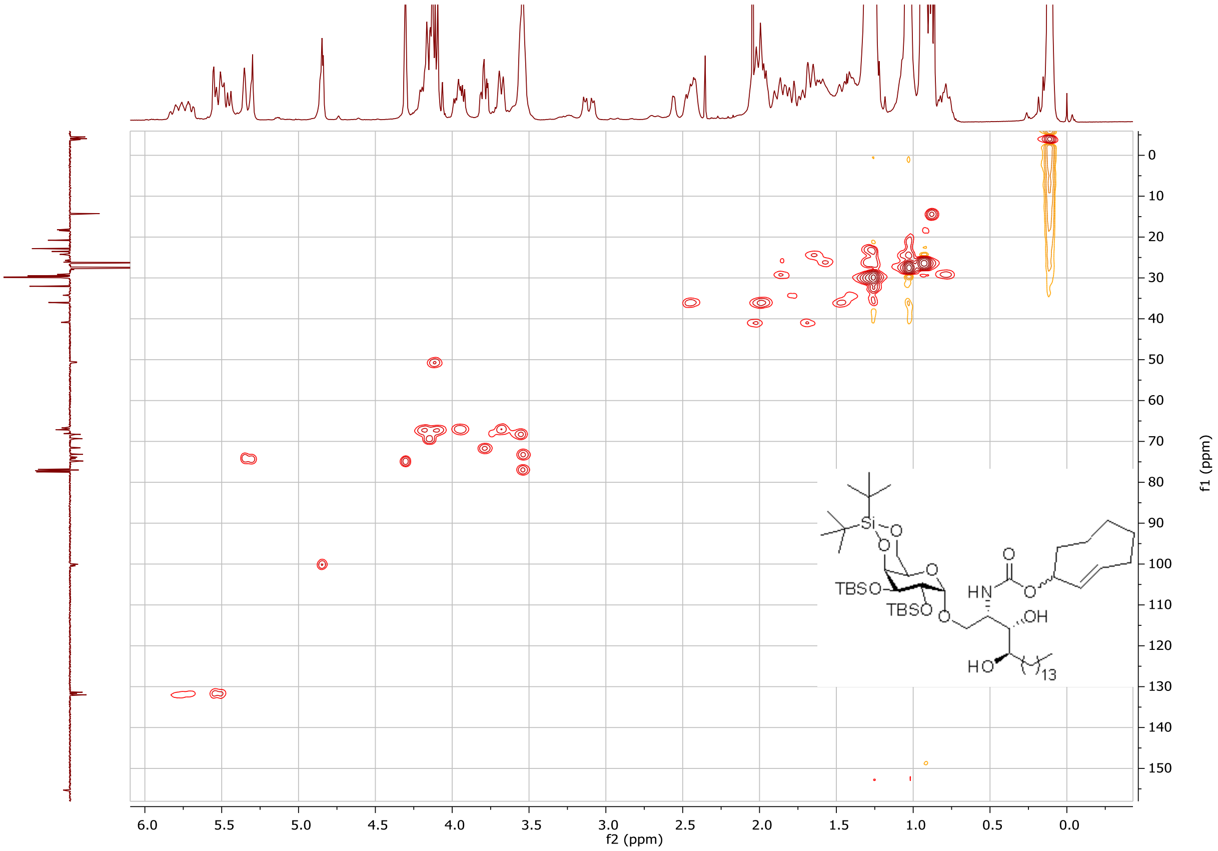
6.0
5.5
5.0
4.5
4.0
3.5
3.0
2.5
2.0
1.5
1.0
0.5
0.0
f2 (ppm)
f1 (ppm)
0
10
20
30
40
50
60
70
80
90
100
110
120
130
140
150
TBSO
TBSO
HN
OH
HO
13
Si

^{1}H, ^{13}C APT, ^{1}H COSY and HSQC spectra of 2 (Pyridine-d_5)

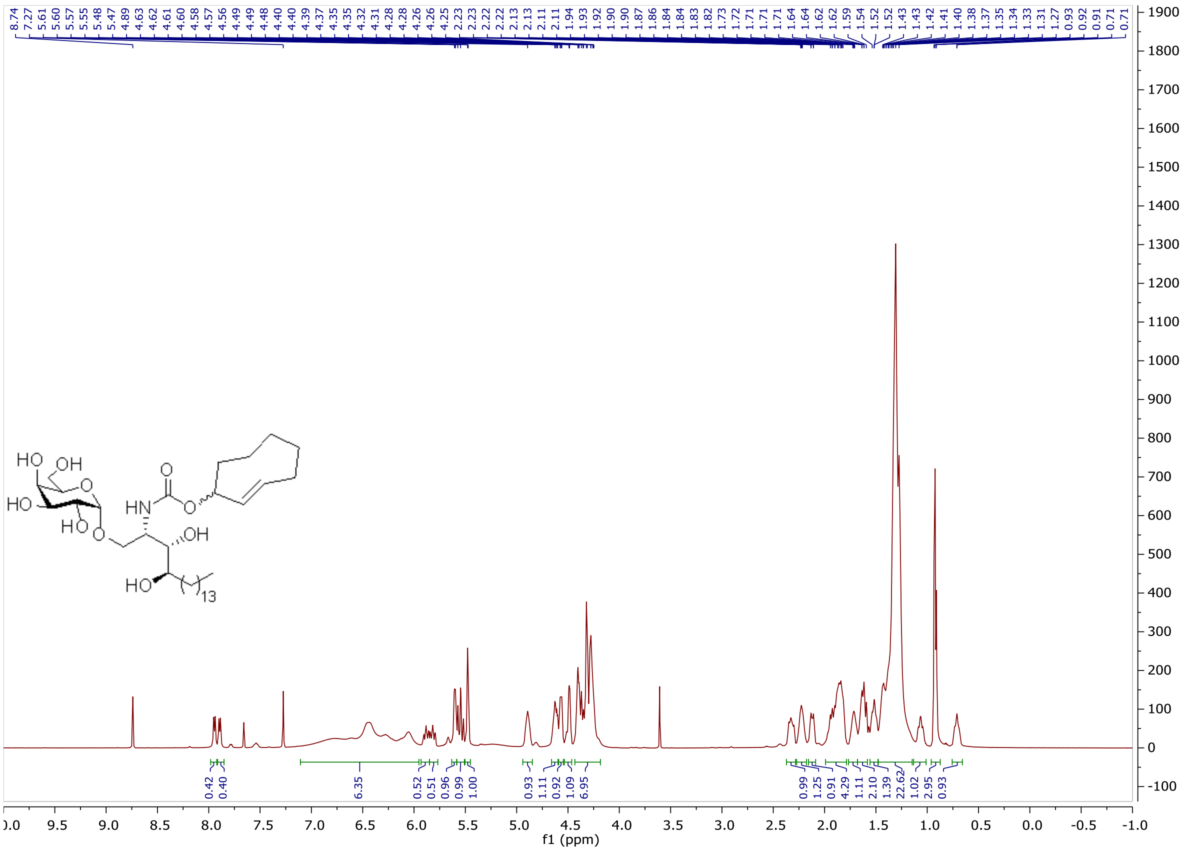

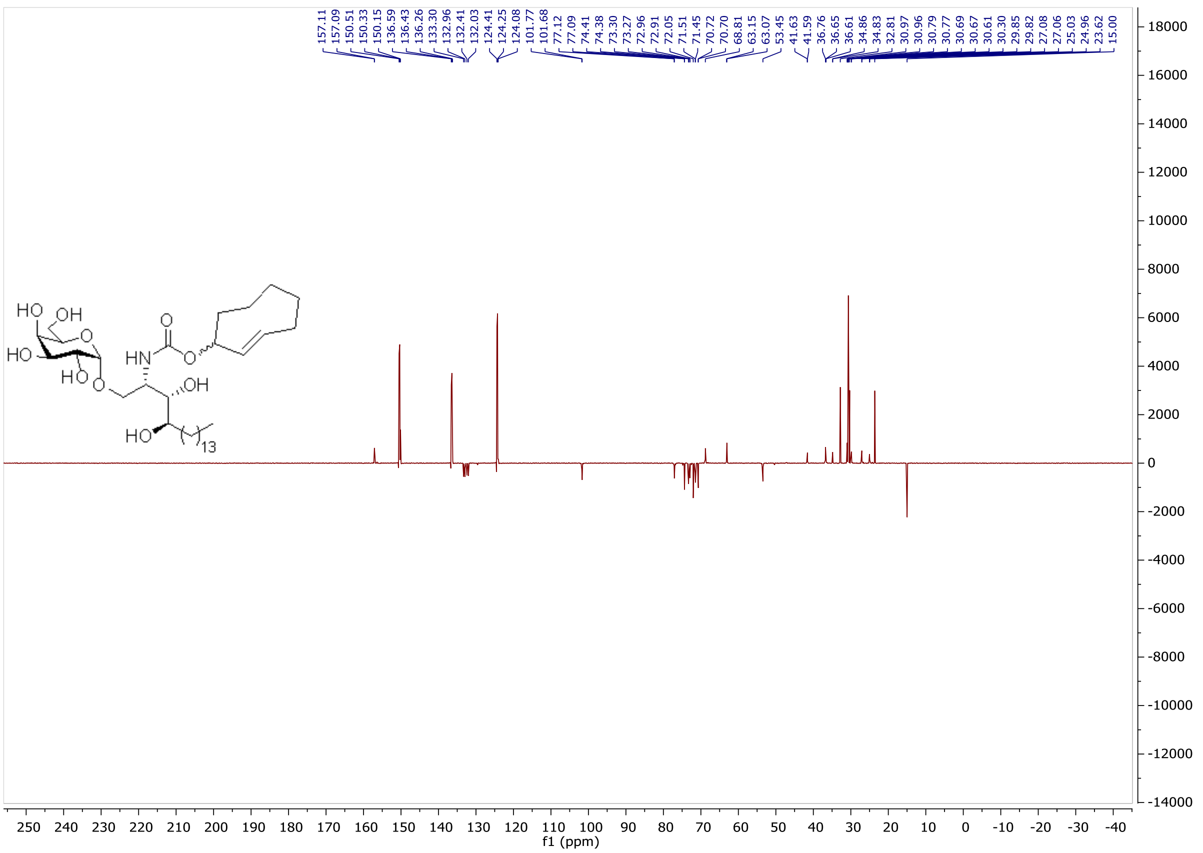

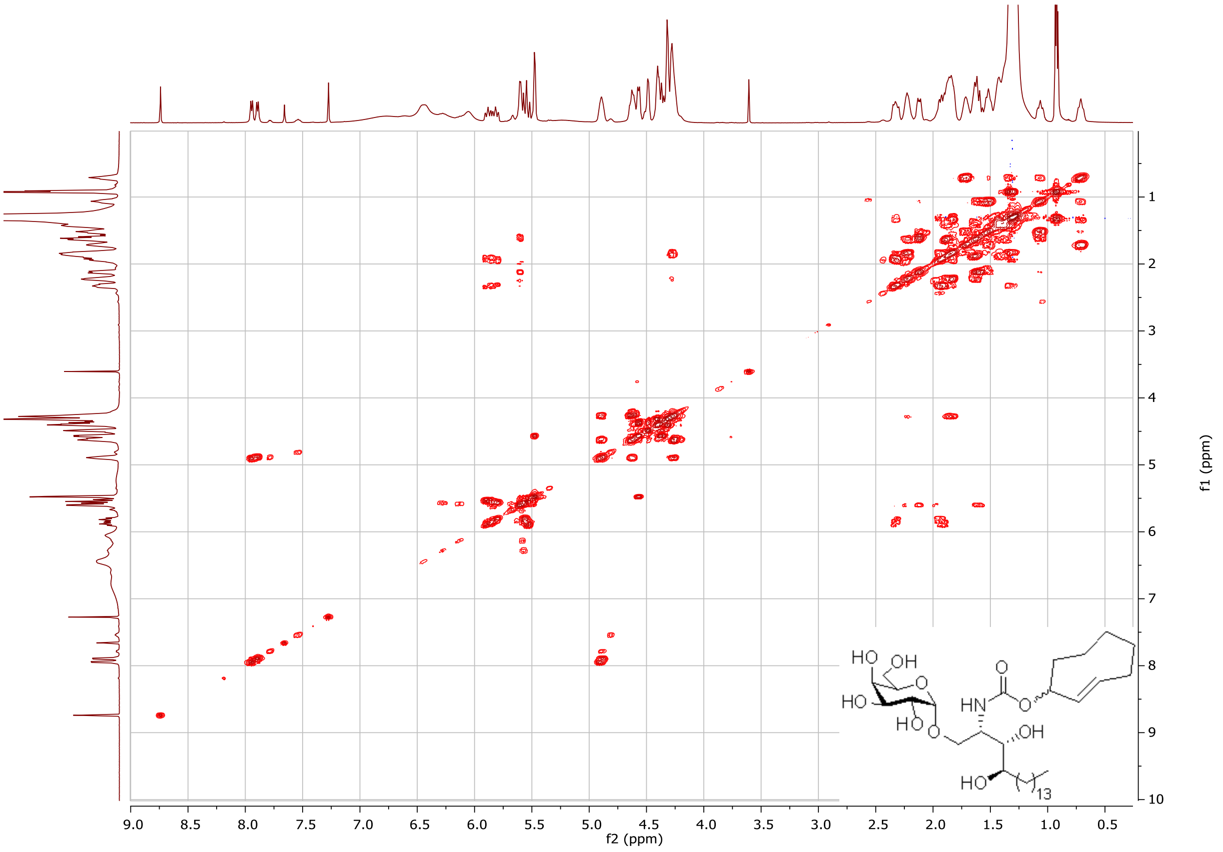
f1 (ppm)
f2 (ppm)
HO
OH
O
HO
HO
O
HN
O
O
OH
HO
13

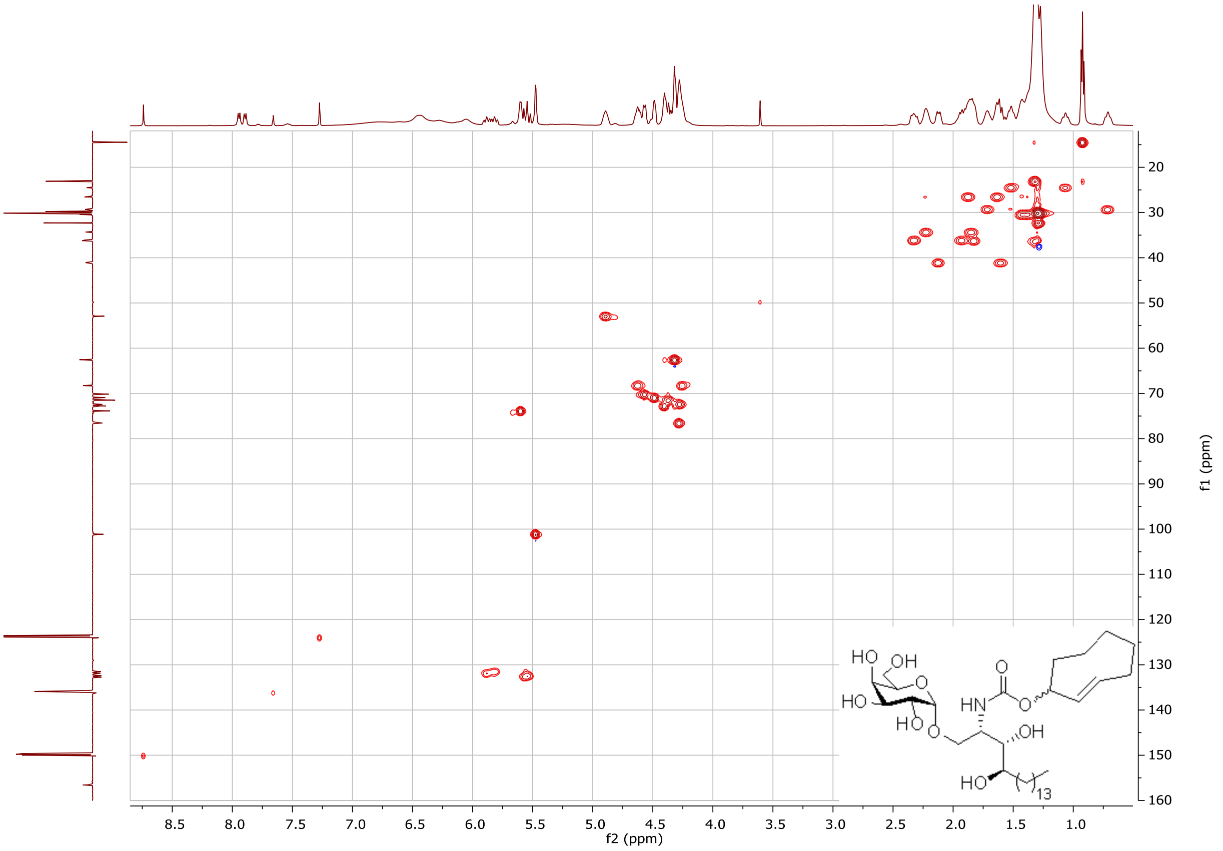
f1 (ppm)
f2 (ppm)
HO
OH
O
HO
HO
O
HN
O
O
OH
HO
13

^{1}H, ^{13}C APT, ^{1}H COSY and HSQC spectra of 2 (Dioxane-d_8)

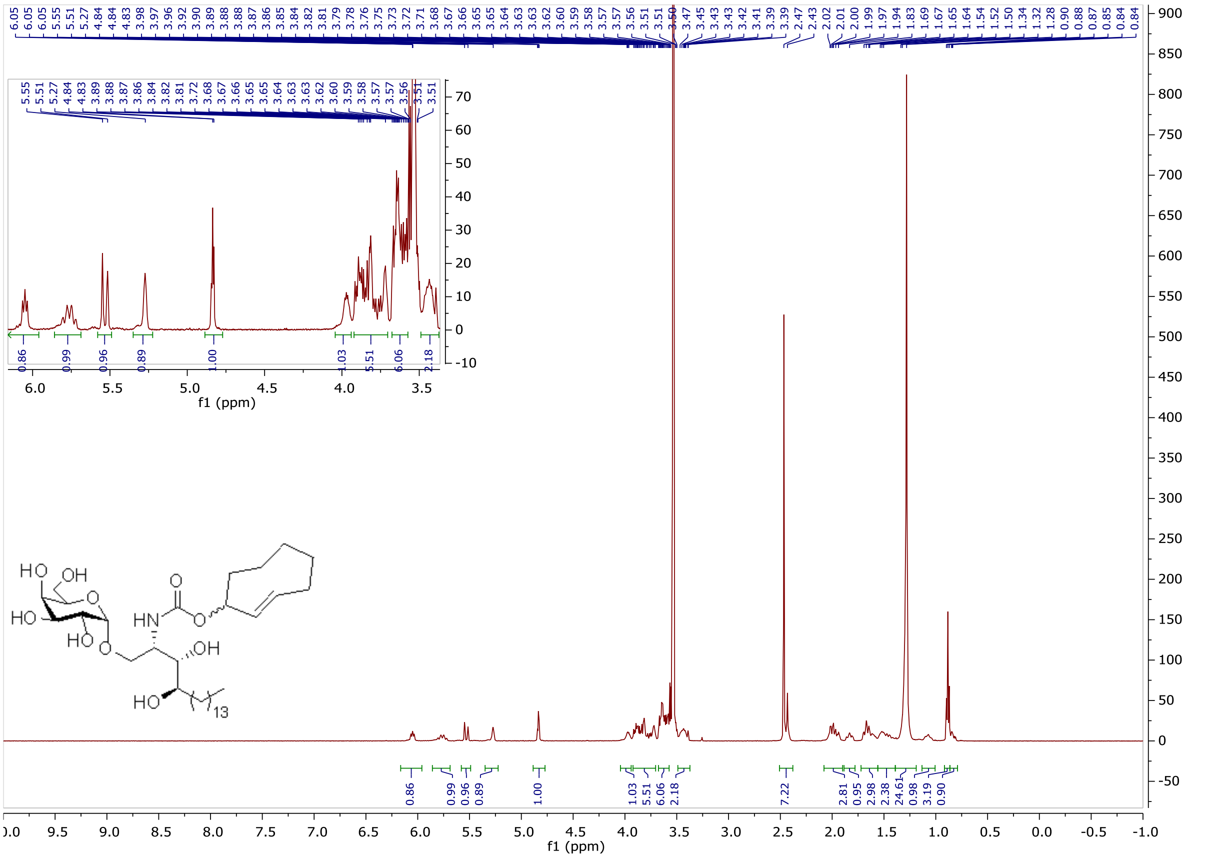

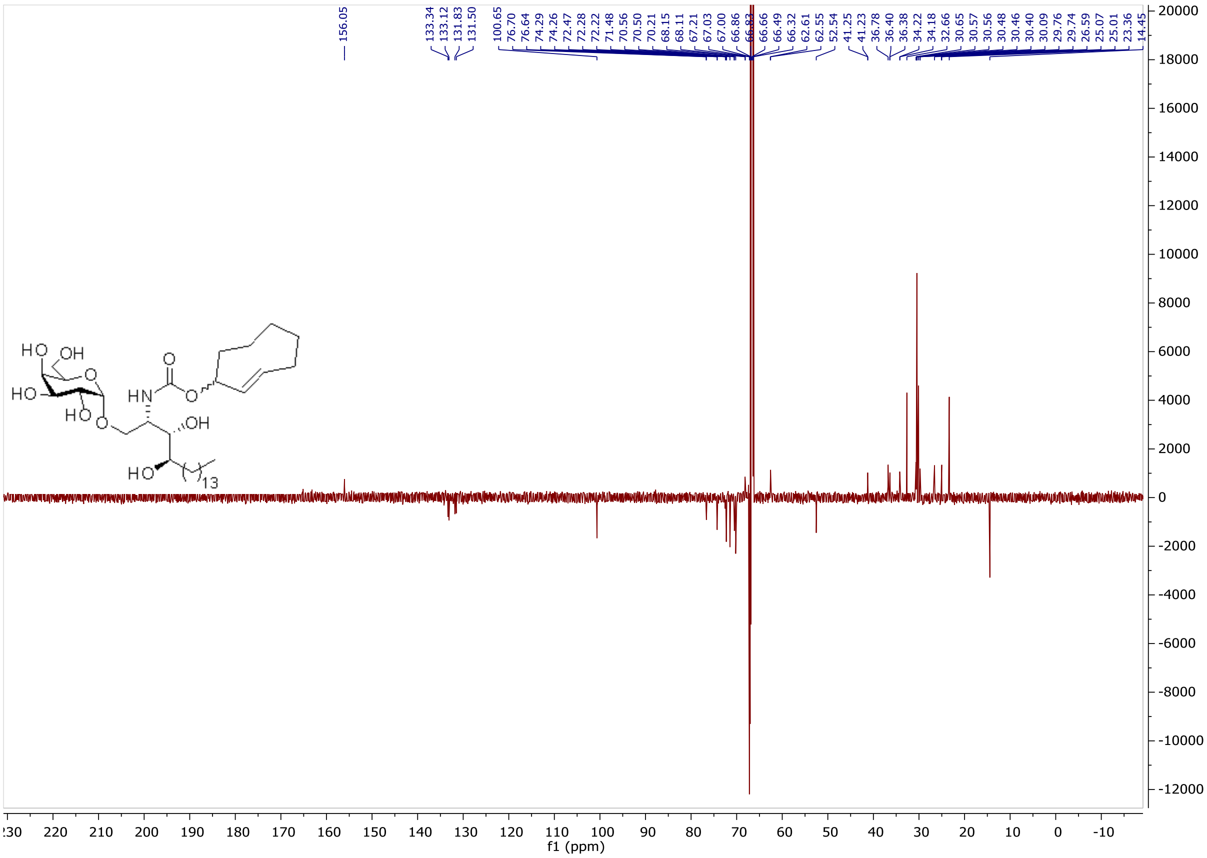

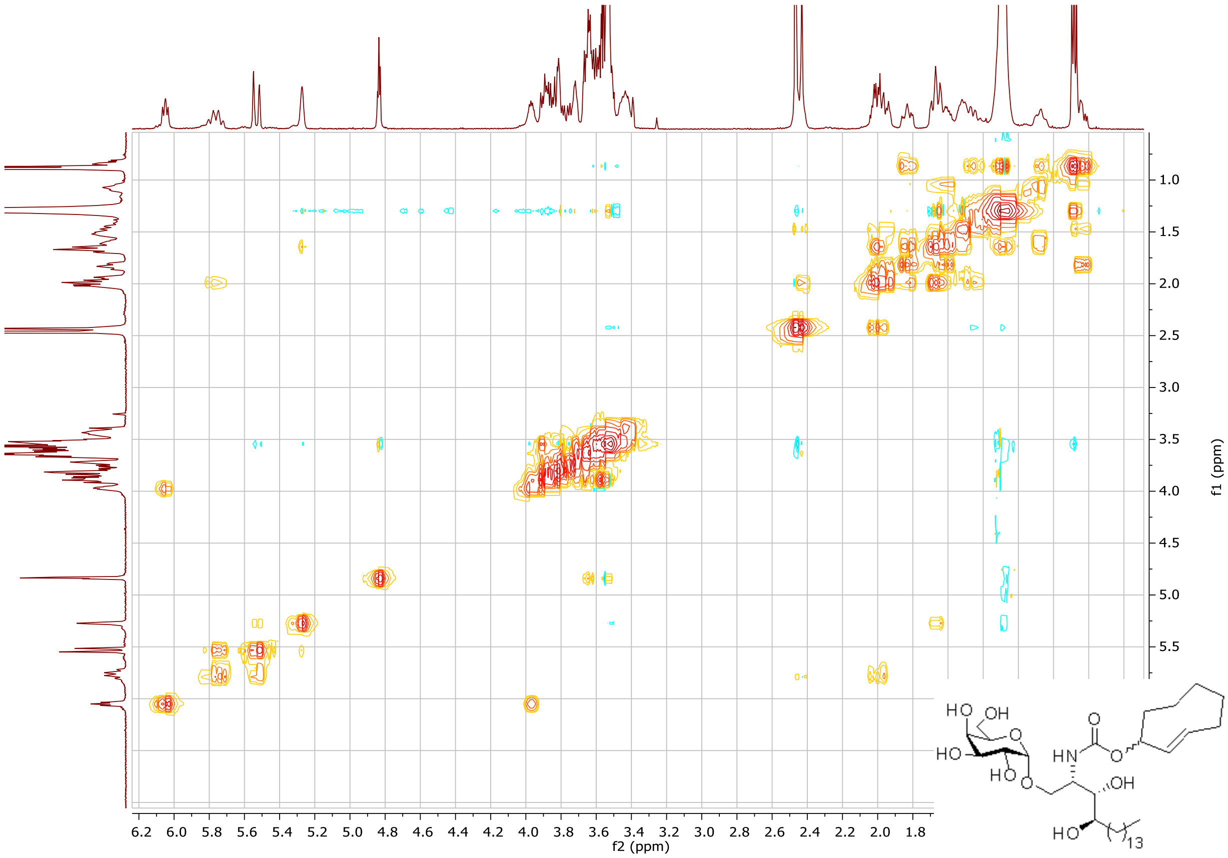
f2 (ppm)
f1 (ppm)

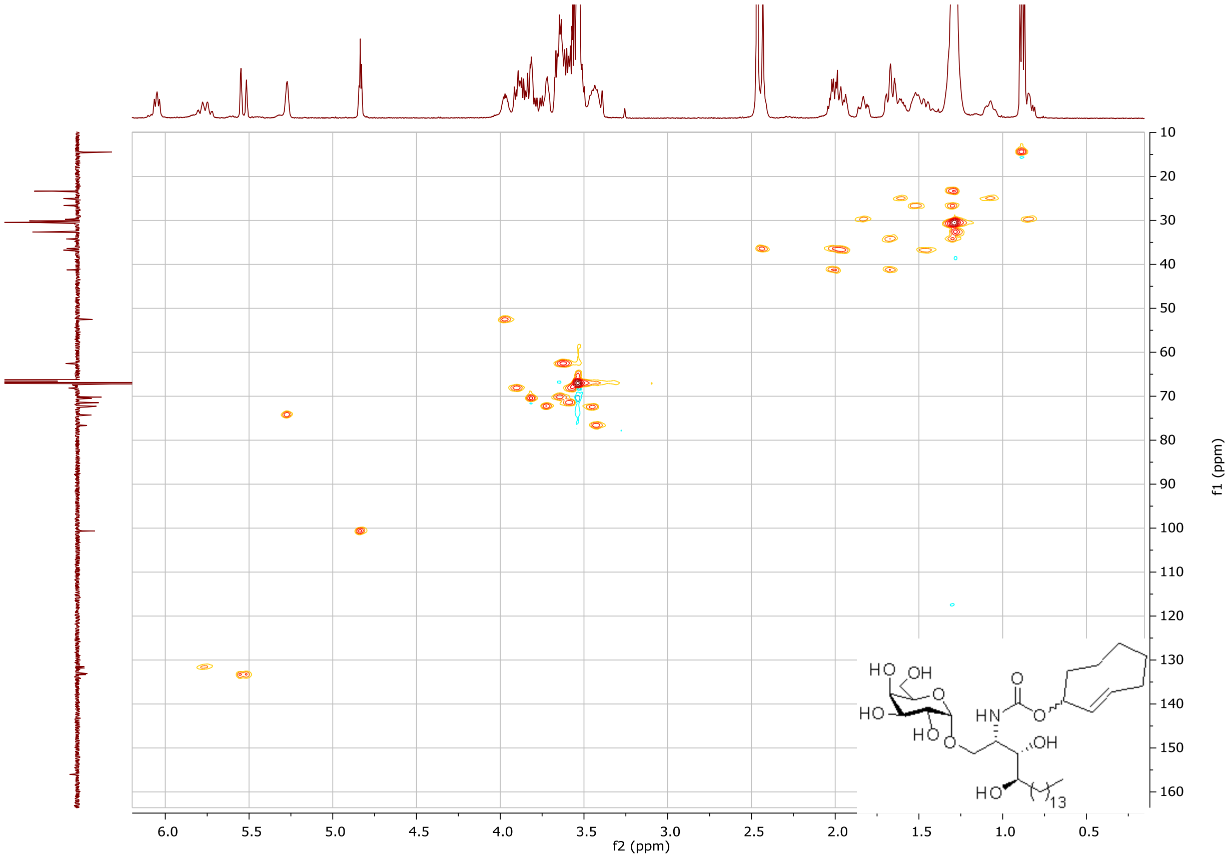
f2 (ppm)
f1 (ppm)

^{1}H, ^{13}C APT, ^{1}H COSY and HSQC spectra of 18

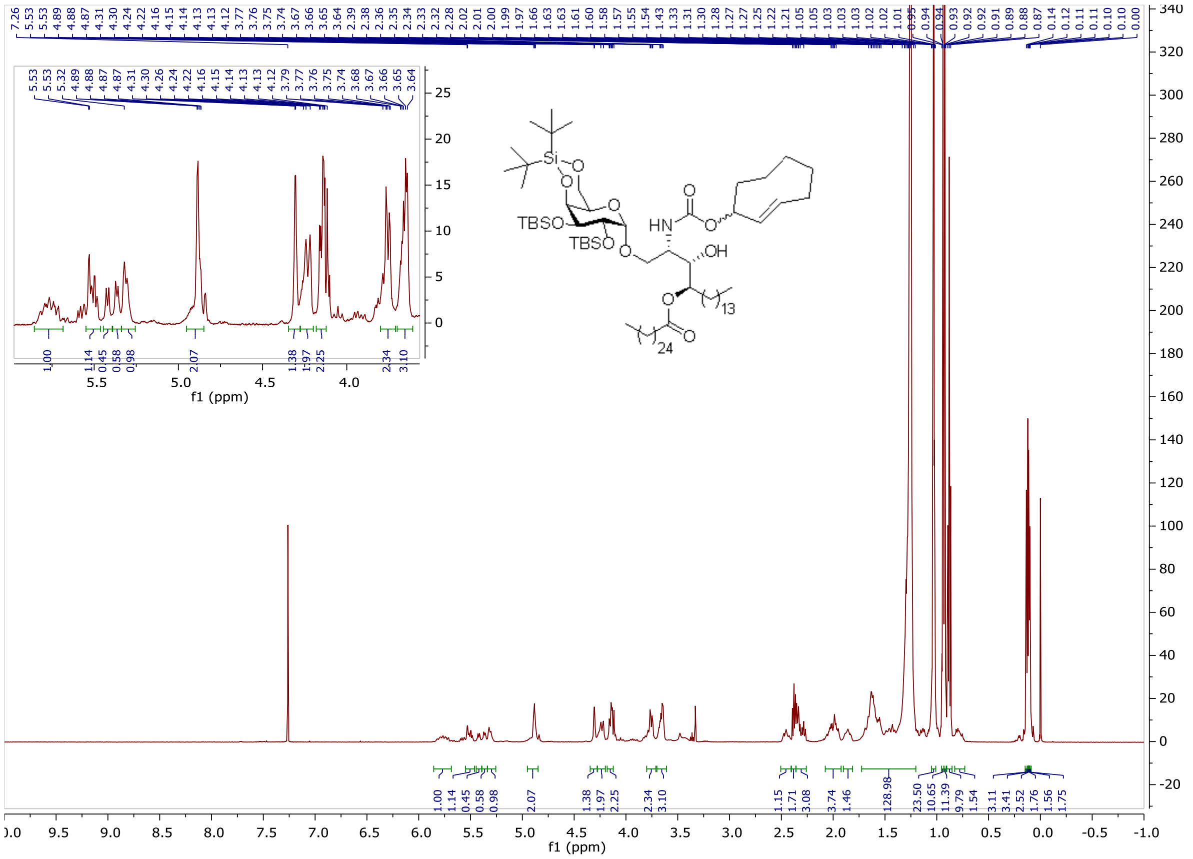

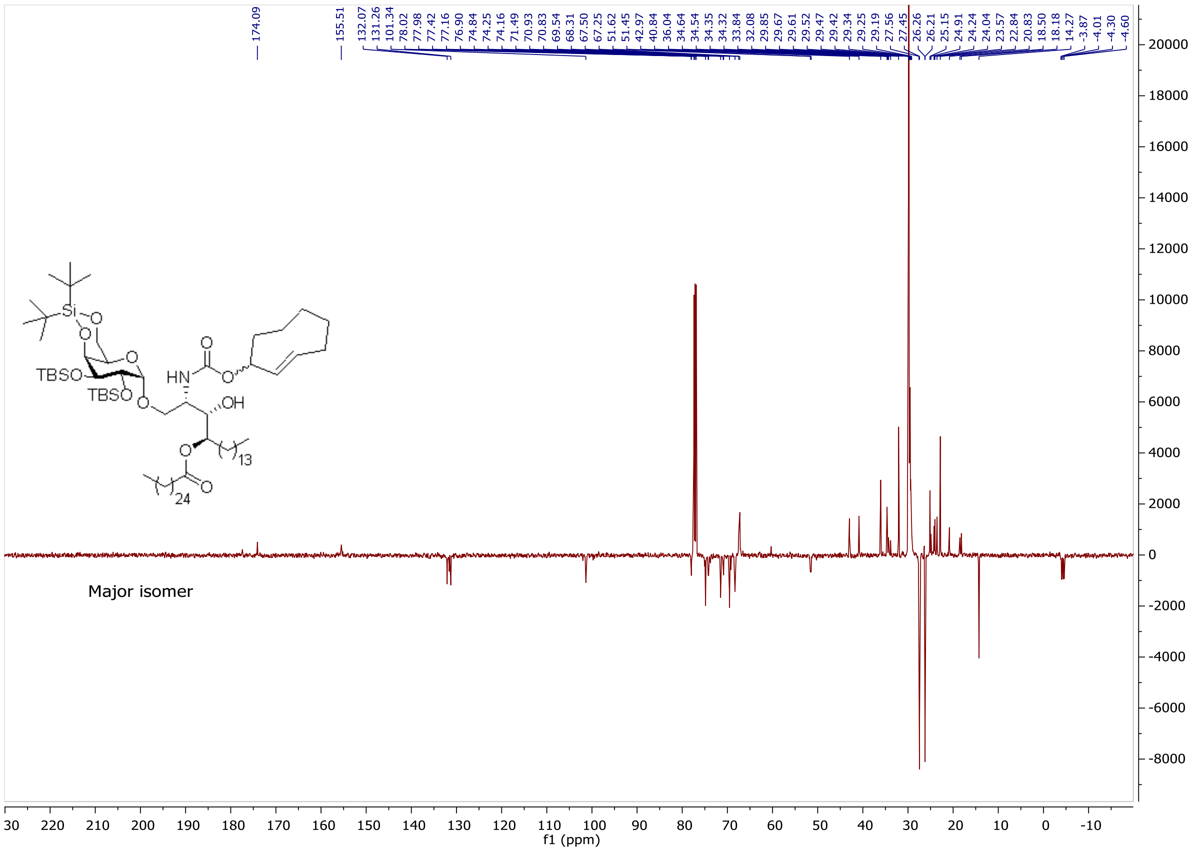
174.09
155.51
132.07
131.26
101.34
78.02
77.98
77.42
77.16
76.90
74.84
74.25
74.16
71.49
70.93
70.83
69.54
68.31
67.50
67.25
51.62
51.45
42.97
40.84
36.04
34.64
34.54
34.35
34.32
33.84
32.08
29.85
29.67
29.61
29.52
29.47
29.42
29.34
29.25
29.19
27.56
27.45
26.26
26.21
25.15
24.91
24.24
24.04
23.57
22.84
20.83
18.50
18.18
14.27
-3.87
-4.01
-4.30
-4.60
TBSO
TBSO
HN
OH
13
24
Major isomer
f1 (ppm)

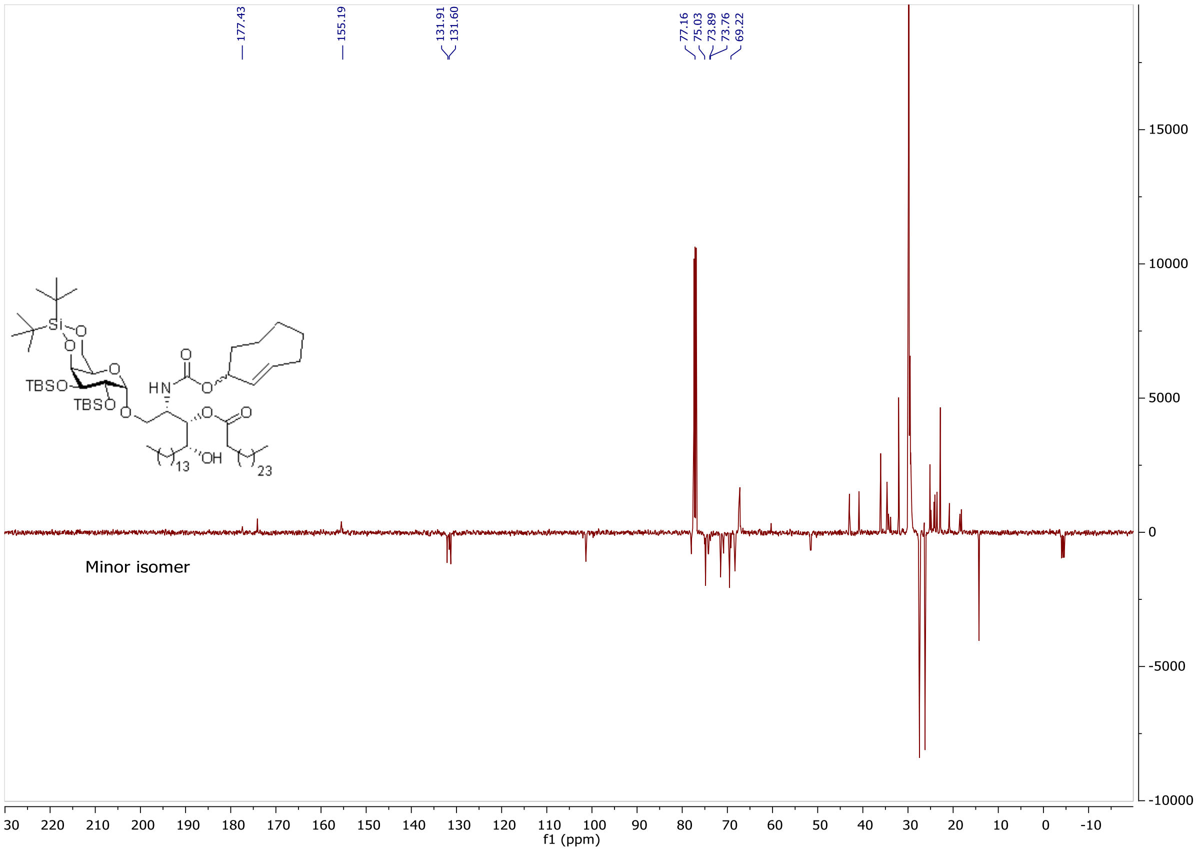
177.43
155.19
131.91
131.60
77.16
75.03
73.89
73.76
69.22
TBSO
TBSO
HN
OH
13
23
Minor isomer
f1 (ppm)

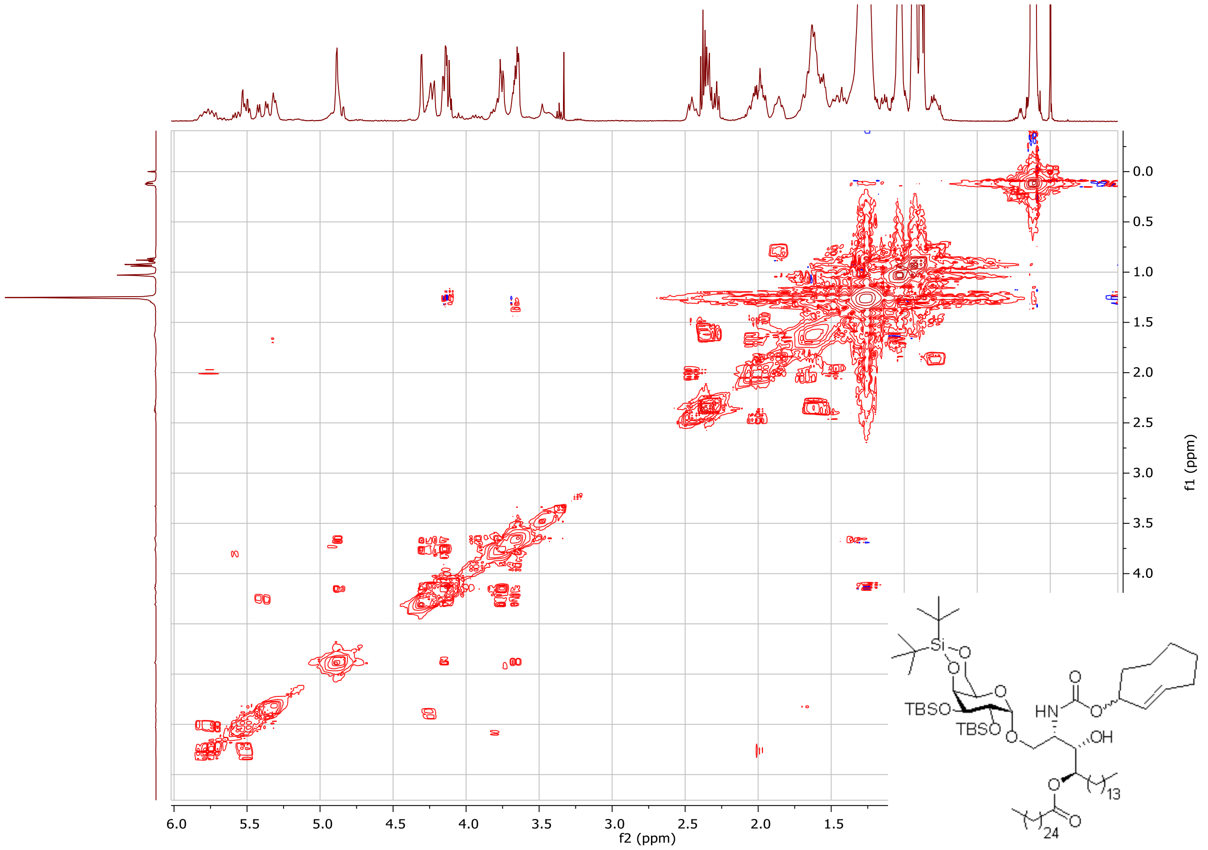
f1 (ppm)
f2 (ppm)
TBSO
TBSO
HN
OH
Si
13
24

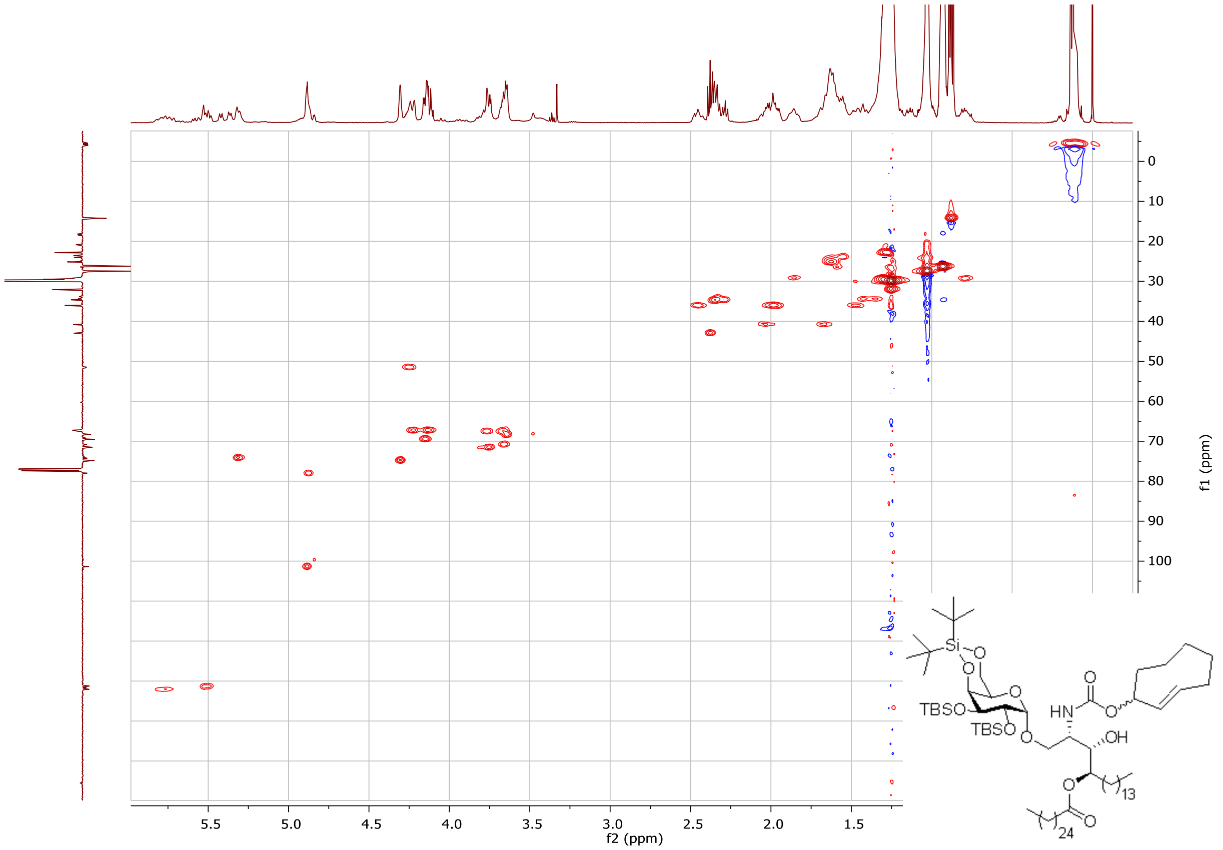
f1 (ppm)
f2 (ppm)
TBSO
TBSO
HN
OH
Si
13
24

^1H, ^{13}C APT, ^1H COSY and HSQC spectra of 1

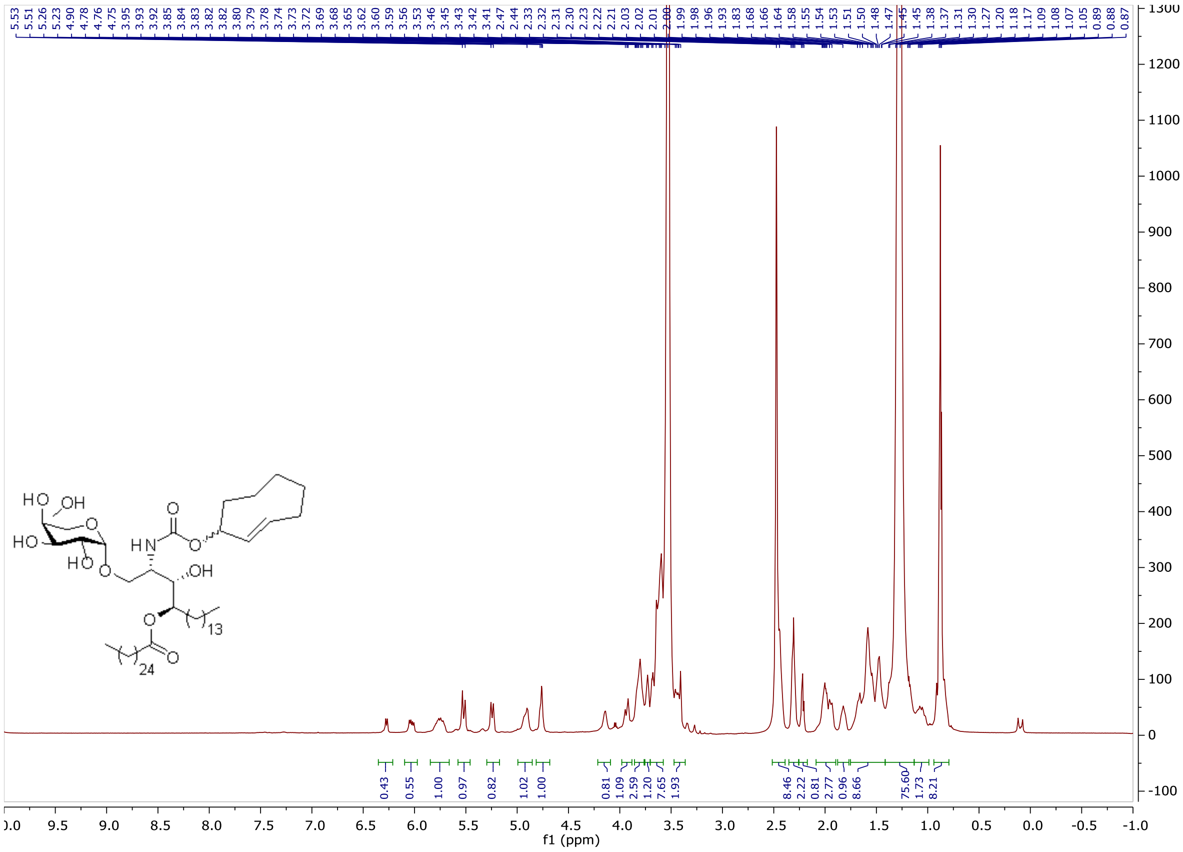

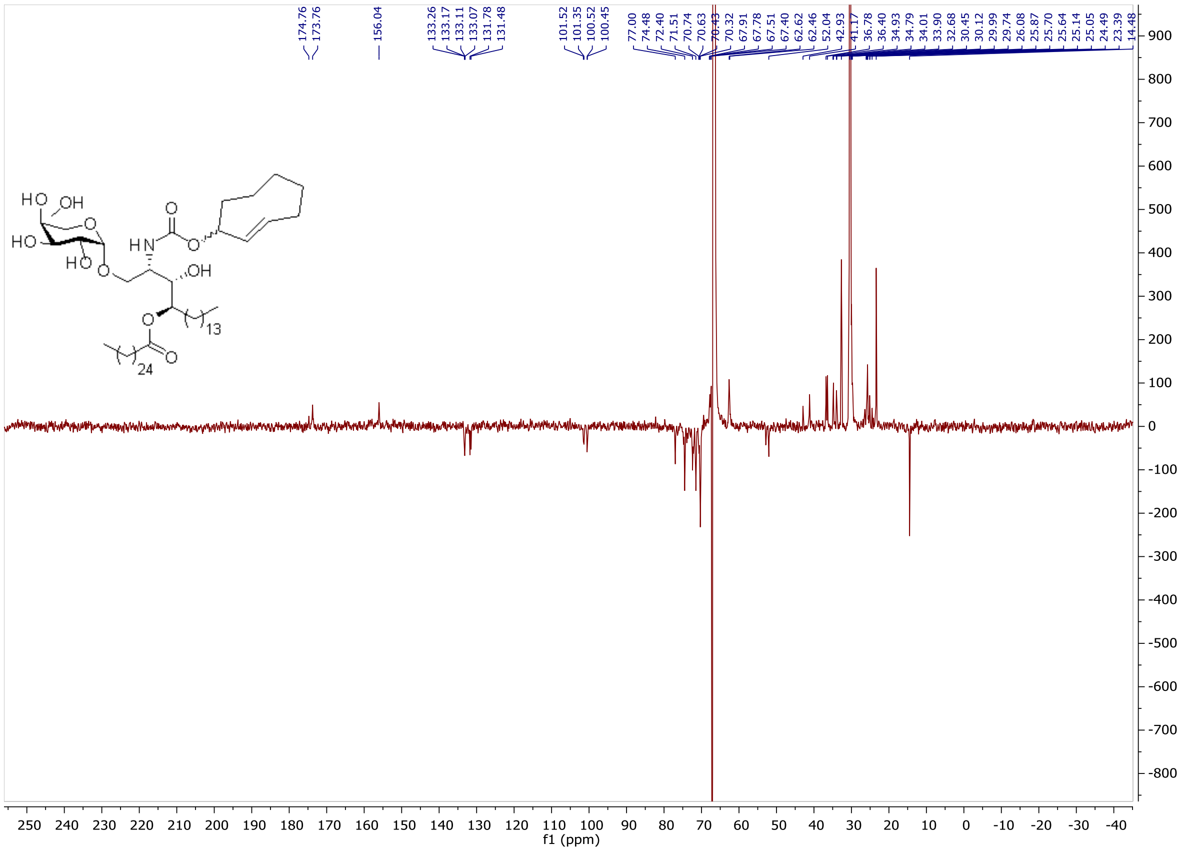

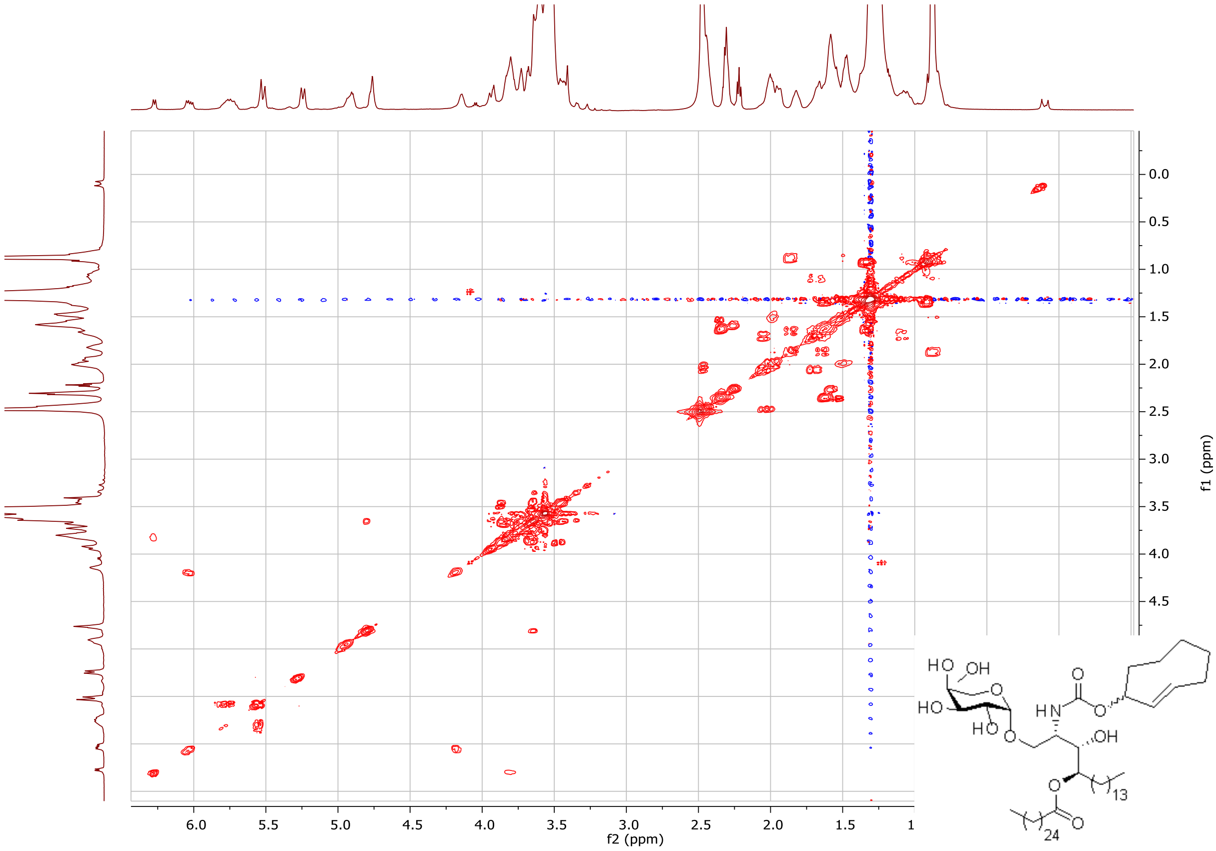

f2 (ppm)
f1 (ppm)
HO
OH
O
HO
HO
O
HN
O
O
OH
O
13
24
O

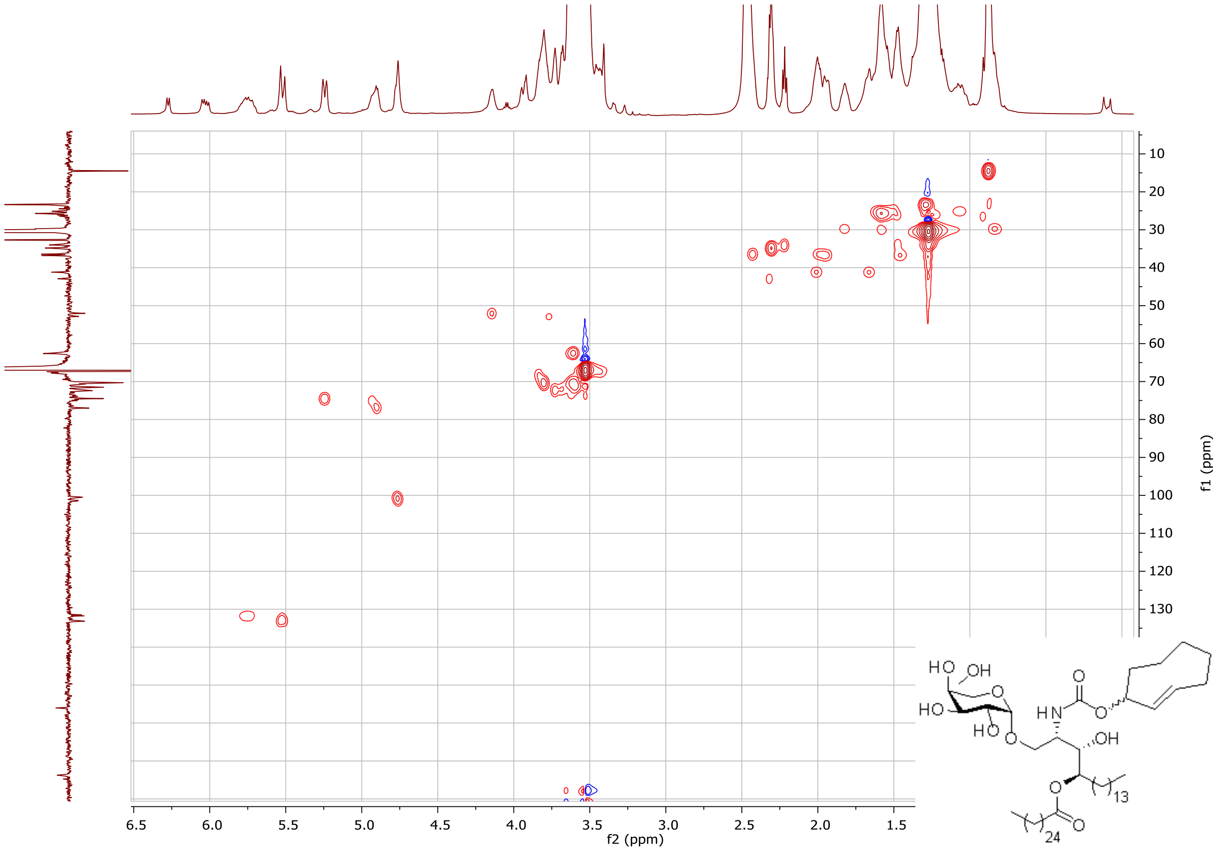

f2 (ppm)
f1 (ppm)
HO
OH
O
HO
HO
O
HN
O
O
OH
O
13
24
O

6. LC-MS spectra

LC-MS spectrum of BODIPY-TCO (10)

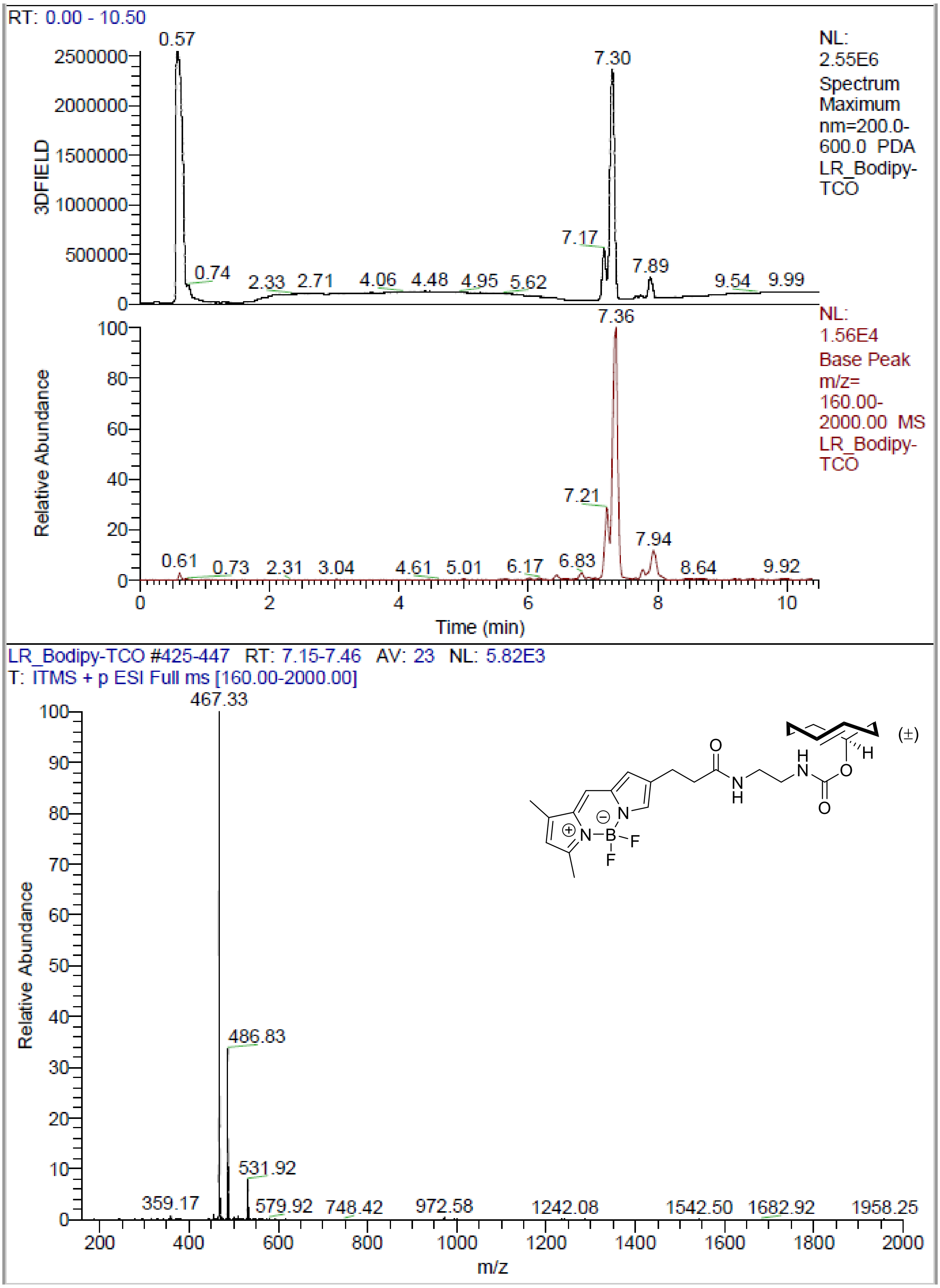

LC-MS spectrum of BODIPY-TCO-DABCYL (9)

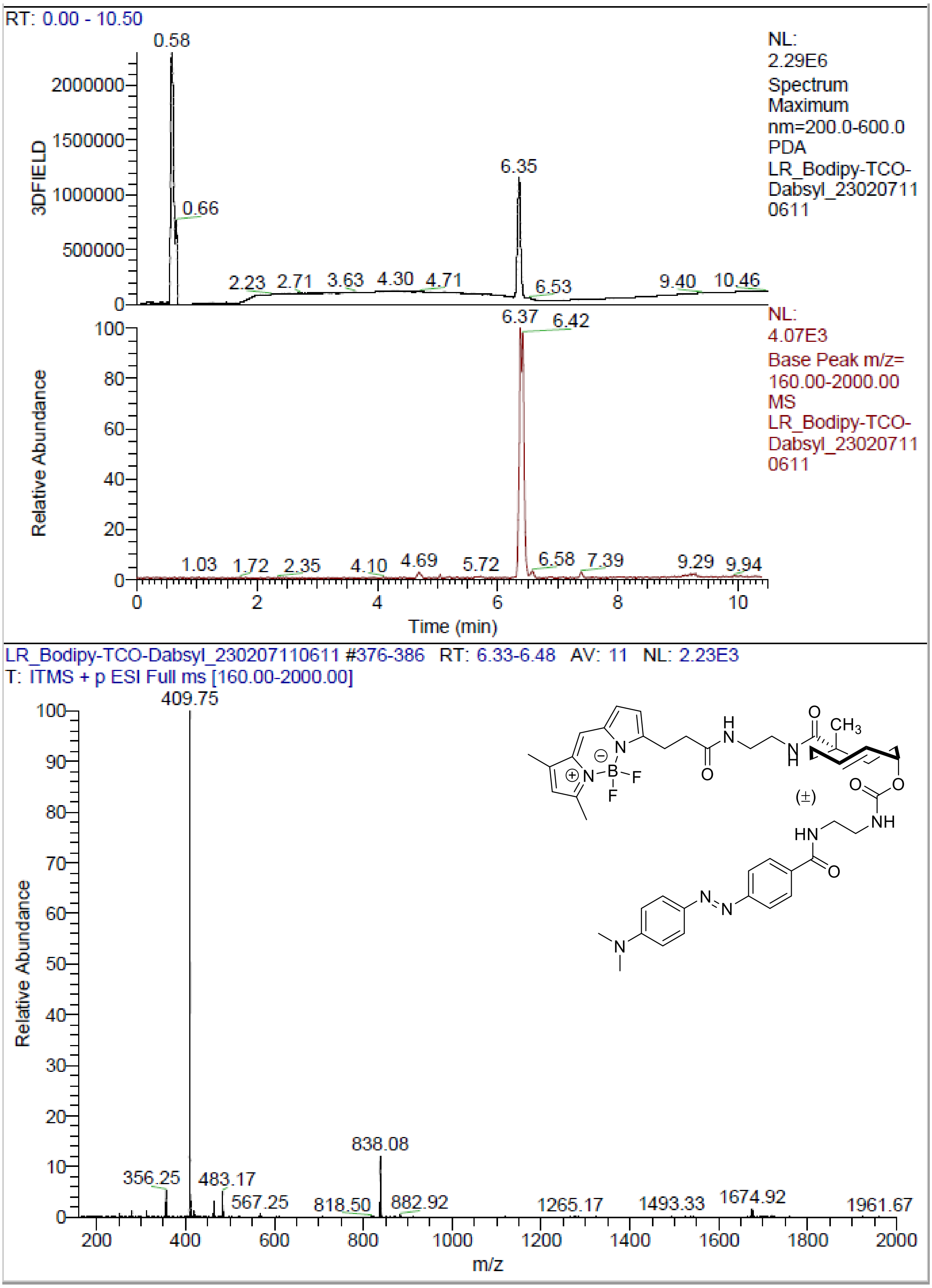

LC/MS analysis of 44

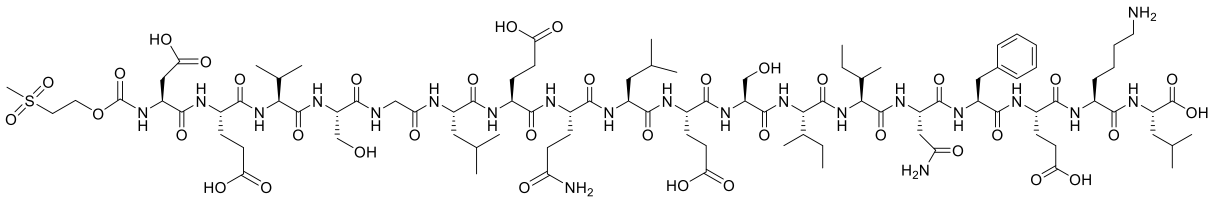

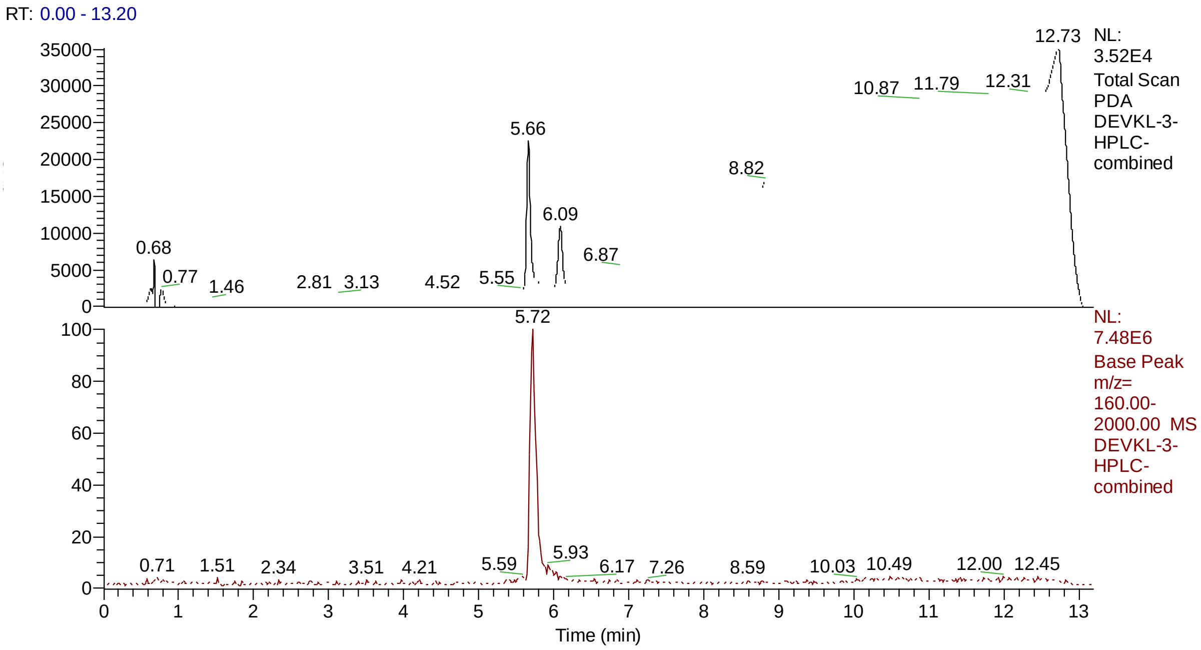

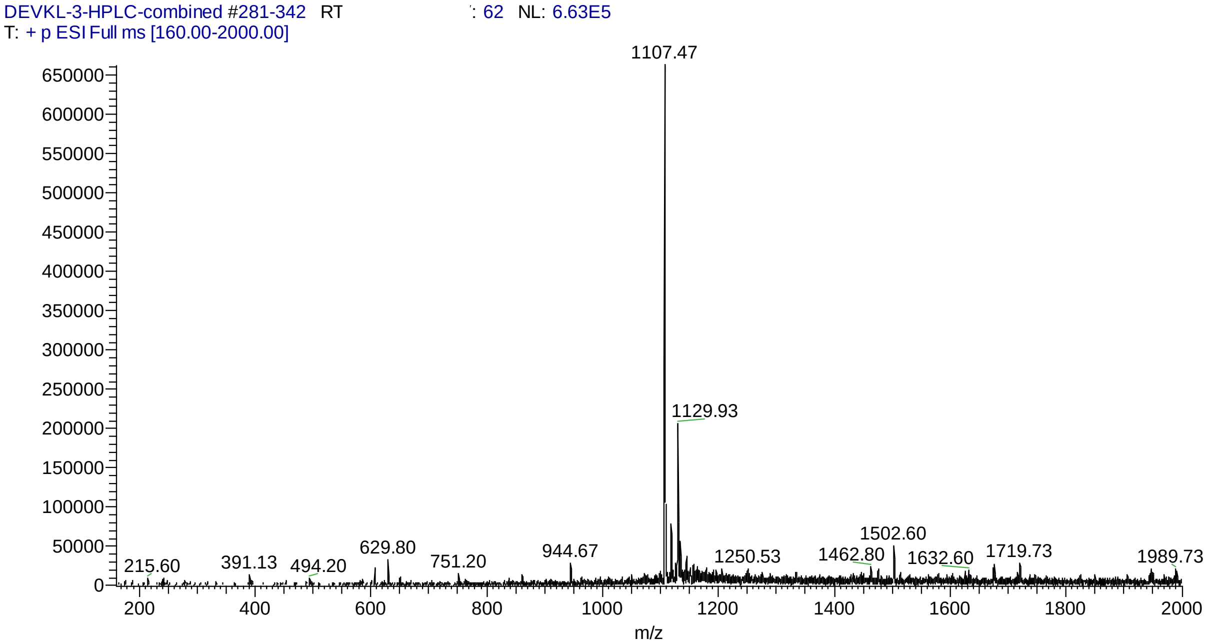

LC/MS analysis of 46

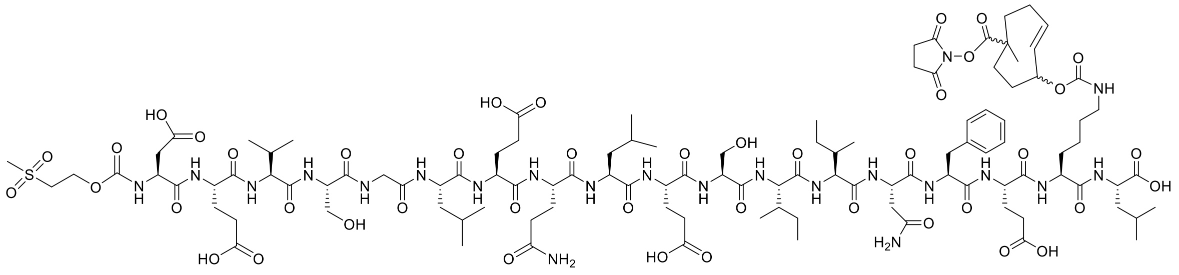

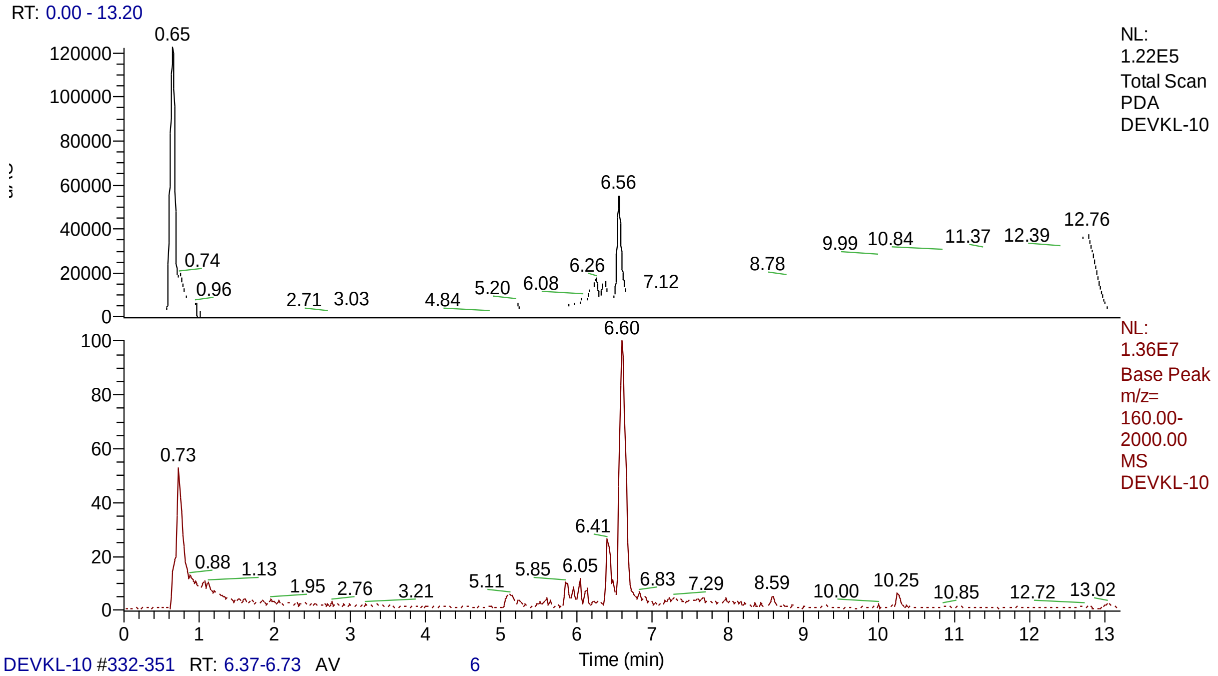

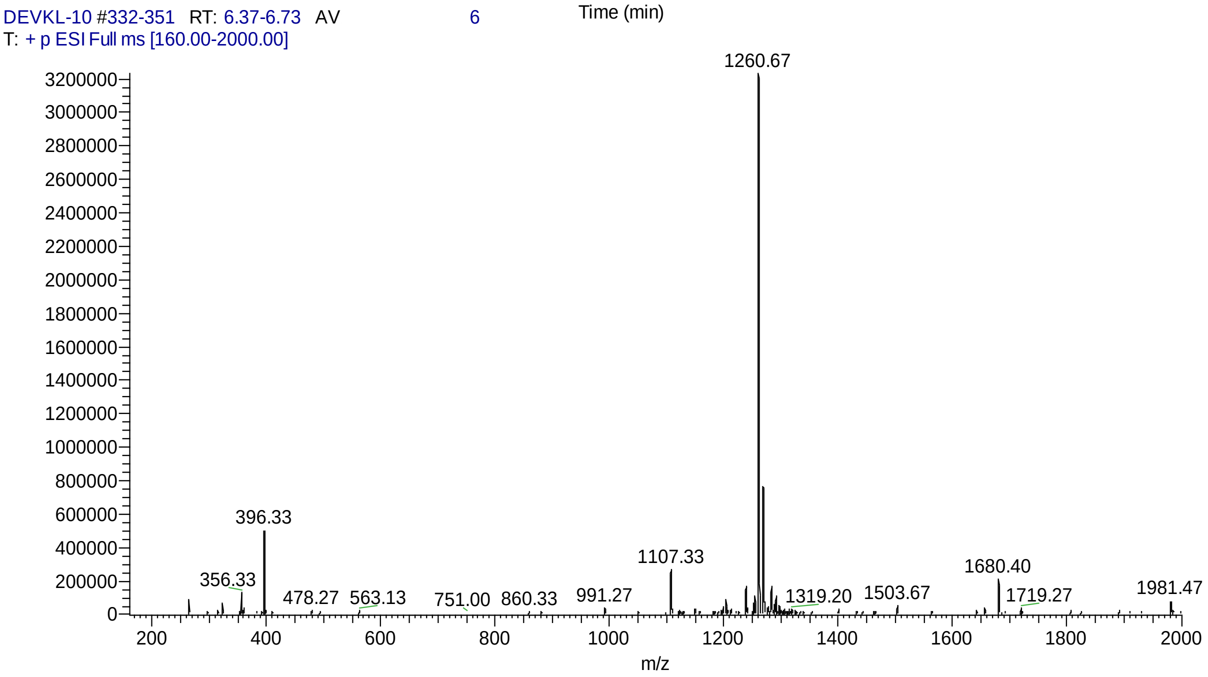

LC/MS analysis of 47

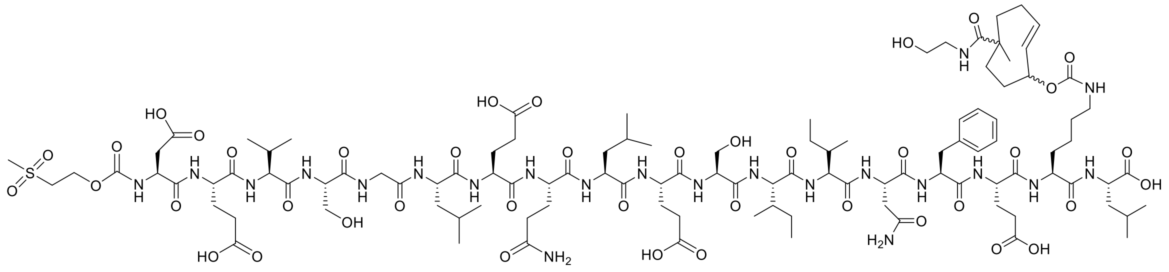

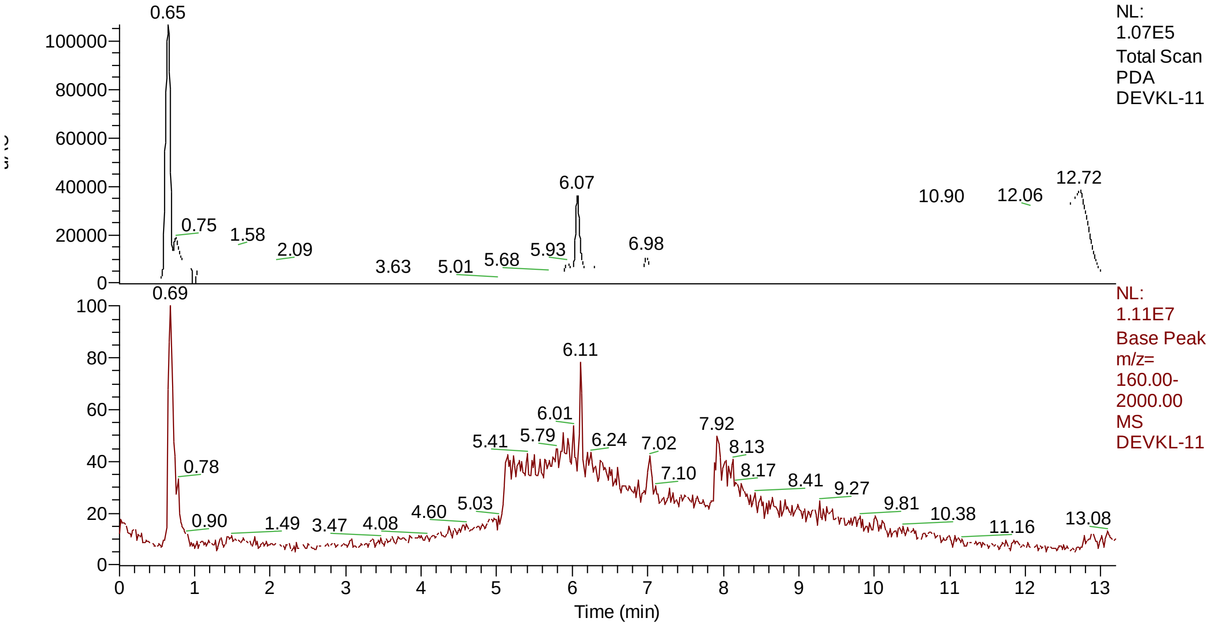

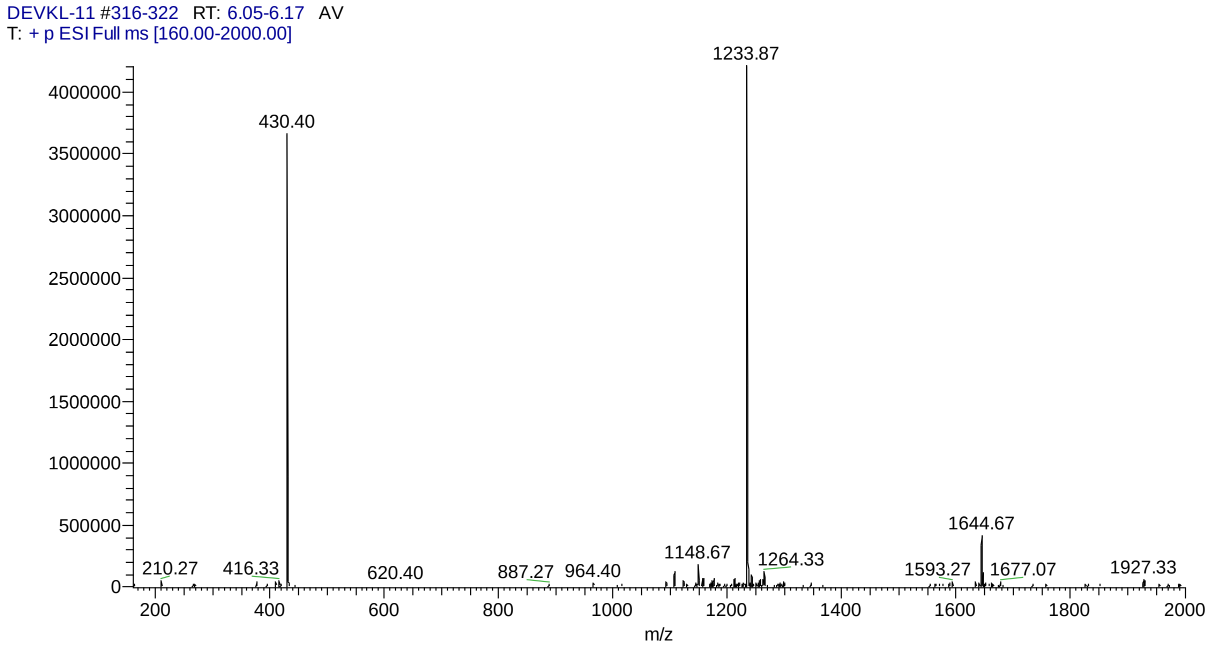

LC/MS analysis of 6

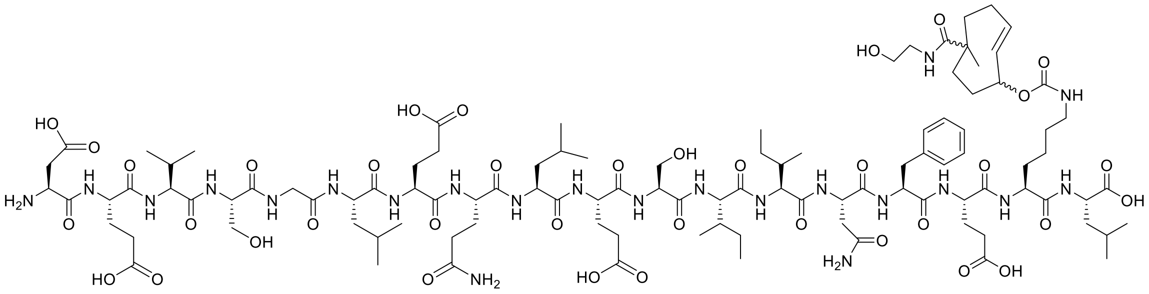

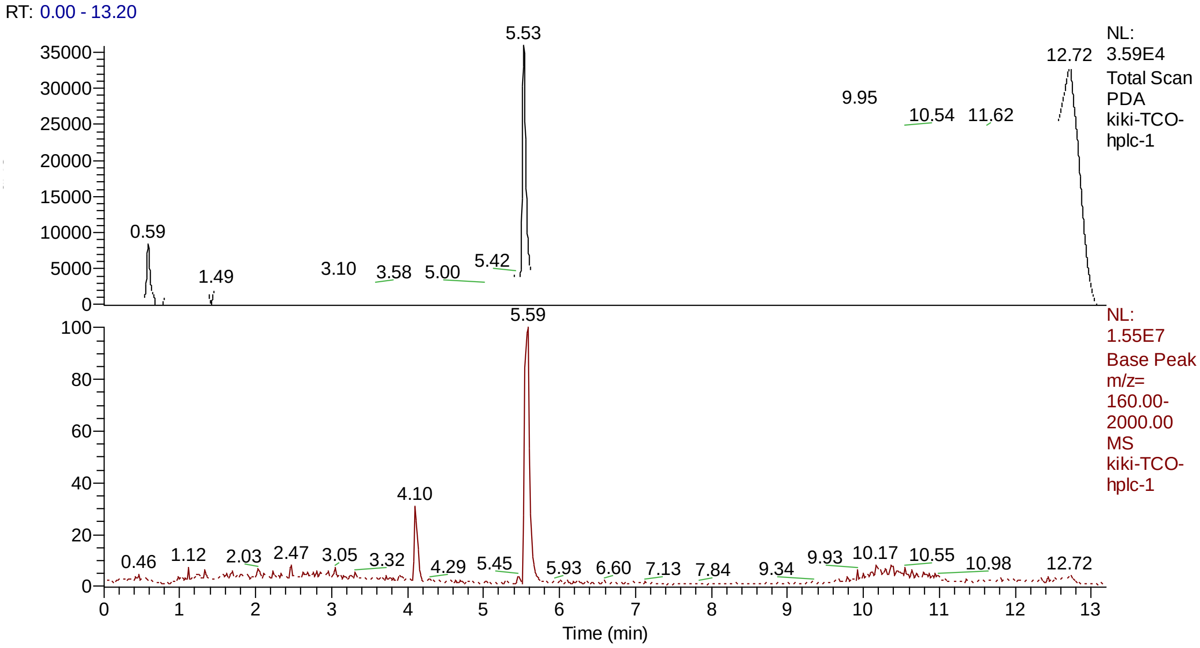

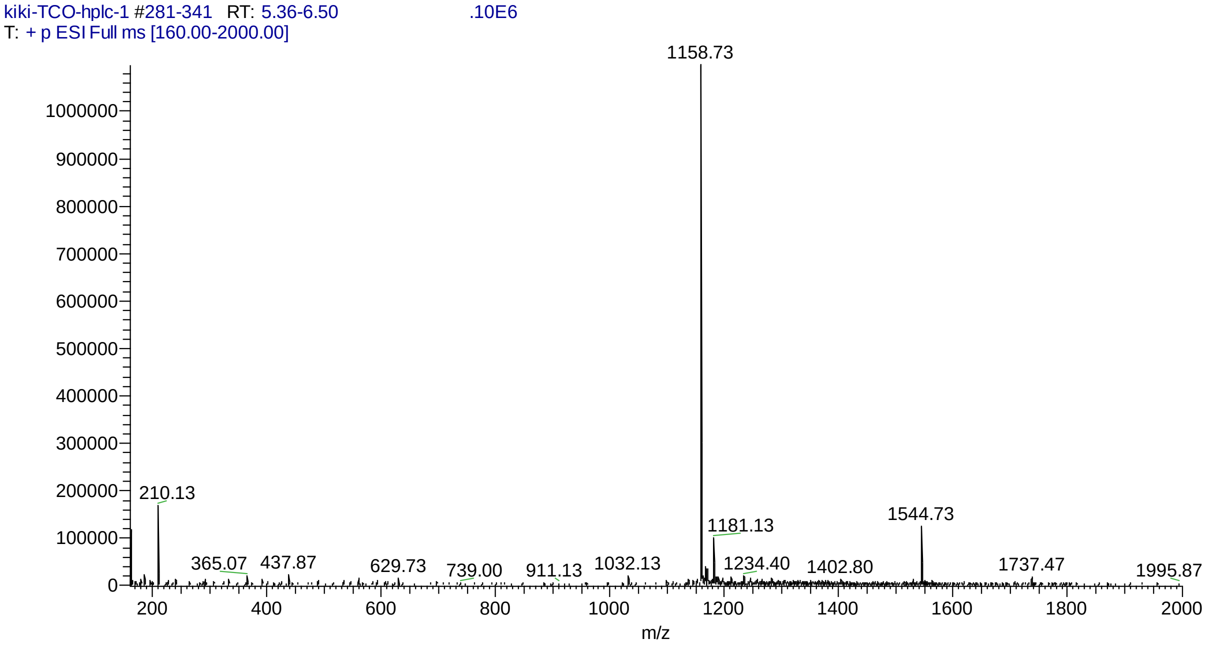

7. Supporting References

[1] A. M. F. van der Gracht, M. A. R. de Geus, M. G. M. Camps, T. J. Ruckwardt, A. J. C. Sarris, J. Bremmers, E. Maurits, J. B. Pawlak, M. M. Posthoorn, K. M. Bonger, D. V. Filippov, H. S. Overkleeft, M. S. Robillard, F. Ossendorp, S. I. van Kasteren, *ACS Chem. Biol.* **2018**, *13*, 1569–1576.

[2] M. A. R. Geus, E. Maurits, A. J. C. Sarris, T. Hansen, M. S. Kloet, K. Kamphorst, W. Hoeve, M. S. Robillard, A. Pannwitz, S. A. Bonnet, J. D. C. Codée, D. V. Filippov, H. S. Overkleeft, S. I. Kasteren, *Chem. Eur. J.* **2020**, *26*, 9900–9904.

[3] C. Winzler, P. Rovere, M. Rescigno, F. Granucci, G. Penna, L. Adorini, V. S. Zimmermann, J. Davoust, P. Ricciardi-Castagnoli, *J. Exp. Med.* **1997**, *185*, 317–28.

[4] S. Bolte, F. P. Cordelières, *J. Microsc.* **2006**, *224*, 213–232.

[5] A. J. C. Sarris, T. Hansen, M. A. R. de Geus, E. Maurits, W. Doelman, H. S. Overkleeft, J. D. C. Codée, D. V. Filippov, S. I. van Kasteren, *Chem. Eur. J.* **2018**, *24*, 18075–18081.

[6] K. Bertheussen, M. van de Plassche, T. Bakkum, B. Gagestein, I. Ttofi, A. J. C. Sarris, H. S. Overkleeft, M. van der Stelt, S. I. van Kasteren, *Angew. Chemie Int. Ed.* **2022**, *61*, DOI 10.1002/anie.202207640.

[7] G. A. van der Marel, J. D. C. Codée, P. Kovác, *Carbohydrate Chemistry: Proven Synthetic Methods, Volume 2*, **2014**.

[8] J. Ohlsson, G. Magnusson, *Carbohydr. Res.* **2000**, *329*, 49–55.

[9] H.-M. Chen, S. G. Withers, *Carbohydr. Res.* **2010**, *345*, 2596–2604.

[10] H. Gold, R. G. Boot, J. M. F. G. Aerts, H. S. Overkleeft, J. D. C. Codée, G. A. van der Marel, *European J. Org. Chem.* **2011**, 1652–1663.

[11] G. T. Potter, G. C. Jayson, G. J. Miller, J. M. Gardiner, *J. Org. Chem.* **2016**, *81*, 3443–3446.

[12] R. J. B. H. N. van den Berg, C. G. N. Korevaar, G. A. van der Marel, H. S. Overkleeft, J. H. van Boom, *Tetrahedron Lett.* **2002**, *43*, 8409–8412.

[13] R. J. B. H. N. van den Berg, T. J. Boltje, C. P. Verhagen, R. E. J. N. Litjens, G. a van der Marel, H. S. Overkleeft, *J. Org. Chem.* **2006**, *71*, 836–839.

[14] S. Kim, S. Lee, T. Lee, H. Ko, D. Kim, *J. Org. Chem.* **2006**, *71*, 8661–8664.

[15] L. Panza, F. Compostella, D. Imperio, *Carbohydr. Res.* **2019**, *472*, 50–57.
